# Channel Expansion in the Ligand-Binding Domain of the Glucocorticoid Receptor Contributes to the Activity of Highly Potent Glucocorticoid Analogues

**DOI:** 10.3390/molecules29071546

**Published:** 2024-03-29

**Authors:** Wesley B. Seaton, Susan J. Burke, Alexander R. Fisch, William A. Schilletter, Mary Grace A. Beck, Gabrielle A. Cassagne, Innocence Harvey, Molly S. Fontenot, J. Jason Collier, Shawn R. Campagna

**Affiliations:** 1 Department of Chemistry, University of Tennessee, Knoxville, TN 37996, USA; wseaton2@vols.utk.edu (W.B.S.);; 2 Pennington Biomedical Research Center, Baton Rouge, LA 70808, USAjason.collier@pbrc.edu (J.J.C.)

**Keywords:** glucocorticoid, anti-inflammatory, transcription regulation, mercaptobenzothiazole, molecular dynamics

## Abstract

Glucocorticoids (GCs) act through the glucocorticoid receptor (GR) and are commonly used as anti-inflammatory and immunosuppressant medications. Chronic GC use has been linked with unwanted complications such as steroid-induced diabetes mellitus (SIDM), although the mechanisms for these effects are not completely understood. Modification of six GC parent molecules with 2-mercaptobenzothiazole resulted in consistently less promoter activity in transcriptional activation assays using a 3xGRE reporter construct while constantly reducing inflammatory pathway activity. The most selective candidate, **DX1**, demonstrated a significant reduction (87%) in transactivation compared to commercially available dexamethasone. **DX1** also maintained 90% of the anti-inflammatory potential of dexamethasone while simultaneously displaying a reduced toxicity profile. Additionally, two novel and highly potent compounds, **DX4** and **PN4**, were developed and shown to elicit similar mRNA expression at attomolar concentrations that dexamethasone exhibits at nanomolar dosages. To further explain these results, Molecular Dynamic (MD) simulations were performed to examine structural changes in the ligand-binding domain of the glucocorticoid receptor in response to docking with the top ligands. Differing interactions with the transcriptional activation function 2 (AF-2) region of the GR may be responsible for lower transactivation capacity in **DX1**. **DX4** and **PN4** lose contact with Arg611 due to a key interaction changing from a stronger hydrophilic to a weaker hydrophobic one, which leads to the formation of an unoccupied channel at the location of the deacylcortivazol (**DAC**)-expanded binding pocket. These findings provide insights into the structure–function relationships important for regulating anti-inflammatory activity, which has implications for clinical utility.

## 1. Introduction

Glucocorticoids (GCs) are a class of anti-inflammatory and immunosuppressive steroid ligands that reduce or attenuate the immune response and are thus used to treat various diseases of inflammation [1]. Physiologically, these ligands are synthesized and released from the adrenal cortex and function by binding to the glucocorticoid receptor (GR; NR3C1) [2,3]. Commonly prescribed GCs, such as dexamethasone (**DX**) and hydrocortisone (**HC**), are used pharmacologically in clinical settings despite their risk to induce cardiovascular diseases, obesity, osteoporosis, and diabetes when used long term [4]. Chronic GC use can lead to steroid-induced diabetes mellitus (SIDM), and therefore discovery of a GC capable of reducing these side effects is highly desired [4].

Around 1.2% of the US population use GCs for approximately 1600 days [5]. This clinically prescribed chronic steroid use enhances the risk of developing SIDM. For example, 40% of prednisolone-treated renal transplant patients develop post-transplant diabetes mellitus (PTDM) [6]. Possible mechanisms for diabetes onset include GCs enhancing the gluconeogenesis pathway in the liver, which increases the synthesis of glucose [7]. Additionally, excess GC promotes insulin resistance in liver, white adipose tissue, and skeletal muscle. Attenuating immunosuppression by limiting GC administration has proven difficult due to an increased risk of graft rejection, and thus, GC therapy continues to be one of the best options for transplant patients [8].

The activated GR–GC complex has two main modes of action after translocating to the nucleus. First, GR homodimers that form following ligand binding selectively associate with glucocorticoid-responsive genomic elements (GREs) in the nucleus and regulate gene transcription through transactivation (TA) [9]. Furthermore, TA also induces genes that code for phosphoenolpyruvate carboxykinase (PCK1), which helps maintain glucose homeostasis via gluconeogenesis [7]. The second mode, thought to be responsible for part of the anti-inflammatory activity of GCs, is termed transrepression (TR) and results from the interaction of the monomer ligand-bound GR complex with transcription factors (TFs) that normally respond to inflammatory cytokines such as IL-1β [10]. TR can lead to direct anti-inflammatory activity. However, this paradigm is more nuanced as TA of genes such as *Dusp1/Mkp1*, *Rgs2*, and *Tsc22d3* have positive therapeutic impact-inducing broncho protection and decreased CCL2 expression, respectively [11,12,13].

Despite the structural diversity of commercially available GCs, none are presently capable of dissociating the two modes of transcription. However, a significant effort has been made with the aim of developing such a selective glucocorticoid receptor modulator (SGRM). For example, substitution of the C-21 hydroxyl group on **HC** with 2-mercaptobenzothiazole (MBT) has been shown to reduce impact on islet β-cell insulin secretion while maintaining anti-inflammatory activity [14]. Such thiazole moieties are known to exhibit unique medicinal properties as they contain both electron-accepting and -donating groups [15]. Interestingly, further modification of this compound through esterification of C-17 with 2-furoyl chloride decreased selectivity due to enhanced TA. This result was expected as furoate ester has proven to dramatically increase potency of several GCs used in treating asthma such as fluticasone furoate (**FF**), mometasone furoate (**MF**), and drug candidates such as VSGC12 [16]. Specifically, VSGC12 induced TR activity at one-fourth the concentration of **FF** while reducing insulin resistance. Accessing this “dosage window”, wherein concentrations are low enough to induce TR but not TA, is a unique approach to selectively modulate the GR. Thus, a specific combination of these functional groups has the potential to produce a powerful SGRM.

**Figure 1:**
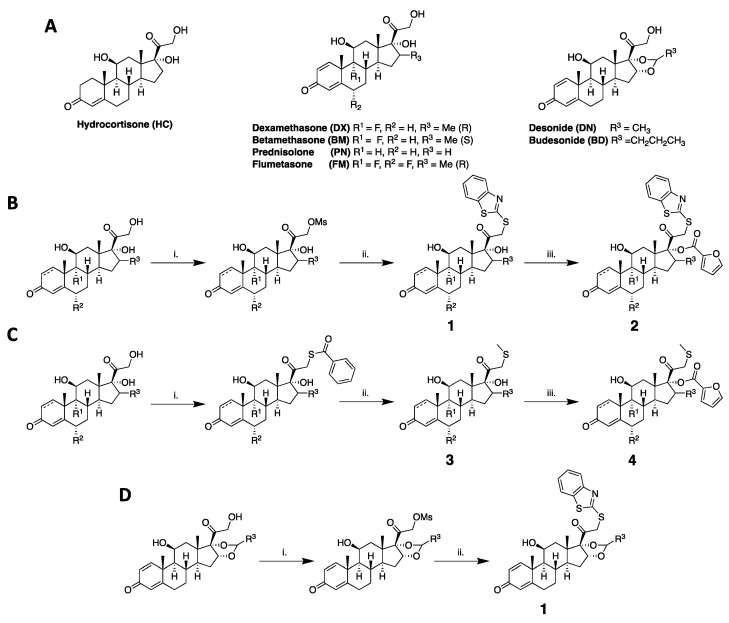
(**A**) Starting glucocorticoid structures. (**B**) Synthetic route for benzothiazole analogues **1** and **2**. Reagents and conditions: (i) methanesulfonyl chloride, DIPEA, CH_2_Cl_2_, 0 °C to rt, 15 h, (ii) 2-mercaptobenzothiazole, K_2_CO_3_, acetone, reflux, (iii) furoyl chloride, DMAP, CH_2_Cl_2_, 0 °C to rt. (**C**) Synthetic route for thiomethyl analogues **3** and **4**. Reagents and conditions: (i) diisopropyl azodicarboxylate, PPh_3_, thiobenzoic acid, THF, 0 °C to rt, 2.5 h, (ii) 1 N NaOH, iodomethane, MeOH, 3.5 h, (iii) furoyl chloride, DMAP, CH_2_Cl_2_, 0 °C to rt. (**D**) Synthetic route for desonide and budesonide analogues 1. Reagents and conditions: (i) methanesulfonyl chloride, DIPEA, CH_2_Cl_2_, 0 °C to rt, 15 h, (ii) 2-mercaptobenzothiazole, K_2_CO_3_, acetone, reflux.

Herein, a total of 24 compounds were synthesized by derivatizing seven common GC scaffolds currently prescribed for human inflammatory conditions (Figure 1A). To understand how these structural modifications altered activity, a series of biological assays were performed to evaluate promoter activity, gene expression, and cellular toxicity. Molecular dynamic (MD) simulations and subsequent computational analyses were performed on the top structures to understand their structure–activity relationships (SARs) within the GR ligand-binding domain. Importantly, a highly selective GC, **DX1**, demonstrated a significant reduction (87%) in TA compared to commercially available **DX** while maintaining TR capacity. Furthermore, two novel, highly potent GCs, **DX4** and **PN4**, elicited GR activity in the attomolar range similar to that of their parent scaffolds, **DX** and **PN**, at the nanomolar and micromolar ranges, respectively. Possible explanations for their altered activity were provided by molecular docking and dynamic simulations. Collectively, these compounds and their unique modes of action could prove useful in understanding how to improve GC-based therapies.

## 2. Results

### 2.1. Series **1** GC Analogues Selectively Repress Ccl2 Promoter Activity without Inducing Transactivation

**Figure 2:**
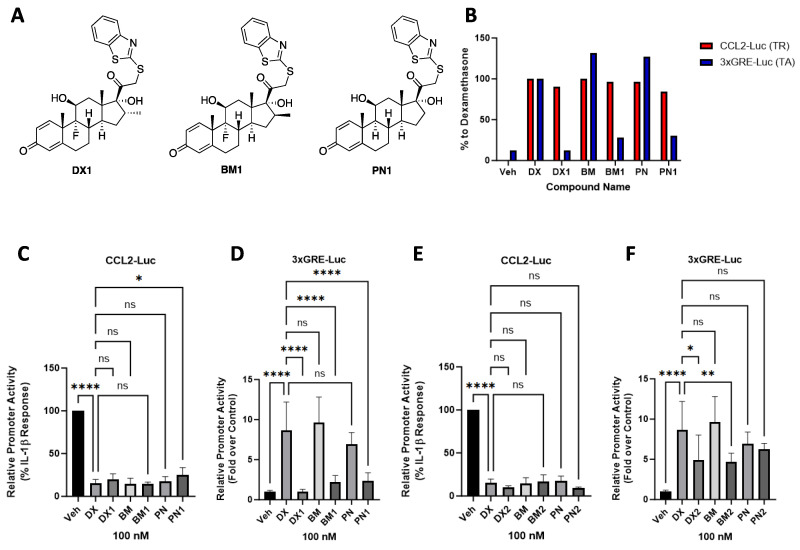
Bioassay data showing relative steroid activity of selected series **1** and **2** compounds. (**A**) Structure of compounds **DX1**, **BM1**, and **PN1**. (**B**) GR-ligand activity in CCL2-Luc (TR) and 3xGRE-Luc (TA) assays as a percent response against dexamethasone at 100 nM for selected compounds. (**C**) CCL2-promoter-luciferase-reporter activity assay; *x*-axis, selected series **1** and their respective parent compound activity at 100 nM; *y*-axis, %-maximal IL-1β response (relative promoter activity). (**D**) 3xGRE-promoter-luciferase-reporter activity assay; *x*-axis, selected series **1** and their respective parent compound activity at 100 nM; *y*-axis, fold over control. (**E**) CCL2-promoter-luciferase-reporter activity assay; *x*-axis, selected series **2** and their respective parent compound activity at 100 nM; *y*-axis, %-maximal IL-1β response (relative promoter activity). (**F**) 3xGRE-promoter-luciferase-reporter activity assay; *x*-axis, selected series **2** and their respective parent compound activity at 100 nM; *y*-axis, fold over control. Data are means ± S.D. (*error bars*). Not significant (ns), *p* > 0.05 vs. vehicle (DMSO); *, *p* < 0.05 vs. vehicle (DMSO); **, *p* < 0.01 vehicle (DMSO); ****, *p* < 0.0001 vs. vehicle (DMSO). Note: Statistical analyses for all pairwise comparisons among the concentrations and vehicle can be found in Supplementary Materials, Sections S4 and S5. This is a figure. Schemes follow the same formatting.

A promoter–luciferase construct containing three copies of the glucocorticoid-response element (3xGRE-Luc) was transfected into 832/13 rat insulinoma cells to assess TA of each compound. Concurrently, the ability of all compounds to repress IL-1β-mediated activation of a promoter–luciferase construct containing 3.6 kb of the CCL2 promoter (CCL2-Luc), was investigated to determine the TR capability of each compound. pIC_50_ and pEC_50_ values were calculated for all compounds that exhibited a dose-dependent response (Table 1). The most selective compound, **DX1**, showed only 13% transcriptional activity of the 3xGRE-Luc when compared with **DX** while maintaining 90% of its TR activity (Table 1). However, furoylation to **DX2** restored 50% TA response in 3xGRE-Luc when compared to **DX**. Repression of IL-1β-induced CCL2 promoter activity by **DX2** was virtually unchanged when compared with **DX1**. Diminished TA of the 3xGRE-Luc and greater discrimination between TR and TA was noted across all steroid scaffolds in series **1** (Figure 2B). For example, at 100 nM, **BM** and **BM1** reduced IL-1β response by 88.7% and 85.7%, respectively (Figure 2C), while only **BM1** selectively reduced 3xGRE activation by 4.6-fold when compared with **BM** (Figure 2D). **PN1** displayed similar selectivity but did lose overall potency in the CCL2-Luc assay with only a 75% response at 100 nM (Figure 2C,D). Furoylation reinstated TA potential across all series **2** compounds, giving them similar activity to their parent steroidal compounds (Figure 2E,F).

### 2.2. DX4 and PN4 Display Attomolar Potency versus Nanomolar Potency for Parent Scaffolds

The addition of an SMe group to the parent steroids instead of MBT led to analogues in series **3**. These served to illuminate the isolating effects sulfur has on potency in the absence of benzothiazole. Furthermore, the addition of this smaller, less nucleophilic group at C-21 allowed for further investigation into the properties of the furoyl group in series **4**. All SMe analogues in series **3** demonstrated a moderate decrease in anti-inflammatory potential when compared with their parent molecules, and the impact on TA potential was negligible in most cases (Table 1). For example, **DX3** induced only 84% suppression of the CCL2-Luc when compared with **DX** while maintaining 94% of its TA activity in 3xGRE-Luc. Trends between series **3** and **4** were not as consistent as anticipated based on the activity of compounds from series **1** and **2**. Some compounds were more active in both luciferase assays, with **DX4** and **PN4** both inducing more TR and TA when compared with **DX3** and **PN3**, respectively. Specific compounds showed an increase in anti-inflammatory activity and decrease in potential side effects upon furoylation of the SMe derivate. For example, at 0.1 nM, the CCL2-Luc assays of **PN4** and **DX4** showed a 74% and 81% anti-inflammatory response, respectively, along with a lowering of the pIC_50_ to greater than 10. For comparison, at the same concentration, PN and DX elicited only 13% and 45% response with pIC_50_ values of 8.4 and 8.3, respectively (Table 1). Additionally, **PN4** displayed reduced activation with an E_max_ of 5.4 in the 3xGRE-Luc assay compared to **PN** with an E_max_ of 10 (Table 1).

**Figure 3:**
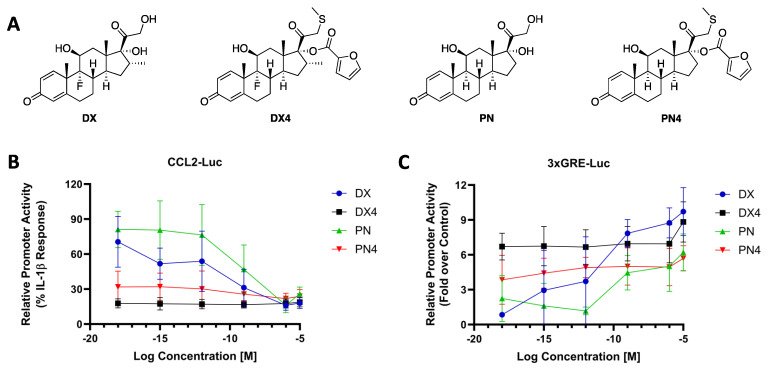
Dose-response curve of the transcription activities of GCs. (**A**) Structures of highly potent compounds **DX4** and **PN4** and their respective parent compounds. (**B**) CCL2-promoter-luciferase-reporter activity assay; *x*-axis, log concentration (molar); *y*-axis, %-maximal IL-1β response (relative promoter activity). (**C**) 3xGRE-promoter-luciferase-reporter activity assay; *x*-axis, log concentration (molar); *y*-axis, fold over control.

Intrigued by these results, **DX4** and **PN4** were further diluted to 10^−18^ M (1 aM) and tested in both CCL2 and 3xGRE luciferase assays and compared with their parent compounds, **DX** and **PN** (Figure 3A). **DX** and **PN** induced little to no significant reduction in CCL2 promoter activity at 1 aM with 29% and 19% anti-inflammatory activity, respectively (Figure 3B). By contrast, **DX4** and **PN4** maintained consistently high TR capabilities, demonstrating an 82% and 68% reduction at the same concentration, respectively. Furthermore, **DX4** and **PN4** displayed high potency as they prompted a 6.7- and 3.8-fold increase in the 3xGRE assay at 1 aM (Figure 3C). Interestingly, **DX4** and **PN4** did not exhibit a typical dose-dependent response. When plotting TR and TA promoter activity over the concentrations tested, **DX** and **PN** exhibited a gradual increase in activity that was dependent on the concentration, while **DX4** and **PN4** displayed less dependence on concentration.

### 2.3. DX1 and DX4 Exhibit Reduced Toxicity When Compared with DX

**Figure 4:**
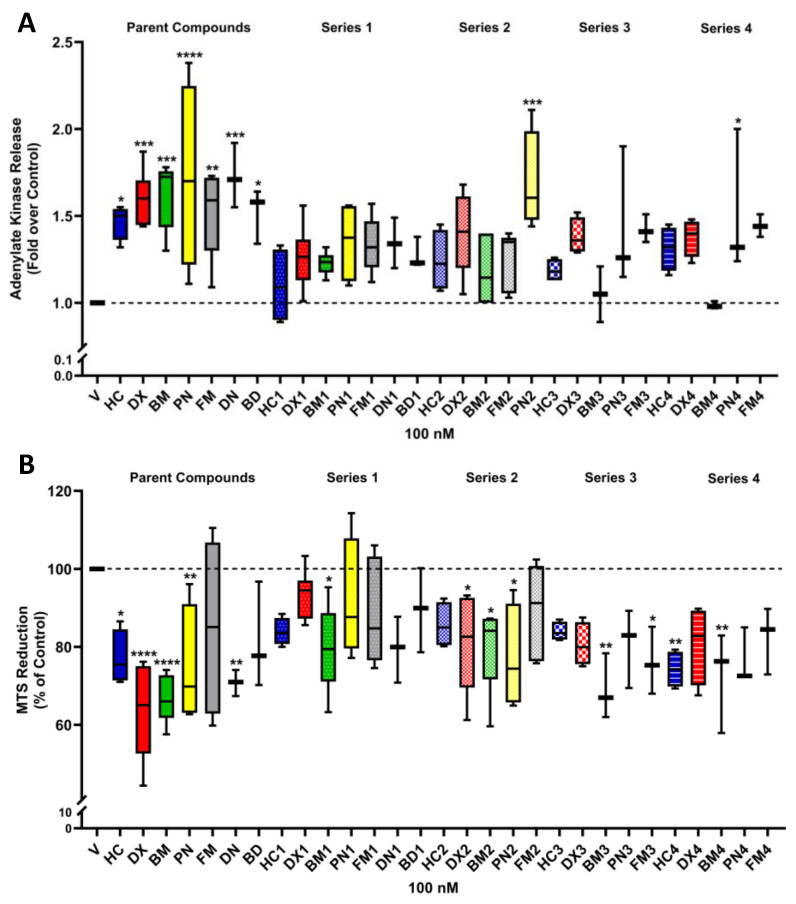
Toxicity assays of 832/13 cells treated with 100 nM concentrations of GC. (**A**) Adenylate kinase release into culture medium after exposure to indicated GC. (**B**) MTS reduction after exposure to indicated GC. *, *p* < 0.05 vs. vehicle (DMSO); **, *p* < 0.01 vs. vehicle (DMSO); ***, *p* < 0.001 vs. vehicle (DMSO); ****, *p* < 0.0001 vs. vehicle (DMSO). Note: For this analysis, all steroids were included in the analysis, and intergroup comparisons were performed using simple one-way ANOVA comparison of each steroid against vehicle control. Data in the Supplementary Materials for this assay only include intragroup comparisons; and therefore, the *p*-values differ among the statistical treatments available in Supplementary Materials, Sections S6 and S7.

GCs can promote cellular toxicity in pancreatic β-cells [17]. To determine the impact on cellular viability during exposure to these compounds, ADK release and MTS reduction were measured as previously described [18]. Release of ADK was measured to determine losses in cellular membrane integrity, and MTS reduction measures cellular respiration activity, which could be taken as an index of proliferation, viability, or both. At 100 nM, nearly all modified GCs displayed a reduced toxicity profile in both assays. For example, **DX** generated a 1.6-fold increase in ADK release, while **DX1**, **DX2**, and **DX4** produced a 1.25, 1.31, and 1.38 increase, respectively, indicating decreased cytotoxicity compared to **DX** (Figure 4A). Further, **DX** promoted a 37% reduction in the MTS assay, **DX1**, **DX2**, and **DX4** induced only a 7%, 19%, and 19% reduction, respectively (Figure 4B). No ADK release was detected for **BM4** when compared with the control vehicle DMSO; note that this is a 60.7% improvement over **BM** toxicity. **BM2** outperformed **BM4** in the MTS assay with only 20.3% reduction against **BM4**′s 27.6% reduction (Figure 4B). Only **HC4** and **FM3** showed potential impacts on viability comparable to the parent scaffolds, and these molecules elicited MTS reduction responses within 2.9% and 8.7% of those measured for the unmodified steroids, respectively (Table S4). Finally, **PN** induced the greatest change of all steroids with a 1.72-fold increase in ADK release and 25.4% MTS reduction. In contrast, the addition of MBT to the **PN** structure (**PN1**) reduced ADK release to 1.25-fold and only decreased MTS reduction by 8.3%.

### 2.4. Sgk1 and Rgs2 Gene Expression Induced at Low Concentrations in Response to DX4 and PN4, but Decreased upon MBT Addition

**Figure 5:**
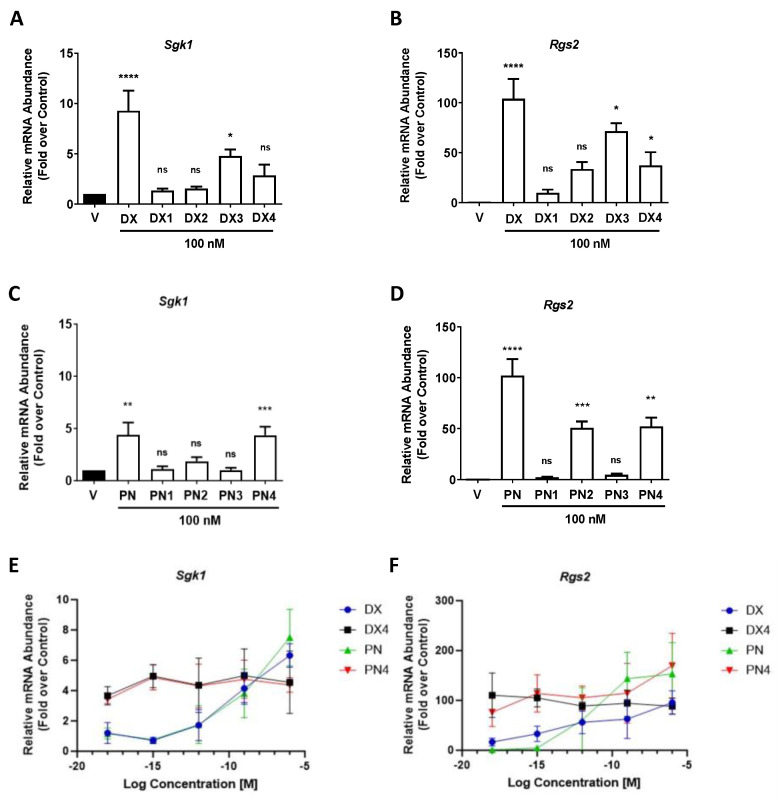
832/13 cells treated with 100 nM concentrations of GCs. (**A**) *Sgk1* and (**B**) *Rgs2* gene expression associated with transactivation after exposure to dexamethasone and its analogues. (**C**) *Sgk1* and (**D**) *Rgs2* gene expression associated with transactivation after exposure to prednisolone and its analogues. Dose-response curve of the transcription activities of highly potent GCs. Not significant (ns), *p* > 0.05 vs. vehicle (DMSO); *, *p* < 0.05 vs. vehicle (DMSO); **, *p* < 0.01 vs. vehicle (DMSO); ***, *p* < 0.001 vs. vehicle (DMSO); ****, *p* < 0.0001 vs. vehicle (DMSO). (**E**) *Sgk1* gene expression; *x*-axis, log concentration (molar); *y*-axis, relative mRNA Abundance (fold over control). (**F**) *Rgs2* gene expression; *x*-axis, log concentration (molar); *y*-axis, relative mRNA Abundance (fold over control).

To determine GC impact on GR target genes, relative mRNA levels of *Sgk1* and *Rgs2* were measured. Upregulation of serum- and glucocorticoid-inducible kinase-1 (*Sgk1*) has been linked to GC-mediated impairment of insulin secretion [19]. At 100 nM concentrations, the expression of *Sgk1* is strongly activated by **DX** in 832/13 cells (7.9-fold). By contrast, **DX1** and **DX2** demonstrated no significant induction of *Sgk1*-relative mRNA abundance relative to vehicle control (Figure 5A). The largest activity change between any analogues and their parent molecule was observed for **BM** and **BM1** with *Sgk1* mRNA abundance in **BM** increased by 8-fold and with no induction of *Sgk1* mRNA abundance observed in response to treatment with **BM1** (Table S7A). The regulator of G-protein signaling 2 (*Rgs2*) gene encodes the *Rgs2* protein, and its increased abundance is linked with cardiac hypertrophy [11]. At a concentration of 100 nM, the relative mRNA abundance of *Rgs2* in response to **DX1** treatment was 2.2% that of **DX** (Figure 5B), and **DX2** prompted 4.3% of the response seen with **DX**. Of note, the most dissociated compound in this assay was **PN1**, demonstrating no significant induction of *Sgk1* mRNA abundance and only 1.9% of the activity when compared with **PN** increases in *Rgs2* transcript levels (Figure 5C,D).

**DX**, **DX4**, **PN**, and **PN4** were diluted to 10^−18^ M and tested for their capacity to induce transcription of *Sgk1* and *Rgs2*. At 1 aM concentrations, **DX4** and **PN4** induced *Sgk1* mRNA abundance by 3.7- and 3.4-fold, respectively (Figure 5E). Significantly, *Rgs2* mRNA abundance was 6.6-fold greater for **DX4** than for **DX** at 1 aM and nearly 75-fold greater for **PN4** than **PN** (Figure 5F). Clear dose-dependent responses can once again be observed in both TA assays for **DX** and **PN**, while **DX4** and **PN4** gene expression profiles stay relatively constant at all concentrations tested.

### 2.5. All GC Analogues, Except HC3, Reduce Ccl2 or Ccl20 Transcript and Protein Abundance

**Figure 6:**
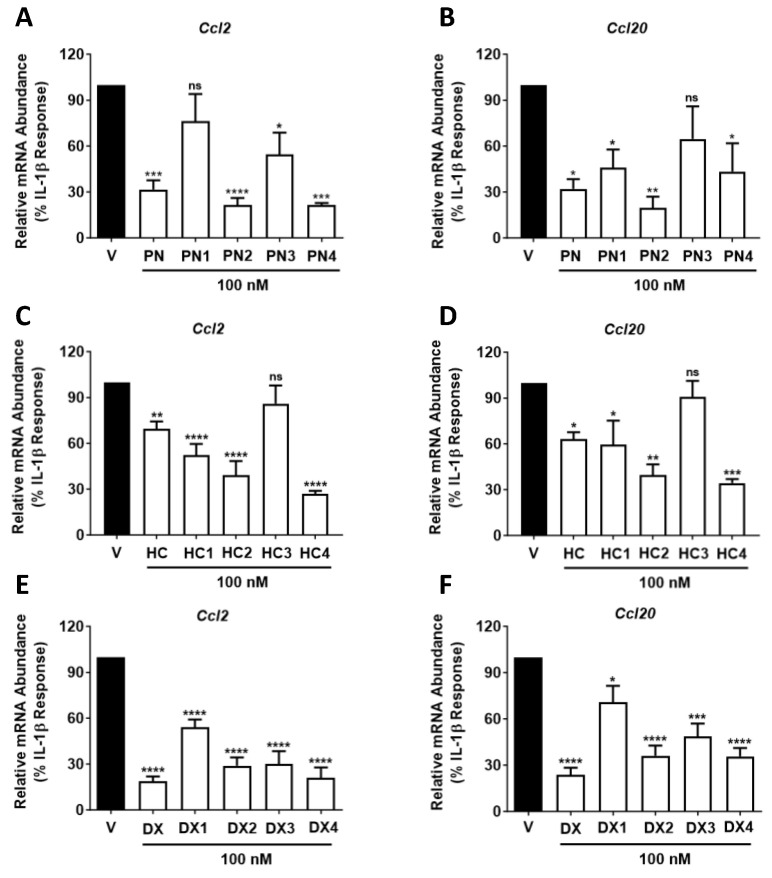
832/13 cells treated with 100 nM concentrations of GCs. (**A**) *Ccl2* and (**B**) *Ccl20* relative mRNA release into medium in response to **PN**-**PN4** versus DMSO (black bar). (**C**) *Ccl2* and (**D**) *Ccl20* relative mRNA release into medium in response to **HC-HC4**. (**E**) Ccl2 and (**F**) *Ccl20* relative mRNA release into medium in response to **DX**-**DX4**. Not significant (ns), *p* > 0.05 vs. vehicle (DMSO); *, *p* < 0.05 vs. vehicle (DMSO); **, *p* < 0.01 vs. vehicle (DMSO); ***, *p* < 0.001 vs. vehicle (DMSO); ****, *p* < 0.0001 vs. vehicle (DMSO).

*Ccl2* and *Ccl20* are both chemokines responsible for recruiting immune cells to sites of inflammation [13,20,21]. The ability of each derivatized compound to repress cytokine-induced expression of the *Ccl2* and *Ccl20* genes was therefore evaluated. The IL-1β-mediated increase in *Ccl2* mRNA was diminished by all compounds except **HC3** and **PN1** at 100 nM, with **PN2** having the largest impact with an 87% decrease compared to vehicle control (Figure 6A). In addition to **PN2**, **HC4** was the only compound to display increased anti-inflammatory activity compared to its parent molecule at this concentration, with a 73% reduction in *Ccl2* expression compared with the 31% decrease observed in response to **HC** (Figure 6C). **DX4** and **PN4** each decreased *Ccl2* mRNA at 100 nM by 79% (Figure 6E), while **HC3** was the least effective compound, reducing *Ccl2* mRNA by only 14%. Furthermore, the effects of all compounds on *Ccl20* transcript levels were also assessed. The chemokine *Ccl20* is of particular interest due to its known upregulation in non-obese diabetic mice (NOD) [22], a T1D model, and db/db mice, a T2D model [18,20]. **PN2**, **HC4**, and **BM2** all displayed stronger anti-inflammatory activity than their parent molecules by suppressing *Ccl20* mRNA abundance by 81%, 66%, and 79%, respectively, at 100 nM concentrations (Figure 6B,D). By contrast, **PN**, **HC**, and **BM** only suppressed *Ccl20* mRNA abundance at the same concentration by 68%, 63%, and 21%, respectively. No analogues of **DX** outperformed their parent compounds (Figure 6F), and, once again, **HC3** displayed significantly reduced potency with only a 9% decrease in *Ccl20* mRNA levels compared to the vehicle control (Figure 6D).

### 2.6. Weakened Interaction between DX1 and AF-2 Domain of GR

**Figure 7:**
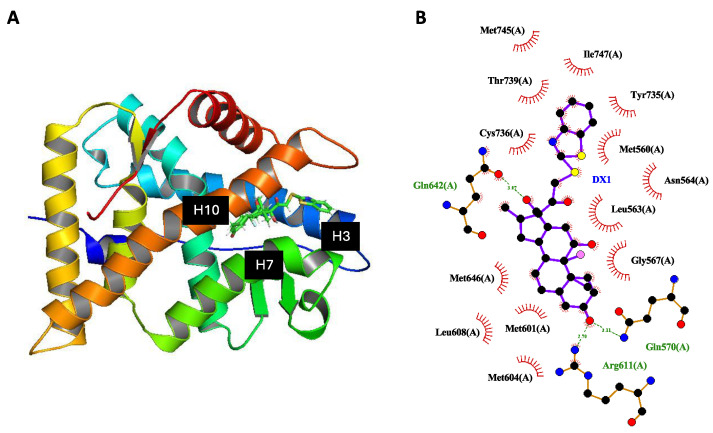
(**A**) Molecular docking experiment with **DX1** inserted into LBD1 of 7prv. (**B**) 2D representations of the ligand–receptor interactions. Red half-circles represent hydrophobic interactions, and the green dotted line represents a hydrogen bond.

In total, **DX1** displayed 13 hydrophobic and 3 polar interactions. The MBT moiety of **DX1** extended into a region between helix 3 (H3), helix 7 (H7), and helix 10 (H10) of the LBD which is not occupied by **DX** (Figure 7A). Despite entering this new space, many of the interactions previously reported for crystal structures of **DX** were retained by **DX1** [23,24]. Indeed, when comparing the amino acid interactions of **DX1** with **DX** (PDB ID: 4udc), 13 out of 16 total interactions were identical (Figure 7B). Interestingly, **DX1** displayed 3 additional hydrophobic interactions with Met745, Met646, and Leu608. Furthermore, residues of H3, helix 4 (H4), and helix 12 (H12) of the LBD together form a mostly hydrophobic area termed the ligand-dependent transcriptional activation function 2 (AF-2), which has been associated with conformational changes in the GR [25]. Within H3, Asn564 stabilizes the AF-2 domain [26,27]. This interaction is weakened upon addition of MBT as it changes from polar to hydrophobic, which may contribute to the improved selectivity in **DX1**.

### 2.7. MBT Structure Selectively Modulates the GR

Because the present results did not fully explain the selective nature of **DX1**, the presence of the MBT moiety was further probed through the synthesis and biological evaluation of 4 additional dexamethasone analogues (Table 2). **DX5**–**DX8** were synthesized utilizing mercaptobenzimidazole (MBI), mercaptobenzoxazole (MBO), mercaptoimidazole (MI), and mercaptothiazole (MT) (Table 2). This slight modification proved to generate a wide range of activity in the 3xGRE-luciferase and CCL2-luciferase assays. To begin, **DX5**–**DX8** all displayed reduced anti-inflammatory activity when compared with **DX** and **DX1**. **DX5** and **DX6** exhibited little selectivity inducing TA with an E_max_ of 3.2 and 4.9, respectively. Interestingly, at lower concentrations, **DX7** induced TA preferentially over TR with a pIC_50_ of 7.1 and pEC_50_ of 7.7. **DX8** did display discrimination between modes of activity with 30% transcriptional activity of the 3xGRE-Luc when compared with **DX**. However, the overall potency of the compound dropped along with the loss of the benzene ring.

### 2.8. DX4 and PN4 Expand the Glucocorticoid Receptor Ligand-Binding Pocket

**Figure 8:**
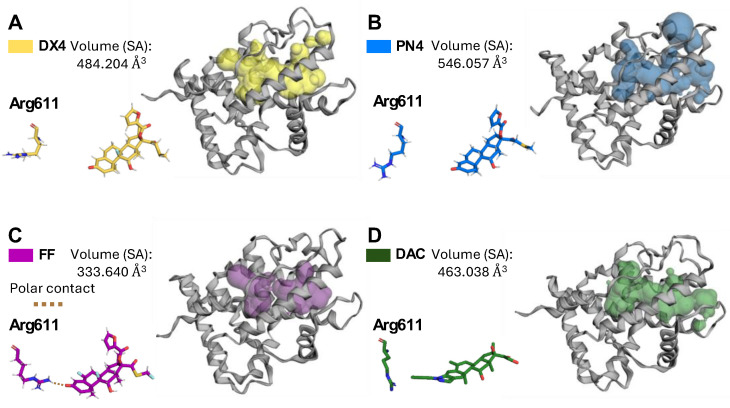
Ligand-binding pocket created in response to GR agonists. Wire represents the protein, and the solid surface represents the ligand-binding pocket area. The red line is placed in the same spot for all structures for scale and reference. (**A**) Ligand-binding pocket and interaction between Arg611 for **DX4** (yellow), (**B**) **PN4** (blue), (**C**) **FF** (purple), and (**D**) **DAC** (green).

**DX4** displayed a total of 13 interactions, all of which were hydrophobic (Supplementary Materials, Section S3). This resulted in a decrease in binding affinity from −12.2 to −10.2 kcal/mol (Table S9). **PN4** displayed the exact same interactions but with three additional hydrophobic interactions. The furoyl group present in **FF**, **DX4**, and **PN4** expanded the LBP and occupied the hydrophobic cavity, as previously described in the literature [16]. Furthermore, the interaction with Asn564 changed from hydrophobic to polar with **PN4**. Two of the most common polar interactions among GCs, the 3-keto group with Arg611 and Gln570, are absent for **DX4** and **PN4**. Arg611 rotates away over time in response to **DX4** and **PN4**, and the interaction never returns (Figure 8A,B). As a result, there is a continuous binding pocket channel formed in response to **DX4** and **PN4** that expands beyond what is typical for **FF** (Figure 8A–C). Indeed, **FF’s** calculated solvent-accessible (SA) pocket volume was ~334 Å^3^, while **DX4** and **PN4** expanded the pocket’s volume to ~484 Å^3^ and ~546 Å^3^, respectively. Increased potency associated with this type of binding pocket expansion along with loss of polar contact with Arg611 has been previously observed with agonist deacylcortivazol (**DAC**), which exhibited a calculated SA pocket volume of ~463 Å^3^ (Figure 8D) [28]. The results of these volume calculations are consistent with prior research, although the magnitudes of the pocket volumes are smaller. Differences can be rationalized due to the use of larger radius probes, different software, and lack of manual refinements to the protein structure. Calculated molecular and SA surface volumes for compounds are available in Table S10.

## 3. Discussion

Glucocorticoids represent a large class of lipophilic steroid molecules frequently used in clinical settings for their anti-inflammatory and immunosuppressive activity [29]. However, their prolonged use leads to a vast array of side effects due to their impact on glucose and lipid homeostasis [4]. A group of MBT-modified glucocorticoids were synthesized and evaluated for their ability to distinguish between TA and TR modes of action. This work reports data for analogues of seven commercially available steroids. All of the MBT compounds, **DX1**, **BM1**, **PN1**, **FM1**, **BD1**, and **DN1**, displayed greater signs of selectivity with reduced TA activity. The top-performing compound, **DX1**, maintained ~90% TR activity of **DX** with only ~13% of the TA activity while simultaneously displaying diminished toxicity in both the MTS and ADK assays.

**BM1**, **PN1**, **FM1**, **BD1**, and **DN1** all showed similar, though less profound, distinctions between modes of activity when compared with their parent molecules. The addition of MBT to all parent scaffolds markedly increased their tolerability in both toxicity assays while decreasing their TA potential. Specifically, **PN1** became the second most tolerable compound in the MTS assay and did not induce expression of the *Sgk1* or *Rgs2* genes. Whether a decrease in *Rgs2* mRNA abundance may counteract the therapeutic effects associated with **PN**, such as improved broncho-protection [11], remains to be established. Additionally, the C-16 stereoisomer of **DX1**, **BM1**, displayed 96% of the TR activity of **DX** with only ~28% TA, making it a potentially valuable alternative to **DX1**. Interestingly, further modification with a furoyl moiety on all the compounds with a C-17 hydroxyl resulted in decreased discrimination between TA and TR while increasing the TR activity in the CCL2-Luc assays of all compounds, except for **BM2**.

The combination of molecular docking and dynamics was used to obtain a more precise picture of ligand–protein interaction between **DX1** and the GR. The MBT moiety expanded into an open region of the GR between H3, H7, and H10 not occupied by **DX**. Despite entering this new space, **DX1** maintained many of the same amino acid interactions as **DX**. However, 2D analysis revealed that **DX1** displayed a hydrophobic interaction with a key amino acid associated with the AF-2 domain, Asn564, while **DX** displays a stronger polar interaction. The weakening of this contact may lower the TA capacity of **DX1** and be partly responsible for changing the ligand activity profile. Further, by synthesizing new analogues and selectively exchanging the various heteroatoms of MBT, as well as removing the benzene ring, we were able to determine that the sulfur in the thiazole ring of **DX1** may help alter modes of action (TA vs. TR) while the benzene ring contributes to its anti-inflammatory potential. However, these results would require further testing to prove definitively.

Despite the decrease in efficacy from series **1** to series **2**, the addition of the furoyl moiety increased the efficacy of all compounds when added to series **3** analogues. Specifically, **DX4** and **PN4** displayed the most profound activity as they induced the expression of two separate genes, *Sgk1* and *Rgs2*, at attomolar concentrations. To our knowledge, this is the first study to document the uniquely potent transcriptional potential of **DX4** and **PN4**, which, in general, were able to induce the same activity as **DX** at one billion- and one millionfold lower concentrations, respectively. It is also the first to report a change in the channel formation of the GR LBP in the absence of a bulky group adjacent to the A ring. However, these new findings are supported by previous work that has shown that ligands, such as **DAC**, which expand the binding pocket, often show activity greater than endogenous steroidal hormones [28]. For **DX4** and **PN4**, an open channel through the binding pocket near the A ring is observed. This channel, combined with the hydrophobic cavity occupied by the furoyl moiety, allows **DX4** and **PN4** to occupy a greater total volume than both **DAC** and **FF**. Thus, this enhanced agonist activity, combined with a newly proposed mode of action, makes these results potentially useful in developing and designing better GR agonists.

## 4. Materials and Methods

### 4.1. Synthesis

Compounds **1**–**4** were synthesized with slight modifications to previously published methods (Figure 1B–D) [14,30,31]. Six different GCs (Figure 1A) were chosen to be derivatized with MBT and both MBT and furoyl to explore how these modifications to the steroidal core impact activity (series **1** and **2**). Unfortunately, the addition of furoyl moiety alone proved synthetically difficult due to cleavage of the furoyl moiety upon removal of C-21 protecting groups, which is potentially promoted by the unmasked oxygen on the side chain that is five atoms away from the furoyl ester carbonyl. Therefore, to investigate the impact of furoyl ester alone, a thiomethyl (SMe) group was introduced at C21 on **HC**, **DX**, **BM**, **PN**, and **FM**, affording series **3**. Subsequent furoylation of these analogues with a catalytic amount of DMAP to activate the furoyl chloride yielded series **4**. **DX5**–**DX8** were synthesized with the appropriate mercapto reagent using the same procedure as series **1**. Complete synthetic procedures and characterizations for all compounds can be found in Supplementary Materials, Sections S1 and S2.

### 4.2. Biological Assays

All in vitro assays were performed using the 832/13 rat insulinoma cell line, which was confirmed to be free of mycoplasma [32]. Cells were cultured in either 12- or 24-well plates, and treated at 80–95% confluence. The 832/13 cells were exposed to the steroidal compounds for 24 h at concentrations indicated in the figure legends. Using both 3-(4,5-dimethylthiazol-2-yl)-5-(3-carboxymethoxyphenyl)-2-(4-sulfophenyl)-2H-tetrazolium (MTS) and adenylate kinase (ADK) assays, cell viability was assessed as described previously [18]. The ability of compounds to repress IL-1β-mediated increases in pro-inflammatory promoter activity was examined using the previously reported CCL2-promoter–luciferase plasmid construct (CCL2-Luc) [13]. The 832/13 cells were transfected with the CCL2-Luc plasmid. At 24 h post-transfection, cells were treated with the concentrations of each compound shown in the figure legends for 1 h, followed by stimulation with 1 ng/mL of IL-1β for 4 h. Furthermore, transactivation activity of the synthesized compounds was determined using plasmid constructs containing three copies of a glucocorticoid-responsive element (3xGRE-Luc) [14]. The 832/13 cells were transfected with the 3xGRE luciferase reporter plasmid. At 24 h post-transfection, the cells were exposed to each synthetic compound at the concentrations shown in the figure legends for 4 h. At the end of each respective treatment period, the cells were harvested using passive lysis buffer (Promega, Madison, WI) and luciferase activity measured in a Glomax luminometer (Promega, Madison, WI, USA). Luciferase activity was normalized to total protein to account for differences in cell numbers between experiments. The ability of each compound to suppress transcription induced by IL-1β was also assessed by examining gene expression of *Ccl2* and *Ccl20*, two distinct chemokine genes known to be upregulated in mouse and human islets exposed to cytokines [20]. The 832/13 cells were cultured in 12-well plates and were treated for 1 h with each compound, then stimulated for a further 3 h with 1 ng/mL IL-1β. To investigate expression of GR target genes, 832/13 cells were treated with each compound for 6 h. Cells were lysed with TRI Reagent (Sigma, St. Louis, MO, USA) and RNA-extracted, cDNA-synthesized, and mRNA expression levels were determined as previously described [33]. Transcript levels were normalized to the housekeeping gene, Ribosomal S9 (*Rs9*). Primer sequences of *Rs9*, *Ccl2*, *Ccl20*, *Rgs2*, and *Sgk1* are available upon request. Parental compounds were purchased from Sigma Aldrich (St. Louis, MO, USA). For low-dose experiments, Dexamethasone and Prednisolone were purchased from two vendors (Sigma Aldrich and Selleck Chemicals LLC; Houston, TX, USA), and an equal number of replicates were performed with compounds from each vendor.

### 4.3. Molecular Docking and PDB Rational

Computational experiments and analysis tools were used to probe the interactions between the top 3 ligands, **DX1**, **DX4**, and **PN4**, and the GR. Using the GR LBD bound to **FF** (PDB ID: 7prv), molecular docking was performed. This crystal structure was chosen because its native ligand, **FF**, is highly similar to two of the three compounds of interest, **DX4** and **PN4**. **FF**, **DX4**, and **PN4** all have three atoms after the C-18 carbonyl, and all possess the furoate ester group branching off C-17. Together, this increased the accuracy of docking results. Furthermore, 7prv is one of the newest crystal structures of the GR and is the first to show the quaternary structure of the GR [34]. The first LBD (LBD1) of this structure was isolated in PyMol (Schrödinger) and used for molecular docking (residues 528–776) [35]. Docking was performed using AutoDock Vina (The Scripps Research Institute) [36]. Excess water molecules and the starting compound were removed from the GR LBD1 in order to prepare the complex for the docking study. The energy of the ligands was optimized in Chem3D prior to docking between the flexible ligand and the rigid 7prv LBD1 protein. LigPlot+ (European Molecular Biology Laboratory) was used to analyze and compare ligand–residue interactions [37]. This procedure was repeated with **DAC** in its native receptor (PDB ID: 3bqd), and initial docking results of native ligands, **FF** and **DAC**, in LBD1 and 3bqd, respectively, validated the docking procedure as the ligands maintained their position within the receptor (Figure S1).

### 4.4. Molecular Dynamic Simulations

Our previous efforts used AutoDock Vina (The Scripps Research Institute) exclusively [36]. However, AutoDock Vina only allows movement of ligand position and atoms inside a static protein. This resulted in relatively weak and potentially unrealistic positioning and low binding affinities of **DX1** due to an inability to accommodate the added steric bulk (Table S9). Therefore, AutoDock Vina was only used to insert the ligands into the receptor and to calculate binding energies before and after MD simulations, while GROMACS [38] was used to obtain more accurate models that were constructed with allowances for protein backbone flexibility. The resulting changes in amino acid positions in response to the synthesized compounds resulted in an optimally positioned ligand within the GR. **DX1**, **DX4**, and **PN4** protein–ligand complexes underwent 100 ns molecular dynamic (MD) simulations using GROMACS software version 2021.5-gcc and CHARMM36 force field [38,39]. **FF** and **DAC** were also simulated in their native receptors (PDB ID: 7prv and 3bqd, respectively) in order to validate results and draw conclusions. Only LBD1 of 7prv was used for the simulations of synthesized ligands and **FF**. The hinge region (residues 489–527) is unresolved, and connectivity between each LBD and DBD of the crystal structure 7prv is based on proximity alone [34]. Between generating force fields for unresolved amino acids, assuming connectivity between domains, and allocating the required amount of computational resources required to simulate the entire structure, we elected to use only this single LBD for analysis. Additionally, full protein changes were out of scope due to interest primarily in the ligand–protein active site and ligand-binding pocket (LBP).

Simulations allowed for the evaluation of the various complexes’ thermodynamic and structural stability in the presence of salts and solvents. After preparation of the ligand and protein topology, a cubic box was defined as the unit cell, and the complex was placed at least 1 nm distance from its edges. The system was solvated with TIP3P water model, and Na^+^ or Cl^−^ ions were added to maintain neutrality [40]. Energy minimization was achieved on the system after it was subjected to 50,000 steps and was stopped when a maximum force of 10.0 kJ/mol was achieved. Following this, NVT (amount of substance, volume, and temperature) and NPT (amount of substance, pressure, and temperature) equilibration were run for 50,000 steps each to ensure that the average temperature and pressure of the system were stable. Temperature and pressure were kept constant at 300 K and 1 bar, respectively. Finally, a 100 ns MD simulation was performed to optimize the structure of each complex. Stability of the system was analyzed and ensured using time vs. RMSD (root-mean-square deviation) plots (Supplementary Materials Sections S10–S12). The resulting trajectories were clustered based on the protein active site. This active site was identified as all protein residues within 10 Angstroms of the starting position of each ligand in VMD [38,41,42]. Gromos clustering was used based on the RMSD of the C-alpha atoms within the active site, and the most central member of the most populated cluster was chosen for 2D analysis in LigPlot+ (European Molecular Biological Laboratory) [37]. **FF** was subjected to the same procedure and analysis as the top ligands to validate these methods. The clustered frame of **FF** in 7prv LBD1 retained all hydrogen bonding interactions with Asn564, Gln570, and Arg611, as well as 8 of the 11 hydrophobic interactions described in the existing literature (Supplementary Materials, Section S3) [43]. Finally, pocket volume calculations were performed on the clustered frame of each ligand–protein complex using CASTp default parameters which included a 1.4 Å radius probe [44]. These settings were used for all calculations to produce consistent results.

## 5. Conclusions

In summary, all series **1** compounds that were modified with MBT exhibited a decrease in TA, with **DX1** showing the most profound selectivity, including retention of ~90% of its anti-inflammatory properties, while reducing transcription of GR target genes, such as *Sgk1*, nearly 8-fold. Series **2** compounds also retained anti-inflammatory activity, but TA potential increased upon furoylation. Finally, while series **3** compounds containing an SMe group lost overall potency, the addition of the furoyl moiety in series **4** restored or even increased TR and TA properties at low concentrations for compounds **DX4** and **PN4**.

**DX1, DX4**, and **PN4** have been identified as anti-inflammatory molecules that have a greater selectivity and increased anti-inflammatory activity when compared with dexamethasone and prednisolone. Additionally, molecular dynamics simulations were performed to determine accurate binding positions of ligands in the GR and revealed an expanded GR LBP in response to **DX4** and **PN4**. Further, this expansion caused an unoccupied channel in the region of the GR LBP similar to that created upon **DAC** binding needed to accommodate the bulk of the arylpyrrazole group. We hypothesize that creation of this channel leads to an overall GR conformation that alters the activity of the protein. Forthcoming in vivo studies and further modifications to the various standard of care glucocorticoids will provide a deeper understanding of the mechanism of GC/GR function and improve the understanding of both clinically available anti-inflammatory compounds as well as novel molecules that have improved side effects profiles.

## Figures and Tables

**Table 1:** General Biological Analysis of CCL2 (TR) and 3xGRE (TA) Promoter Activity.

Compd		CCL2			3xGRE	
	pIC_50_	E_max_	^b^ %DX	pEC_50_	E_max_	^b^ %DX
DX	8.3	88.6	100.0	6.8	7.9	100.0
^a^ HC1	6.9	77.0	94.0	5.3	1.8	24.6
^a^ HC2	6.2	72.8	82.2	7.4	8.2	104.8
HC3	NDR	37.1	41.8	NDR	1.4	17.2
HC4	7.1	78.6	88.7	AAC	3.6	45.9
DX1	7.2	80.0	90.3	NDR	1.0	12.6
DX2	8.3	89.7	101.3	6.7	4.9	62.2
DX3	AAC	75.1	84.7	NDR	7.4	94.4
DX4	AAC	84.7	95.6	AAC	8.9	113.0
BM	8.3	88.7	100.1	9.8	10.3	131.3
BM1	7.2	85.7	96.7	7.2	2.2	28.0
BM2	7.8	83.3	94.0	7.8	4.7	59.9
BM3	8.9	83.5	94.3	7.3	4.8	60.6
BM4	7.2	81.6	92.1	7.5	4.9	62.0
PN	8.4	85.1	96.1	9.4	10.0	127.2
PN1	5.9	75.0	84.7	6.1	2.4	30.5
PN2	AAC	90.9	102.6	8.8	7.5	95.9
PN3	7.1	49.6	56.0	6.6	3.4	43.3
PN4	AAC	80.8	91.2	AAC	5.4	69.2
FM	AAC	90.4	102.1	AAC	10.2	129.5
FM1	7.5	85.9	97.0	8.8	4.8	60.4
FM2	AAC	88.3	99.6	7.9	11.7	149.1
FM3	AAC	82.2	92.8	AAC	5.6	71.6
FM4	AAC	75.9	85.7	AAC	4.9	62.6
DN	8.8	83.2	93.9	AAC	9.6	121.7
DN1	7.9	65.6	74.1	7.0	2.5	31.8
BD	AAC	83.4	94.1	AAC	7.0	88.7
BD1	6.6	57.6	65.0	6.0	2.0	25.3

^a^ Selected values taken from previously published work [14]. ^b^ Values represented as percentage of maximal response of dexamethasone. AAC = Active at all concentrations. NDR = No dose-dependent response. Note: All tabulated raw values and standard deviations are available in Supplementary Materials, Sections S4–S9.

**Table 2:** General Biological Analysis of CCL2 (TR) and 3xGRE (TA) Promoter Activity of DX Analogues.

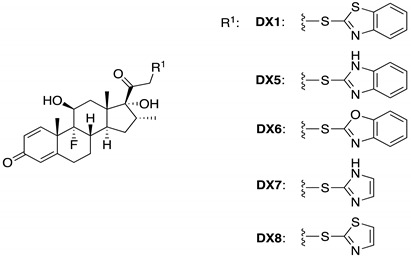						
Compd		CCL2			3xGRE	
	pIC_50_	E_max_	^a^ %DX	pEC_50_	E_max_	^a^ %DX
**DX**	8.3	88.6	100.0	6.8	7.9	100.0
**DX1**	7.2	80.0	90.3	NDR	1.0	12.6
**DX5**	6.2	67.2	75.8	5.8	3.2	40.5
**DX6**	7.1	65.0	73.4	7.5	4.9	61.7
**DX7**	7.1	66.7	75.3	7.7	6.5	82.4
**DX8**	6.3	54.0	60.9	6.9	2.3	29.5

^a^ Values represented as percentage of maximal response of dexamethasone. NDR = No dose-dependent response.

## Data Availability

Data are contained within the article.

## Supplementary Materials

The following supporting information can be downloaded at: https://www.mdpi.com/article/10.3390/molecules29071546/s1, Figure S1: Confirming the reliability of the docking process; Table S1A: CCL2-Luc Assay Raw Data, Table S1B: Low-Dose CCL2-Luc Assay Raw Data, Table S2A: 3xGRE-Luc Assay Raw Data, Table S2B: Low-Dose 3xGRE-Luc Assay Raw Data, Table S3: ADK Assay Raw Data, Table S4: MTS Assay Raw Data, Table S5: *Ccl2* Assay Raw Data, Table S6: *Ccl20* Assay Raw Data, Table S7A: *Sgk1* Assay Raw Data, Table S7B: Low-Dose *Sgk1* Assay Raw Data, Table S8A: *Rgs2* Assay Raw Data, Table S8B: Low-Dose *Rgs2* Assay, Table S9: Relative Binding Affinities of Docked Compounds, Table S10: Pocket Volume Calculations.
