# Supplementary material for: Channel Expansion in the Ligand-Binding Domain of the Glucocorticoid Receptor Contributes to the Activity of Highly Potent Glucocorticoid Analogues
Source: Molecules. 2024 Mar 29;29(7):1546. doi: 10.3390/molecules29071546 (PMC11013598; doi:10.3390/molecules29071546)
Supplement: Supplementary file 1 [file molecules-29-01546-s001.zip › molecules-2942760-supplementary.pdf]

Supporting Information

Channel Expansion in the Ligand-Binding Domain of the Glucocorticoid Receptor Contributes to the Activity of Highly Potent Glucocorticoid Analogues

Wesley B. Seaton 1, Susan J. Burke 2, Alexander R. Fisch 1,†, William A. Schilletter 1, Mary Grace A. Beck 2, Gabrielle A. Cassagne 2, Innocence Harvey 2, Molly S. Fontenot 2, J. Jason Collier 2 and Shawn R. Campagna 1,*

1 Department of Chemistry, University of Tennessee, Knoxville, TN 37996, USA; wseaton2@vols.utk.edu (W.B.S.); fischar@ornl.gov (A.R.F.); wschille@vols.utk.edu (W.A.S.)
2 Pennington Biomedical Research Center, Baton Rouge, LA 70808, USA; susan.burke@pbrc.edu (S.J.B.); marygrace.beck19@gmail.com (M.G.A.B.); gabrielle.cassagne@pbrc.edu (G.A.C.); innocence.harvey@pbrc.edu (I.H.); molly.fontenot@pbrc.edu (M.S.F.); jason.collier@pbrc.edu (J.J.C.)
* Correspondence: campagna@utk.edu
† Current address: Biosciences Division, Oak Ridge National Laboratory, Oak Ridge, TN 37831, USA.

Contents

1. Synthetic Procedures and Characterization……………………………..…..S3 - S12
2. ^{1}H and ^{13}C NMR Spectra…………………………………………..…….S13 - S36
3. LigPlot Analysis Following Molecular Dynamics Simulation in 7prv…......S37 - S39
4. CCL2 Promoter Activity……………………………………….………..S40 - S59
5. 3xGRE Promoter Activity………………………………...…..……..……S60 - S78
6. Adenylate Kinase Release………………………………………..……..S79 - S81
7. MTS Reduction…………………………………………………..……….S82 - S84
8. *Ccl2* and *Ccl20* Gene Expression……………………………………...S85 - S88
9. *Sgk1* and *Rgs2* Gene Expression……………………………………....S89 - S94
10. Protein backbone RMSD plots of 7prv with steroids throughout the MD trajectory of 10 ns………………………………………………………..……….…...S95
11. RMSD plots of ligand heavy atom throughout the MD trajectory of 10 ns……………………………………………………………………...……….....S95
12. RMSF plot of ligand bound 7prv……………………………………...………… .S96
13. Relative Binding Affinities of Compounds Before and After MD Simulation…...S97

1. Synthetic Procedures and Characterization

General Methods: Unless otherwise noted, all reactions were performed in inert atmospheric conditions using dry solvents. All reagents and solvents were obtained from commercial suppliers. ^{1}H NMR and ^{13}C NMR spectra were taken on either a Varian Inova 500 MHz instrument or Bruker 5 mm SmartProbe 500MHz in $CDCl_3$ solutions. Thin-layer chromatography (TLC) was performed with Sorbent Technologies silica G w/UV254 TLC plates. High resolution mass spectrum were performed on an Exactive Plus Orbitrap mass spectrometer with an ESI source operating in positive ionization mode. Specific rotations were taken on a Perkin Elmer 241 Polarimeter and IR were performed on a Thermo Nicolet IR-1000 FTIR.

General procedure for mercapto derivitization – Steroid (1.34 mmol) was stirred in 25ml of dichloromethane and cooled to 0 °C. Diisopropylethylamine (DIPEA) (1.3 mL, 10.2 mmol) and mesylchloride (MsCl) (0.16 mL, 2.01 mmol) were then added and the reaction was slowly warmed to room temperature. The reaction was allowed to stir for 15 h until no changed was observed in TLC and then concentrated *in vacuo*. The resulting oil was redissolved in 25 mL DCM and washed with 50 mL saturated bicarbonate. The aqueous layer was extracted with 10 mL DCM. The combined organic layers were washed with brine (25 mL x 2), dried with $MgSO_4$, filtered, and concentrated *in vacuo*. The tan mesylate was carried through crude to the next reaction.

To a solution of steroid mesylate (0.25 g, 0.567 mmol) in acetone (50 mL) was added potassium carbonate (0.784 g, 5.67 mmol) and appropriate mercapto reagent (1.14 mmol) and subjected to reflux for 1 h. The mixture was filtered through a short pad of silica and concentrated *in vacuo*. Purification of the crude product was accomplished using flash chromatography (40% EtOAc in hexanes) to give a white or tan product.

(8S,9R,10S,11S,13S,14S,16R,17R)-17-(2-(benzo[d]thiazol-2-ylthio)acetyl)-9-fluoro-11,17-dihydroxy-10,13,16-trimethyl-6,7,8,9,10,11,12,13,14,15,16,17-dodecahydro-3H-cyclopenta[a]phenanthren-3-one (DX1): white solid obtained in 41% yield; mp 210.7-212 °C; TLC (Silica G w/UV254) R_f = 0.59, 10:1 CH_2Cl_2:MeOH; $[\alpha]_D^{20}$ = +219.66 (c 0.61, CH_2Cl_2); ^{1}H NMR (500 MHz, $CDCl_3$) δ 7.85 (d, *J* = 8.2 Hz, 1H), 7.73 (d, *J* = 7.9 Hz, 1H), 7.43 (t, *J* = 8.4 Hz, 1H), 7.33 (t, *J* = 8.2 Hz, 1H), 7.21 (s, 1H), 6.34 (d, *J* = 12.1 Hz, 1H), 6.13 (s, 1H), 5.12 (d, *J* = 15.4 Hz, 1H), 4.42 (d, *J* = 7.8 Hz, 1H), 3.31 (d, *J* = 15.4 Hz, 1H), 3.09 – 3.18 (m, 1H), 2.75 (d, *J* = 14.2 Hz, 1H), 2.57 – 2.67 (m, 1H), 2.30 – 2.48 (m, 3H), 2.17 (s, 1H), 1.80 – 1.91 (m, 1H), 1.61 – 1.76 (m, 2H), 1.56 (s, 4H), 1.30 (td, *J* = 8.0, 4.0 Hz, 1H), 1.25 (s, 1H), 1.04 (s, 3H), 0.92 (d, *J* = 7.2 Hz, 3H).;^{13}C NMR (126 MHz, $CDCl_3$) δ 206.02, 186.58, 168.28, 166.14, 152.00, 150.48, 134.31, 129.92, 126.78, 125.23, 121.34, 120.53, 100.78, 99.38, 92.10, 72.48, 48.38, 47.45, 43.65, 37.66, 35.00, 34.40, 32.54, 31.11, 29.29, 27.31, 23.02, 17.27, 15.57; IR (NaCl, thin film) ν_{max} (cm^{-1}): 3344, 2940, 2871, 1658, 1426, 753; HRMS (ESI) m/z: $[M+H]^+$ Calcd for $C_{29}H_{32}FNO_4S_2$, 542.1831; found 542.1833.

(8S,9R,10S,11S,13S,14S,16R,17R)-17-(2-((1H-benzo[d]imidazol-2-yl)thio)acetyl)-9-fluoro-11,17-dihydroxy-10,13,16-trimethyl-6,7,8,9,10,11,12,13,14,15,16,17-dodecahydro-3H-cyclopenta[a]phenanthren-3-one (DX5): white solid obtained in 36.5% yield; mp 181.1-182.1

°C; TLC (Silica G w/UV254) R_f = 0.37, 10:1 CH_2Cl_2:MeOH; $[\alpha]_D^{20}$ = +94.2 (c 0.54, MeOH); ^{1}H NMR (500 MHz, DMSO) δ 7.41 (s, 1H), 7.33 (d, *J* = 10.2 Hz, 1H), 7.12 (dd, *J* = 6.0, 3.2 Hz, 2H), 6.68 (s, 1H), 6.23 (dd, *J* = 10.1, 2.0 Hz, 1H), 6.02 (s, 1H), 5.40 (d, *J* = 4.9 Hz, 1H), 4.76 (d, *J* = 16.2 Hz, 1H), 4.20 (s, 1H), 3.84 (d, *J* = 16.0 Hz, 1H), 2.91 – 2.99 (m, 1H), 2.63 (td, *J* = 14.0, 6.1 Hz, 1H), 2.31 – 2.44 (m, 3H), 2.18 – 2.26 (m, 1H), 2.08 (s, 1H), 1.76 – 1.84 (m, 1H), 1.64 (t, *J* = 11.8 Hz, 2H), 1.50 (s, 3H), 1.33 – 1.43 (m, 1H), 1.07 – 1.18 (m, 2H), 0.91 (s, 3H), 0.79 (d, *J* = 7.2 Hz, 3H). ^{13}C NMR (126 MHz, DMSO) δ 207.37, 185.78, 167.59, 153.30, 150.92, 129.45, 124.60, 102.44, 101.05, 91.78, 71.16, 70.87, 48.53, 48.35, 47.72, 43.76, 40.59, 40.50, 40.42, 40.33, 40.26, 40.17, 40.09, 40.00, 39.92, 39.83, 39.67, 39.50, 37.22, 36.44, 34.84, 34.13, 33.98, 32.54, 31.16, 30.78, 27.76, 23.48, 23.43, 16.96, 15.87. IR (NaCl, thin film) ν_{max} (cm^{-1}): 3362, 2926, 1655, 1307, 759; HRMS (ESI) m/z: [M+H]$^+$ Calcd for $C_{29}H_{33}FN_2O_4S$, 525.2223; found 525.2213.

(8*S*,9*R*,10*S*,11*S*,13*S*,14*S*,16*R*,17*R*)-17-(2-(benzo[*d*]oxazol-2-ylthio)acetyl)-9-fluoro-11,17-dihydroxy-10,13,16-trimethyl-6,7,8,9,10,11,12,13,14,15,16,17-dodecahydro-3*H*-cyclopenta[*a*]phenanthren-3-one (DX6): white solid obtained in 31% yield; mp 180.9-182.9 °C; TLC (Silica G w/UV254) R_f = 0.46, 10:1 CH_2Cl_2:MeOH; $[\alpha]_D^{20}$ = +75.3 (c 0.56, CH_2Cl_2); ^{1}H NMR (500 MHz, $CDCl_3$) δ 7.48 (d, *J* = 9.3 Hz, 1H), 7.44 (d, *J* = 7.3 Hz, 1H), 7.27 – 7.30 (m, 2H), 7.20 (d, *J* = 10.2 Hz, 1H), 6.35 (d, *J* = 10.1 Hz, 1H), 6.14 (s, 1H), 5.03 (d, *J* = 15.3 Hz, 1H), 4.43 (d, *J* = 7.6 Hz, 1H), 3.15 (ddd, *J* = 11.0, 7.2, 3.9 Hz, 1H), 3.08 (d, *J* = 15.3 Hz, 1H), 2.78 (d, *J* = 14.0 Hz, 1H), 2.58 – 2.66 (m, 1H), 2.40 – 2.45 (m, 2H), 2.37 (dd, *J* = 16.1, 4.0 Hz, 1H), 1.83 – 1.89 (m, 2H), 1.73 (d, *J* = 12.1 Hz, 1H), 1.63 (dd, *J* = 12.3, 5.4 Hz, 1H), 1.56 (s, 3H), 1.46 (d, *J* = 14.3 Hz, 1H), 1.33 (d, *J* = 4.0 Hz, 2H), 1.04 (s, 3H), 0.93 (d, *J* = 7.1 Hz, 3H). ^{13}C NMR (126 MHz, $CDCl_3$) δ 206.49, 186.49, 165.87, 165.26, 152.09, 151.69, 140.21, 130.02, 125.23, 124.89, 124.61, 117.77, 110.31, 91.84, 77.26, 77.01, 76.76, 72.50, 72.19, 48.12, 47.39, 43.62, 37.95, 35.02, 34.37, 34.21, 32.55, 31.08, 27.28, 23.04, 17.30, 15.25. IR (NaCl, thin film) ν_{max} (cm^{-1}): 3361, 2927, 1653, 1308, 758; HRMS (ESI) m/z: [M+H]$^+$ Calcd for $C_{29}H_{32}FNO_5S$, 526.2063; found 526.2047.

(8*S*,9*R*,10*S*,11*S*,13*S*,14*S*,16*R*,17*R*)-17-(2-((1*H*-imidazol-2-yl)thio)acetyl)-9-fluoro-11,17-dihydroxy-10,13,16-trimethyl-6,7,8,9,10,11,12,13,14,15,16,17-dodecahydro-3*H*-cyclopenta[*a*]phenanthren-3-one (DX7): white solid obtained in 16.3% yield; mp 240.5-249.0 °C; TLC (Silica G w/UV254) R_f = 0.34, 10:1 CH_2Cl_2:MeOH; $[\alpha]_D^{20}$ = +82.3 (c 0.55, MeOH); ^{1}H NMR (500 MHz, cd_3od) δ 7.30 – 7.44 (m, 1H), 7.00 (s, 2H), 6.28 (dd, *J* = 10.1, 1.9 Hz, 1H), 6.08 (s, 1H), 4.49 (d, *J* = 16.2 Hz, 1H), 4.25 (d, *J* = 8.9 Hz, 1H), 3.63 (d, *J* = 16.2 Hz, 1H), 3.01 – 3.10 (m, 1H), 2.71 (td, *J* = 14.0, 5.7 Hz, 1H), 2.47 (td, *J* = 12.1, 5.1 Hz, 1H), 2.39 (dt, *J* = 11.9, 3.3 Hz, 3H), 2.25 (td, *J* = 11.9, 8.4 Hz, 1H), 2.15 (s, 1H), 1.88 (dt, *J* = 12.9, 6.0 Hz, 1H), 1.71 (q, *J* = 11.8 Hz, 1H), 1.58 (s, 3H), 1.44 – 1.57 (m, 2H), 1.29 (s, 1H), 1.19 (ddd, *J* = 12.2, 8.1, 4.0 Hz, 1H), 0.95 (s, 3H), 0.82 (d, *J* = 7.2 Hz, 3H). ^{13}C NMR (126 MHz, MeOD) δ 187.64, 169.61, 154.51, 128.40, 123.73, 91.60, 71.66, 71.36, 48.93, 48.74, 48.22, 48.10, 48.05, 47.93, 47.88, 47.76, 47.59, 47.42, 47.25, 47.08, 43.52, 36.29, 34.88, 34.23, 34.07, 32.09, 30.82, 27.40, 22.26, 22.21, 16.04, 14.07. ν_{max} (cm^{-1}): 3361, 2928, 1655, 1306, 757; HRMS (ESI) m/z: [M+H]$^+$ Calcd for $C_{25}H_{31}FN_2O_4S$, 475.2067; found 475.2078.

(8*S*,9*R*,10*S*,11*S*,13*S*,14*S*,16*R*,17*R*)-9-fluoro-11,17-dihydroxy-10,13,16-trimethyl-17-(2-(thiazol-2-ylthio)acetyl)-6,7,8,9,10,11,12,13,14,15,16,17-dodecahydro-3*H*-cyclopenta[*a*]phenanthren-3-one (DX8): white solid obtained in 38.2% yield; ; mp 191.1-191.8 °C; TLC (Silica G w/UV254) R_f = 0.29, 10:1 CH_2Cl_2:MeOH; $[\alpha]_D^{20}$ = +10.0 (c 0.55, CH_2Cl_2); ^{1}H NMR (500 MHz, $CDCl_3$) δ 7.57 (d, *J* = 3.7 Hz, 1H), 7.27 (s, 1H), 7.21 (d, *J* = 10.1 Hz, 1H), 6.34 (d, *J* = 12.1 Hz, 1H), 6.12 (s, 1H), 5.03 (d, *J* = 15.4 Hz, 1H), 4.41 (d, *J* = 8.2 Hz, 1H), 3.45 (d, *J* = 15.4 Hz, 1H), 3.07 – 3.19 (m, 1H), 2.72 – 2.57 (m, 2H), 2.32 – 2.40 (m, 3H), 1.84 (d, *J* = 6.7 Hz, 1H), 1.70 (d, *J* = 12.1 Hz, 1H), 1.60 (d, *J* = 14.5 Hz, 2H), 1.55 (s, 3H), 1.26 (s, 3H), 1.01 (s, 3H), 0.89 (d, *J* = 7.2 Hz, 3H). ^{13}C NMR (126 MHz, $CDCl_3$) δ 186.51, 165.88, 151.85, 129.95, 125.21, 119.69, 91.79, 77.26, 77.01, 76.76, 72.51, 72.20, 48.32, 48.14, 47.43, 43.72, 37.47, 35.17, 34.41, 34.26, 32.51, 31.06, 29.71, 27.26, 23.03, 22.98, 17.17, 15.06. ν_{max} (cm^{-1}): 3360, 2928, 1655, 1097, 758; HRMS (ESI) m/z: $[M+H]^+$ Calcd for $C_{25}H_{30}FNO_4S_2$, 492.1679; found 492.1665.

(8S,9R,10S,11S,13S,14S,16S,17R)-17-(2-(benzo[d]thiazol-2-ylthio)acetyl)-9-fluoro-11,17-dihydroxy-10,13,16-trimethyl-6,7,8,9,10,11,12,13,14,15,16,17-dodecahydro-3H-cyclopenta[a]phenanthren-3-one (BM1): white solid obtained in 49% yield; mp 202.4-207.8 °C; TLC (Silica G w/UV254) R_f = 0.52, 10:1 CH_2Cl_2:MeOH; $[\alpha]_D^{20}$ = +80.1 (c 0.8, CH_2Cl_2); ^{1}H NMR (500 MHz, $CDCl_3$) δ 7.73 (dd, *J* = 8.2, 3.9 Hz, 2H), 7.42 (t, *J* = 7.8 Hz, 1H), 7.31 (t, *J* = 7.6 Hz, 1H), 7.20 (d, *J* = 10.1 Hz, 1H), 6.34 (dd, *J* = 10.1, 2.0 Hz, 1H), 6.14 (s, 1H), 4.35 (d, *J* = 9.2 Hz, 1H), 4.21 (d, *J* = 16.4 Hz, 1H), 4.15 (d, *J* = 15.0 Hz, 1H), 2.64 (td, *J* = 13.8, 6.1 Hz, 1H), 2.44 – 2.51 (m, 2H), 2.38 – 2.43 (m, 1H), 2.34 (q, *J* = 8.3 Hz, 1H), 2.26 (td, *J* = 12.3, 6.3 Hz, 1H), 2.17 (s, 1H), 2.09 – 2.16 (m, 1H), 1.89 – 2.94 (m, 1H), 1.67 (td, *J* = 13.3, 5.3 Hz, 1H), 1.56 (s, 3H), 1.40 (d, *J* = 15.4 Hz, 1H), 1.24 – 1.35(m, 1H), 1.20 (s, 3H), 1.15 (d, *J* = 8.4 Hz, 1H), 1.09 (d, *J* = 7.6 Hz, 3H); ^{13}C NMR (126 MHz, $CDCl_3$) δ 186.51, 165.97, 151.96, 129.90, 127.65, 126.08, 125.20, 121.50, 121.12, 119.59, 100.80, 99.39, 90.09, 72.59, 60.40, 48.35, 47.97, 43.37, 37.88, 35.13, 33.95, 33.79, 31.11, 27.54, 23.03, 22.99, 20.00, 17.50, 14.21; IR (NaCl, thin film) ν_{max} (cm^{-1}): 3383, 2933, 2357, 1659, 1428, 889; HRMS (ESI) m/z: $[M+H]^+$ Calcd for $C_{29}H_{32}FNO_4S_2$, 542.1835; found 542.1837.

(8S,9S,10R,11S,13S,14S,17R)-17-(2-(benzo[d]thiazol-2-ylthio)acetyl)-11,17-dihydroxy-10,13-dimethyl-6,7,8,9,10,11,12,13,14,15,16,17-dodecahydro-3H-cyclopenta[a]phenanthren-3-one (PN1): white solid obtained in 38% yield; mp 174.8 – 189.6 °C; TLC (Silica G w/UV254) R_f = 0.44, 10:1 CH_2Cl_2:MeOH; $[\alpha]_D^{20}$ = +138.84 (c 0.77, CH_2Cl_2); ^{1}H NMR (500 MHz, $CDCl_3$) δ 7.67 – 7.78 (m, 2H), 7.39 (t, *J* = 7.1 Hz, 1H), 7.30 (d, *J* = 7.1 Hz, 1H), 7.27 (s, 1H), 6.27 (d, *J* = 10.0 Hz, 1H), 6.03 (s, 1H), 5.81 (s, 1H), 4.95 (d, *J* = 15.3 Hz, 1H), 4.52 (s, 1H), 3.32 (d, *J* = 15.3 Hz, 1H), 2.81 (t, *J* = 11.6 Hz, 1H), 2.57 (td, *J* = 13.6, 4.6 Hz, 1H), 2.31 – 2.39 (m, 2H), 2.16 (s, 2H), 1.80 – 1.96 (m, 2H), 1.53 – 1.67 (m, 3H), 1.46 (s, 3H), 1.41 – 1.45 (m, 1H), 1.13 – 1.21 (m, 2H), 0.98 (s, 3H); ^{13}C NMR (126 MHz, $CDCl_3$) δ 205.26, 186.66, 170.09, 156.13, 128.10, 127.51, 126.05, 122.67, 122.63, 121.59, 119.96, 90.92, 70.60, 60.54, 55.42, 51.66, 47.31, 44.19, 40.70, 39.85, 35.35, 34.08, 32.21, 31.56, 24.27, 21.29, 17.80, 14.35; IR (NaCl, thin film) ν_{max} (cm^{-1}): 3345, 2936, 2362, 1622, 648; HRMS (ESI) m/z: $[M+H]^+$ Calcd for $C_{28}H_{31}NO_4S_2$, 510.1767; found 510.1774.

(6S,8S,9R,10S,11S,13S,14S,16R,17R)-17-(2-(benzo[d]thiazol-2-ylthio)acetyl)-6,9-difluoro-11,17-dihydroxy-10,13,16-trimethyl-6,7,8,9,10,11,12,13,14,15,16,17-dodecahydro-3H-cyclopenta[a]phenanthren-3-one (FM1): tan solid obtained in 41% yield; mp 210.2-211.8 °C; TLC (Silica G w/UV254) R_f = 0.59, 10:1 CH_2Cl_2:MeOH; $[\alpha]_D^{20}$ = +138.76 (c 0.51, CH_2Cl_2); ^{1}H NMR (500 MHz, $CDCl_3$) δ 7.79 (d, *J* = 8.2 Hz, 1H), 7.72 (d, *J* = 8.1 Hz, 1H), 7.41 (t, *J* = 7.8 Hz, 1H), 7.29 – 7.35 (m, 1H), 7.13 (d, *J* = 10.4 Hz, 1H), 6.45 (s, 1H), 6.38 (d, *J* = 10.1 Hz, 1H), 5.25 – 5.49 (m, 1H), 5.06 (d, *J* = 15.0 Hz, 1H), 4.44 (d, *J* = 7.7 Hz, 1H), 3.17 (ddd, *J* = 11.0, 7.1, 3.7 Hz, 1H), 3.08 (d, *J* = 15.2 Hz, 1H), 2.81 (d, *J* = 14.5 Hz, 1H), 2.52 – 2.61 (m, 1H), 2.35 – 2.49 (m, 1H), 2.29 (t, *J* = 5.9 Hz, 1H), 1.69 – 1.88 (m, 3H), 1.54 (s, 3H), 1.49 (d, *J* = 13.0 Hz, 1H), 1.33 (s, 1H), 1.25 (s, 1H), 1.03 (s, 3H), 0.91 (d, *J* = 7.1 Hz, 3H); ^{13}C NMR (126 MHz, $CDCl_3$) δ 206.48, 185.61, 167.30, 151.40, 150.51, 134.84, 130.27, 126.50, 124.92, 121.29, 121.08, 120.74, 92.02, 87.40, 87.37, 85.90, 72.24, 71.94, 47.36, 43.29, 37.68, 36.24, 34.88, 33.62, 32.89, 32.35, 23.08, 17.27, 15.56; IR (NaCl, thin film) ν_{max} (cm^{-1}): 3343, 2913, 2363, 1617, 754; HRMS (ESI) m/z: $[M+H]^+$ Calcd for $C_{29}H_{31}F_2NO_4S_2$, 560.1735; found 560.1741.

(6aR,6bS,7S,8aS,8bS,11aR,12aS,12bS)-8b-(2-(benzo[d]thiazol-2-ylthio)acetyl)-7-hydroxy-6a,8a,10,10-tetramethyl-1,2,6a,6b,7,8,8a,8b,11a,12,12a,12b-dodecahydro-4H-naphtho[2',1':4,5]indeno[1,2-d][1,3]dioxol-4-one (DN1): white solid obtained in 32% yield; mp 218.3-230 °C; TLC (Silica G w/UV254) R_f = 0.54, 10:1 CH_2Cl_2:MeOH; $[\alpha]_D^{20}$ = +118.56 (c 0.84, CH_2Cl_2); ^{1}H NMR (500 MHz, $CDCl_3$) δ 7.86 (d, *J* = 7.2 Hz, 1H), 7.77 (d, *J* = 7.0 Hz, 1H), 7.43 (t, *J* = 7.1 Hz, 1H), 7.34 (t, *J* = 7.0 Hz, 1H), 7.28 (d, *J* = 10.0 Hz, 1H), 6.28 (d, *J* = 10.1 Hz, 1H), 6.03 (s, 1H), 5.04 (d, *J* = 4.6 Hz, 1H), 4.70 (d, *J* = 18.1 Hz, 1H), 4.62 (d, *J* = 18.1 Hz, 1H), 4.55 (s, 1H), 2.51 – 2.59 (m, 1H), 2.33 (d, *J* = 11.2 Hz, 1H), 2.16 (s, 2H), 2.13 (d, *J* = 3.4 Hz, 1H), 2.03 – 2.09 (m, 2H), 1.73 (d, *J* = 7.3 Hz, 1H), 1.56 – 1.63 (m, 2H), 1.46 (s, 6H), 1.22 (s, 3H), 1.14 (t, *J* = 11.7 Hz, 2H), 0.98 (s, 3H); ^{13}C NMR (126 MHz, $CDCl_3$) δ 202.88, 186.59, 156.35, 127.99, 127.88, 126.54, 122.56, 121.55, 119.90, 111.33, 102.24, 98.59, 81.90, 81.18, 70.20, 60.39, 55.33, 50.04, 45.73, 44.17, 39.75, 34.07, 33.97, 31.96, 30.75, 26.59, 25.61, 21.01, 16.80, 14.21; IR (NaCl, thin film) ν_{max} (cm^{-1}): 3383, 2931, 3258, 1654, 755; HRMS (ESI) m/z: $[M+H]^+$ Calcd for $C_{31}H_{35}NO_5S_2$, 566.2029; found 566.2036.

(6aR,6bS,7S,8aS,8bS,12aS,12bS)-8b-(2-(benzo[d]thiazol-2-ylthio)acetyl)-7-hydroxy-6a,8a-dimethyl-10-propyl-1,2,6a,6b,7,8,8a,8b,11a,12,12a,12b-dodecahydro-4H-naphtho[2',1':4,5]indeno[1,2-d][1,3]dioxol-4-one (BD1): white solid obtained in 22% yield; mp 213.5-214.5 °C; TLC (Silica G w/UV254) R_f = 0.54, 10:1 CH_2Cl_2:MeOH; $[\alpha]_D^{20}$ = +120.16 (c 0.54, CH_2Cl_2); ^{1}H NMR (500 MHz, $CDCl_3$) δ 7.78 (dd, *J* = 22.3, 8.0 Hz, 2H), 7.40 (t, *J* = 7.7 Hz, 1H), 7.30 (t, *J* = 7.6 Hz, 1H), 6.29 (d, *J* = 4.0 Hz, 1H), 6.03 (s, 1H), 5.23 (s, 1H), 4.91 (s, 1H), 4.76 (s, 1H), 4.55 – 4.59 (m, 1H), 4.52 (s, 1H), 4.43 (s, 1H), 2.53 – 2.61 (m, 1H), 2.35 (d, *J* = 10.1 Hz, 1H), 2.04 – 2.24 (m, 4H), 1.73 – 1.86 (m, 2H), 1.68 (q, *J* = 4.0 Hz, 2H), 1.55 – 1.63 (m, 2H), 1.47 (s, 4H), 1.37 – 1.44 (m, 2H), 1.17 (t, *J* = 11.9 Hz, 2H), 1.05 (s, 1H), 1.00 (s, 1H), 0.94 (d, *J* = 11.7 Hz, 3H); ^{13}C NMR (126 MHz, $CDCl_3$) δ 202.58, 201.10, 186.38, 169.38, 155.67, 152.73, 135.63, 128.10, 126.14, 124.53, 122.66, 121.24, 108.52, 104.59, 99.26, 83.31, 70.18, 55.27, 52.89, 49.88, 47.17, 45.89, 43.95, 41.66, 37.23, 35.14, 34.01, 31.88, 31.03, 21.16, 17.55, 14.06. ; IR (NaCl, thin film) ν_{max} (cm^{-1}): 3352, 2931, 1655, 756; HRMS (ESI) m/z: $[M+H]^+$ Calcd for $C_{32}H_{37}NO_5S_2$, 580.2186; found 580.2192.

General procedure for compound 2 and 4 derivitization: Steroid 1 (0.115 g, 0.212 mmol) in 5 mL CH_2Cl_2 was cooled to 0°C. DMAP (0.065g, 0.531 mmol) and 2-furoyl chloride (0.031g, 0.024 mL, 0.255 mmol) were added and the reaction was warmed to room temperature and allowed to stir for 28 h. The reaction was diluted with 5 mL CH_2Cl_2 and washed with brine (15 mL x 3), dried with $MgSO_4$, filtered, and concentrated *in vacuo*. Purification of the crude product was accomplished using flash chromatography (1% MeOH in CH_2Cl_2) to give a white product.

(8S,9R,10S,11S,13S,14S,16R,17R)-17-(2-(benzo[d]thiazol-2-ylthio)acetyl)-9-fluoro-11-hydroxy-10,13,16-trimethyl-3-oxo-6,7,8,9,10,11,12,13,14,15,16,17-dodecahydro-3H-cyclopenta[a]phenanthren-17-yl furan-2-carboxylate (DX2): white solid obtained in 70% yield; mp 217.4-224.7°C; TLC (Silica G w/UV254) R_f = 0.60, 10:1 CH_2Cl_2:MeOH; $[\alpha]_D^{20}$ = +82.78 (c 0.64, CH_2Cl_2); ^{1}H NMR (500 MHz, $CDCl_3$) δ 7.74 – 7.80 (m, 2H), 7.60 (d, *J* = 1.0 Hz, 1H), 7.41 (t, *J* = 7.6 Hz, 1H), 7.31 (t, *J* = 7.6 Hz, 1H), 7.17 – 7.22 (m, 2H), 6.52 (d, *J* = 5.3 Hz, 1H), 6.37 (d, *J* = 10.0 Hz, 1H), 6.16 (s, 1H), 4.44 – 4.60 (m, 3H), 3.52 (s, 1H), 2.63 (t, *J* = 8.3 Hz, 2H), 2.42 (d, *J* = 21.4 Hz, 3H), 1.89 (d, *J* = 12.2 Hz, 3H), 1.66 (dt, *J* = 12.1, 6.3 Hz, 1H), 1.57 (s, 3H), 1.32 – 1.40 (m, 1H), 1.26 (s, 1H), 1.18 (s, 3H), 1.02 (d, *J* = 7.2 Hz, 3H); ^{13}C NMR (126 MHz, $CDCl_3$) δ 186.39, 165.95, 165.67, 158.05, 152.71, 151.68, 147.46, 143.20, 135.61, 130.01, 126.03, 125.25, 124.39, 121.25, 119.47, 112.17, 100.77, 99.37, 72.11, 71.80, 48.62, 47.99, 43.72, 37.27, 36.04, 34.07, 33.91, 33.63, 32.48, 30.91, 27.32, 22.98, 16.84, 16.70; IR (NaCl, thin film) ν_{max} (cm^{-1}): 3419, 2937, 2358, 1662, 757; HRMS (ESI) m/z: $[M+H]^+$ Calcd for $C_{34}H_{34}FNO_6S_2$, 636.1890; found 636.1889.

(8S,9R,10S,11S,13S,14S,16S,17R)-17-(2-(benzo[d]thiazol-2-ylthio)acetyl)-9-fluoro-11-hydroxy-10,13,16-trimethyl-3-oxo-6,7,8,9,10,11,12,13,14,15,16,17-dodecahydro-3H-cyclopenta[a]phenanthren-17-yl furan-2-carboxylate (BM2): white solid obtained in 56% yield; mp 155.4-161.5 °C; TLC (Silica G w/UV254) R_f = 0.58, 10:1 CH_2Cl_2:MeOH; $[\alpha]_D^{20}$ = +159.0 (c 0.62, CH_2Cl_2); ^{1}H NMR (500 MHz, $CDCl_3$) δ 7.76 (d, *J* = 7.9 Hz, 2H), 7.62 – 7.67 (m, 1H), 7.38 – 7.43 (m, 1H), 7.28 – 7.33 (m, 1H), 7.21 – 7.25 (m, 2H), 6.55 (dd, *J* = 3.5, 1.7 Hz, 1H), 6.39 (d, *J* = 10.1 Hz, 1H), 6.16 (s, 1H), 4.59 (d, *J* = 17.1 Hz, 1H), 4.55 (s, 1H), 4.12 (d, *J* = 17.2 Hz, 1H), 2.77 (d, *J* = 14.3 Hz, 1H), 2.65 (td, *J* = 13.1, 6.1 Hz, 1H), 2.45 – 2.55 (m, 1H), 2.41 (dd, *J* = 13.9, 3.4 Hz, 1H), 2.32 (dt, *J* = 15.3, 7.8 Hz, 1H), 2.18 (dt, *J* = 11.6, 6.0 Hz, 1H), 2.11 (d, *J* = 14.3 Hz, 1H), 1.90 – 2.00 (m, 2H), 1.63 (dd, *J* = 13.0, 4.8 Hz, 1H), 1.58 (s, 3H), 1.41 (d, *J* = 7.5 Hz, 3H), 1.25 (s, 2H), 1.04 (s, 3H; . ^{13}C NMR (126 MHz, $CDCl_3$) δ 186.59, 152.14, 129.84, 126.70, 126.48, 124.99, 122.36, 121.34, 120.42, 91.09, 90.30, 72.24, 68.57, 55.99, 52.09, 48.26, 47.57, 46.78, 43.38, 40.59, 39.19, 38.39, 37.68, 35.13, 33.69, 32.65, 31.50, 30.92, 27.53, 23.85, 23.01, 21.10, 19.98, 17.53; IR (NaCl, thin film) ν_{max} (cm^{-1}): 3382, 2933, 2358, 1717, 757; HRMS (ESI) m/z: $[M+H]^+$ Calcd for $C_{34}H_{34}FNO_6S_2$, 636.1890; found 636.1890.

(8S,9S,10R,11S,13S,14S,17R)-17-(2-(benzo[d]thiazol-2-ylthio)acetyl)-11-hydroxy-10,13-dimethyl-3-oxo-6,7,8,9,10,11,12,13,14,15,16,17-dodecahydro-3H-cyclopenta[a]phenanthren-17-yl furan-2-carboxylate (PN2): white solid obtained in 82% yield; mp 149.3 - 156.9 °C; TLC (Silica G w/UV254) R_f = 0.56, 10:1 CH_2Cl_2:MeOH; $[\alpha]_D^{20}$ = +115.44 (c 0.56, CH_2Cl_2); ^{1}H NMR (500 MHz, $CDCl_3$) δ 7.75 (dd, *J* = 14.9, 8.1 Hz, 2H), 7.60 (s, 1H), 7.38 (t, *J* = 7.7 Hz, 1H), 7.29 (d, *J* = 13.8 Hz, 2H), 7.22 (d, *J* = 3.5 Hz, 1H), 6.53 (d, *J* = 5.4 Hz, 1H), 6.31 (d, *J* = 10.1 Hz, 1H), 6.04 (s, 1H), 4.57 (d, *J* = 17.1 Hz, 2H), 4.37 (d, *J* = 17.1 Hz, 1H), 2.99 – 3.11 (m, 1H), 2.54 – 2.64 (m, 1H), 2.35 (d, *J* = 13.7 Hz, 2H), 2.18 – 2.25 (m, 1H), 2.09 – 2.18 (m, 1H), 2.06 (s, 1H), 1.95 – 2.02 (m, 1H), 1.79 (td, *J* = 17.9, 8.3 Hz, 2H), 1.67 (s, 1H), 1.50

– 1.56 (m, 1H), 1.48 (s, 3H), 1.13 – 1.20 (m, 2H), 1.10 (s, 3H); ^{13}C NMR (126 MHz, $CDCl_3$) δ 197.51, 186.67, 169.93, 158.28, 156.54, 147.52, 146.19, 143.65, 128.15, 127.88, 126.75, 122.57, 121.58, 119.93, 119.55, 112.33, 96.66, 70.08, 55.41, 55.30, 52.75, 47.33, 44.22, 43.27, 39.20, 34.00, 32.01, 31.92, 31.77, 31.24, 24.34, 20.92, 16.26; IR (NaCl, thin film) ν_{max} (cm^{-1}): 3565, 2935, 2308, 1655, 756; HRMS (ESI) m/z: $[M+H]^+$ Calcd for $C_{33}H_{33}NO_6S_2$, 604.1822; found 604.1823.

(6S,8S,9R,10S,11S,13S,14S,16R,17R)-17-(2-(benzo[d]thiazol-2-ylthio)acetyl)-6,9-difluoro-11-hydroxy-10,13,16-trimethyl-3-oxo-6,7,8,9,10,11,12,13,14,15,16,17-dodecahydro-3H-cyclopenta[a]phenanthren-17-yl furan-2-carboxylate (FM2): white solid obtained in 60.7% yield; mp 231.4-237.0°C; TLC (Silica G w/UV254) R_f = 0.56, 10:1 CH_2Cl_2:MeOH; $[\alpha]_D^{20}$ = +176.9 (c 0.56, CH_2Cl_2); ^{1}H NMR (500 MHz, cdcl$_3$) δ 7.79 (dd, *J* = 19.7, 8.4 Hz, 2H), 7.61 (d, *J* = 0.9 Hz, 1H), 7.42 (t, *J* = 7.0 Hz, 1H), 7.30 – 7.35 (m, 1H), 7.20 (d, *J* = 3.5 Hz, 1H), 7.12 (d, *J* = 10.3 Hz, 1H), 6.53 (dd, *J* = 3.5, 1.7 Hz, 1H), 6.47 (s, 1H), 6.40 (dd, *J* = 10.1, 1.9 Hz, 1H), 5.41 (ddd, *J* = 48.5, 10.7, 5.7 Hz, 1H), 4.61 (d, *J* = 16.4 Hz, 1H), 4.48 (s, 1H), 3.54 (s, 1H), 2.62 (d, *J* = 16.4 Hz, 1H), 2.47 (d, *J* = 10.5 Hz, 2H), 2.30 – 2.34 (m, 1H), 1.94 (dd, *J* = 22.7, 12.6 Hz, 2H), 1.80 – 1.87 (m, 1H), 1.55 (s, 3H), 1.39 (s, 1H), 1.33 (s, 1H), 1.26 (s, 1H), 1.17 (d, *J* = 11.2 Hz, 3H), 1.03 (d, *J* = 7.1 Hz, 2H), 0.87 – 0.93 (m, 1H); ^{13}C NMR (126 MHz, DMSO) δ 184.90, 166.28, 163.37, 158.03, 152.77, 152.29, 149.11, 143.14, 135.33, 129.65, 126.86, 125.05, 122.35, 121.56, 120.26, 113.18, 101.12, 99.72, 88.00, 86.57, 70.70, 70.42, 48.35, 43.68, 36.08, 35.75, 34.18, 33.31, 32.44, 23.15, 16.97, 16.34.IR (NaCl, thin film) ν_{max} (cm^{-1}): 3382, 2932, 2358, 1716, 757; HRMS (ESI) m/z: $[M+H]^+$ Calcd for $C_{34}H_{33}F_2NO_6S_2$, 654.1790; found 654.1792

General procedure for S-thiobenzoate intermediate: PPh_3 (0.668 g, 2.55 mmol) and diisopropyl-azodicarboxylate (DIAD) (0.5 mL, 2.55 mmol) were stirred in THF (10 mL) at 0°C for 30 min. A solution of the appropriate steroid (0.5 g, 1.27 mmol) and thiobenzoic acid in THF (5 mL) were then transferred dropwise via cannula and allowed to react for 1 h at 0°C, followed by an additional hour at room temperature. The reaction was quenched with 30 mL of saturated bicarbonate and the product was extracted with 30 mL ethyl acetate and concentrated *in vacuo*. Purification of the crude product was accomplished using flash chromatography (40% EtOAc in hexanes) to give a white solid.

S-(2-((8S,9S,10R,11S,13S,14S,17R)-11,17-dihydroxy-10,13-dimethyl-3-oxo-2,3,6,7,8,9,10,11,12,13,14,15,16,17-tetradecahydro-1H-cyclopenta[a]phenanthren-17-yl)-2-oxoethyl) benzothioate (HCbz): ^{1}H NMR (500 MHz, $CDCl_3$) δ 7.97 (d, *J* = 8.4 Hz, 2H), 7.60 (t, *J* = 7.5 Hz, 1H), 7.43 – 7.48 (m, 2H), 5.70 (s, 1H), 4.49 (d, *J* = 16.6 Hz, 2H), 3.72 (d, *J* = 16.8 Hz, 1H), 2.97 (s, 1H), 2.87 (s, 1H), 2.45 – 2.55 (m, 2H), 2.37 (dt, *J* = 16.8, 4.2 Hz, 1H), 2.15 – 2.29 (m, 3H), 1.99 – 2.11 (m, 2H), 1.80 – 1.93 (m, 3H), 1.75 (d, *J* = 11.1 Hz, 1H), 1.59 (s, 1H), 1.54 (s, 2H), 1.46 (s, 3H), 1.17 (d, *J* = 12.8 Hz, 1H), 1.08 (d, *J* = 11.1 Hz, 1H), 1.01 (s, 3H); HRMS (ESI) m/z: $[M+H]^+$ Calcd for $C_{28}H_{34}O_5S$, 483.2205; found 483.2203.

S-(2-((8S,9R,10S,11S,13S,14S,16R,17R)-9-fluoro-11,17-dihydroxy-10,13,16-trimethyl-3-oxo-6,7,8,9,10,11,12,13,14,15,16,17-dodecahydro-3H-cyclopenta[a]phenanthren-17-yl)-2-oxoethyl) benzothioate (DXbz): ^{1}H NMR (500 MHz, $CDCl_3$) δ 7.96 (d, *J* = 7.3 Hz, 2H), 7.60 (t, *J* = 7.5 Hz, 1H), 7.40 – 7.51 (m, 2H), 7.20 (d, *J* = 10.1 Hz, 1H), 6.35 (d, *J* = 10.1 Hz, 1H), 6.13 (s, 1H), 4.47 (d, *J* = 16.6 Hz, 1H), 4.42 (d, *J* = 9.5 Hz, 1H), 3.59 (d, *J* = 16.8 Hz, 1H), 3.20 (s, 1H),

3.17 (s, 1H), 2.62 (d, J = 13.7 Hz, 2H), 2.29 – 2.44 (m, 3H), 1.82 – 1.88 (m, 1H), 1.71 – 1.80 (m, 1H), 1.65 (d, J = 12.2 Hz, 2H), 1.58 (s, 2H), 1.51 (t, J = 2.9 Hz, 1H), 1.27 (s, 1H), 1.24 (s, 1H), 1.07 (s, 3H), 0.96 (d, J = 7.2 Hz, 3H); HRMS (ESI) m/z: $[M+H]^+$ Calcd for $C_{29}H_{33}FO_5S$, 513.2111; found 513.2108.

S-(2-((8S,9R,10S,11S,13S,14S,16S,17R)-9-fluoro-11,17-dihydroxy-10,13,16-trimethyl-3-oxo-6,7,8,9,10,11,12,13,14,15,16,17-dodecahydro-3H-cyclopenta[a]phenanthren-17-yl)-2-oxoethyl) benzothioate (BMbz): ^{1}H NMR (500 MHz, cdcl$_3$) δ 7.97 (d, J = 7.2 Hz, 2H), 7.60 (t, J = 7.5 Hz, 1H), 7.43 – 7.49 (m, 2H), 7.23 (d, J = 10.0 Hz, 1H), 6.36 (d, J = 12.0 Hz, 1H), 6.14 (s, 1H), 4.40 (d, J = 11.2 Hz, 1H), 4.12 (d, J = 1.0 Hz, 1H), 2.64 (td, J = 14.1, 6.5 Hz, 1H), 2.40 (d, J = 11.6 Hz, 3H), 2.20 (q, J = 7.7 Hz, 1H), 2.06 – 2.14 (m, 2H), 1.87 – 1.93 (m, 2H), 1.58 – 1.68 (m, 3H), 1.26 (t, J = 7.1 Hz, 4H), 1.19 (s, 1H), 1.17 (s, 2H), 1.15 (s, 4H); HRMS (ESI) m/z: $[M+H]^+$ Calcd for $C_{29}H_{33}FO_5S$, 513.2111; found 513.2106.

S-(2-((8S,9S,10R,11S,13S,14S,17R)-11,17-dihydroxy-10,13-dimethyl-3-oxo-6,7,8,9,10,11,12,13,14,15,16,17-dodecahydro-3H-cyclopenta[a]phenanthren-17-yl)-2-oxoethyl) benzothioate (PNbz): ^{1}H NMR (500 MHz, cdcl$_3$) δ 7.96 (d, J = 7.2 Hz, 2H), 7.60 (t, J = 7.5 Hz, 1H), 7.51 – 7.42 (m, 2H), 7.28 (s, 1H), 6.28 (d, J = 10.1 Hz, 1H), 6.03 (s, 1H), 4.46 (d, J = 16.8 Hz, 1H), 3.74 (d, J = 16.8 Hz, 1H), 2.91 (s, 1H), 2.86 (t, J = 11.6 Hz, 1H), 2.55 – 2.62 (m, 1H), 2.36 (d, J = 10.6 Hz, 1H), 2.24 – 2.10 (m, 3H), 2.04 (s, 1H), 1.84 – 1.91 (m, 1H), 1.78 (dd, J = 30.1, 11.8 Hz, 2H), 1.47 (s, 4H), 1.26 (t, J = 7.2 Hz, 2H), 1.15 (dd, J = 11.2, 3.4 Hz, 2H), 1.02 (s, 3H); Calcd for $C_{28}H_{32}O_5S$, 481.2049; found 481.2042.

S-(2-((6S,8S,9R,10S,11S,13S,14S,16R,17R)-6,9-difluoro-11,17-dihydroxy-10,13,16-trimethyl-3-oxo-6,7,8,9,10,11,12,13,14,15,16,17-dodecahydro-3H-cyclopenta[a]phenanthren-17-yl)-2-oxoethyl) benzothioate (FMbz): ^{1}H NMR (500 MHz, CDCl$_3$) δ 7.95 (d, J = 8.6 Hz, 2H), 7.60 (t, J = 7.5 Hz, 1H), 7.46 (t, J = 7.4 Hz, 2H), 7.13 (d, J = 10.1 Hz, 1H), 6.44 (s, 1H), 6.38 (d, J = 10.1 Hz, 1H), 5.30 – 5.46 (m, 1H), 4.46 (d, J = 16.7 Hz, 1H), 4.42 (ddd, J = 9.2, 4.4, 2.3 Hz, 1H), 3.58 (d, J = 16.7 Hz, 1H), 3.24 (s, 1H), 2.63 (d, J = 14.5 Hz, 1H), 2.37 – 2.52 (m, 2H), 2.26 (d, J = 3.9 Hz, 1H), 1.74 – 1.83 (m, 2H), 1.67 (d, J = 14.8 Hz, 1H), 1.62 (t, J = 2.9 Hz, 1H), 1.59 (s, 2H), 1.30 – 1.33 (m, 1H), 1.27 (s, 1H), 1.24 (s, 1H), 1.06 (s, 3H), 0.97 (d, J = 7.2 Hz, 3H); Calcd for $C_{29}H_{32}F_2O_5S$, 531.2017; found 531.2020.

General procedure for compound 3 derivatization: A solution of steroid S-thiobenzoate (0.937 mmol) in MeOH (25 mL) and 1 N NaOH (2.5 mL) was stirred for 30 min at room temperature. Iodomethane (0.319 g, 0.140 mL, 2.25 mmol) was added and the reaction proceeded for 3 h at room temperature. The reaction was quenched with water (50 mL) and the product was extracted with ethyl acetate. Purification of the crude product was accomplished using flash chromatography (40% EtOAc in hexanes) to give a white product.

(8S,9S,10R,11S,13S,14S,17R)-11,17-dihydroxy-10,13-dimethyl-17-(2-(methylthio)acetyl)-1,2,6,7,8,9,10,11,12,13,14,15,16,17-tetradecahydro-3H-cyclopenta[a]phenanthren-3-one (HC3): white solid obtained in 33% yield; mp 226.6 - 231.3 °C; TLC (Silica G w/UV254) R_f = 0.52, 10:1 CH_2Cl_2:MeOH; $[\alpha]_D^{20}$ = +122.06 (c 0.89, CH_2Cl_2); ^{1}H NMR (500 MHz, CDCl$_3$) δ 5.69 (s, 1H), 4.47 (s, 1H), 3.49 (d, J = 13.6 Hz, 1H), 3.28 (d, J = 13.6 Hz, 1H), 2.73 (t, J = 11.7 Hz, 1H), 2.42 – 2.51 (m, 2H), 2.36 (dt, J = 16.8, 4.3 Hz, 1H), 2.25 (d, J = 17.4 Hz, 1H), 2.19 (s, 1H), 2.16 (t, J = 4.5 Hz, 1H), 2.12 (s, 2H), 2.05 (d, J = 14.2 Hz, 3H), 1.86 (td, J = 13.4, 4.3 Hz, 2H), 1.80 – 1.72 (m,

1H), 1.64 – 1.53 (m, 3H), 1.49 (d, *J* = 11.8 Hz, 1H), 1.44 (s, 3H), 1.25 (s, 1H), 1.14 (d, *J* = 9.3 Hz, 1H), 1.04 (d, *J* = 11.1 Hz, 1H), 0.99 (d, *J* = 17.5 Hz, 3H); ^{13}C NMR (126 MHz, $CDCl_3$) δ 206.13, 199.39, 171.87, 122.44, 89.57, 68.49, 56.00, 51.59, 48.06, 39.95, 39.74, 39.21, 35.05, 34.81, 33.81, 32.72, 32.07, 31.43, 24.00, 21.05, 17.91, 15.80; IR (NaCl, thin film) ν_{max} (cm^{-1}): 3430, 2933, 2360, 1664, 1298, 760; HRMS (ESI) m/z: $[M+H]^+$ Calcd for $C_{22}H_{32}O_4S$, 393.2094; found 393.2097.

(8S,9R,10S,11S,13S,14S,16R,17R)-9-fluoro-11,17-dihydroxy-10,13,16-trimethyl-17-(2-(methylthio)acetyl)-6,7,8,9,10,11,12,13,14,15,16,17-dodecahydro-3H-cyclopenta[a]phenanthren-3-one (DX3): white solid obtained in 65.1% yield; mp 194.8 - 198.3 °C; TLC (Silica G w/UV254) R_f = 0.52, 10:1 CH_2Cl_2:MeOH; $[\alpha]_D^{20}$ = +128.40 (c 1, CH_2Cl_2); ^{1}H NMR (500 MHz, $CDCl_3$) δ 7.18 (d, *J* = 10.1 Hz, 1H), 6.34 (d, *J* = 8.2 Hz, 1H), 6.13 (s, 1H), 4.36 (d, *J* = 7.6 Hz, 1H), 3.55 (d, *J* = 12.8 Hz, 1H), 3.07 (s, 1H), 3.03 (d, *J* = 12.7 Hz, 1H), 2.49 – 2.65 (m, 2H), 2.28 – 2.41 (m, 3H), 2.16 (d, *J* = 12.4 Hz, 1H), 2.09 (s, 2H), 1.81 (d, *J* = 11.1 Hz, 1H), 1.75 (d, *J* = 10.8 Hz, 1H), 1.57 (d, *J* = 12.4 Hz, 2H), 1.54 (s, 4H), 1.36 (d, *J* = 16.6 Hz, 1H), 1.23 – 1.27 (m, 1H), 1.06 (d, *J* = 8.1 Hz, 3H), 0.92 (d, *J* = 7.2 Hz, 3H); ^{13}C NMR (126 MHz, $CDCl_3$) δ 205.40, 186.48, 165.85, 151.76, 129.97, 125.21, 100.75, 91.34, 72.14, 62.54, 48.69, 43.86, 39.06, 37.33, 36.05, 34.21, 32.39, 31.05, 27.32, 22.95, 17.42, 15.56, 14.59; IR (NaCl, thin film) ν_{max} (cm^{-1}): 3454, 2939, 1660, 1239, 891; HRMS (ESI) m/z: $[M+H]^+$ Calcd for $C_{23}H_{31}FO_4S$, 423.2000; found 423.2002.

(8S,9R,10S,11S,13S,14S,16S,17R)-9-fluoro-11,17-dihydroxy-10,13,16-trimethyl-17-(2-(methylthio)acetyl)-6,7,8,9,10,11,12,13,14,15,16,17-dodecahydro-3H-cyclopenta[a]phenanthren-3-one (BM3): white solid obtained in 24.4% yield; mp 186.2 - 191.5°C; TLC (Silica G w/UV254) R_f = 0.46, 10:1 CH_2Cl_2:MeOH; $[\alpha]_D^{20}$ = + 106.47 (c 0.77, CH_2Cl_2); ^{1}H NMR (500 MHz, $CDCl_3$) δ 7.17 (d, *J* = 10.1 Hz, 1H), 6.33 (d, *J* = 9.0 Hz, 1H), 6.12 (s, 1H), 4.38 (d, *J* = 14.0 Hz, 1H), 3.32 (dd, *J* = 118.5, 12.7 Hz, 1H), 2.80 (d, *J* = 13.7 Hz, 1H), 2.63 (t, *J* = 13.6 Hz, 1H), 2.32 – 2.55 (m, 3H), 2.18 (s, 2H), 1.99 – 2.13 (m, 3H), 1.91 (s, 2H), 1.66 (dd, *J* = 22.2, 11.1 Hz, 2H), 1.55 (t, *J* = 12.5 Hz, 5H), 1.42 (dd, *J* = 33.0, 14.5 Hz, 1H), 1.28 (d, *J* = 25.6 Hz, 2H), 1.16 (s, 1H), 1.13 (d, *J* = 6.9 Hz, 3H); ^{13}C NMR (126 MHz, $CDCl_3$) δ 205.36, 186.54, 165.99, 151.95, 129.89, 125.18, 89.63, 72.53, 49.30, 48.97, 48.34, 45.32, 43.56, 41.30, 37.74, 34.99, 33.98, 31.08, 27.59, 22.95, 19.96, 17.64, 15.63; IR (NaCl, thin film) ν_{max} (cm^{-1}): 3384, 2932, 1658, 1051, 703; HRMS (ESI) m/z: $[M+H]^+$ Calcd for $C_{23}H_{31}FO_4S$, 423.2000; found 423.2001.

(8S,9S,10R,11S,13S,14S,17R)-11,17-dihydroxy-10,13-dimethyl-17-(2-(methylthio)acetyl)-6,7,8,9,10,11,12,13,14,15,16,17-dodecahydro-3H-cyclopenta[a]phenanthren-3-one (PN3): white solid obtained in 42% yield; mp 232.9 – 233.0°C; TLC (Silica G w/UV254) R_f = 0.46, 10:1 CH_2Cl_2:MeOH; $[\alpha]_D^{20}$ = +98.83 (c 0.83, CH_2Cl_2); ^{1}H NMR (500 MHz, $CDCl_3$) δ 7.24 (d, *J* = 10.1 Hz, 1H), 6.27 (d, *J* = 10.1 Hz, 1H), 6.02 (s, 1H), 4.49 (s, 1H), 3.39 – 3.53 (m, 1H), 3.27 (d, *J* = 13.6 Hz, 1H), 2.72 (ddd, *J* = 14.6, 11.3, 3.1 Hz, 1H), 2.61 – 2.53 (m, 1H), 2.34 (d, *J* = 11.6 Hz, 1H), 2.18 (d, *J* = 8.7 Hz, 1H), 2.12 (s, 2H), 1.81 – 1.87 (m, 1H), 1.72 (t, *J* = 11.2 Hz, 1H), 1.53 – 1.62 (m, 3H), 1.45 (s, 3H), 1.26 (s, 2H), 1.08 – 1.15 (m, 2H), 1.02 (s, 2H), 0.99 (s, 1H), 0.85 (s, 3H); ^{13}C NMR (126 MHz, $CDCl_3$) δ 186.46, 169.70, 155.84, 128.00, 122.53, 89.52, 77.22, 70.37, 55.27, 51.00, 48.26, 44.00, 39.94, 39.70, 34.82, 33.96, 32.01, 31.28, 24.22, 21.13, 17.82, 15.78; HRMS (ESI) m/z: $[M+H]^+$ Calcd for $C_{22}H_{30}O_4S$, 391.1936; found 391.1933.

(6S,8S,9R,10S,11S,13S,14S,16R,17R)-6,9-difluoro-11,17-dihydroxy-10,13,16-trimethyl-17-(2-(methylthio)acetyl)-6,7,8,9,10,11,12,13,14,15,16,17-dodecahydro-3H-cyclopenta[a]phenanthren-3-one (FM3): tan solid obtained in 45.9% yield; mp 142.9-155.2; TLC (Silica G w/UV254) R_f = 0.56, 10:1 CH_2Cl_2:MeOH; $[\alpha]_D^{20}$ = + 138.84 (c 0.41, CH_2Cl_2); ^1H NMR (500 MHz, $CDCl_3$) δ 7.10 (d, *J* = 10.1 Hz, 1H), 6.42 (s, 1H), 6.36 (d, *J* = 10.2 Hz, 1H), 5.37 (ddd, *J* = 48.7, 11.7, 6.6 Hz, 1H), 4.38 (s, 1H), 3.54 (d, *J* = 12.7 Hz, 1H), 3.06 – 3.19 (m, 1H), 2.70 – 3.05 (m, 1H), 2.49 – 2.60 (m, 1H), 2.41 (s, 2H), 2.27 (s, 1H), 2.11 (d, *J* = 29.9 Hz, 2H), 1.67 – 1.86 (m, 2H), 1.54 (d, *J* = 15.7 Hz, 4H), 1.31 – 1.44 (m, 2H), 1.27 (d, *J* = 6.3 Hz, 2H), 1.02 – 1.12 (m, 3H), 0.86 – 1.01 (m, 3H); ^{13}C NMR (126 MHz, $CDCl_3$) δ 205.14, 196.90, 185.52, 150.34, 130.32, 121.26, 91.23, 87.31, 85.84, 71.93, 62.47, 48.68, 43.57, 38.97, 37.16, 36.04, 33.81, 32.83, 32.25, 23.07, 17.35, 15.54, 14.54; HRMS (ESI) m/z: $[M+H]^+$ Calcd for $C_{23}H_{30}F_2O_4S$, 441.1906; found 441.1909.

General procedure for compound 4 derivatization: same as compound 2.

(8S,9S,10R,11S,13S,14S,17R)-11-hydroxy-10,13-dimethyl-17-(2-(methylthio)acetyl)-3-oxo-2,3,6,7,8,9,10,11,12,13,14,15,16,17-tetradecahydro-1H-cyclopenta[a]phenanthren-17-yl furan-2-carboxylate (HC4): white solid obtained in 12.2% yield; mp 152.5-160.8°C; TLC (Silica G w/UV254) R_f = 0.57, 10:1 CH_2Cl_2:MeOH; $[\alpha]_D^{20}$ = + 81.02 (c 0.54, CH_2Cl_2); ^1H NMR (500 MHz, $cdcl_3$) δ 7.62 (t, *J* = 6.4 Hz, 1H), 7.09 – 7.24 (m, 1H), 6.55 (d, *J* = 7.1 Hz, 1H), 5.72 (d, *J* = 7.7 Hz, 1H), 4.54 (s, 1H) 3.37 – 3.54 (m, 1H), 3.31 (s, 1H), 2.99 – 3.16 (m, 1H), 2.76 (s, 1H), 2.47 – 2.56 (m, 2H), 2.31 – 2.43 (m, 2H), 2.23 (d, *J* = 22.3 Hz, 3H), 2.09 (d, *J* = 22.0 Hz, 4H), 1.92 (dd, *J* = 27.1, 10.6 Hz, 3H), 1.79 (d, *J* = 7.8 Hz, 1H), 1.46 (s, 3H), 1.30 (d, *J* = 11.1 Hz, 2H), 1.10 (d, *J* = 11.4 Hz, 1H), 1.05 (s, 2H), 0.92 (d, *J* = 22.1 Hz, 2H); ^{13}C NMR (126 MHz, $cdcl_3$) δ 199.37, 171.69, 157.92, 147.03, 122.49, 119.10, 118.35, 112.12, 96.53, 70.12, 68.25, 55.99, 54.93, 52.94, 47.09, 40.51, 39.22, 35.02, 33.84, 32.62, 32.39, 31.96, 31.49, 30.87, 23.80, 20.98, 17.01; IR (NaCl, thin film) ν_{max} (cm^{-1}): 3482, 2928, 1661, 1300, 761 ; HRMS (ESI) m/z: $[M+H]^+$ Calcd for $C_{27}H_{34}O_6S$, 487.2149; found 487.2156.

(8S,9R,10S,11S,13S,14S,16R,17R)-9-fluoro-11-hydroxy-10,13,16-trimethyl-17-(2-(methylthio)acetyl)-3-oxo-6,7,8,9,10,11,12,13,14,15,16,17-dodecahydro-3H-cyclopenta[a]phenanthren-17-yl furan-2-carboxylate (DX4): white solid obtained in 22.9% yield; mp 158.3-165.3°C; TLC (Silica G w/UV254) R_f = 0.55, 10:1 CH_2Cl_2:MeOH; $[\alpha]_D^{20}$ = + 36.05 (c 0.46, CH_2Cl_2); ^1H NMR (500 MHz, $CDCl_3$) δ 7.60 (d, *J* = 9.9 Hz, 1H), 7.17 (d, *J* = 14.3 Hz, 2H), 6.51 (s, 1H), 6.37 (d, *J* = 10.2 Hz, 1H), 6.16 (s, 1H), 5.71 (d, *J* = 84.1 Hz, 1H), 4.45 (s, 1H), 3.97 – 3.54 (m, 1H), 3.51 – 3.29 (m, 1H), 2.71 – 2.52 (m, 2H), 2.49 – 2.35 (m, 3H), 2.32 – 2.21 (m, 2H), 1.85 (dd, *J* = 22.2, 11.5 Hz, 2H), 1.60 (s, 2H), 1.56 (s, 4H), 1.32 (s, 1H), 1.25 (d, *J* = 6.3 Hz, 3H), 1.18 (s, 1H), 1.10 (t, *J* = 7.8 Hz, 1H), 1.00 (dd, *J* = 24.4, 6.6 Hz, 2H), 0.94 – 0.85 (m, 1H); ^{13}C NMR (126 MHz, $CDCl_3$) δ 186.50, 165.80, 158.05, 151.76, 147.54, 143.52, 130.21, 125.43, 119.32, 112.31, 72.38, 72.06, 60.54, 48.65, 43.95, 37.31, 35.98, 34.30, 34.15, 33.58, 31.11, 27.50, 23.17, 23.12, 21.19, 17.03, 16.56, 14.35; IR (NaCl, thin film) ν_{max} (cm^{-1}): 3392, 2934, 2359, 1660, 1301, 758; HRMS (ESI) m/z: $[M+H]^+$ Calcd for $C_{28}H_{33}FO_6S$, 517.2055; found 517.2058.

(8S,9R,10S,11S,13S,14S,16R,17R)-9-fluoro-11-hydroxy-10,13,16-trimethyl-17-(2-(methylthio)acetyl)-3-oxo-6,7,8,9,10,11,12,13,14,15,16,17-dodecahydro-3H-cyclopenta[a]phenanthren-17-yl furan-2-carboxylate (BM4): white solid obtained in 35.9%

yield; mp 203.5-211; TLC (Silica G w/UV254) R_f = 0.54, 10:1 CH_2Cl_2:MeOH; $[\alpha]_D^{20}$ = + 44.98 (c 1.1, CH_2Cl_2); ^{1}H NMR (500 MHz, $CDCl_3$) δ 7.63 (s, 1H), 7.21 (s, 1H), 6.89 (d, *J* = 10.4 Hz, 1H), 6.55 (s, 1H), 6.33 (d, *J* = 10.2 Hz, 1H), 6.14 (s, 1H), 6.03 (d, *J* = 27.2 Hz, 1H), 5.63 (s, 1H), 3.43 (d, *J* = 12.5 Hz, 1H), 3.07 (d, *J* = 12.5 Hz, 1H), 2.61 (d, *J* = 9.5 Hz, 1H), 2.44 (d, *J* = 8.7 Hz, 3H), 2.16 (s, 2H), 2.10 (t, *J* = 6.8 Hz, 1H), 2.07 (s, 1H), 1.94 (d, *J* = 7.0 Hz, 1H), 1.70 – 1.59 (m, 2H), 1.54 (s, 3H), 1.41 (d, *J* = 7.5 Hz, 3H), 1.26 (s, 2H), 1.19 (s, 1H), 1.16 (d, *J* = 6.7 Hz, 1H), 1.12 (d, *J* = 7.0 Hz, 1H), 1.08 (s, 1H), 1.03 (s, 1H); ^{13}C NMR (126 MHz, $CDCl_3$) δ 205.01, 185.97, 164.44, 156.60, 150.15, 147.33, 144.07, 130.52, 125.49, 119.20, 112.22, 89.42, 72.46, 72.12, 49.55, 48.66, 47.37, 43.20, 41.24, 34.93, 33.58, 31.10, 27.52, 23.11, 19.93, 17.31, 15.93, 15.52; IR (NaCl, thin film) ν_{max} (cm^{-1}): 3384, 2933, 2359, 1700, 1295, 757; HRMS (ESI) m/z: $[M+H]^+$ Calcd for $C_{28}H_{33}FO_6S$, 517.2055; found 517.2061.

(8S,9S,10R,11S,13S,14S,17R)-11-hydroxy-10,13-dimethyl-17-(2-(methylthio)acetyl)-3-oxo-6,7,8,9,10,11,12,13,14,15,16,17-dodecahydro-3H-cyclopenta[a]phenanthren-17-yl furan-2-carboxylate (PN4): white solid obtained in yield 19.9%; mp 159.2-161.3 ; TLC (Silica G w/UV254) R_f = 0.59, 10:1 CH_2Cl_2:MeOH; $[\alpha]_D^{20}$ = 25.7 (c 0.50, CH_2Cl_2); ^{1}H NMR (500 MHz, cdcl$_3$δ 7.61 (d, *J* = 1.0 Hz, 1H), 7.28 (d, *J* = 10.0 Hz, 1H), 7.19 (dd, *J* = 3.5, 0.9 Hz, 1H), 6.53 (dd, *J* = 3.5, 1.7 Hz, 1H), 6.32 (dd, *J* = 10.1, 1.9 Hz, 1H), 6.05 (s, 1H), 4.56 (s, 1H), 3.43 (d, *J* = 15.9 Hz, 1H), 3.28 (d, *J* = 15.9 Hz, 1H), 2.89 – 3.09 (m, 1H), 2.60 (td, *J* = 13.6, 5.5 Hz, 1H), 2.33 – 2.39 (m, 1H), 2.27 (dd, *J* = 14.4, 3.7 Hz, 1H), 2.22 (s, 3H), 2.16 – 2.20 (m, 1H), 2.09 – 2.16 (m, 1H), 1.93 (ddd, *J* = 16.0, 9.3, 6.5 Hz, 1H), 1.85 – 1.89 (m, 1H), 1.79 – 1.84 (m, 1H), 1.74 (dd, *J* = 18.2, 11.0 Hz, 1H), 1.50 – 1.56 (m, 1H), 1.48 (s, 3H), 1.25 (s, 2H), 1.12 – 1.20 (m, 2H), 1.08 (s, 3H)); ^{13}C NMR (126 MHz, $CDCl_3$) δ 200.92, 186.53, 169.79, 157.89, 155.98, 147.11, 144.05, 128.07, 122.61, 119.22, 112.16, 96.55, 70.17, 55.40, 52.41, 47.33, 44.04, 40.43, 39.21, 33.94, 31.94, 31.33, 30.81, 24.02, 21.07, 16.95; IR (NaCl, thin film) ν_{max} (cm^{-1}): 3356, 2922, 1655, 1307, 762; HRMS (ESI) m/z: $[M+H]^+$ Calcd for $C_{27}H_{32}O_6S$, 485.1998; found 485.1982

(6S,8S,9R,10S,11S,13S,14S,16R,17R)-6,9-difluoro-11-hydroxy-10,13,16-trimethyl-17-(2-(methylthio)acetyl)-3-oxo-6,7,8,9,10,11,12,13,14,15,16,17-dodecahydro-3H-cyclopenta[a]phenanthren-17-yl furan-2-carboxylate (FM4): tan solid obtained in 15.9% yield; mp 137.4-155.6; ; TLC (Silica G w/UV254) R_f = 0.59, 10:1 CH_2Cl_2:MeOH; $[\alpha]_D^{20}$ = + 24.91 (c 0.42, CH_2Cl_2); ^{1}H NMR (500 MHz, cdcl$_3$) δ 7.61 (d, *J* = 7.0 Hz, 1H), 7.10 – 7.24 (m, 2H), 6.50 – 6.58 (m, 1H), 6.43 – 6.48 (m, 1H), 6.40 (d, *J* = 10.3 Hz, 1H), 5.31 – 5.76 (m, 2H), 4.43 (s, 1H), 3.31 (s, 1H), 2.50 – 2.69 (m, 3H), 2.23 – 2.33 (m, 3H), 2.19 (s, 1H), 1.85 (dd, *J* = 23.0, 13.8 Hz, 3H), 1.54 (s, 2H), 1.41 (d, *J* = 13.1 Hz, 3H), 1.24 (d, *J* = 10.8 Hz, 2H), 1.17 (s, 1H), 1.09 (s, 1H), 0.95 – 1.02 (m, 3H); ^{13}C NMR (126 MHz, $CDCl_3$) δ 185.41, 157.85, 150.21, 148.80, 147.45, 130.92, 130.42, 124.71, 119.64, 119.27, 112.21, 87.18, 85.72, 71.68, 71.13, 48.50, 48.04, 43.52, 36.95, 35.82, 34.33, 33.69, 33.31, 30.34, 29.71, 23.06, 16.84, 16.48; IR (NaCl, thin film) ν_{max} (cm^{-1}): 3326, 2980, 2359, 1663, 1297, 753; HRMS (ESI) m/z: $[M+H]^+$ Calcd for $C_{28}H_{32}F_2O_6S$, 535.1960; found 535.1963.

2. ^1H and ^{13}C NMR Spectra

^1H NMR of **DX1**

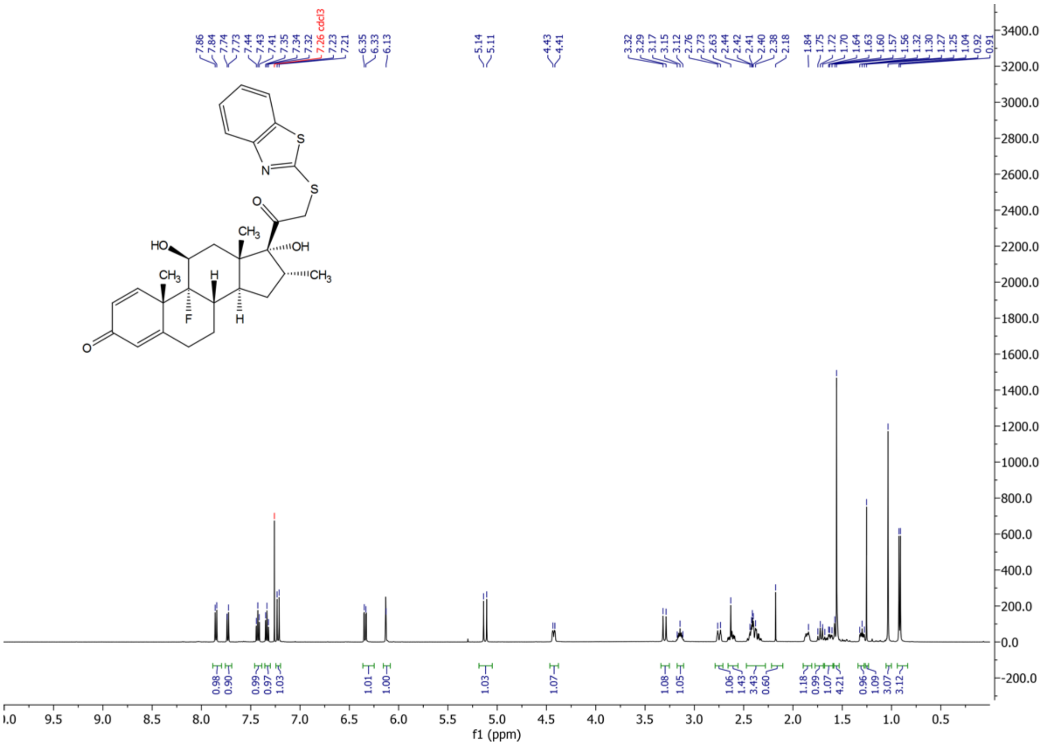

^{13}C NMR of **DX1**

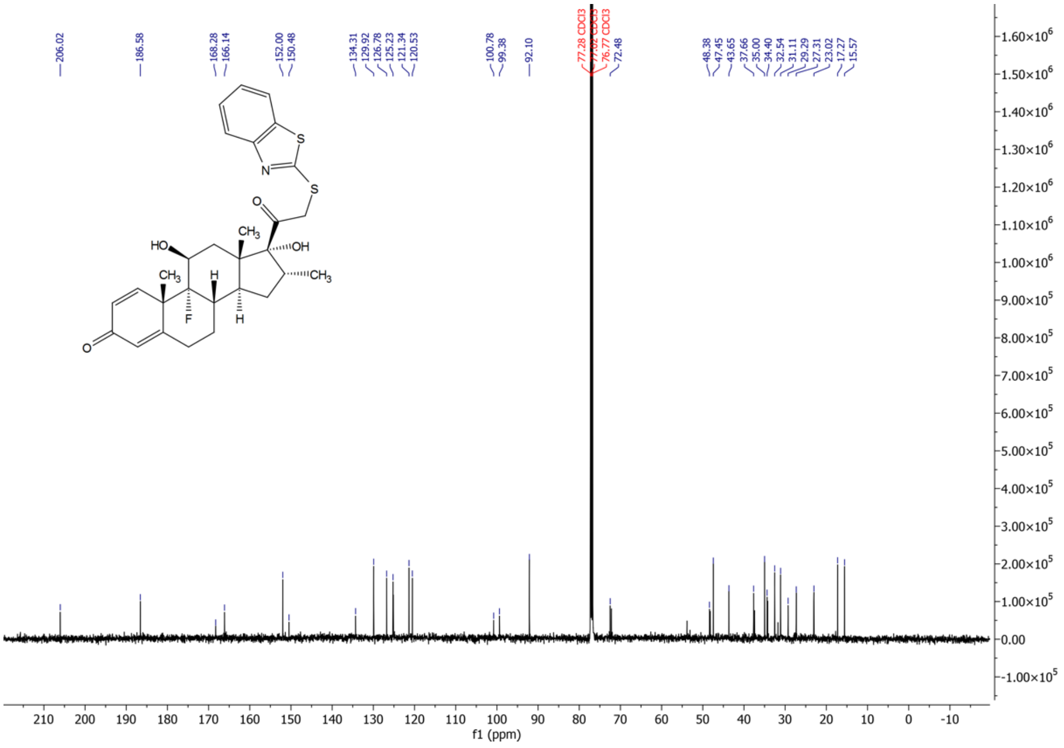

^{1}H NMR of **DX5**

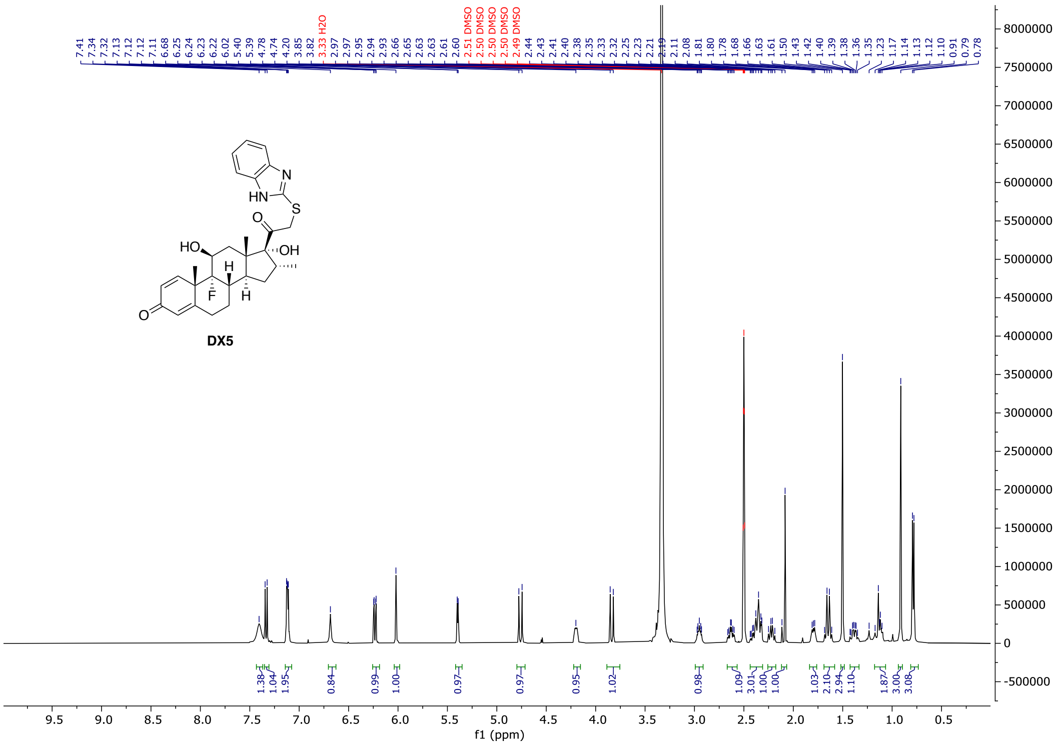

^{13}C NMR of **DX5**

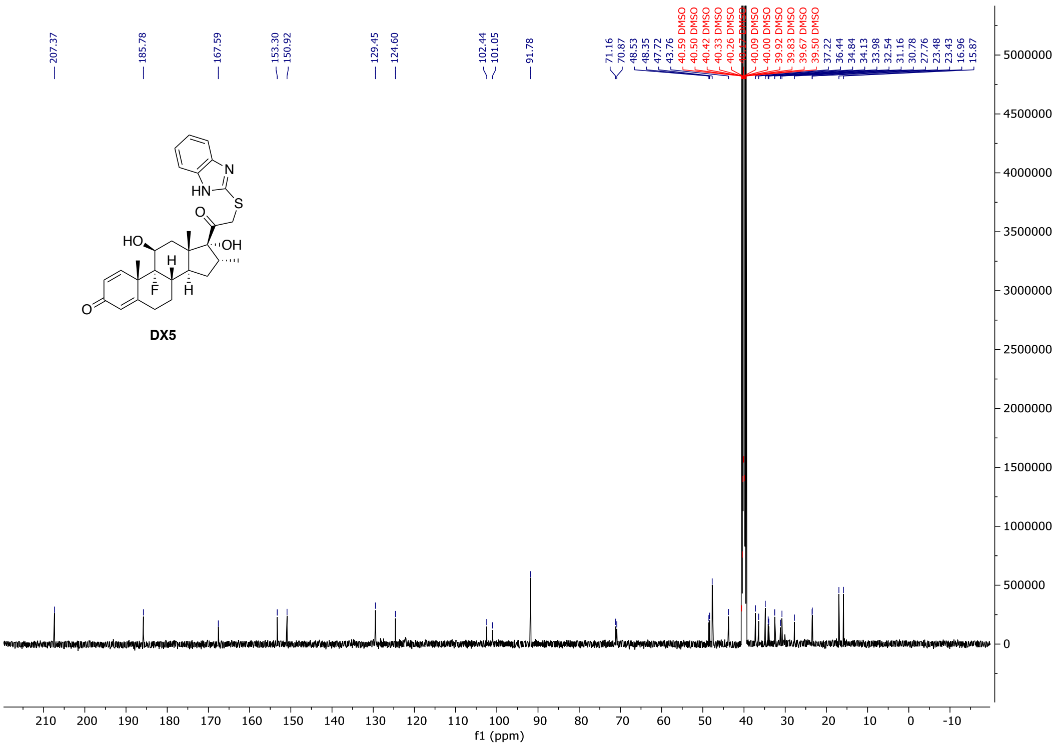

^{1}H NMR of **DX6**

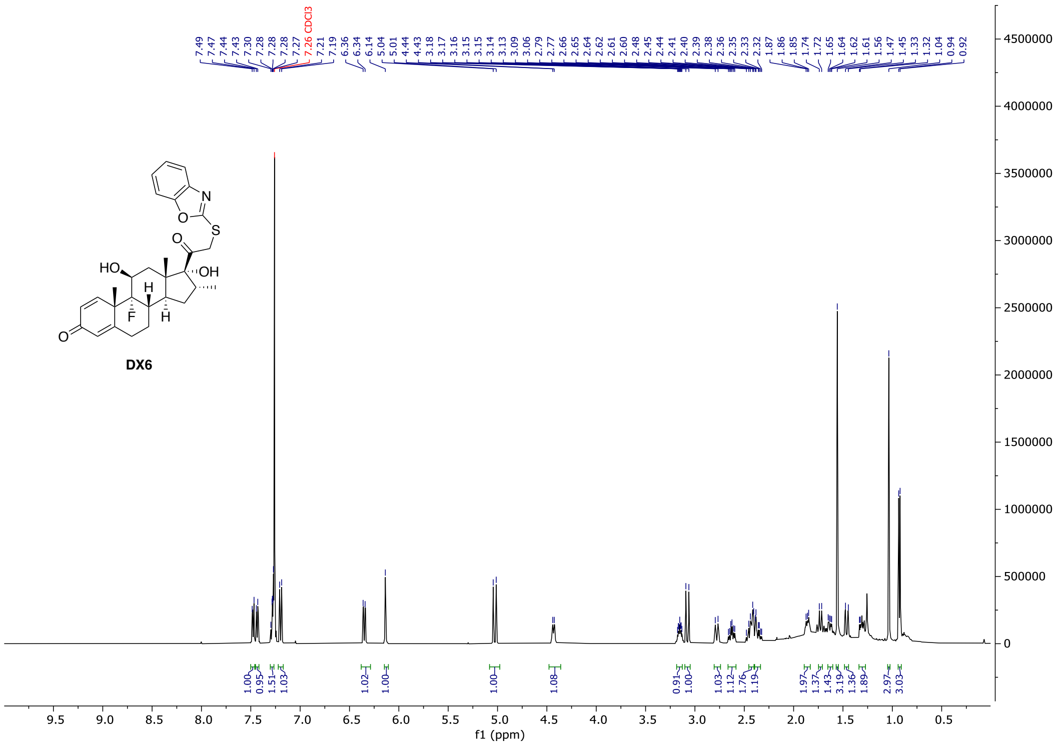

^{13}C NMR of **DX6**

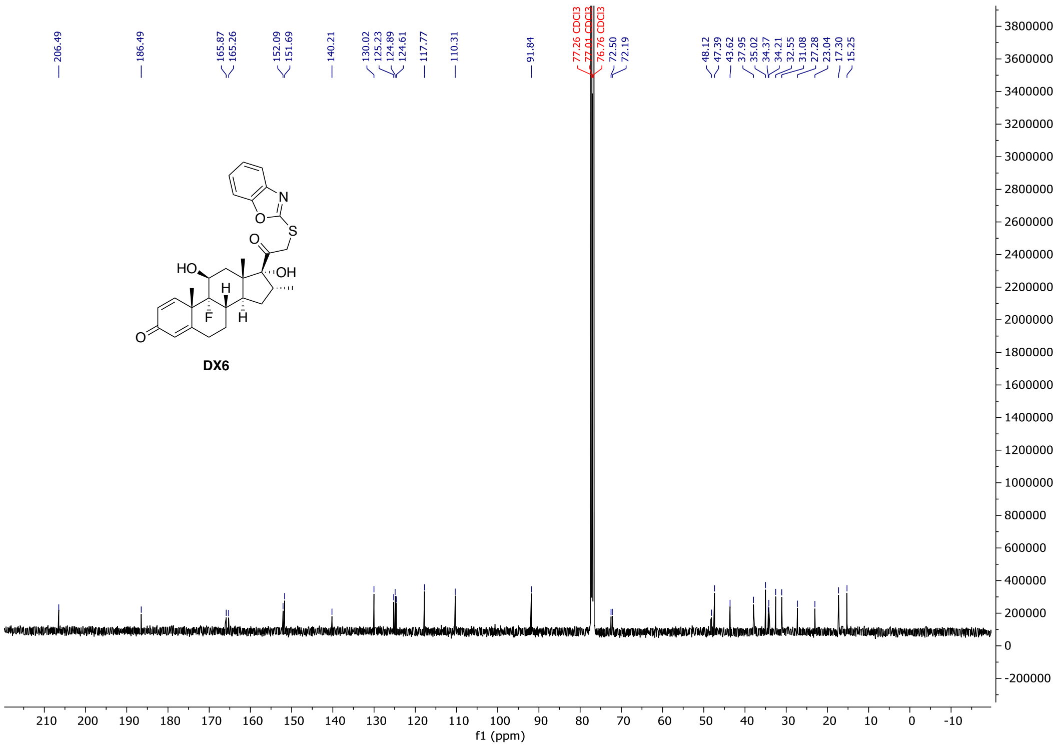

^{1}H NMR of **DX7**

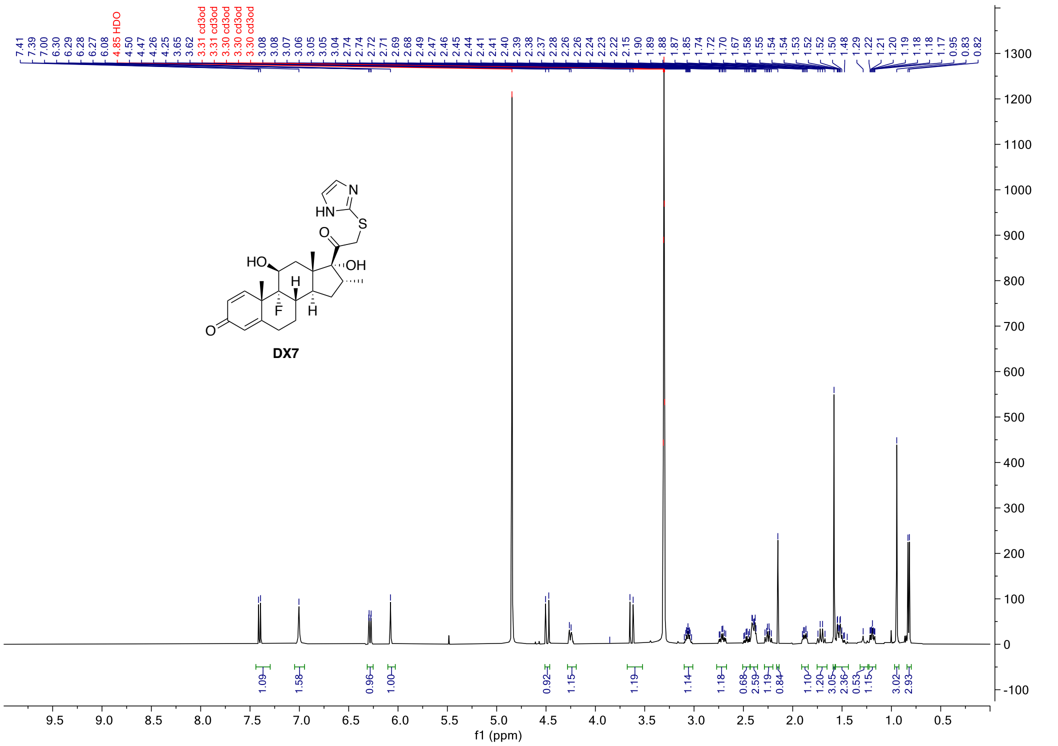

^{13}C NMR of **DX7**

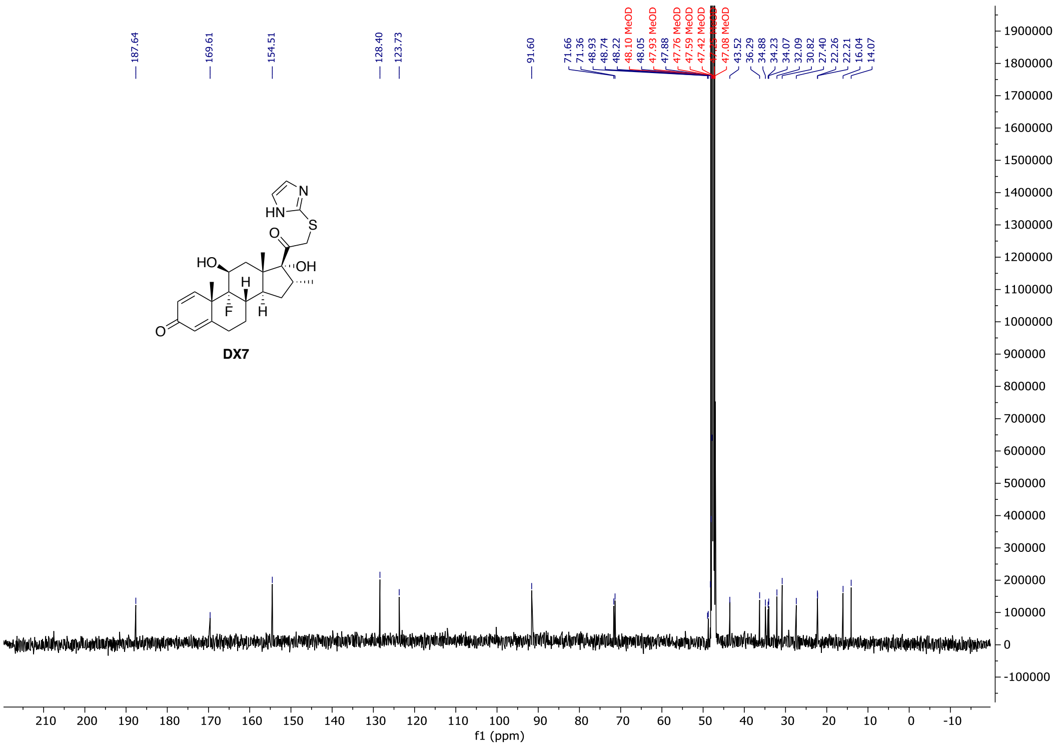

^{1}H NMR of **DX8**

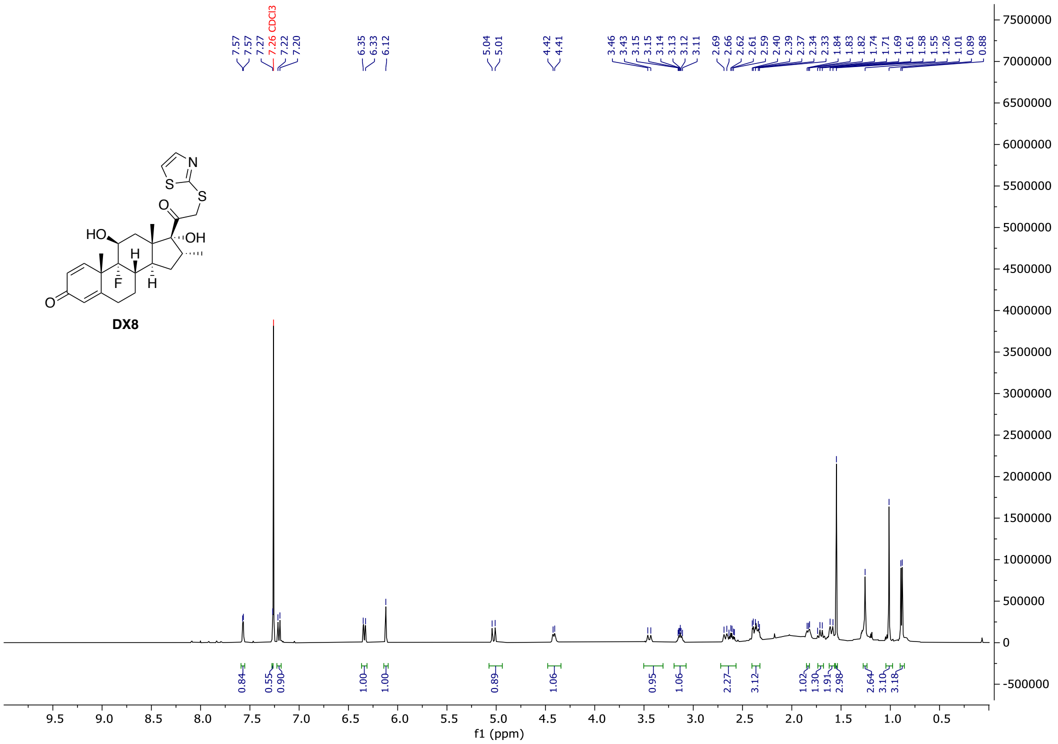

^{13}C NMR of **DX8**

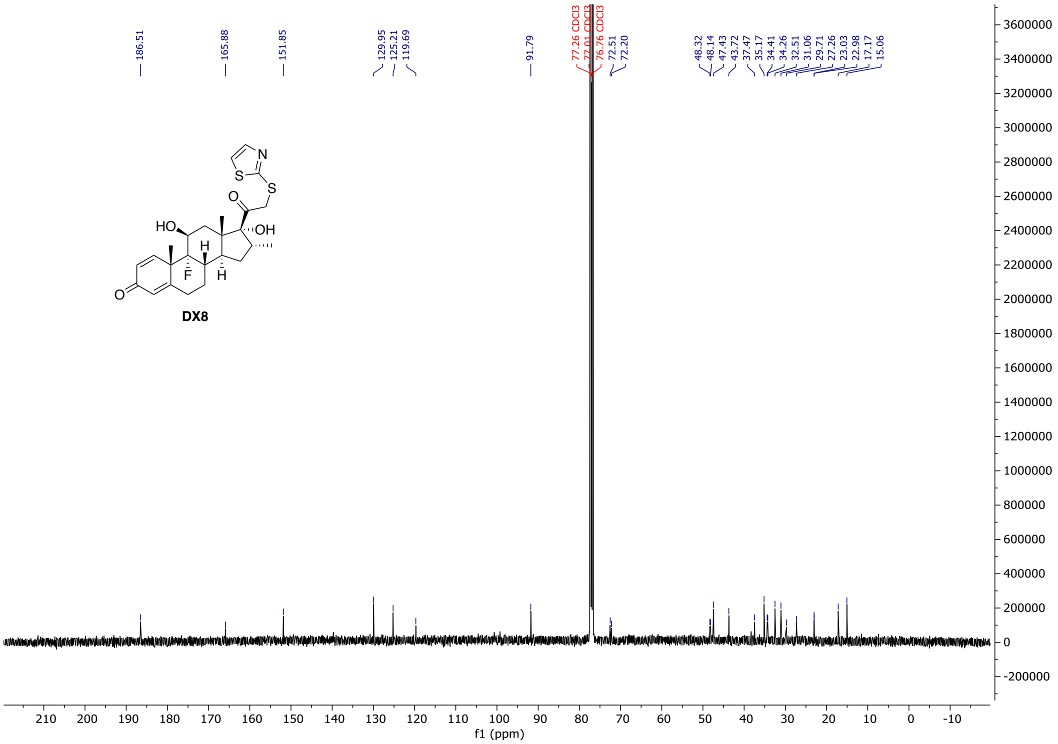

^{1}H NMR of **BM1**

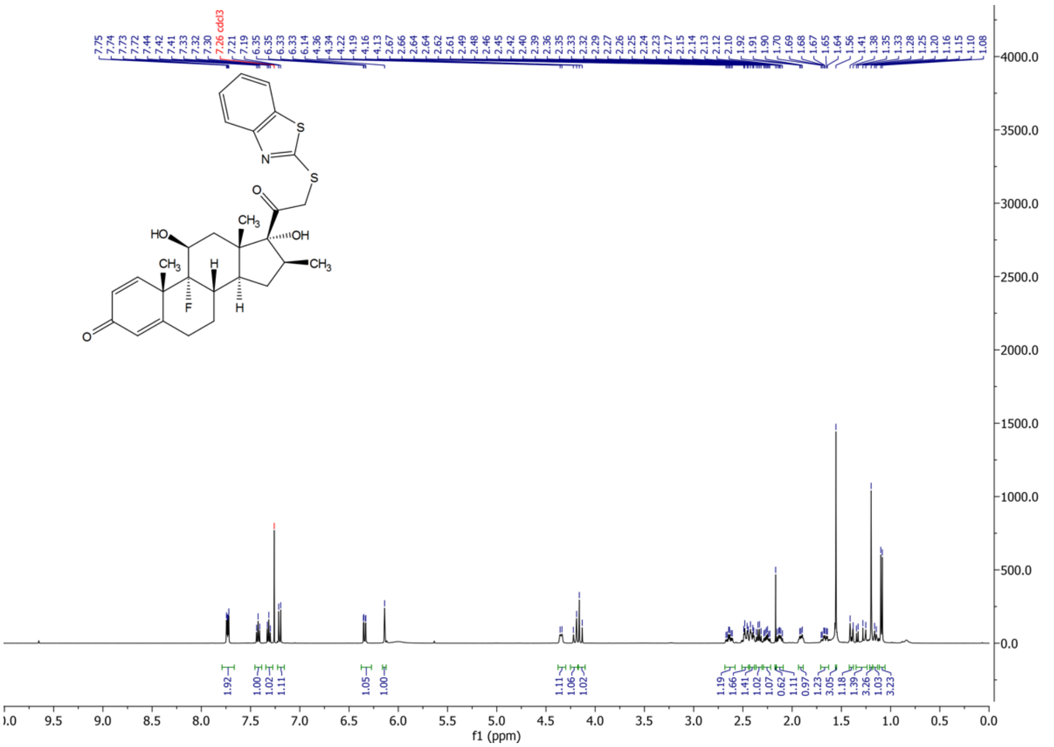

^{13}C NMR of **BM1**

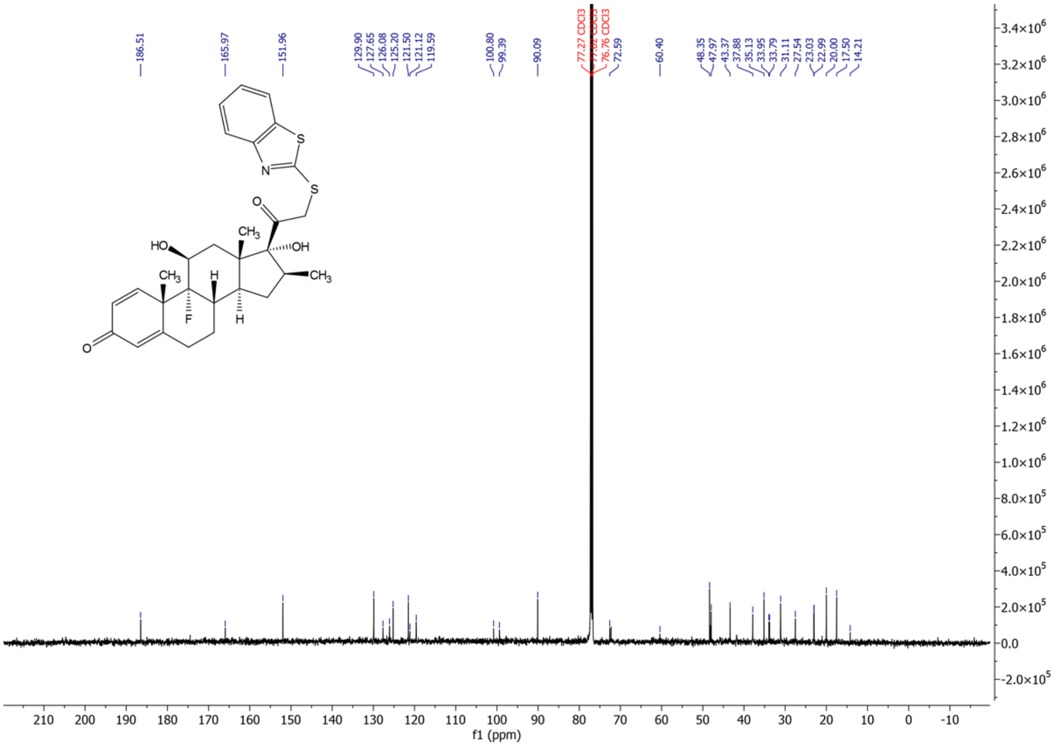

^{1}H NMR of **PN1**

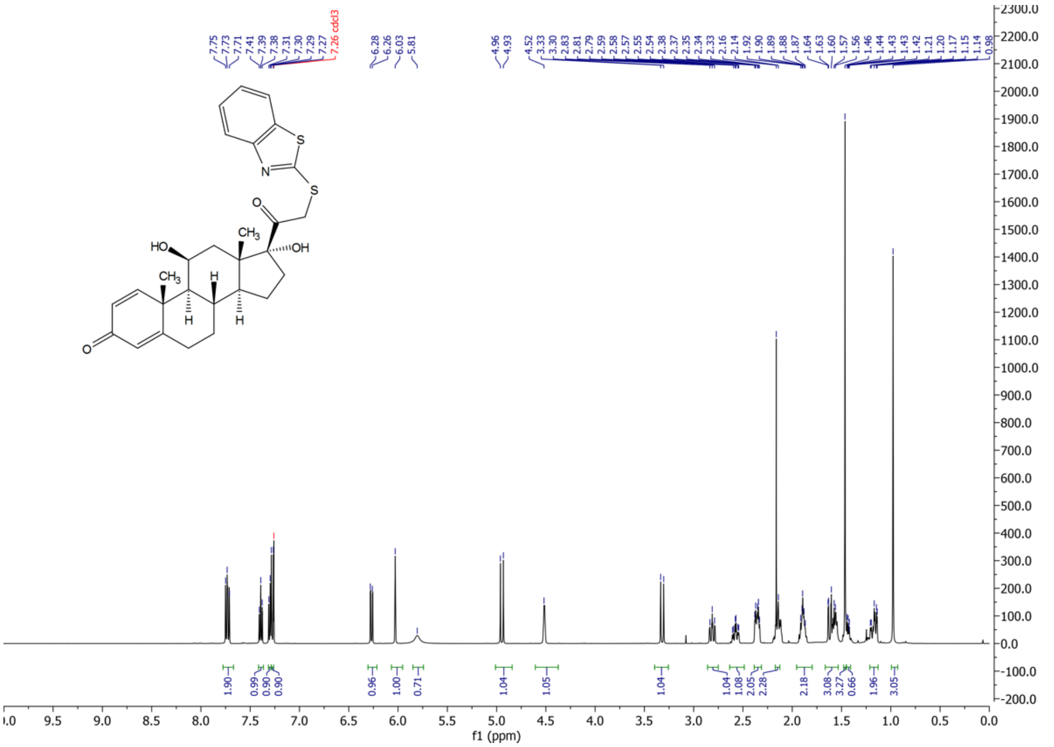

^{13}C NMR of **PN1**

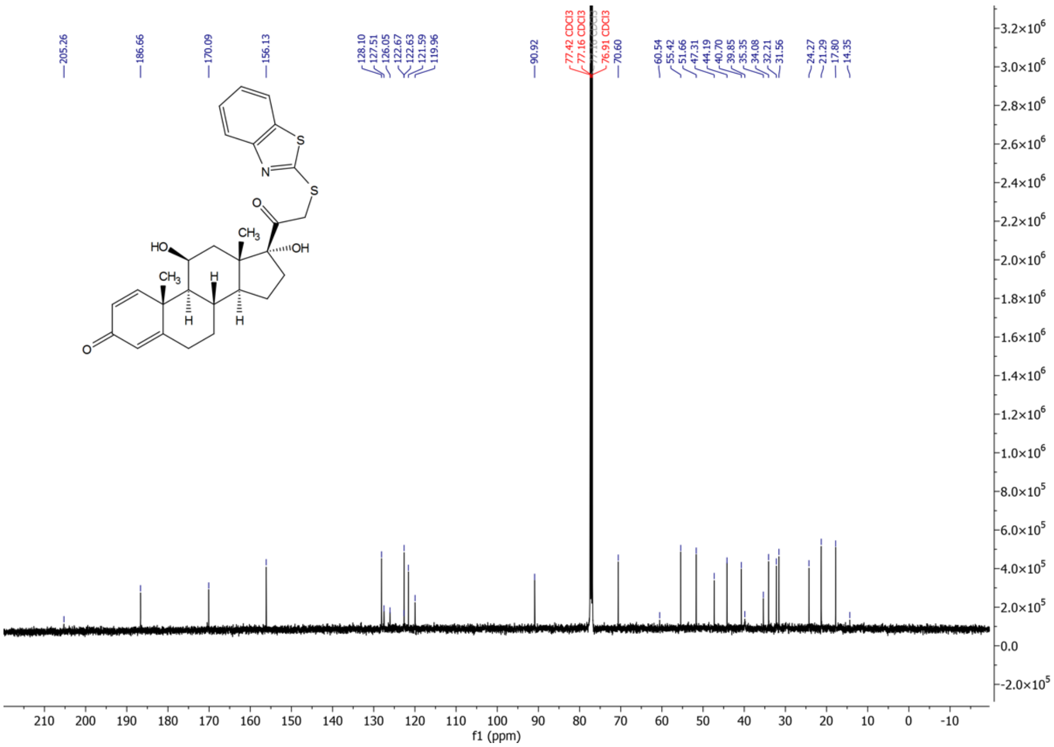

^{1}H NMR of **FM1**

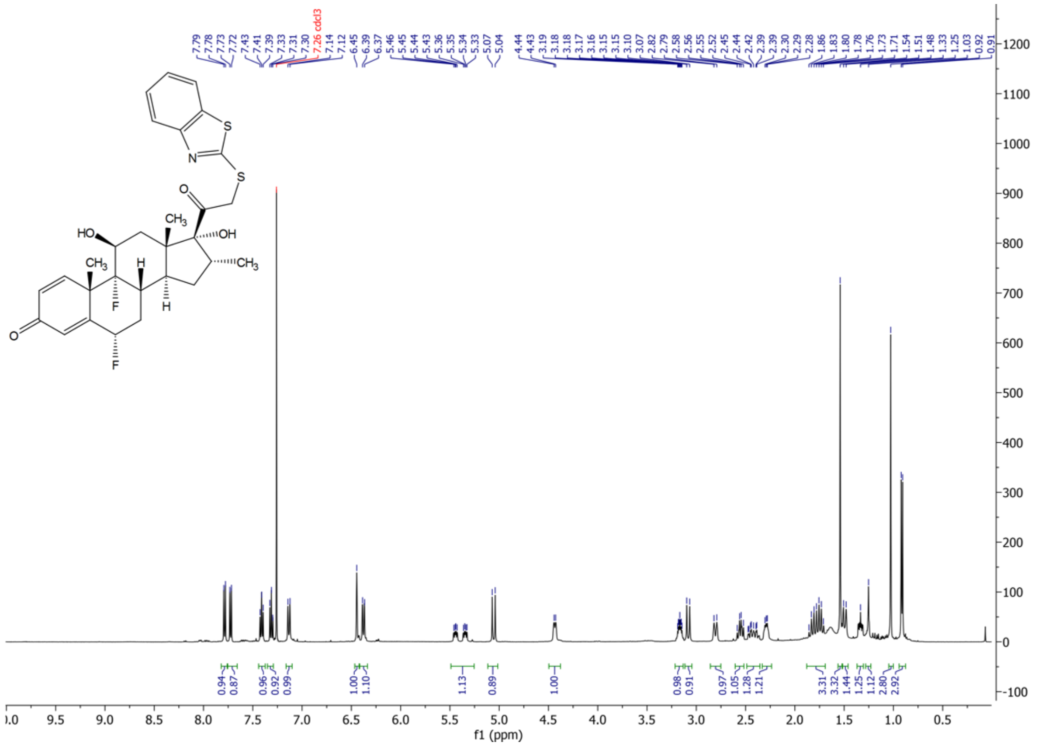

^{13}C NMR of **FM1**

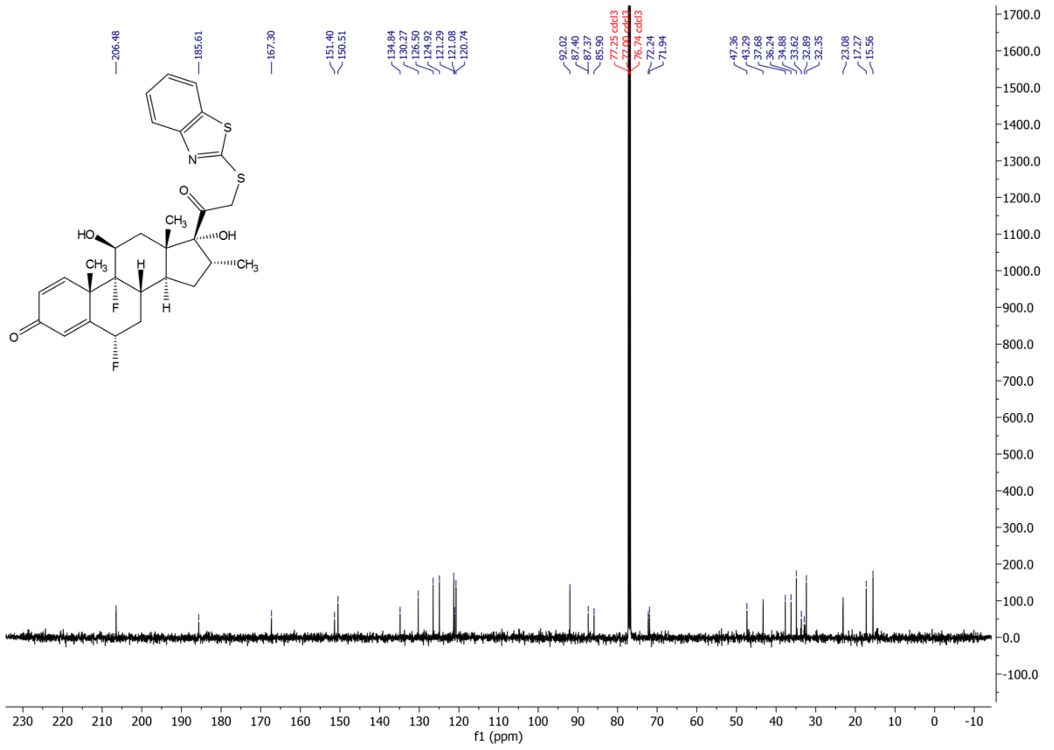

^{1}H NMR of **DN1**

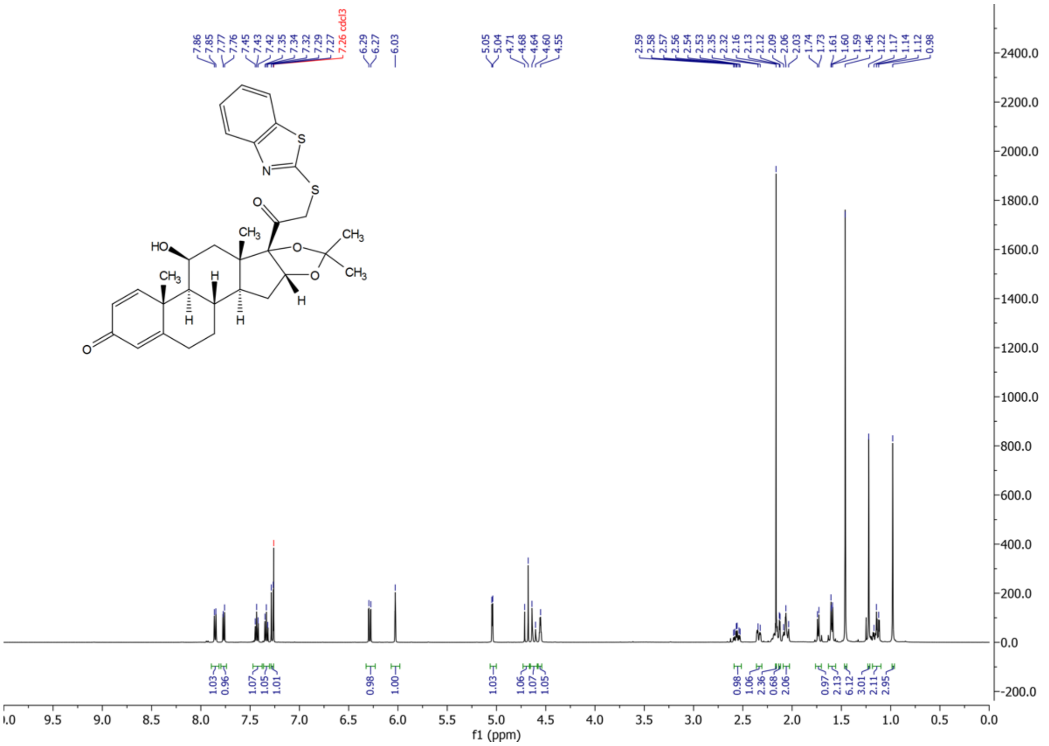

^{13}C NMR of **DN1**

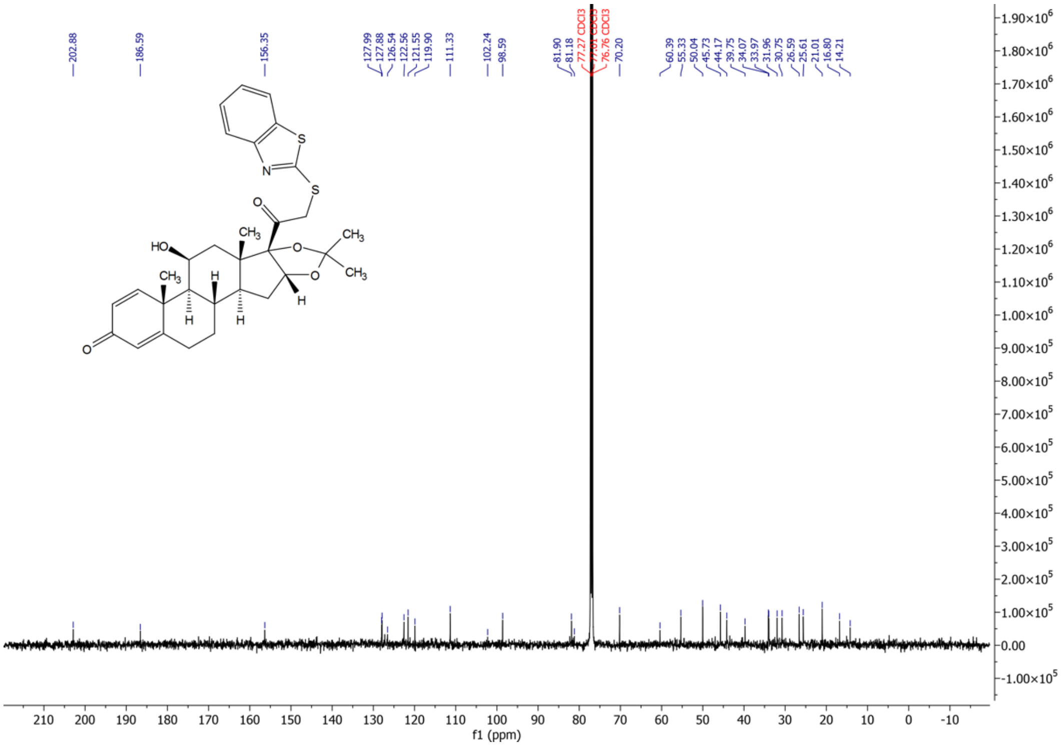

^{1}H NMR of **BD1**

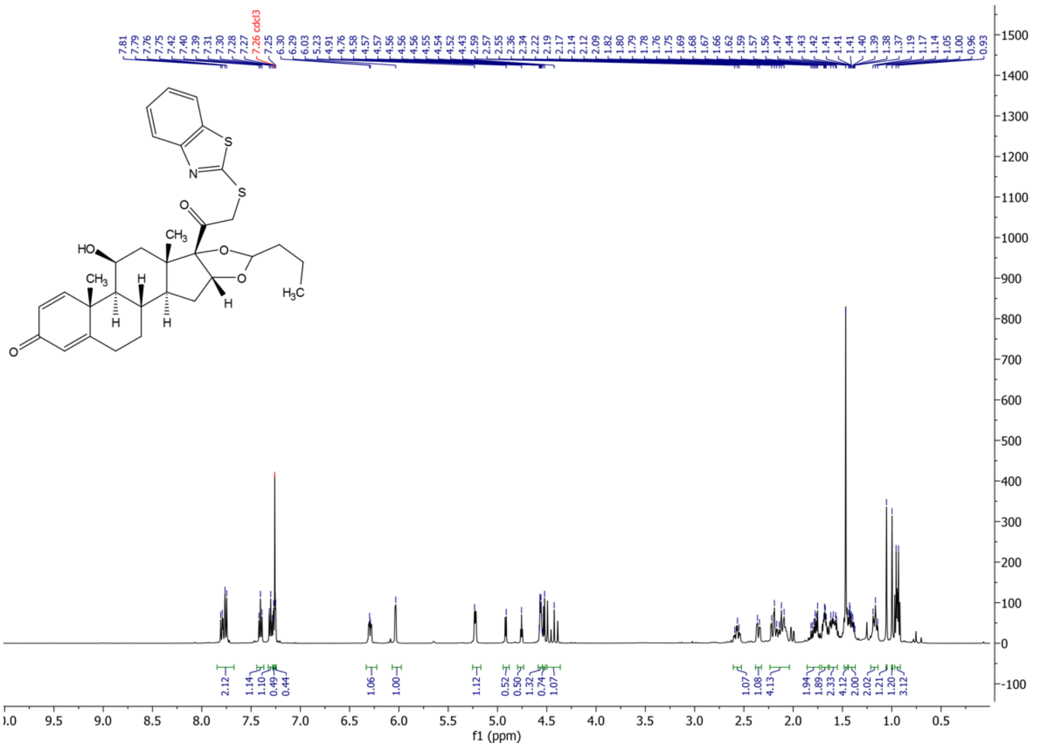

^{13}C NMR of **BD1**

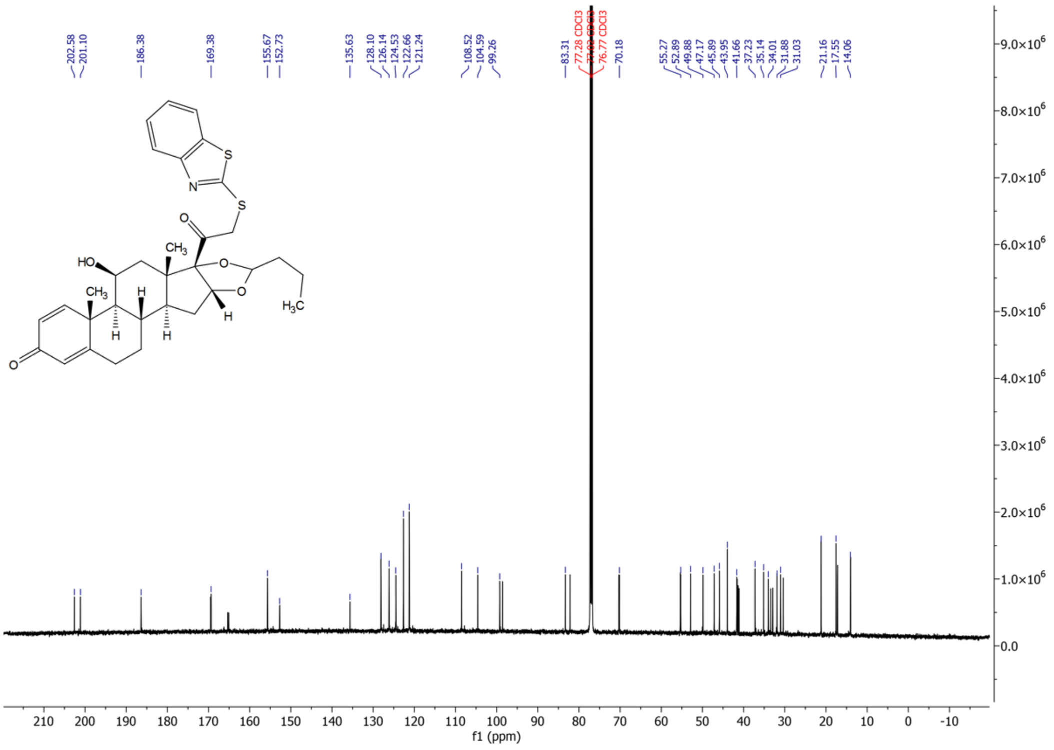

^{1}H NMR of **DX2**

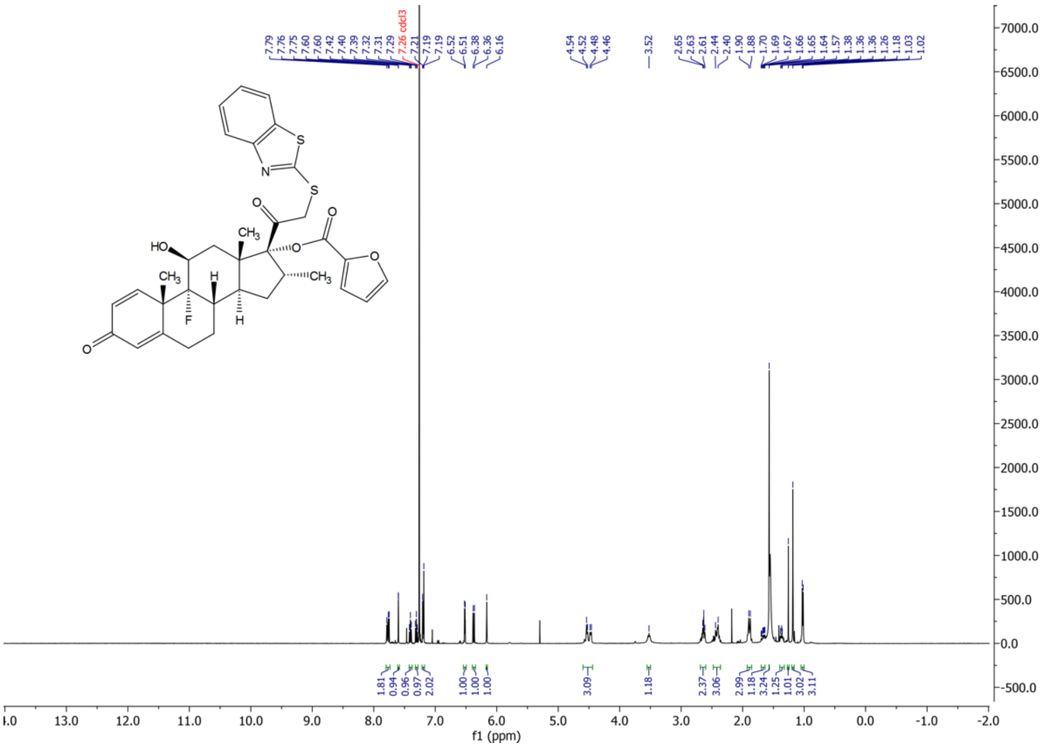

^{13}C NMR of **DX2**

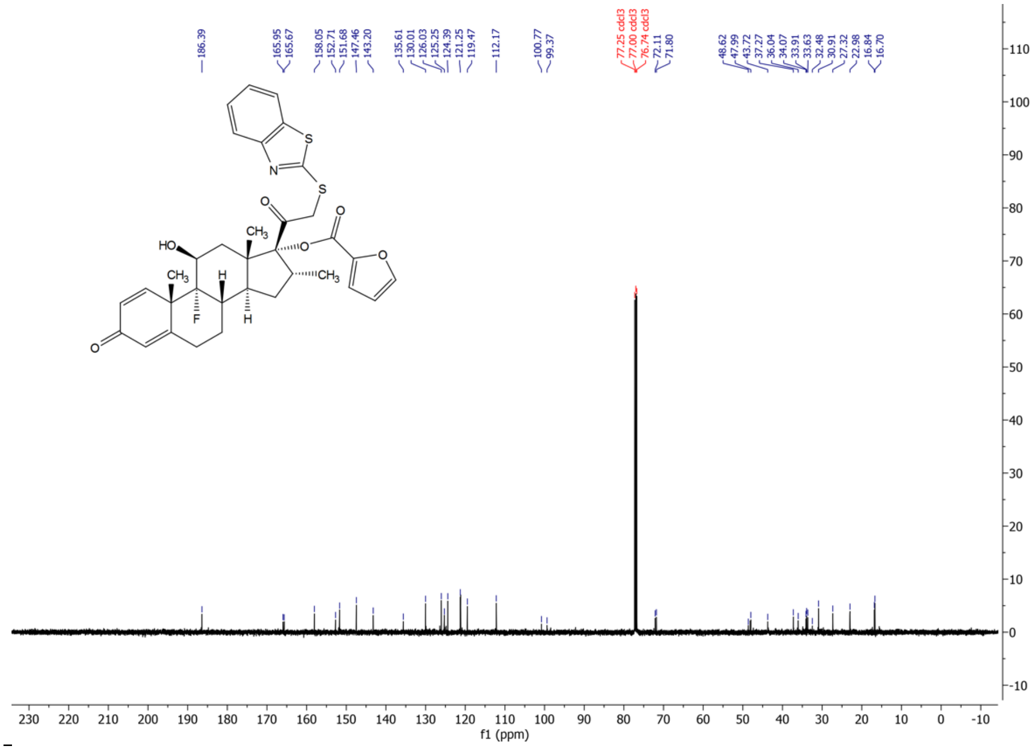

^{1}H NMR of **BM2**

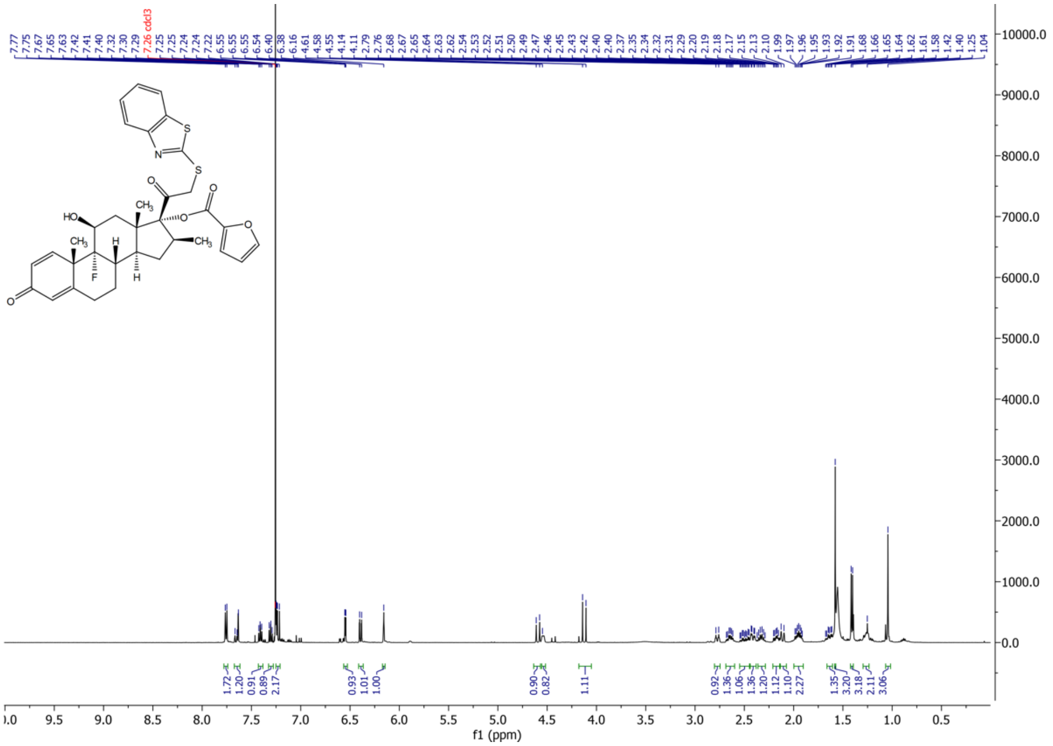

^{13}C NMR of **BM2**

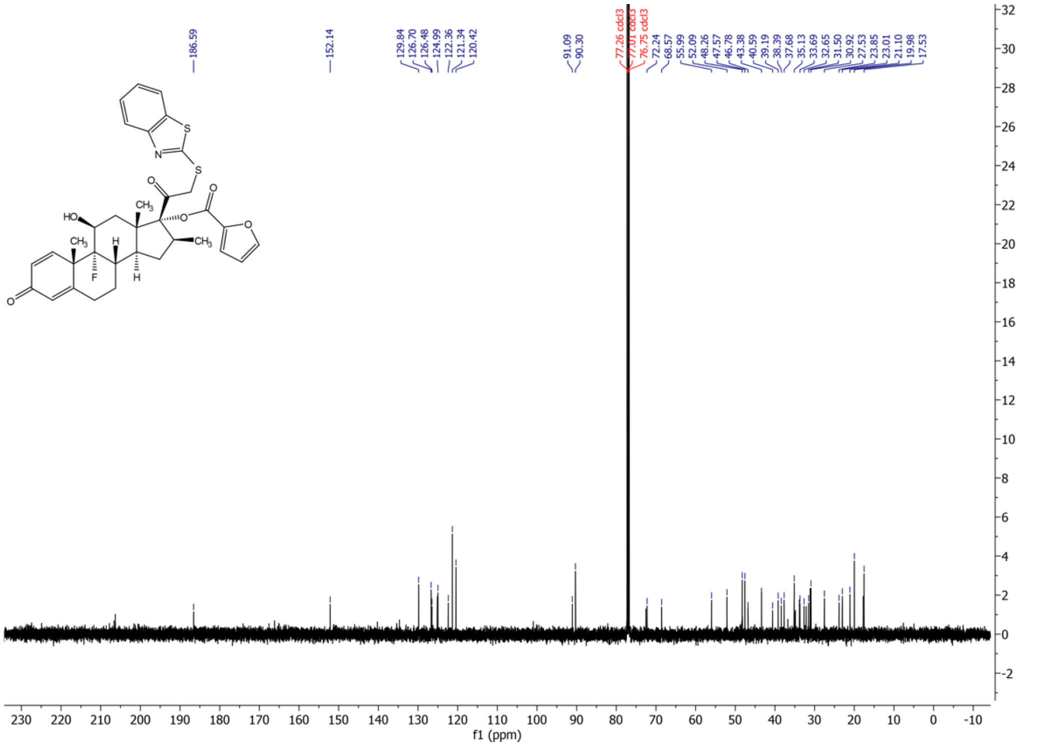

^{1}H NMR of **PN2**

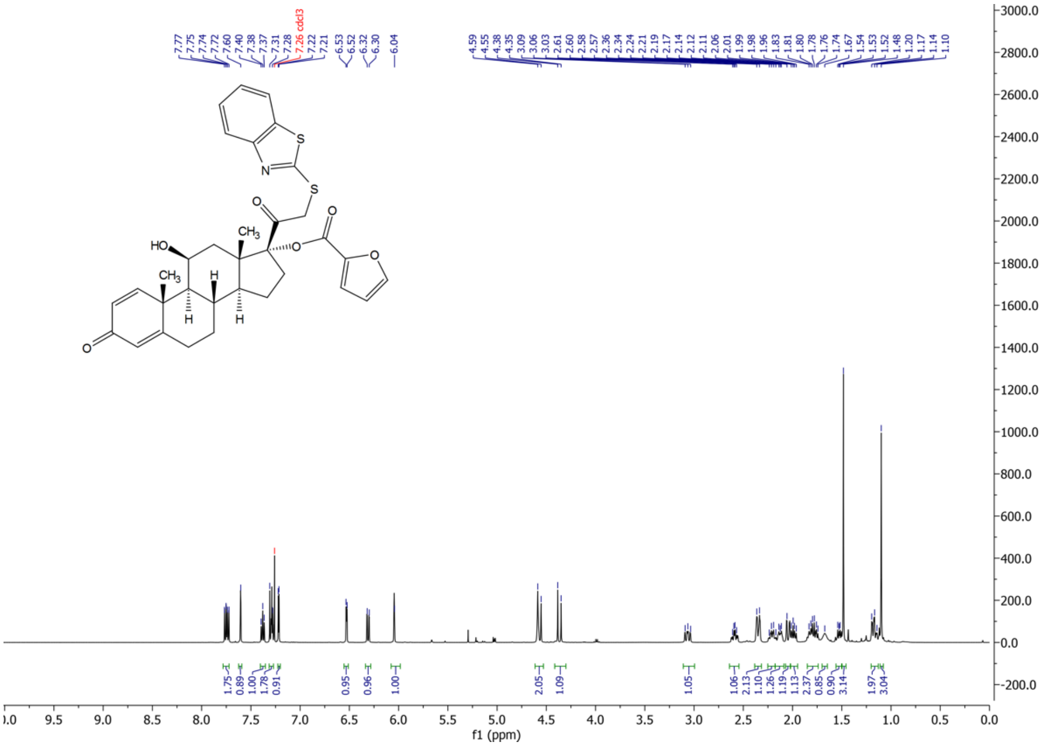

^{13}C NMR of **PN2**

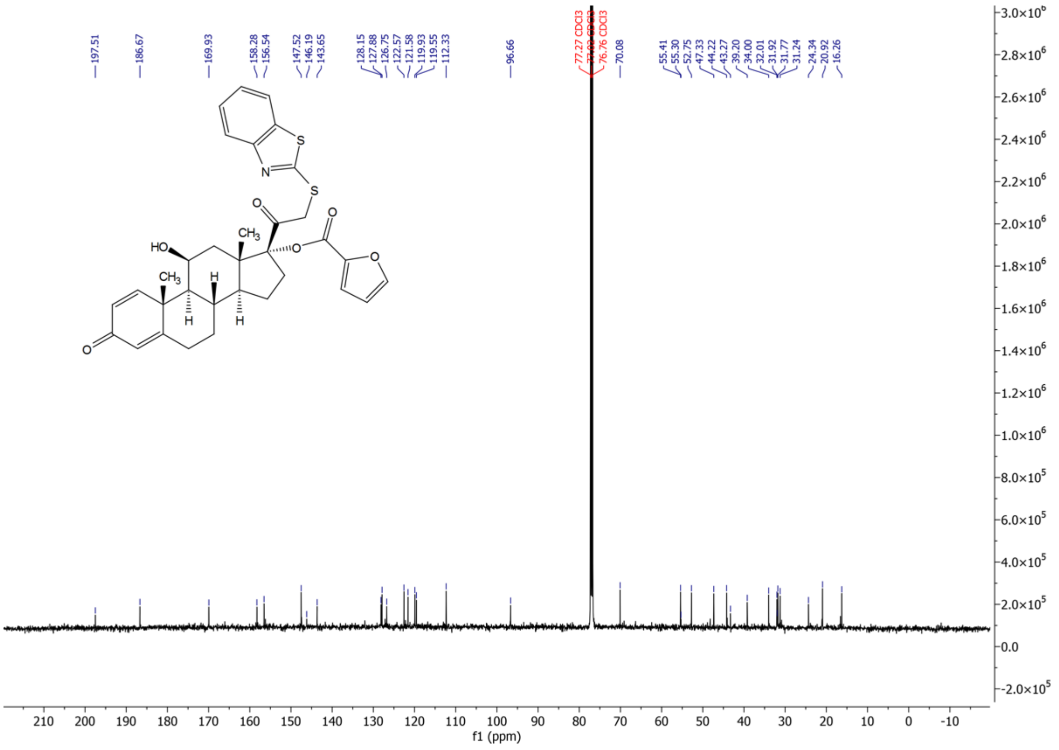

^{1}H NMR of **FM2**

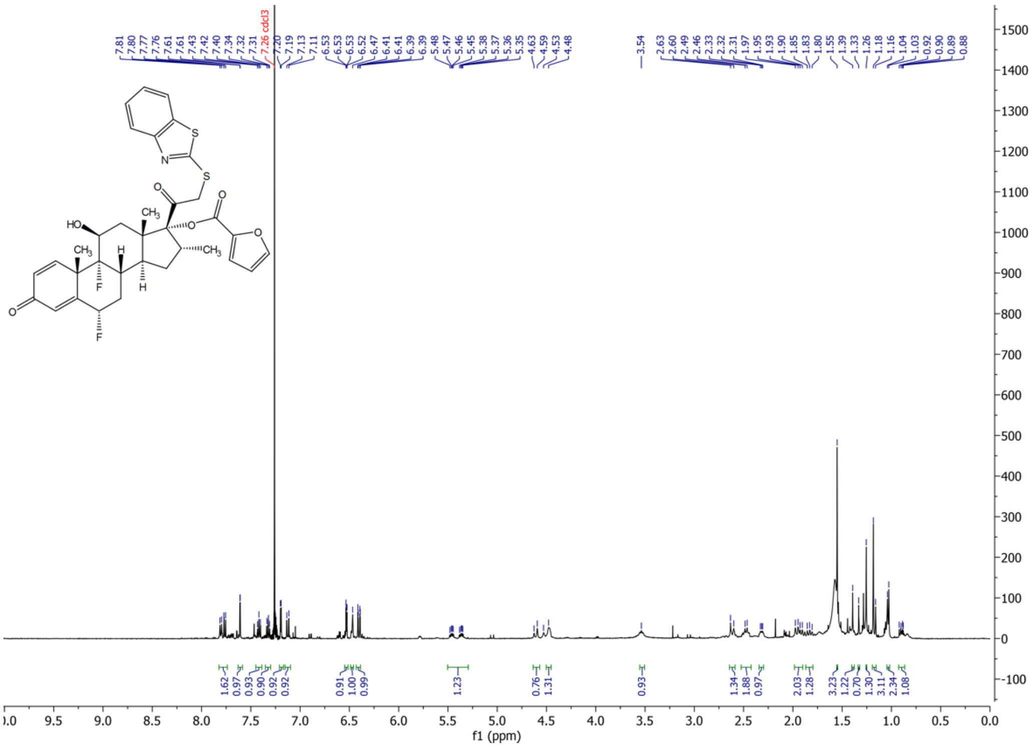

^{13}C NMR of **FM2**

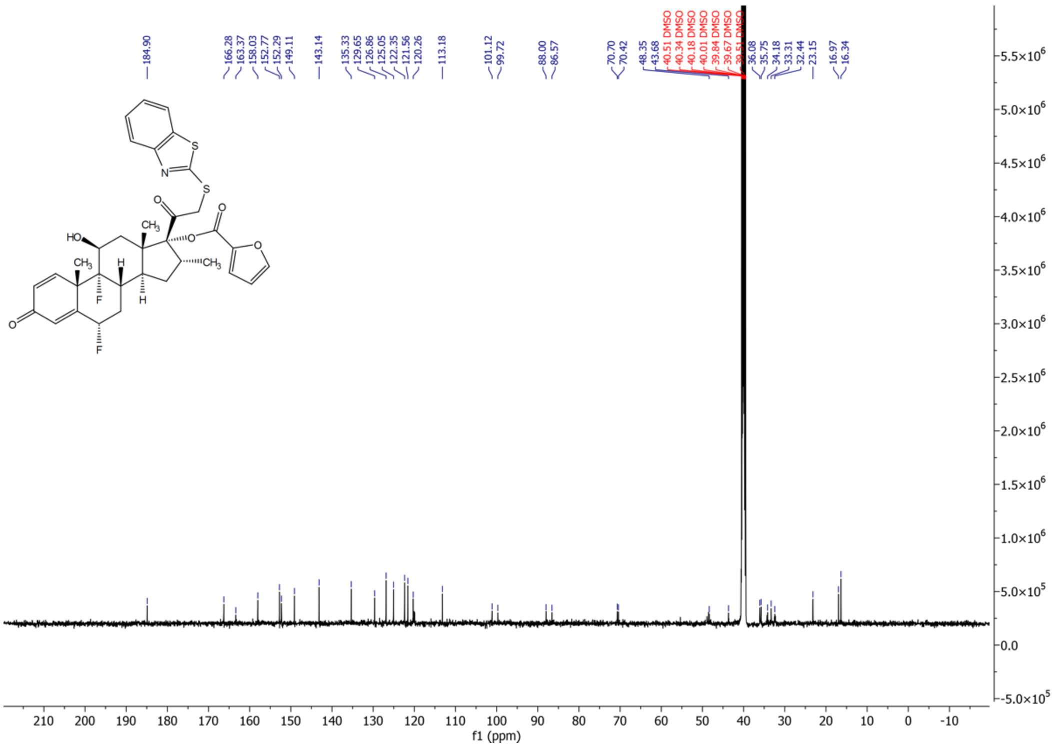

^{1}H NMR of **HC3**

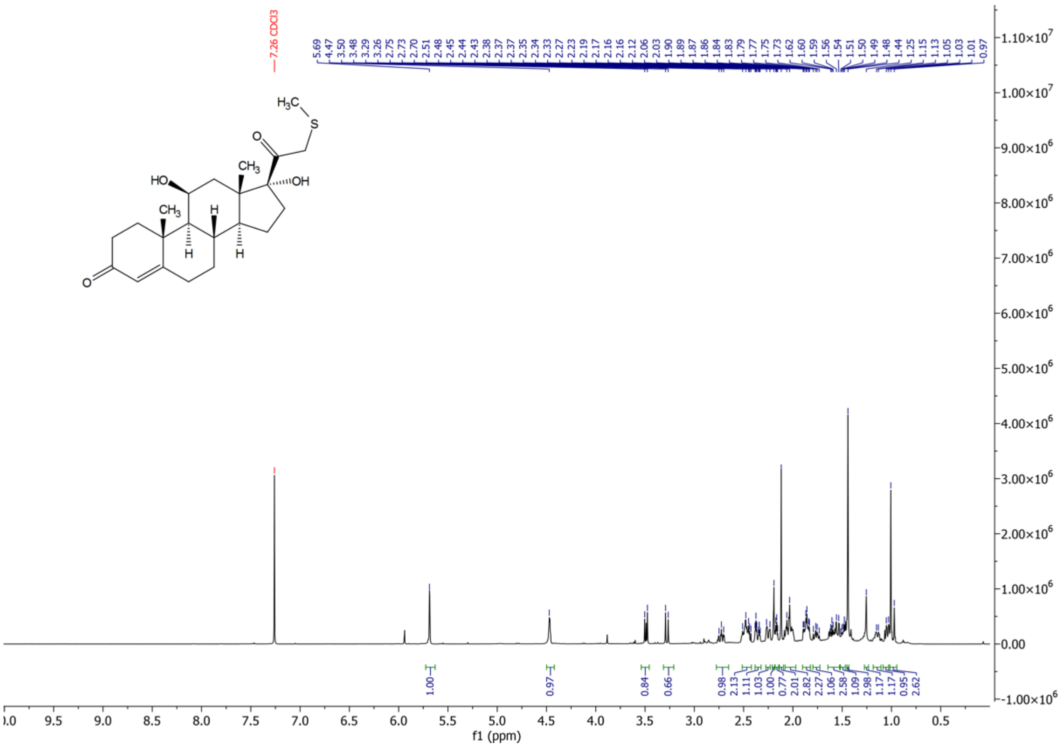

^{13}C NMR of **HC3**

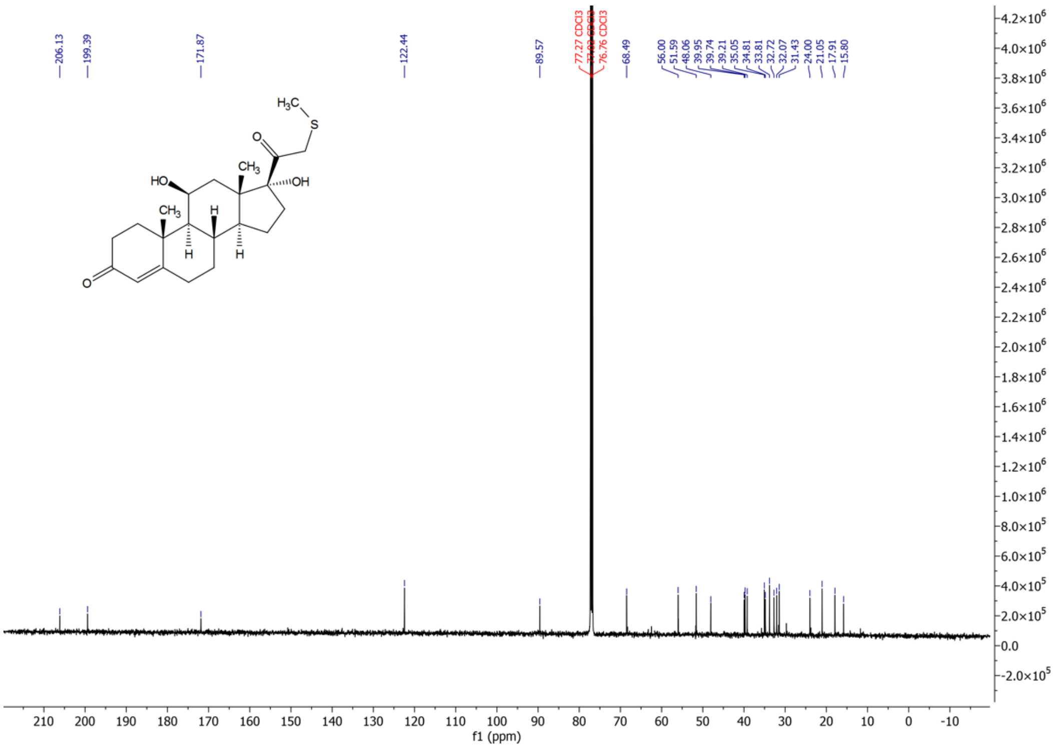

^{1}H NMR of **DX3**

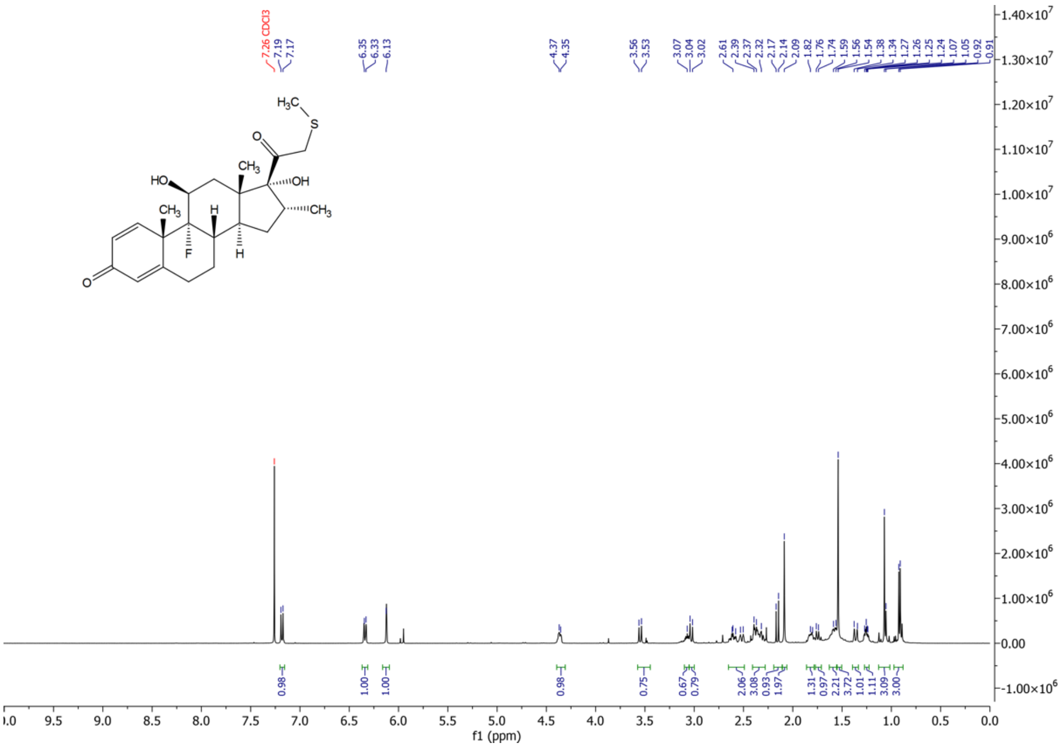

^{13}C NMR of **DX3**

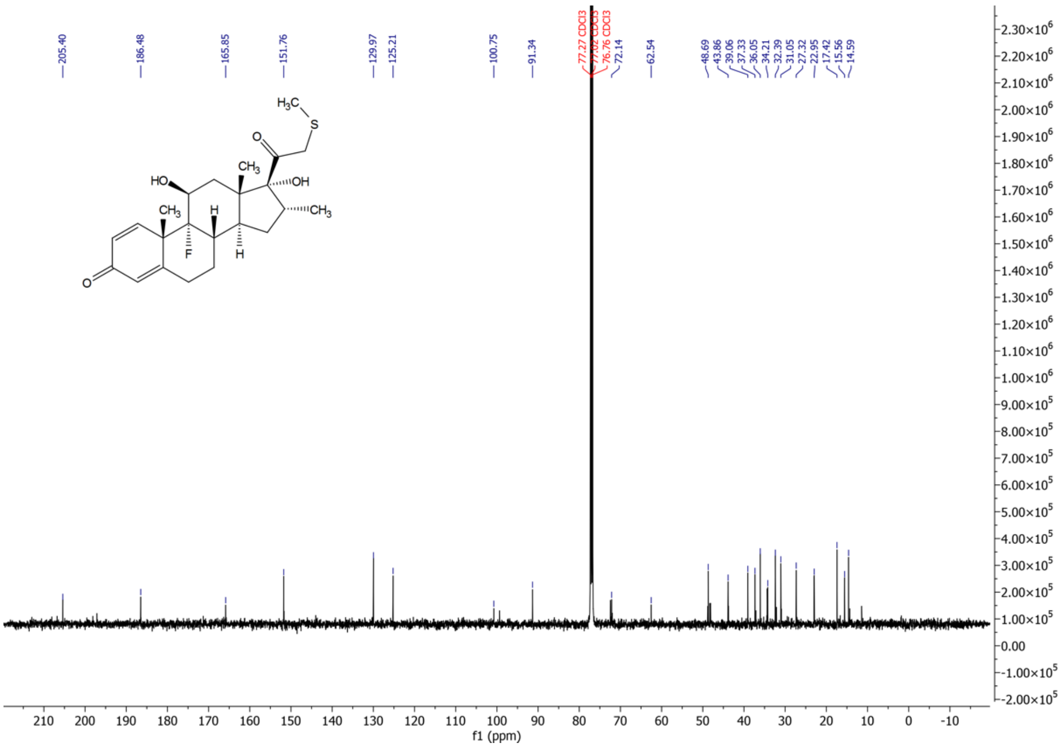

^{1}H NMR of **BM3**

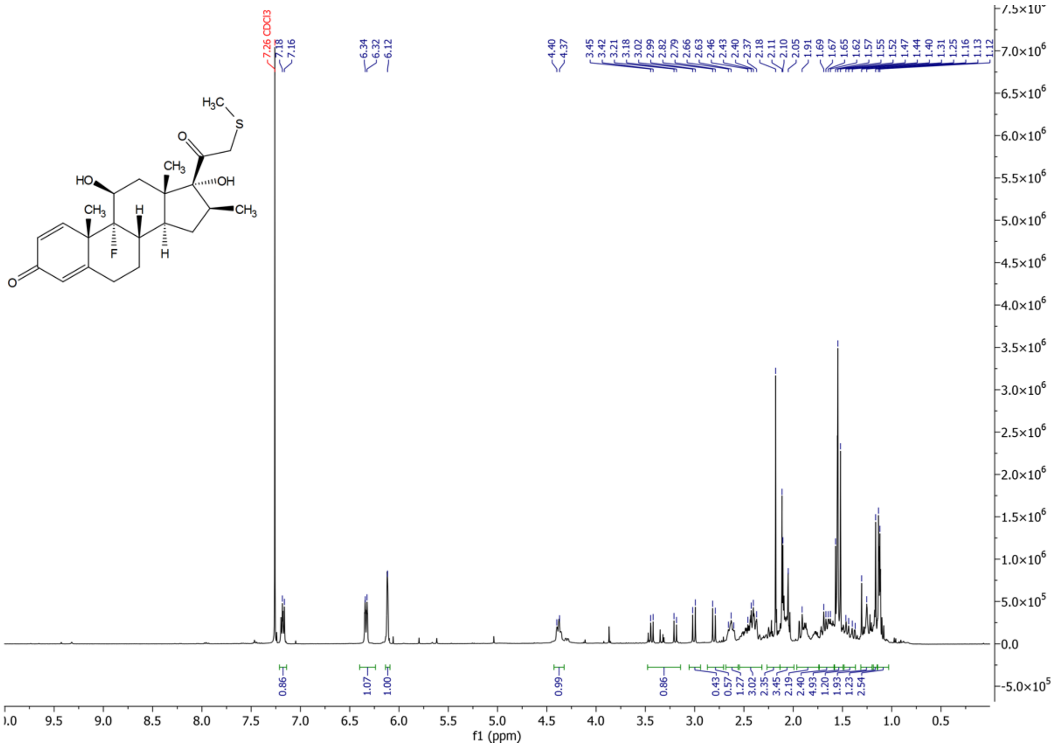

^{13}C NMR of **BM3**

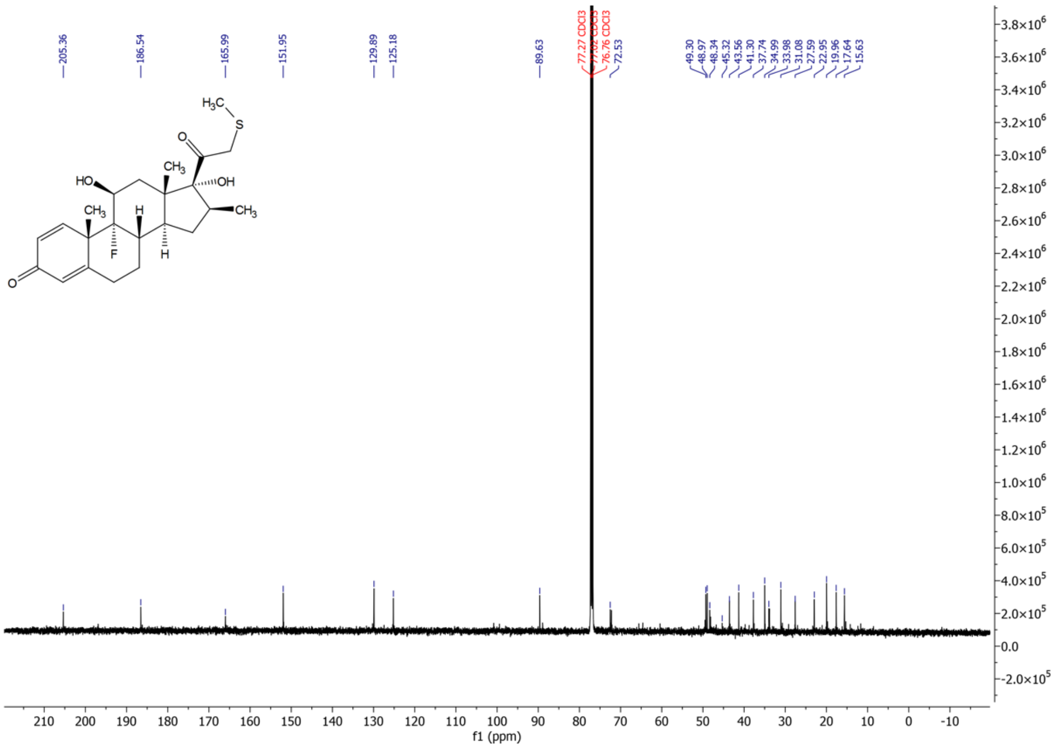

^{1}H NMR of **PN3**

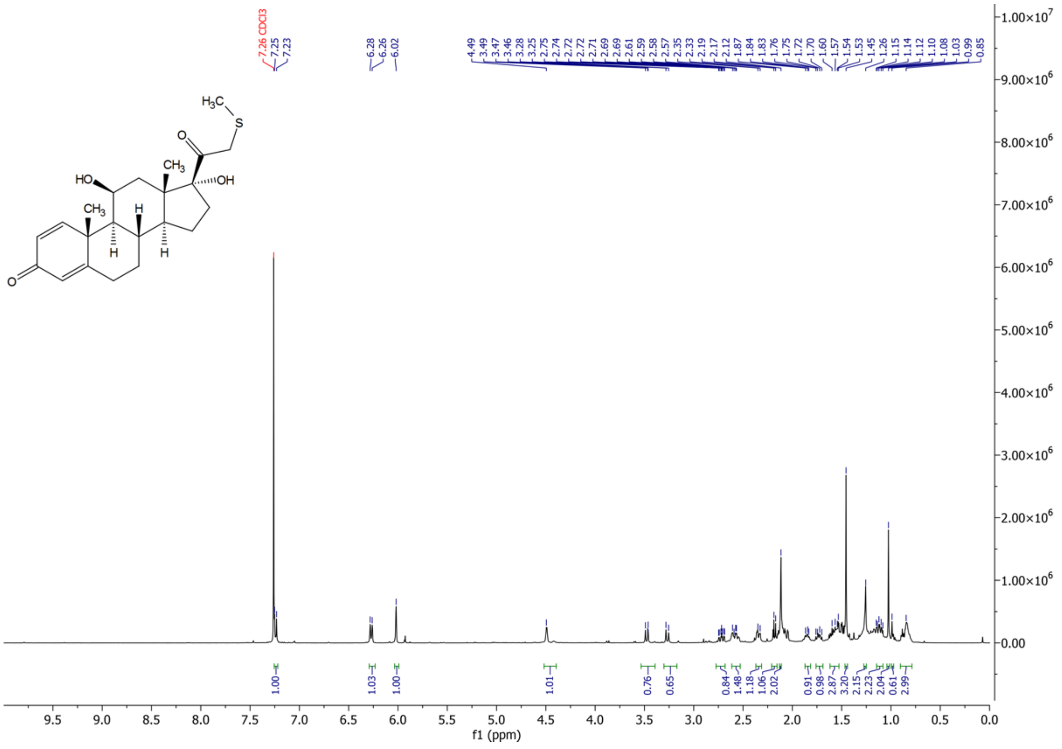

^{13}C NMR of **PN3**

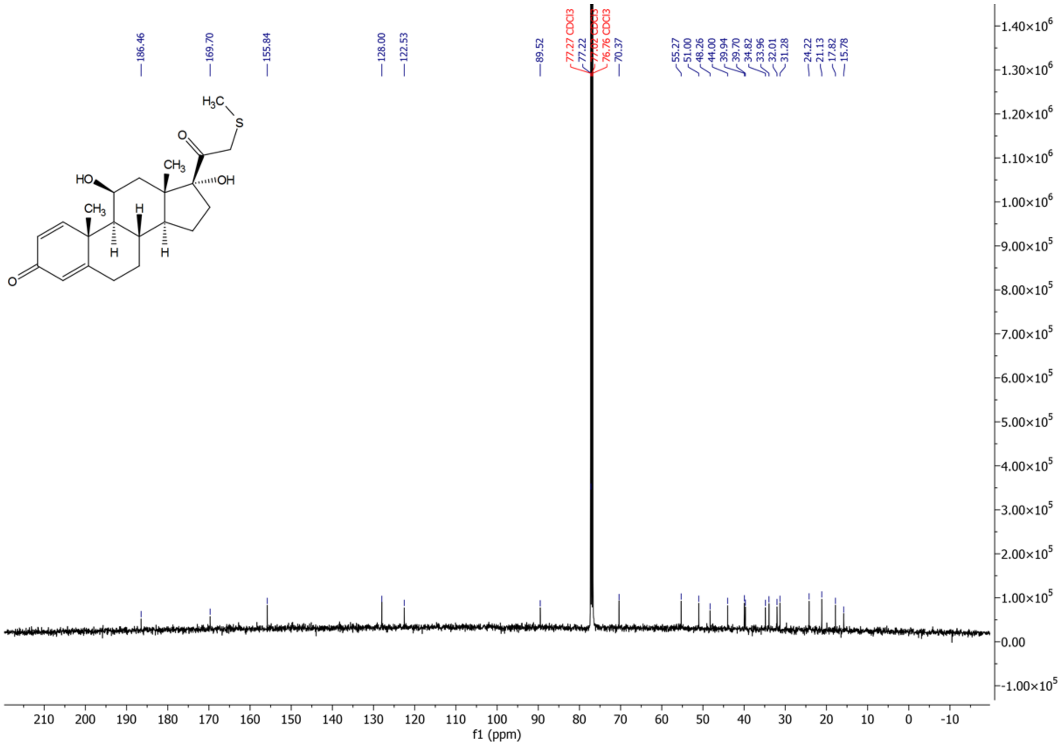

^{1}H NMR of **FM3**

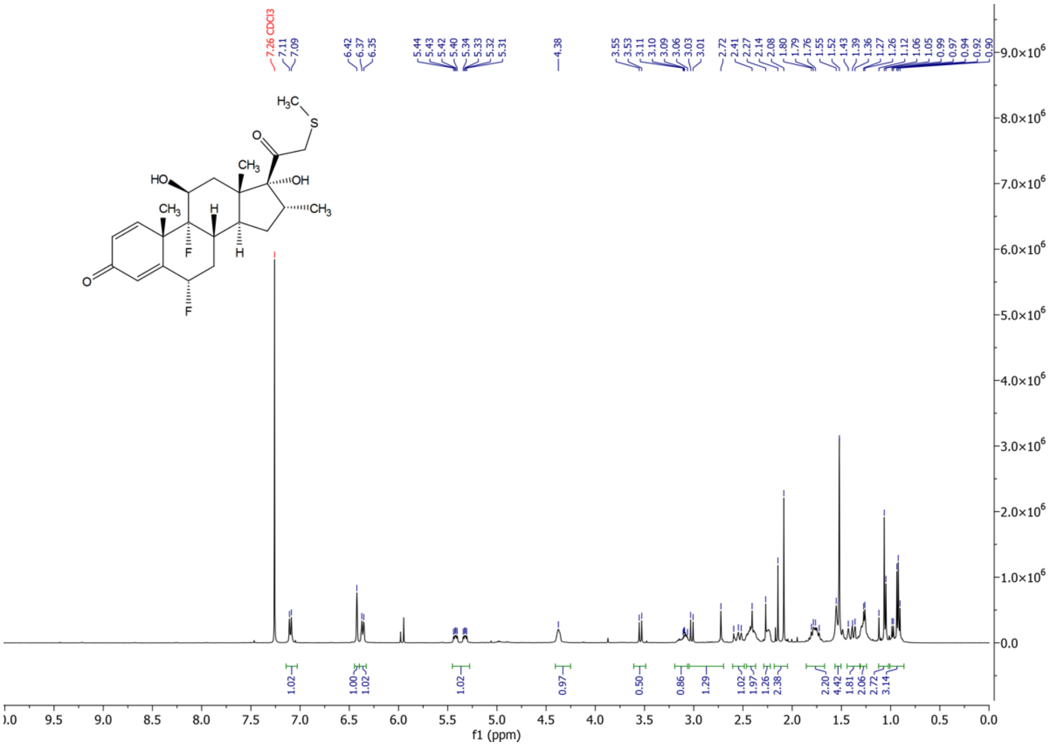

^{13}C NMR of **FM3**

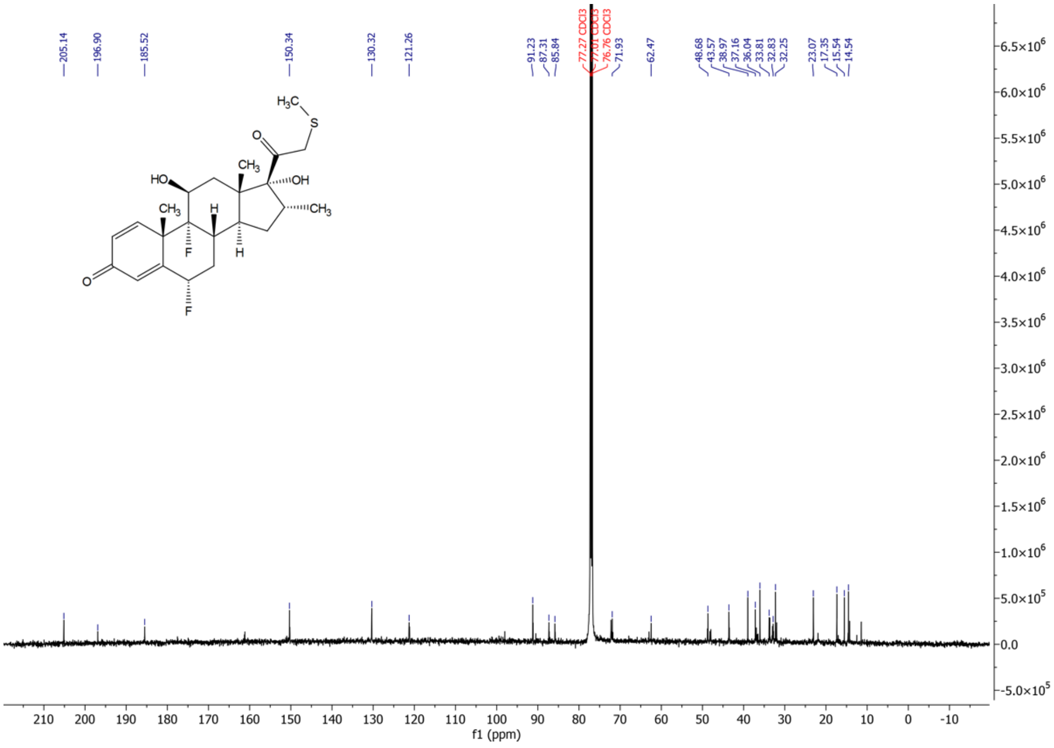

^{1}H NMR of **HC4**

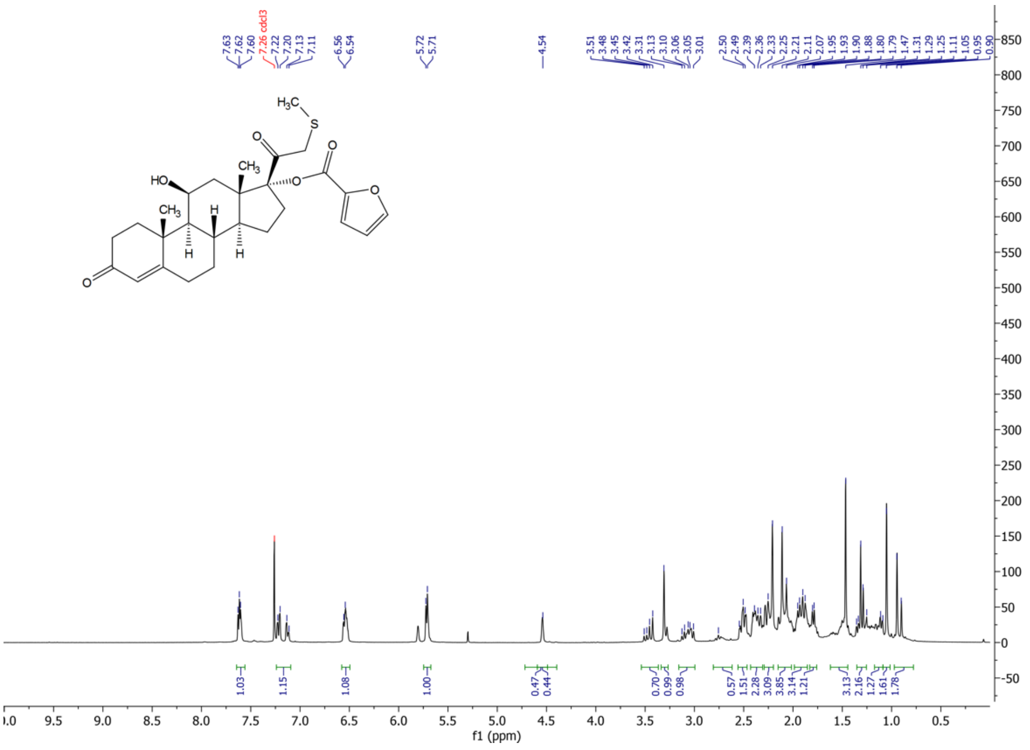

^{13}C NMR of **HC4**

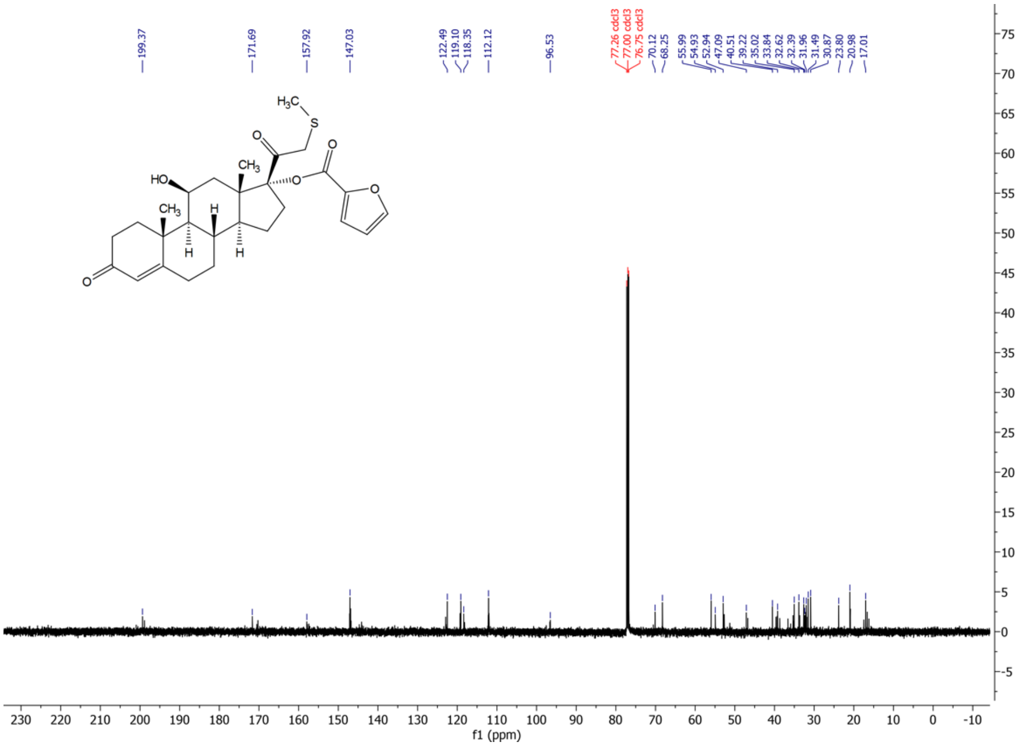

^{1}H NMR of **DX4**

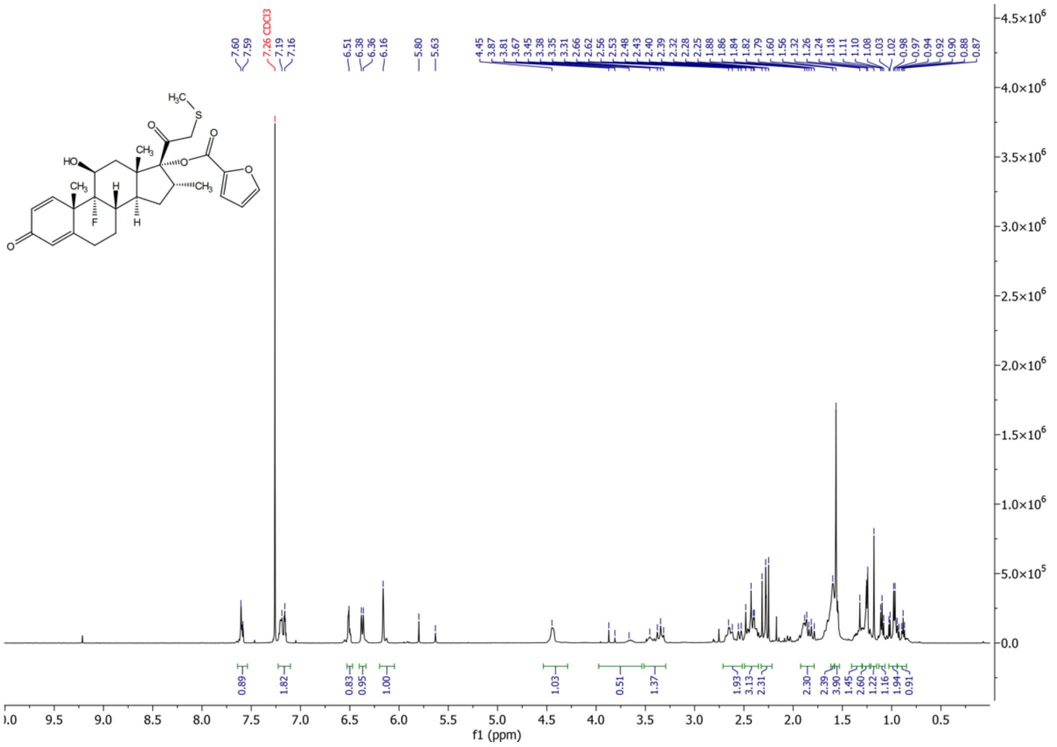

^{13}C NMR of **DX4**

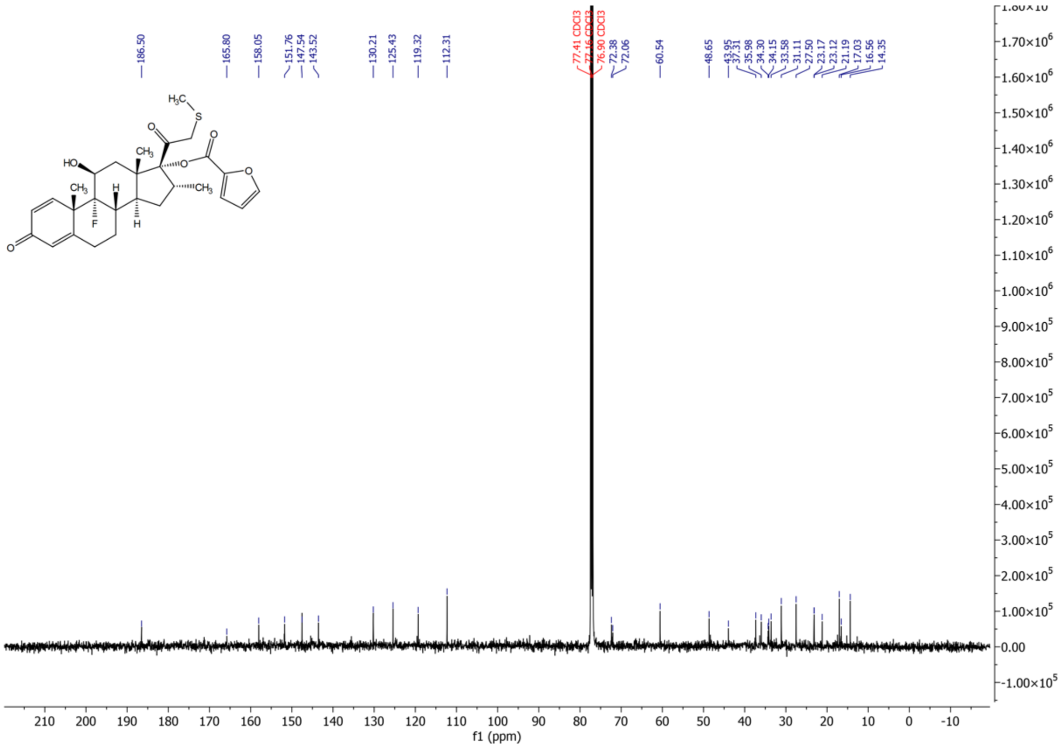

^{1}H NMR of **BM4**

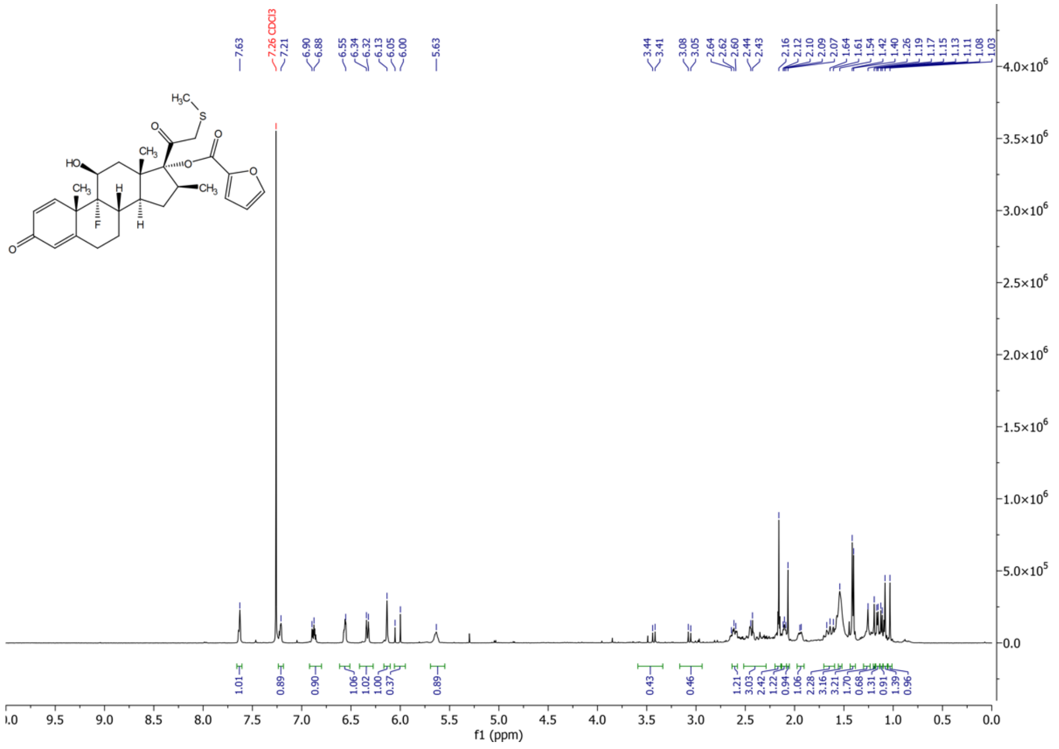

^{13}C NMR of **BM4**

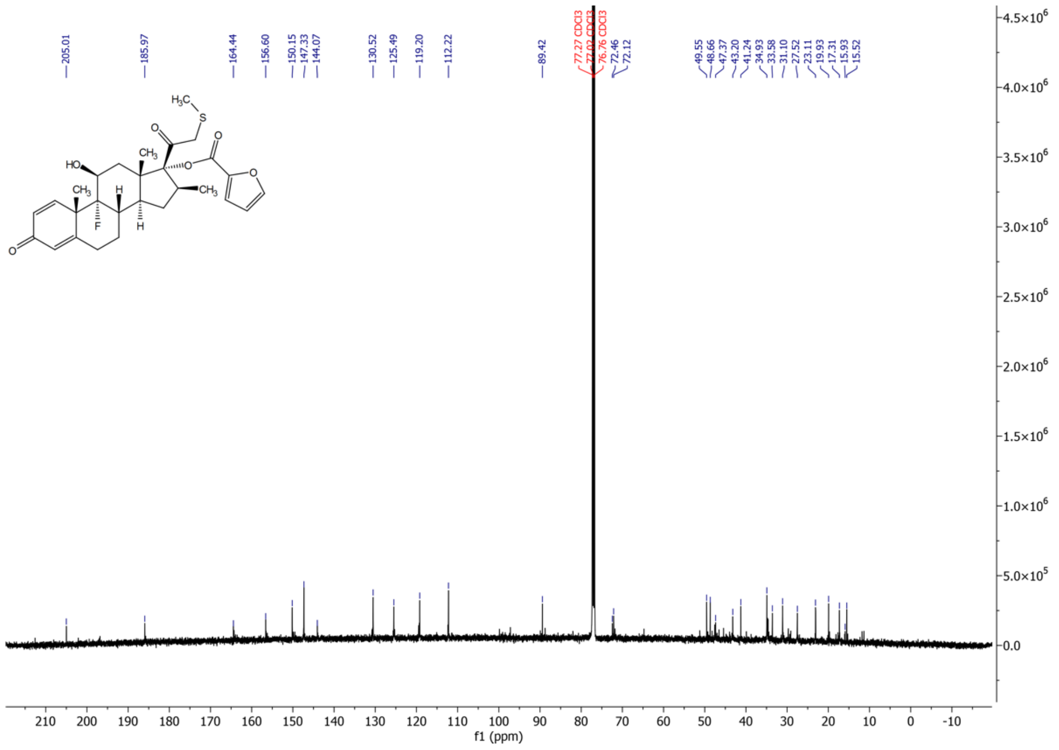

^{1}H NMR of **PN4**

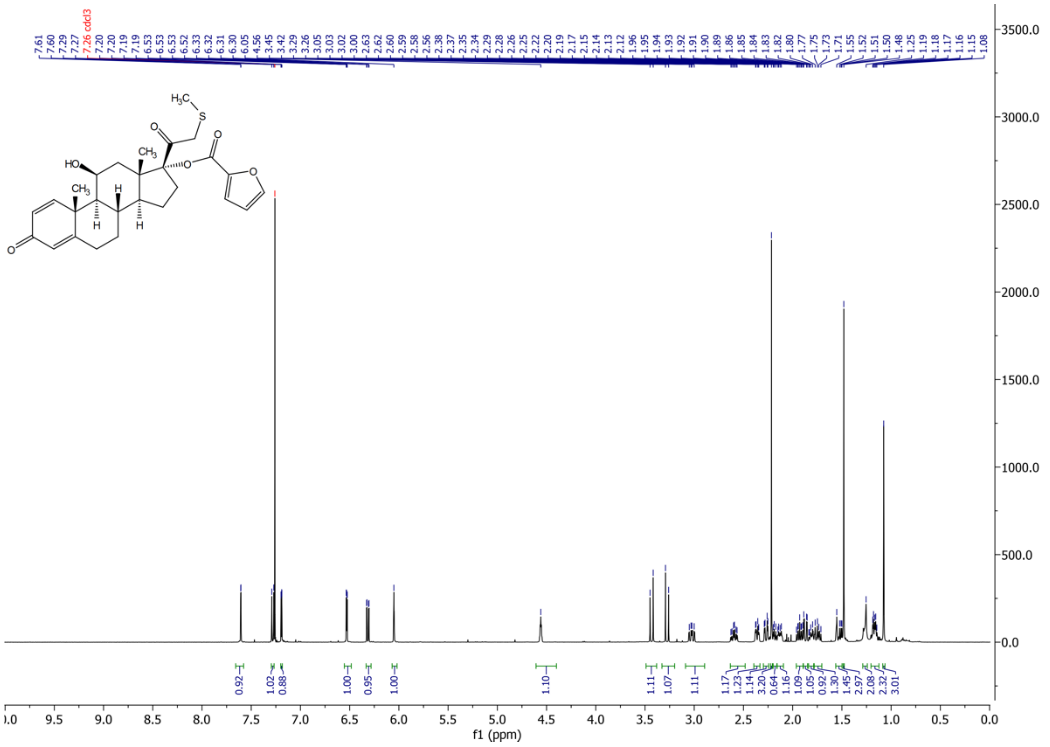

^{13}C NMR of **PN4**

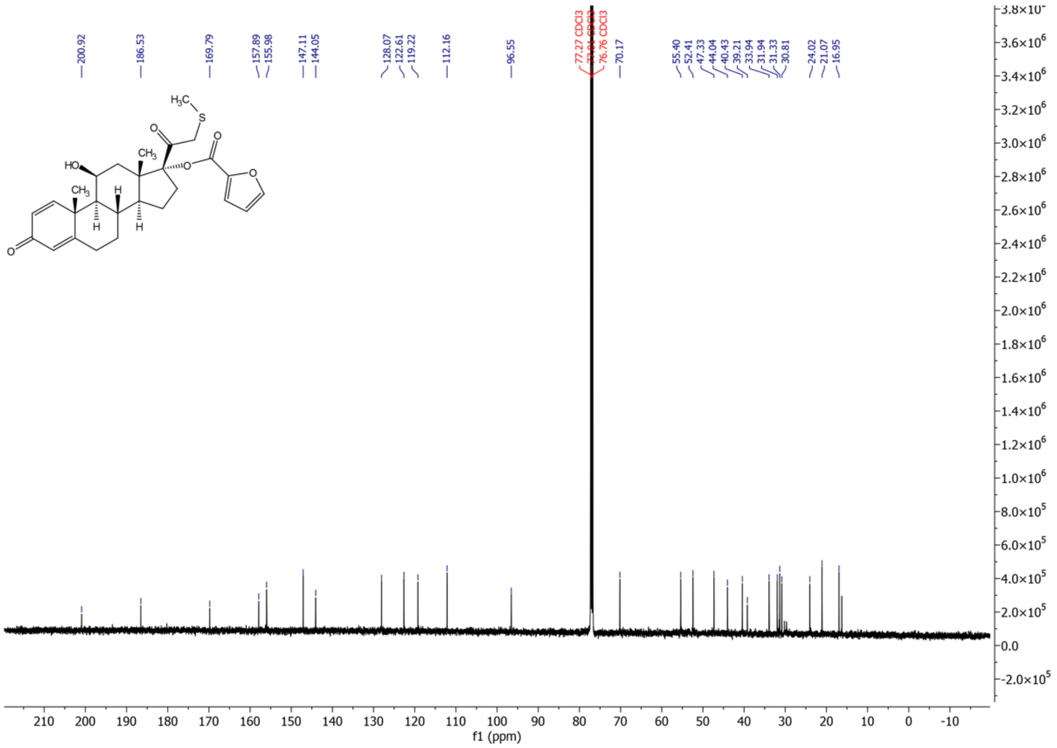

^{1}H NMR of **FM4**

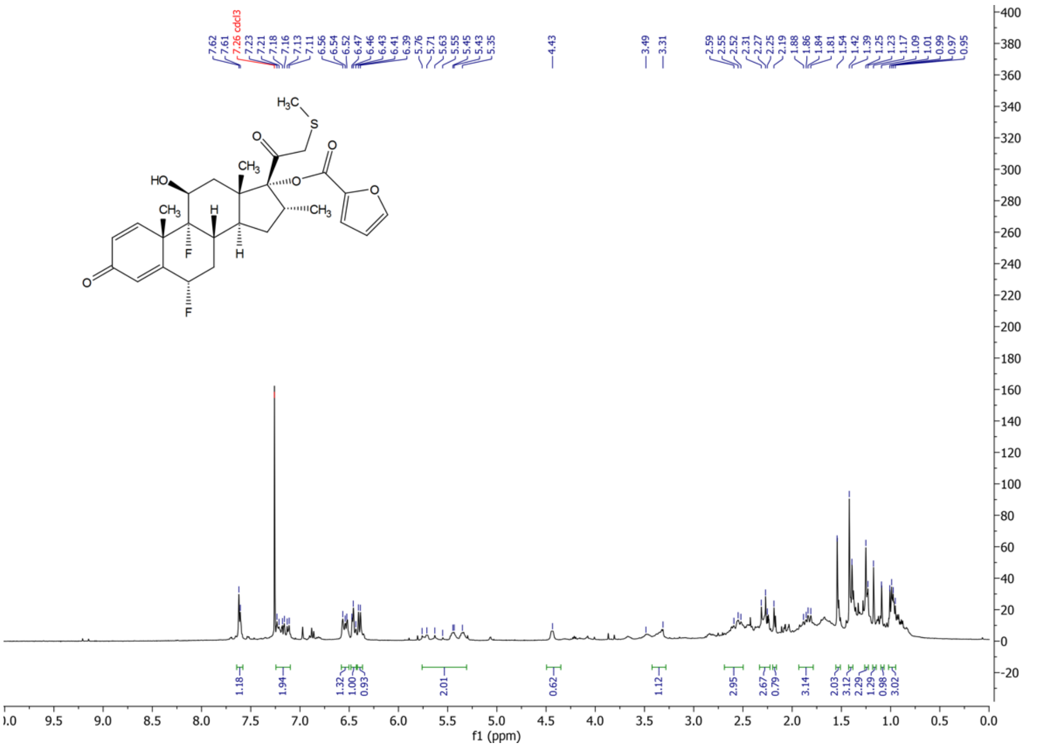

^{13}C NMR of **FM4**

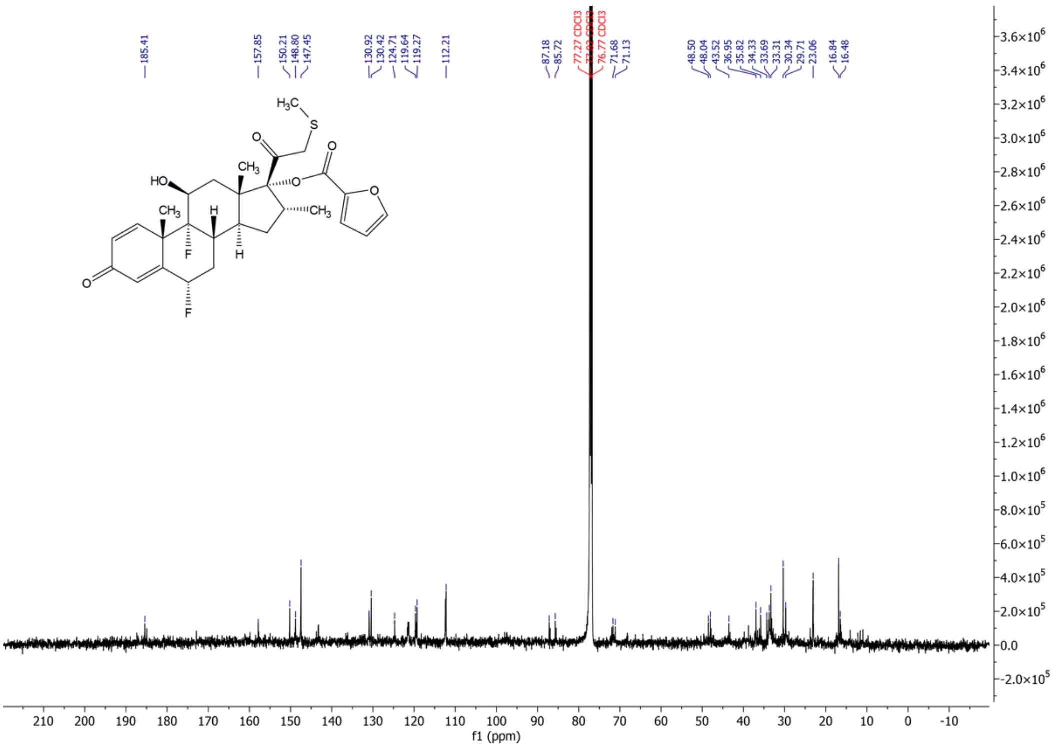

3. *LigPlot Analysis in Clustered Frame of Molecular Dynamics Simulation in 7prv*

LigPlot Legend:

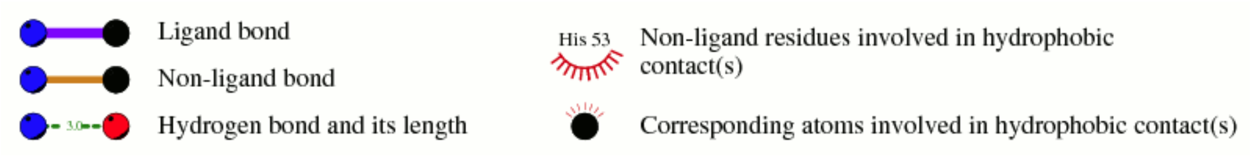

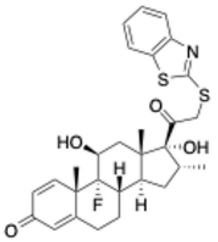

DX1

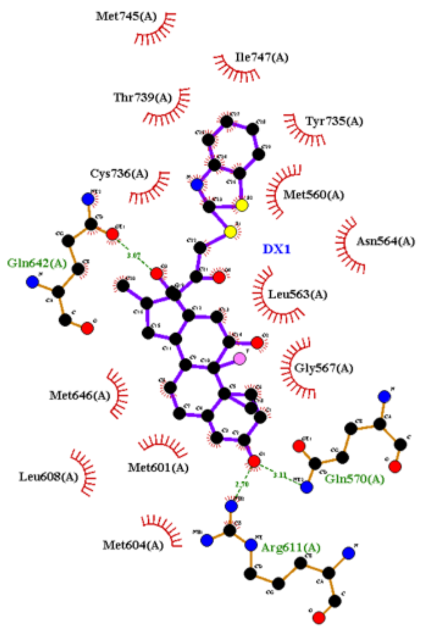

DX1

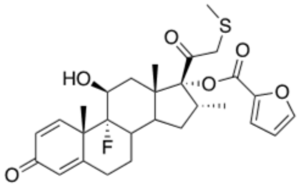

DX4

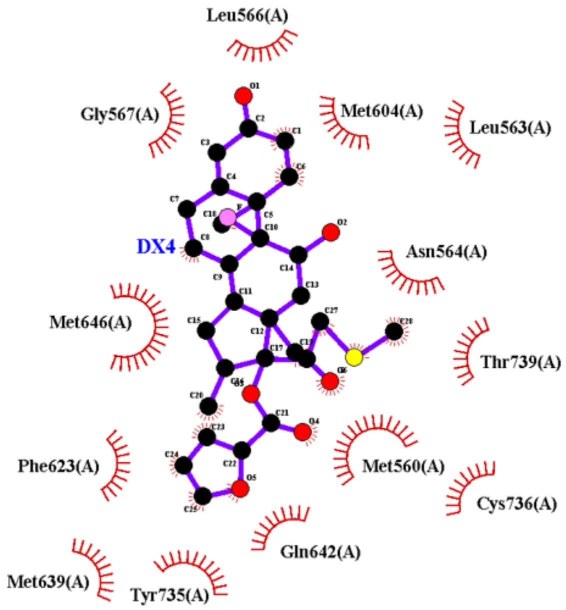

DX4

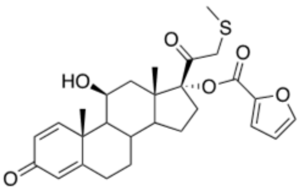

PN4

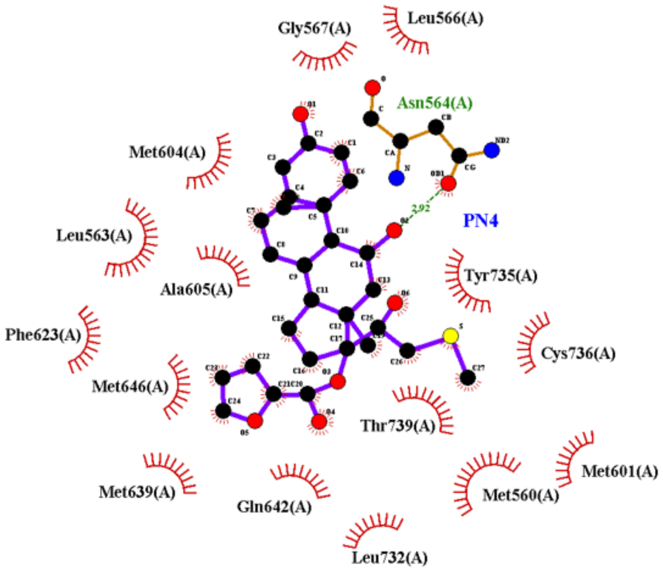

PN4

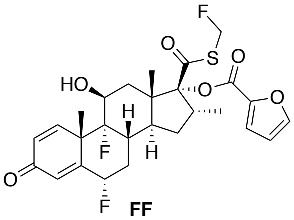

FF

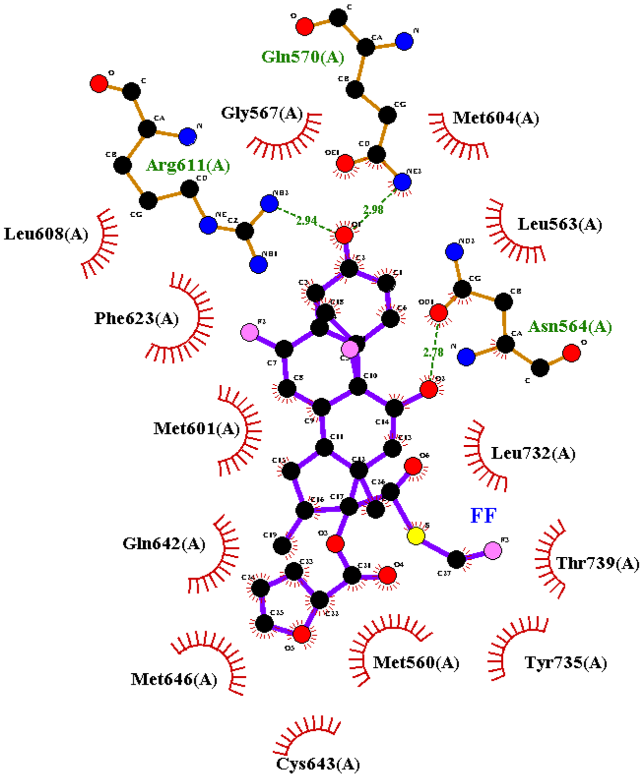

Gln570(A)
Gly567(A)
Met604(A)
Arg611(A)
Leu608(A)
Leu563(A)
Phe623(A)
Asn564(A)
Met601(A)
Leu732(A)
FF
Gln642(A)
Thr739(A)
Met646(A)
Met560(A)
Tyr735(A)
Cys643(A)
2.94
2.98
2.78

FF

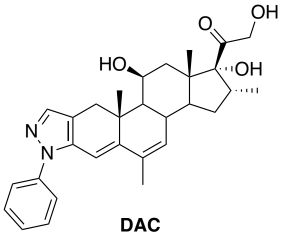

DAC

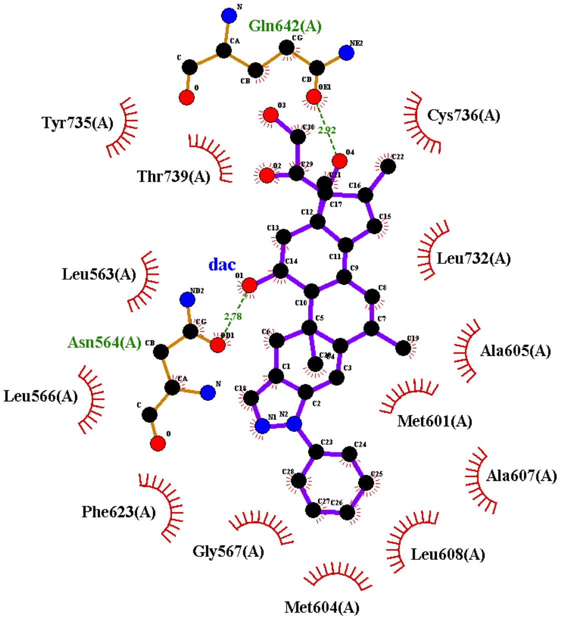

Gln642(A)
Tyr735(A)
Cys736(A)
Thr739(A)
2.92
dac
Leu563(A)
Leu732(A)
Asn564(A)
2.78
Ala605(A)
Leu566(A)
Met601(A)
Ala607(A)
Phe623(A)
Gly567(A)
Leu608(A)
Met604(A)

DAC

4. CCL2 Promoter Activity

Table S1A: CCL2-Luc Assay Raw Data

	CCL2 (%max IL-1β response)					
	Concentration of Compound (M)					
Compd	1X10^-10	1X10^-9	1X10^-8	1X10^-7	1X10^-6	1X10^-5
DX	55.4±18.6	45.7±12.2	27.7±5.42	14.8±7.80	12.5±5.80	11.4±0.33
HC3	91.0±24.8	64.3±6.32	85.7±31.6	79.8±22.1	62.9±24.6	73.0±33.1
HC4	46.2±17.8	29.4±14.5	36.6±25.6	29.1±24.7	21.4±12.3	22.6±15.9
DX1	66.6±9.61	68.7±11.8	66.5±14.1	37.7±2.06	18.9±1.51	20.2±6.05
DX2	47.6±5.19	44.2±14.7	22.8±4.35	17.1±3.01	9.63±4.06	10.3±1.69
DX3	34.0±5.53	31.6±9.95	26.7±7.58	25.4±0.94	24.9±4.02	26.3±2.99
DX4	19.3±4.10	16.9±0.26	17.1±2.70	16.2±2.96	15.4±2.46	15.3±1.26
DX5	63.6±22.7	65.3±13.8	61.2±6.29	69.7±13.4	36.8±1.16	32.9±10.1
DX6	64.9±16.8	65.8±18.8	60.5±22.3	49.6±23.9	35.0±19.8	35.4±15.0
DX7	78.4±18.9	70.6±12.5	70.7±19.7	52.0±12.3	34.2±14.1	33.3±13.4
DX8	76.0±22.8	70.6±14.5	66.4±15.8	64.2±9.83	56.2±17.3	46.1±6.61
BM	57.6±10.9	38.5±6.82	31.6±8.18	17.0±4.17	11.3±4.13	14.2±8.11
BM1	72.1±10.2	75.2±13.4	68.2±14.6	39.4±4.25	27.9±5.53	14.3±2.49
BM2	54.0±12.5	51.6±12.9	38.8±11.6	28.3±7.27	20.2±7.95	16.7±7.79
BM3	68.8±7.84	45.7±14.4	35.3±10.3	26.0±2.7	19.5±3.68	16.5±3.31
BM4	79.6±11.2	68.8±4.14	64.1±17.4	42.8±29.8	31.3±29.7	18.4±11.5
PN	87.3±12.3	62.0±15.2	41.8±16.7	20.4±7.90	14.9±4.76	15.8±6.53
PN1	90.3±28.6	89.3±4.98	88.0±7.36	73.2±12.0	60.1±14.2	25.0±8.70
PN2	41.5±5.04	36.9±7.79	18.0±3.13	13.9±3.48	12.2±3.48	9.10±1.18
PN3	98.9±26.9	93.8±24.8	87.7±20.9	72.4±20.7	56.9±12.4	50.4±25.9
PN4	26.0±4.80	25.1±9.12	25.1±6.19	21.4±4.01	19.6±5.70	19.2±5.70
FM	31.7±4.90	23.5±10.6	15.3±7.26	11.9±5.78	9.58±2.32	12.2±5.56
FM1	48.1±11.8	51.1±13.2	39.8±17.6	26.6±7.31	19.9±7.66	14.0±4.75
FM2	31.3±18.9	26.4±2.45	22.0±4.23	16.8±4.26	14.0±4.19	11.7±4.72
FM3	37.6±9.95	26.3±4.77	22.1±3.93	18.6±3.93	18.6±5.60	17.8±4.96
FM4	29.4±8.93	30.9±17.0	27.4±16.7	32.7±22.3	29.3±13.2	24.1±13.4
DN	70.8±13.1	50.1±5.42	31.0±5.73	20.9±0.36	17.2±8.11	16.8±6.86
DN1	95.2±25.8	72.3±11.0	66.5±5.57	44.0±5.76	34.2±8.95	34.4±6.81
BD	20.0±6.86	19.9±4.39	18.9±3.24	17.1±2.01	17.4±3.76	16.6±1.60
BD1	99.3±19.4	86.9±15.1	94.4±6.42	77.9±19.8	53.3±8.33	42.4±3.73

Table S1B: Low-Dose CCL2-Luc Assay Raw Data

	CCL2 (%max IL-1β response)					
	Concentration of Compound (M)					
Compd	1X10^-18	1X10^-15	1X10^-12	1X10^-9	1X10^-6	1X10^-5
DX	70.5±21.8	51.8±13.4	53.8±25.8	31.3±15.8	16.1±4.16	18.0±4.58
DX4	17.8±3.91	17.4±5.33	17.1±4.05	16.7±2.93	17.6±3.83	18.8±4.03
PN	65.9±15.3	65.3±15.6	69.5±16.8	51.2±17.1	20.3±5.93	26.1±4.53
PN4	31.8±13.6	32.1±11.7	30.2±15.4	25.6±5.59	21.9±4.58	23.9±5.64

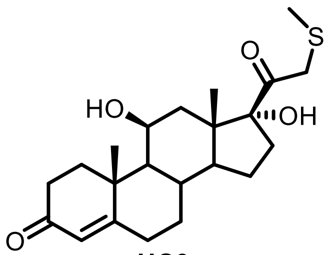

HC3

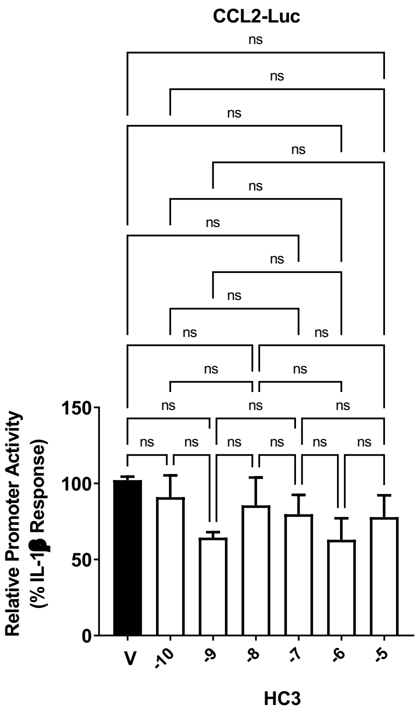

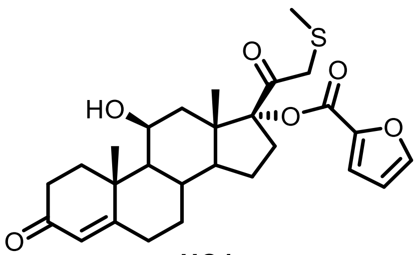

HC4

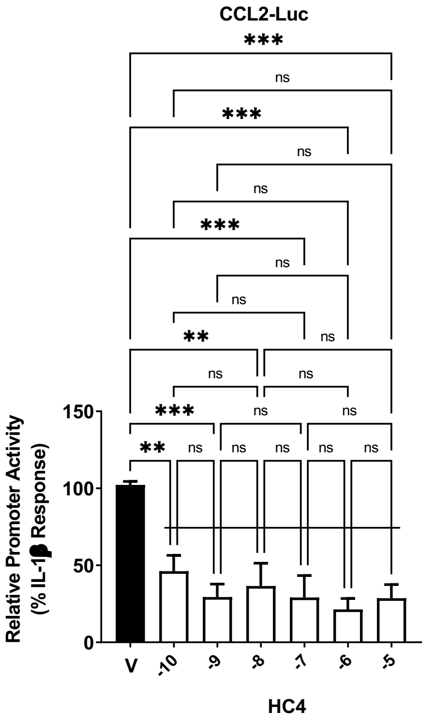
CCL2-Luc
Relative Promoter Activity (% IL-1β Response)
150
100
50
0
V
-10
-9
-8
-7
-6
-5
HC4
***
**
ns

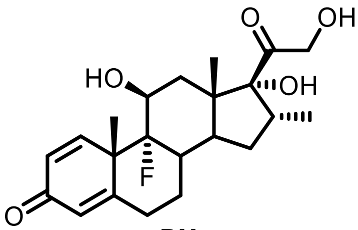

DX

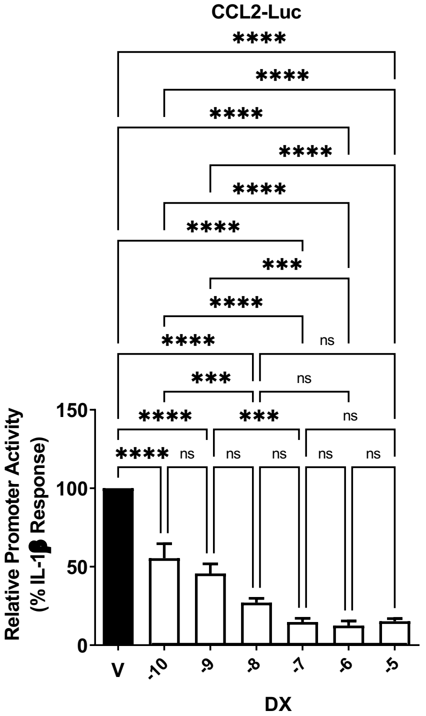
CCL2-Luc
Relative Promoter Activity (% IL-1β Response)
150
100
50
0
V
-10
-9
-8
-7
-6
-5
DX
****
***
ns

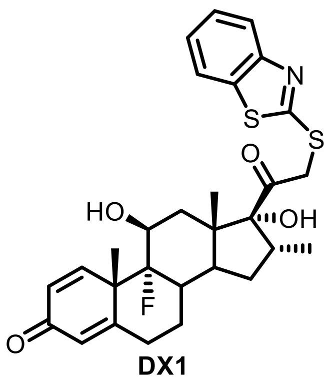

DX1

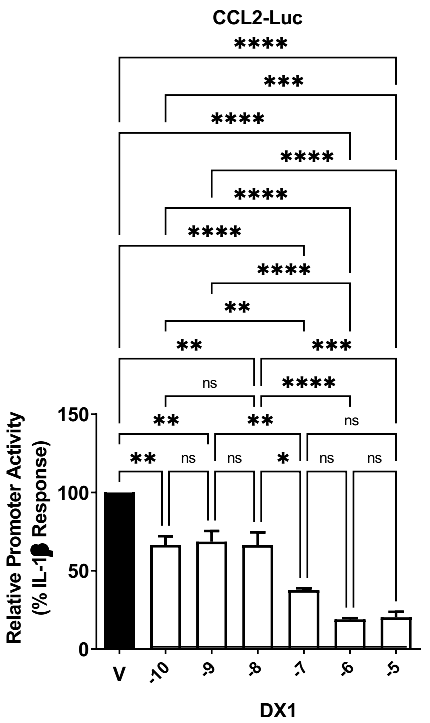
CCL2-Luc
Relative Promoter Activity
(% IL-1β Response)
V
-10
-9
-8
-7
-6
-5
DX1

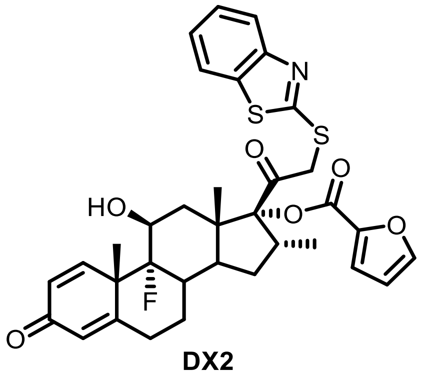

DX2

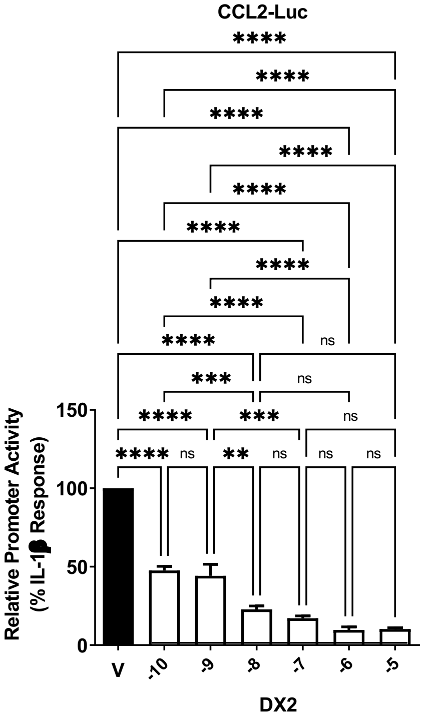
CCL2-Luc
Relative Promoter Activity
(% IL-1β Response)
V
-10
-9
-8
-7
-6
-5
DX2

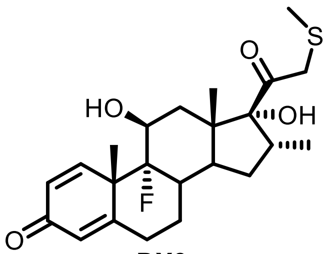

DX3

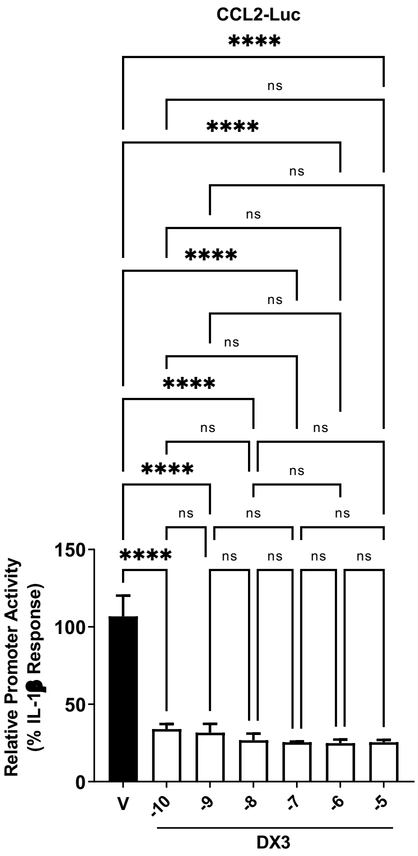
CCL2-Luc
Relative Promoter Activity
(% IL-1β Response)
150
100
50
0
V
-10
-9
-8
-7
-6
-5
DX3
****
ns

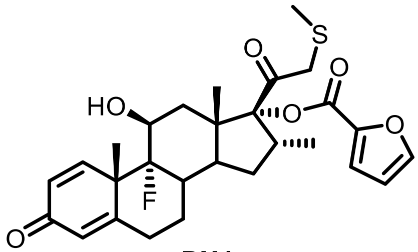
HO
O
O
S
O
O
O
F

DX4

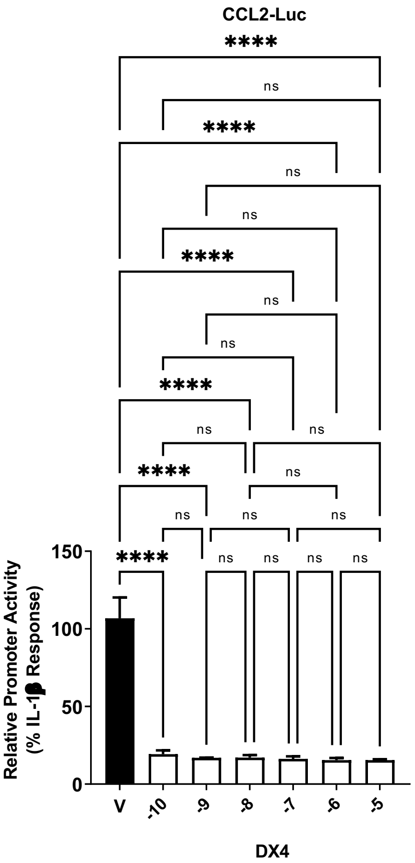
CCL2-Luc
Relative Promoter Activity
(% IL-1β Response)
V
-10
-9
-8
-7
-6
-5
DX4

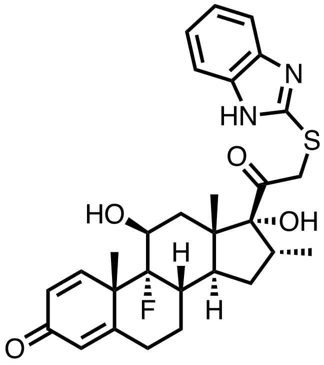
N
HN
S
O
HO
OH
H
F
H
O

DX5

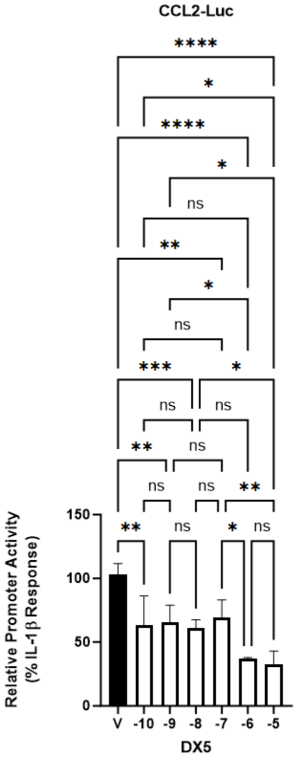
CCL2-Luc
Relative Promoter Activity
(% IL-1β Response)
V
-10
-9
-8
-7
-6
-5
DX5

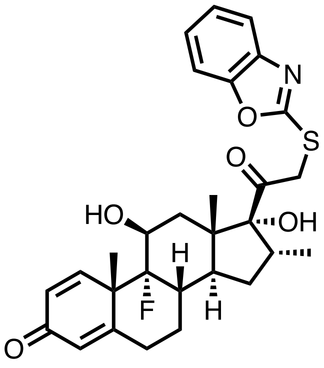
N
O
S
O
HO
OH
H
F
H
O

DX6

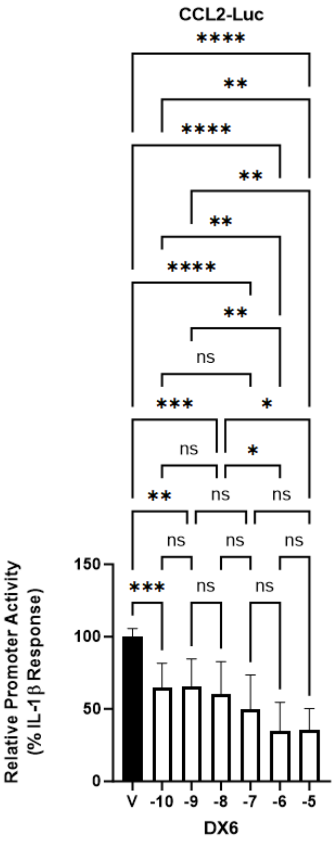
CCL2-Luc
Relative Promoter Activity
(% IL-1β Response)
150
100
50
0
V
-10
-9
-8
-7
-6
-5
DX6
****
**
***
*
ns

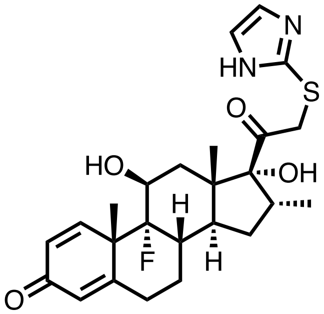
N
HN
S
O
HO
OH
H
F
H
O

DX7

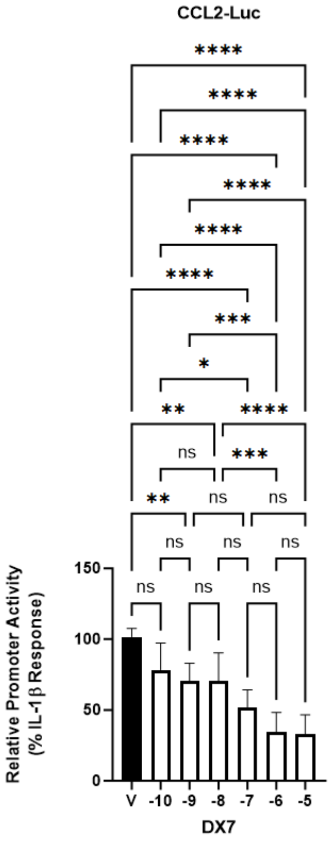
CCL2-Luc
Relative Promoter Activity
(% IL-1β Response)
150
100
50
0
V
-10
-9
-8
-7
-6
-5
DX7
****
***
**
*
ns

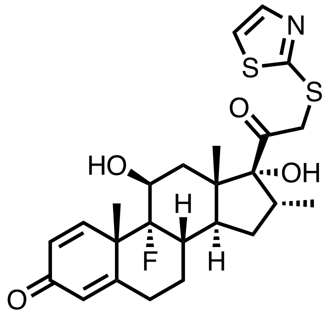
HO
OH
O
S
N
H
F

DX8

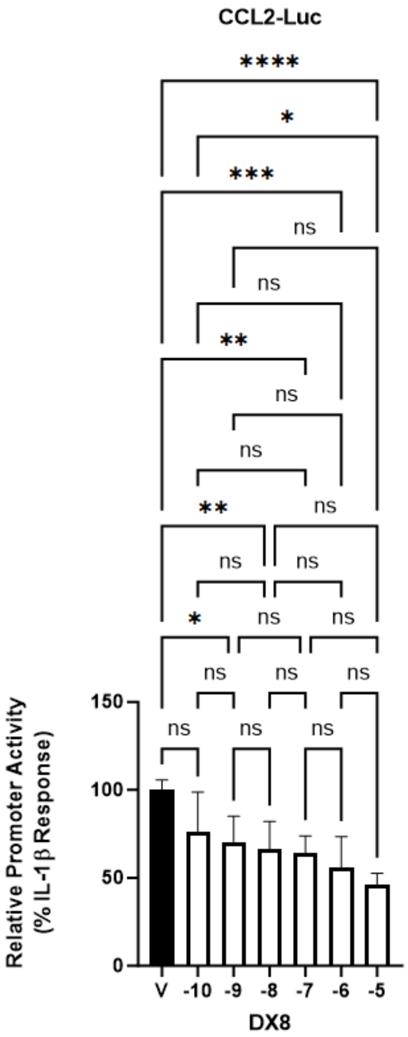
CCL2-Luc
Relative Promoter Activity
(% IL-1β Response)
150
100
50
0
V
-10
-9
-8
-7
-6
-5
DX8
****
*
***
**
ns

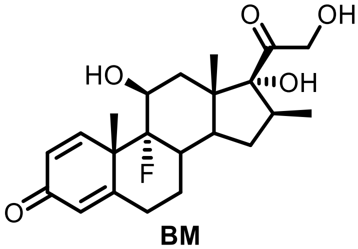
HO
OH
O
OH
F
O

BM

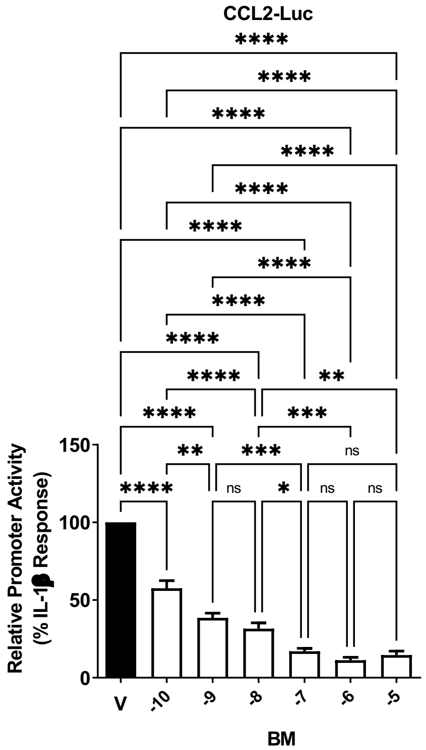
CCL2-Luc
Relative Promoter Activity
(% IL-1β Response)
150
100
50
0
V
-10
-9
-8
-7
-6
-5
BM
****
***
**
*
ns

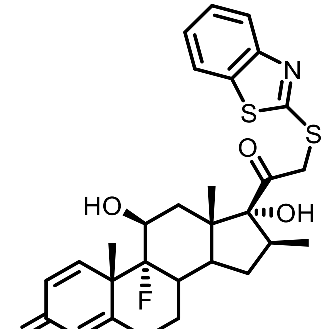
N
S
S
O
HO
OH
F
O

BM1

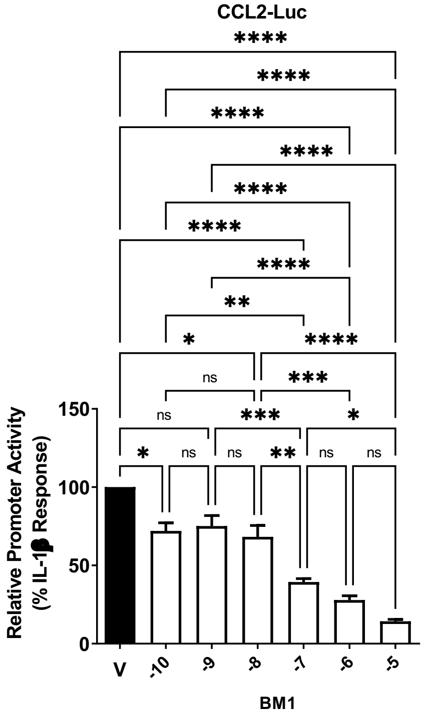
CCL2-Luc
Relative Promoter Activity
(% IL-1β Response)
150
100
50
0
V
-10
-9
-8
-7
-6
-5
BM1
****
***
**
*
ns

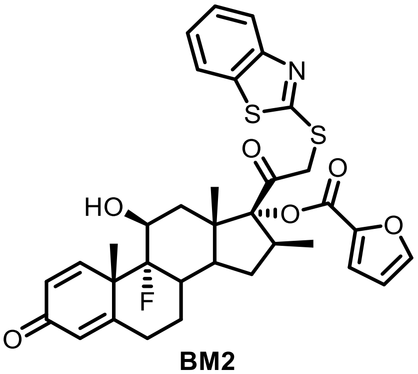

BM2

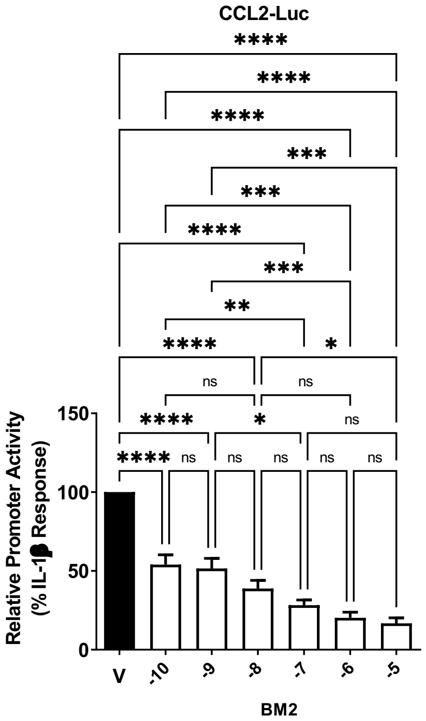
CCL2-Luc
Relative Promoter Activity
(% IL-1β Response)
150
100
50
0
V
-10
-9
-8
-7
-6
-5
BM2
****
***
**
*
ns

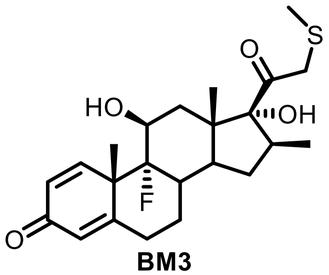

BM3

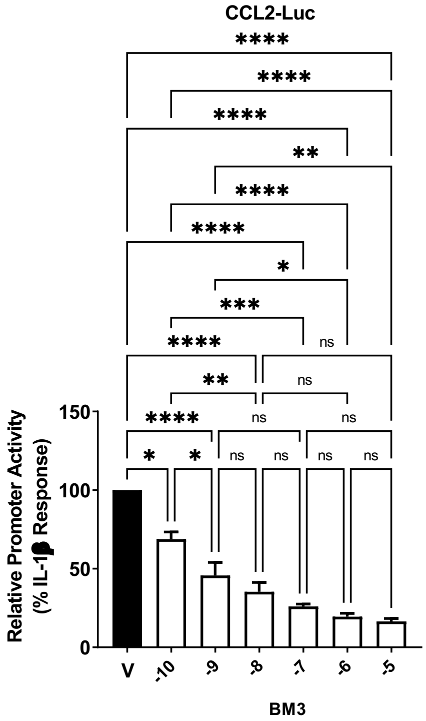
CCL2-Luc
Relative Promoter Activity
(% IL-1β Response)
150
100
50
0
V
-10
-9
-8
-7
-6
-5
BM3
****
***
**
*
ns

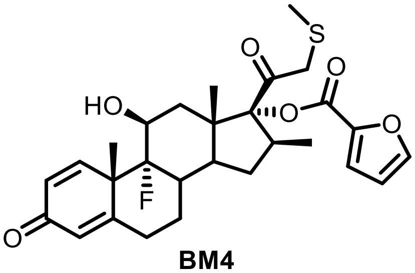

BM4

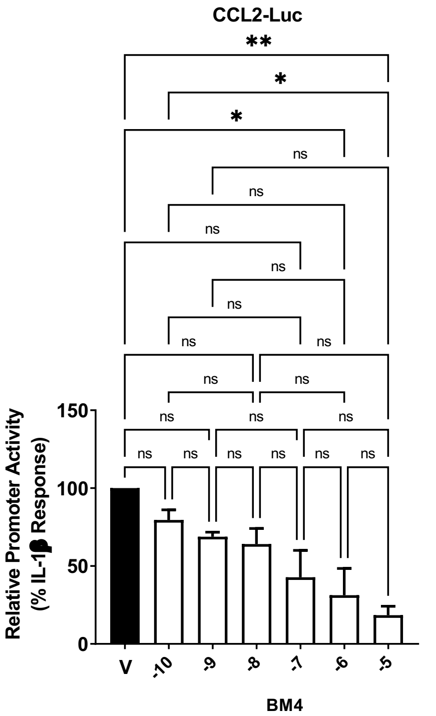
CCL2-Luc
Relative Promoter Activity
(% IL-1β Response)
150
100
50
0
V
-10
-9
-8
-7
-6
-5
BM4
**
*
*
ns

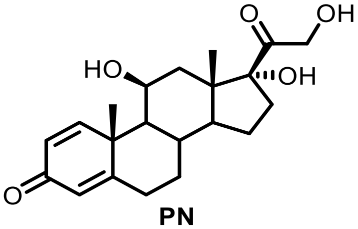

PN

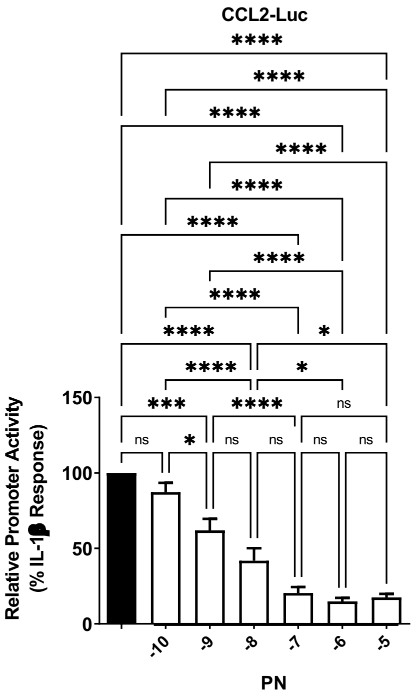
CCL2-Luc
Relative Promoter Activity
(% IL-1β Response)
150
100
50
0
-10
-9
-8
-7
-6
-5
PN
****
***
*
ns

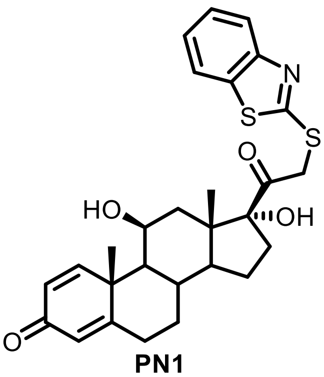

PN1

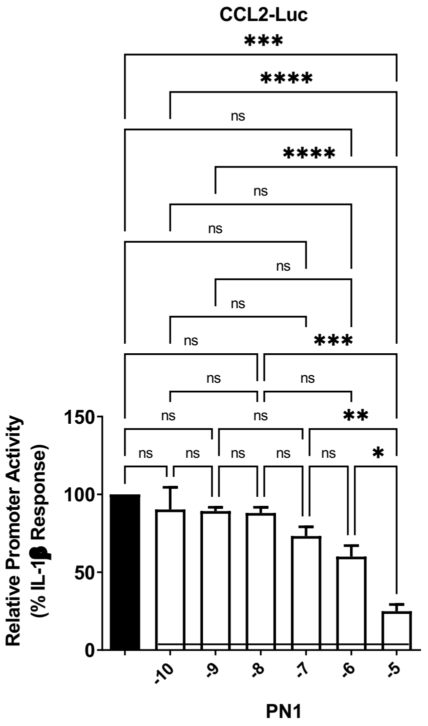

CCL2-Luc
Relative Promoter Activity
(% IL-1β Response)
150
100
50
0
-10
-9
-8
-7
-6
-5
PN1

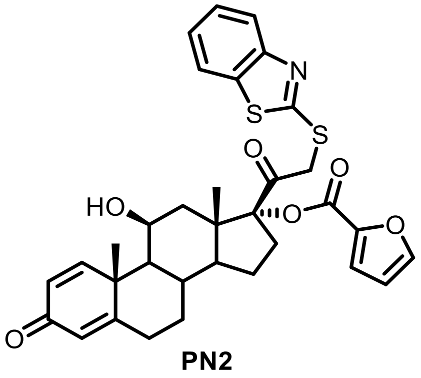

PN2

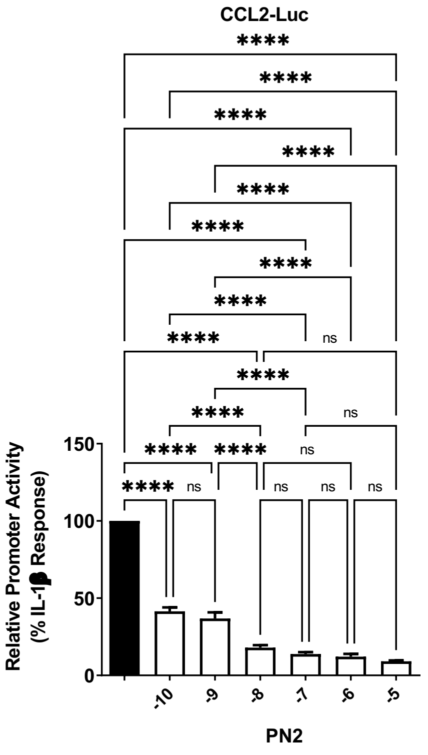

CCL2-Luc
Relative Promoter Activity
(% IL-1β Response)
150
100
50
0
-10
-9
-8
-7
-6
-5
PN2

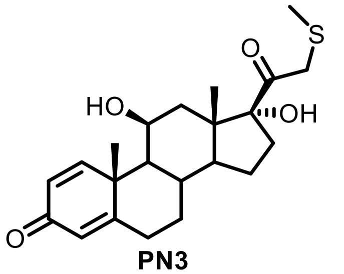

PN3

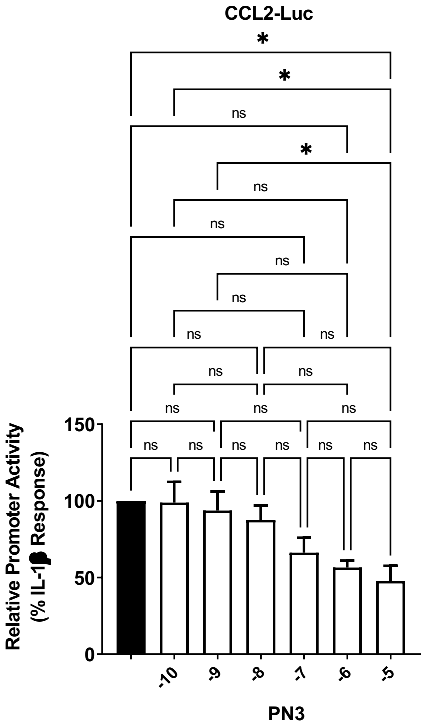
CCL2-Luc
Relative Promoter Activity
(% IL-1β Response)
0
50
100
150
-10
-9
-8
-7
-6
-5
PN3

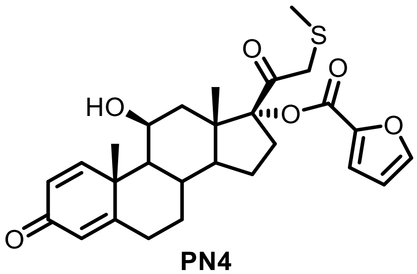

PN4

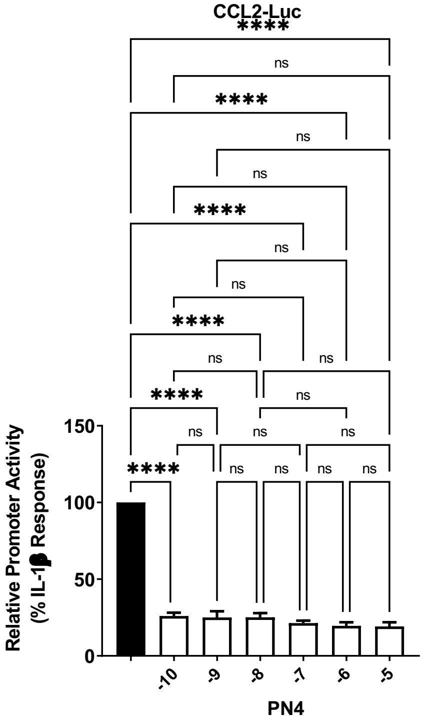
CCL2-Luc
Relative Promoter Activity
(% IL-1β Response)
0
50
100
150
-10
-9
-8
-7
-6
-5
PN4

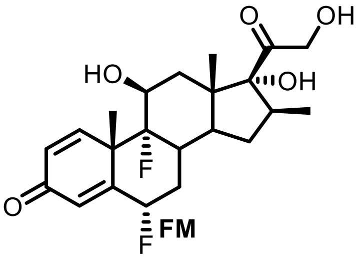

FM

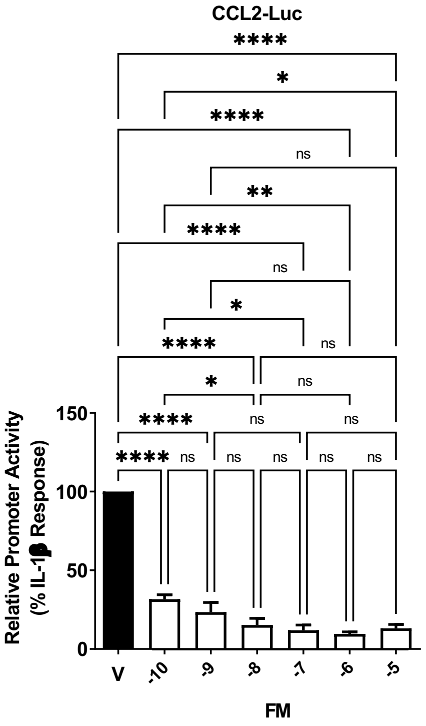

CCL2-Luc
Relative Promoter Activity (% IL-1β Response)
150
100
50
0
V
-10
-9
-8
-7
-6
-5
FM

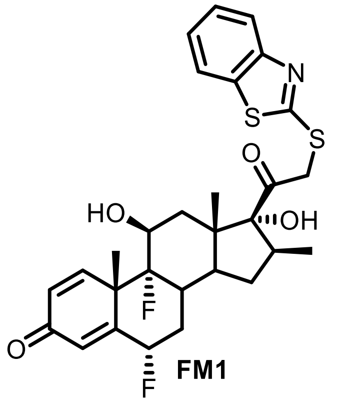

FM1

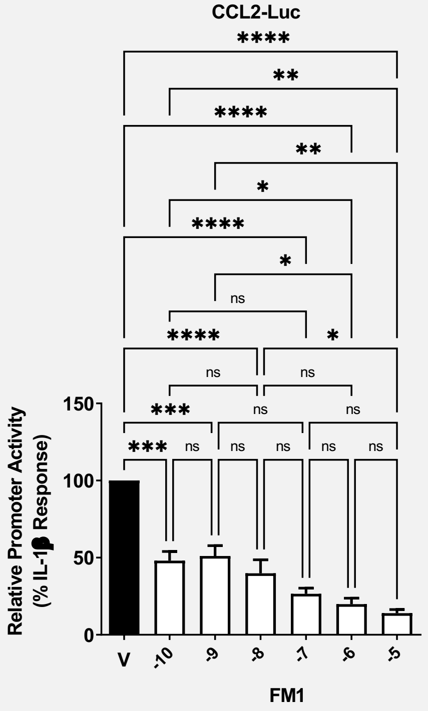

CCL2-Luc
Relative Promoter Activity (% IL-1β Response)
150
100
50
0
V
-10
-9
-8
-7
-6
-5
FM1

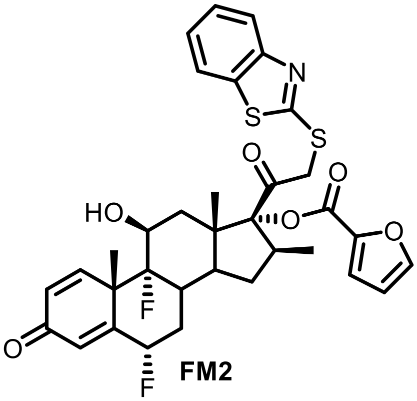

FM2

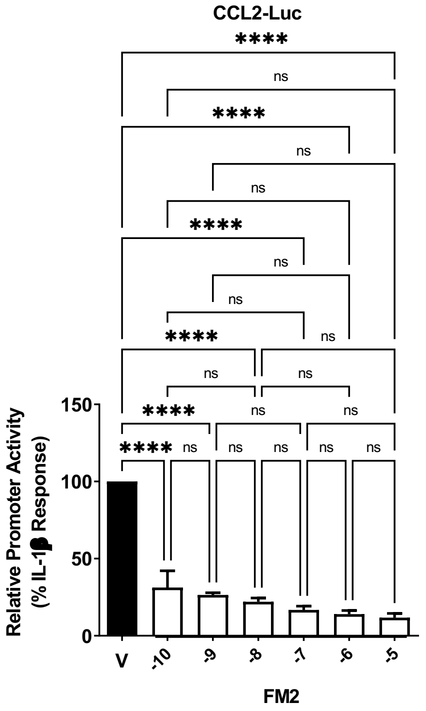
CCL2-Luc
Relative Promoter Activity
(% IL-1β Response)
150
100
50
0
V
-10
-9
-8
-7
-6
-5
FM2

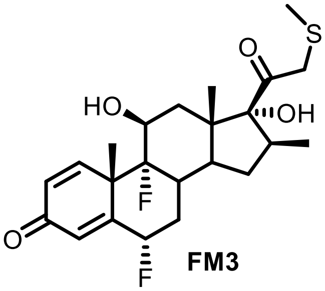

FM3

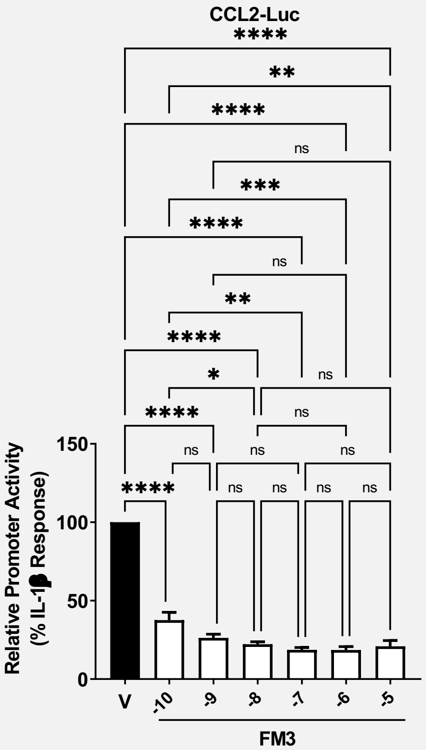
CCL2-Luc
Relative Promoter Activity
(% IL-1β Response)
150
100
50
0
V
-10
-9
-8
-7
-6
-5
FM3

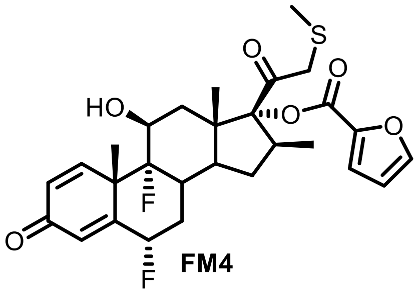
HO
O
S
O
O
O
F
F

FM4

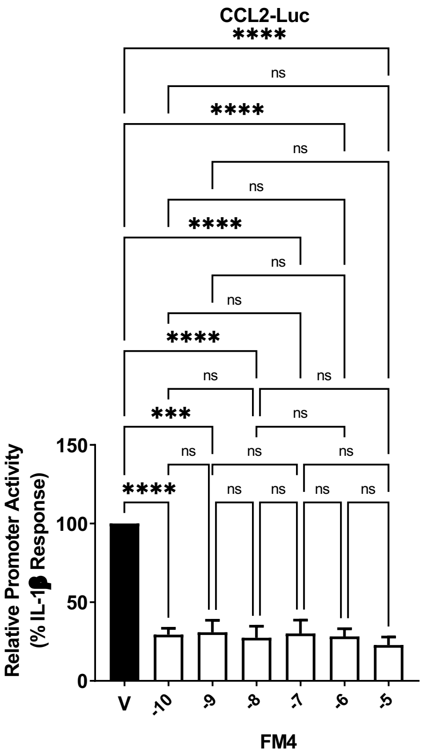
CCL2-Luc
Relative Promoter Activity
(% IL-1β Response)
150
100
50
0
V
-10
-9
-8
-7
-6
-5
FM4
****
***
ns

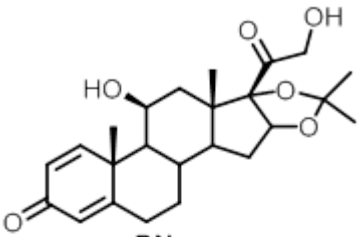
HO
O
OH
O
O
O

DN

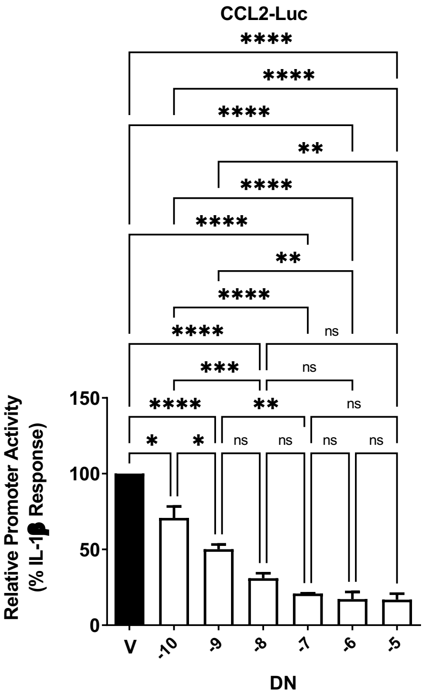
CCL2-Luc
Relative Promoter Activity
(% IL-1β Response)
150
100
50
0
V
-10
-9
-8
-7
-6
-5
DN
****
***
**
*
ns

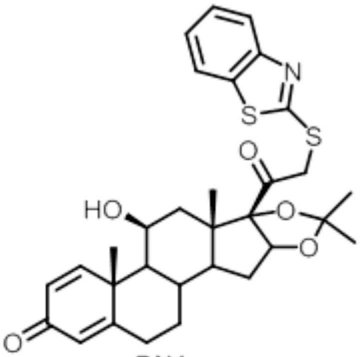
DN1

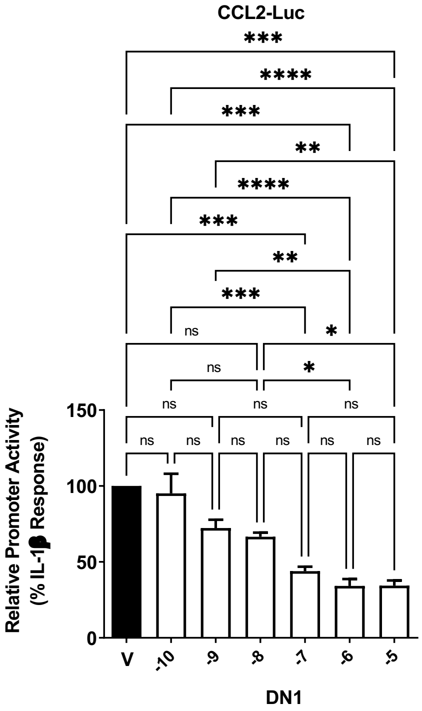
CCL2-Luc
Relative Promoter Activity
(% IL-1β Response)
150
100
50
0
V
-10
-9
-8
-7
-6
-5
DN1

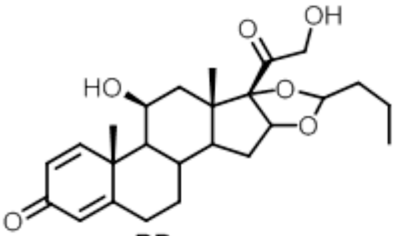
BD

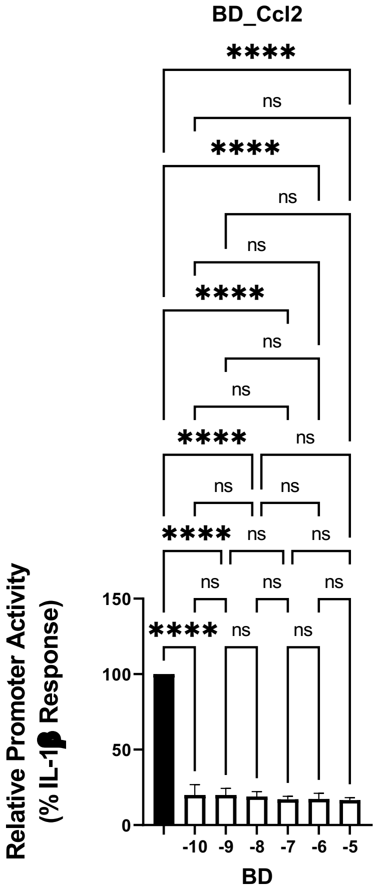
BD_Ccl2
Relative Promoter Activity
(% IL-1β Response)
150
100
50
0
-10
-9
-8
-7
-6
-5
BD

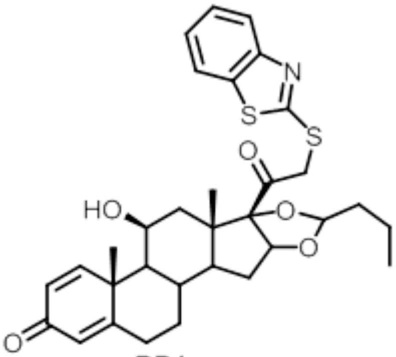

BD1

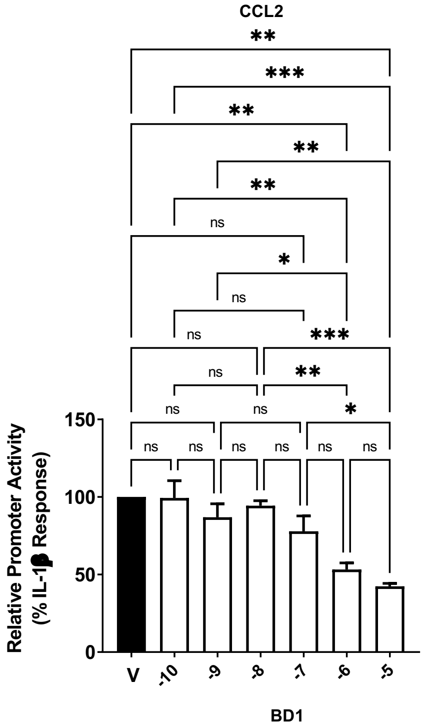
CCL2
Relative Promoter Activity
(% IL-1β Response)
150
100
50
0
V
-10
-9
-8
-7
-6
-5
BD1

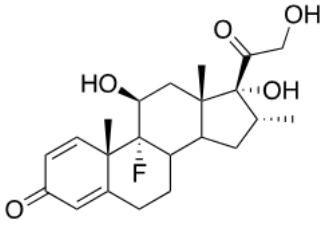

DX

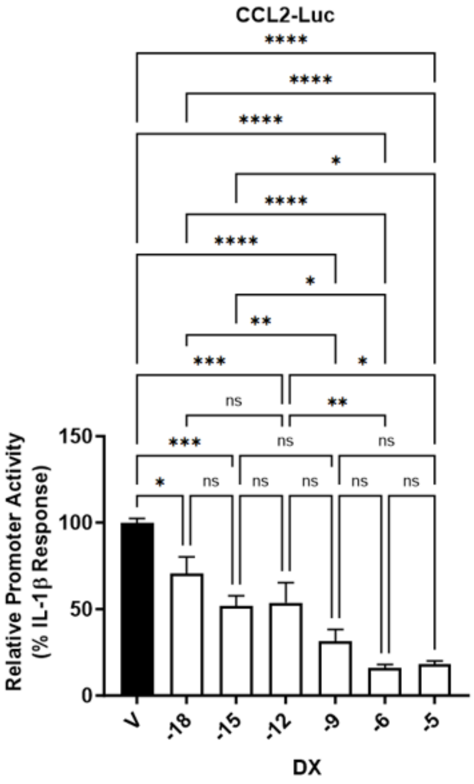
CCL2-Luc
Relative Promoter Activity
(% IL-1β Response)
150
100
50
0
V
-18
-15
-12
-9
-6
-5
DX

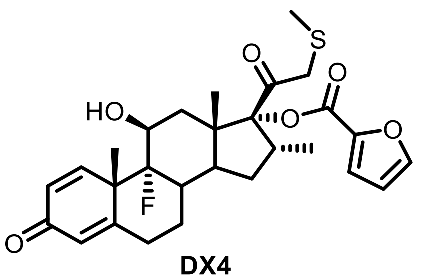
HO
O
S
O
O
O
F
O

DX4

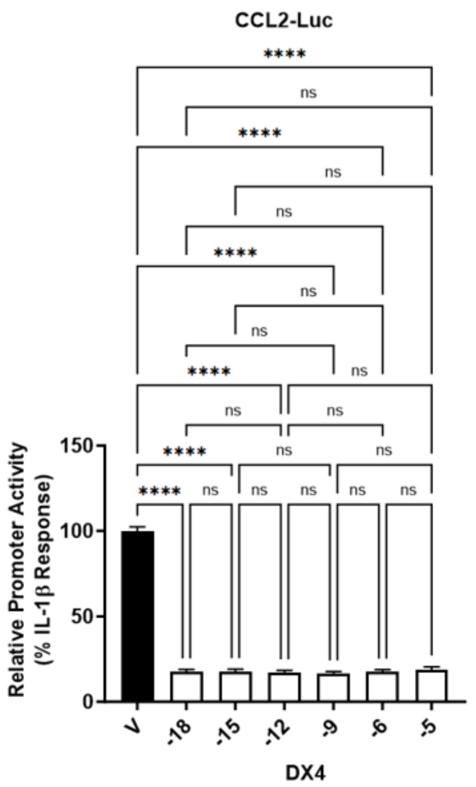
CCL2-Luc
Relative Promoter Activity
(% IL-1β Response)
150
100
50
0
V
-18
-15
-12
-9
-6
-5
DX4

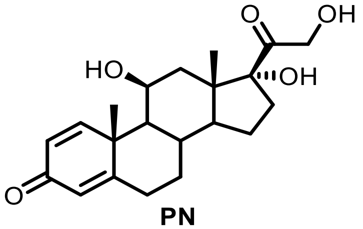
HO
O
OH
OH
O

PN

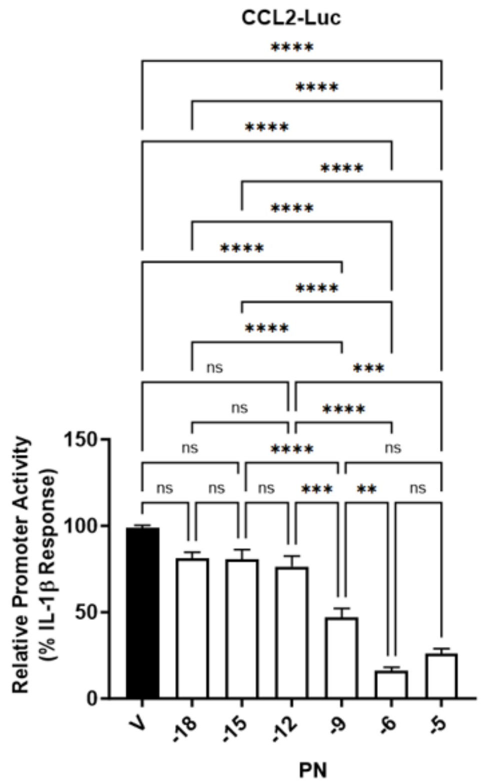
CCL2-Luc
Relative Promoter Activity
(% IL-1β Response)
150
100
50
0
V
-18
-15
-12
-9
-6
-5
PN

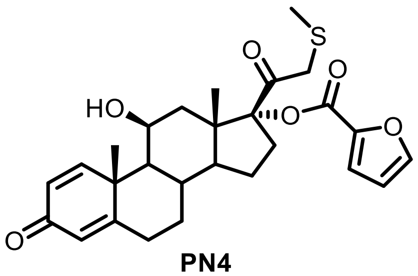

PN4

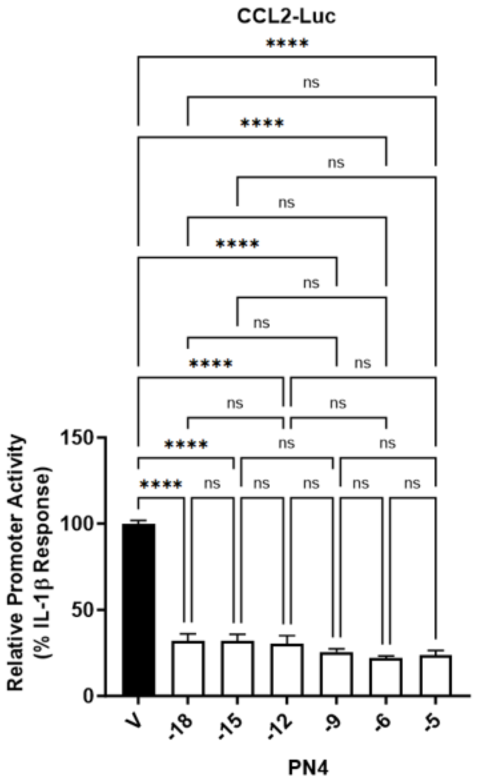
CCL2-Luc
Relative Promoter Activity
(% IL-1β Response)
150
100
50
0
V
-18
-15
-12
-9
-6
-5
PN4
****
ns

5. 3xGRE Promoter Activity

Table S2A: 3xGRE-Luc Assay Raw Data

	3xGRE (Fold over control – DMSO)					
	Concentration of Compound (M)					
Compd	1X10^-10	1X10^-9	1X10^-8	1X10^-7	1X10^-6	1X10^-5
DX	1.04±0.24	1.56±1.22	1.89±1.58	3.55±0.97	7.75±2.84	7.86±3.36
HC3	5.65±2.15	5.30±2.32	5.13±2.22	4.85±1.39	4.72±2.64	5.06±2.44
HC4	3.94±0.41	3.99±0.28	3.86±0.08	4.08±0.34	3.76±0.29	3.25±0.81
DX1	0.65±0.12	0.73±0.15	0.73±0.23	0.73±0.13	0.96±0.42	0.99±0.31
DX2	0.81±0.06	0.85±0.20	1.84±1.35	2.39±2.04	3.78±2.88	4.89±3.13
DX3	7.42±4.16	6.21±3.02	6.19±3.44	6.20±2.76	5.77±3.29	6.65±3.79
DX4	8.88±4.10	8.07±4.50	8.55±4.55	8.51±4.25	7.34±3.63	7.78±4.02
DX5	1.06±0.19	0.926±0.19	0.84±0.08	0.95±0.12	1.98±0.53	3.20±0.68
BM	6.52±2.20	8.98±2.28	10.3±2.94	10.3±2.91	9.38±2.21	9.42±3.87
BM1	0.80±0.14	0.85±0.13	0.93±0.17	1.68±0.25	1.95±0.90	2.20±0.82
BM2	1.62±0.85	1.89±0.81	2.52±0.74	4.75±1.68	4.58±1.37	4.71±1.05
BM3	3.54±1.70	4.10±1.89	4.07±1.96	4.32±1.85	4.54±1.39	4.76±1.49
BM4	0.93±0.12	0.94±0.18	1.24±0.06	4.34±1.49	4.71±1.68	4.87±1.87
PN	3.50±1.72	6.58±2.91	8.03±2.14	10.0±3.22	7.80±1.55	7.58±1.11
PN1	0.95±0.25	0.85±0.21	0.95±0.13	1.08±0.13	1.85±0.40	2.40±0.96
PN2	2.16±1.34	3.07±0.75	6.76±0.90	7.54±1.55	6.06±1.82	6.24±0.75
PN3	1.06±0.42	1.29±0.49	1.10±0.17	1.85±0.34	2.97±0.70	3.4±0.86
PN4	4.95±1.41	4.59±1.40	4.71±1.15	5.44±1.61	5.29±1.63	5.19±1.38
FM	9.20±4.51	8.48±2.30	8.73±3.36	10.1±4.90	8.73±5.30	10.2±8.71
FM1	2.38±0.31	3.18±0.97	4.40±1.78	4.53±1.79	4.75±1.71	4.68±1.37
FM2	4.60±1.31	6.06±2.39	6.88±1.34	11.7±5.18	9.71±1.42	11.7±7.07
FM3	4.86±0.55	4.72±1.09	5.52±1.03	5.45±1.29	5.63±1.74	4.91±1.13
FM4	4.47±1.73	4.57±0.83	4.92±0.71	4.82±0.69	4.83±1.06	4.46±0.77
DN	5.00±3.55	9.57±3.09	8.33±0.47	8.90±0.85	8.47±0.55	9.33±0.86
DN1	0.88±0.19	0.98±0.25	0.98±0.30	1.68±0.49	2.35±0.69	2.50±0.94
BD	6.30±0.85	6.97±0.85	6.33±0.79	6.71±0.51	6.36±0.33	6.01±0.72
BD1	0.90±0.14	0.95±0.11	0.89±0.12	0.98±0.16	1.50±0.19	1.99±0.20

Table S2B: Low-Dose 3xGRE-Luc Assay Raw Data

	3xGRE (Fold over control – DMSO)					
	Concentration of Compound (M)					
Compd	1X10^-18	1X10^-15	1X10^-12	1X10^-9	1X10^-6	1X10^-5
DX	0.85±0.13	2.94±3.43	3.72±3.83	7.85±1.18	8.75±1.29	9.71±2.06
DX4	6.70±1.16	6.75±1.70	6.67±1.49	6.95±1.48	6.94±1.64	8.82±1.74
PN	2.24±1.97	1.602±1.934	1.17±0.33	4.46±1.49	5.01±2.16	6.21±1.59
PN4	3.85±2.10	4.42±1.29	4.91±0.87	4.99±1.61	4.95±1.61	5.70±1.08

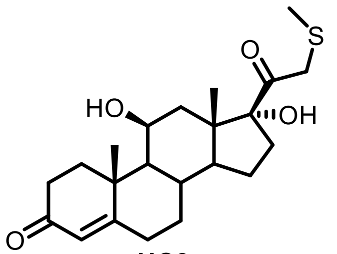

HC3

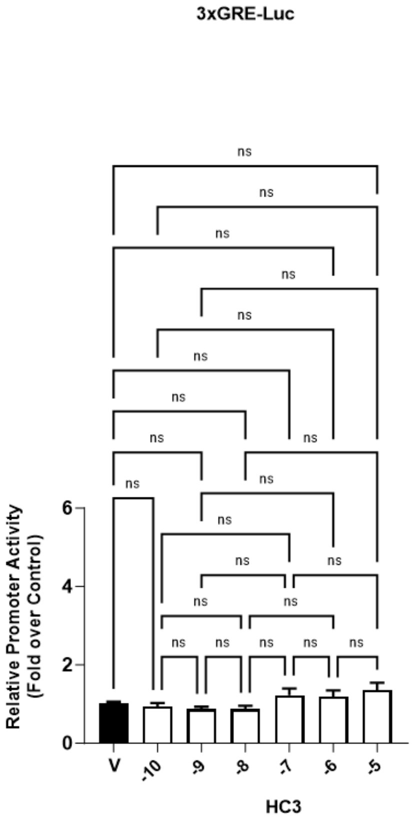

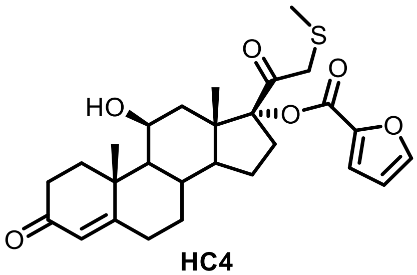

HC4

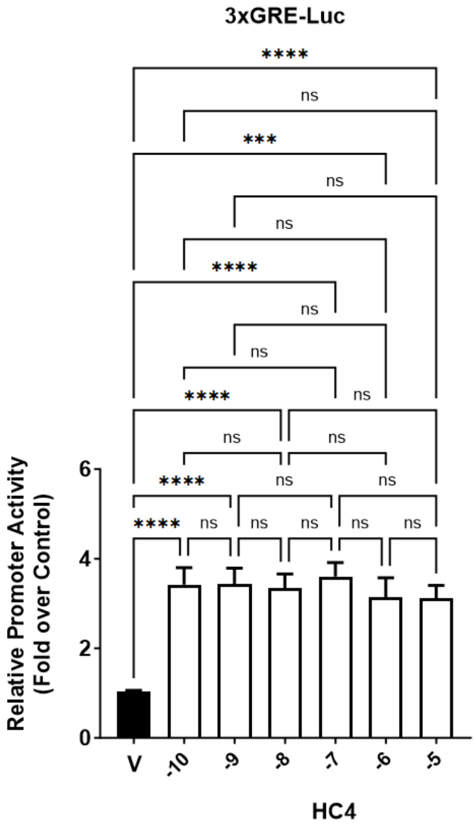

3xGRE-Luc
Relative Promoter Activity (Fold over Control)
V
-10
-9
-8
-7
-6
-5
HC4

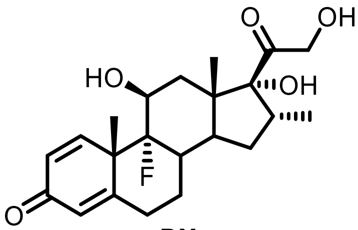

DX

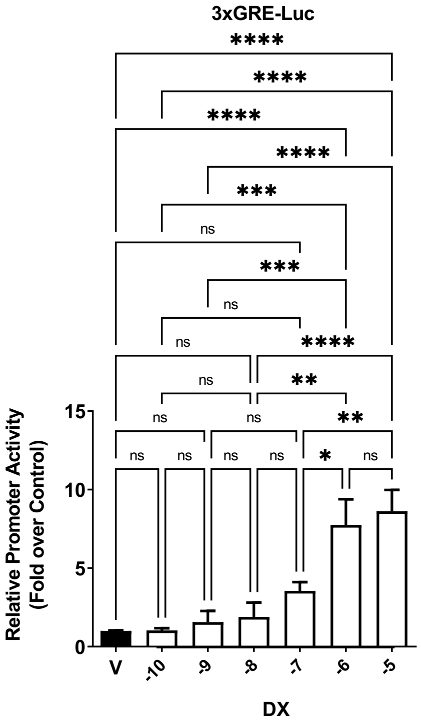

3xGRE-Luc
Relative Promoter Activity (Fold over Control)
V
-10
-9
-8
-7
-6
-5
DX

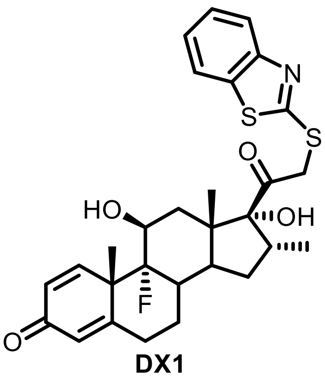

DX1

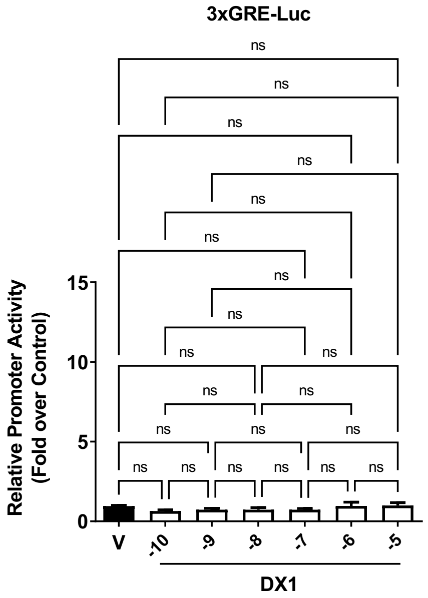
3xGRE-Luc
Relative Promoter Activity (Fold over Control)
15
10
5
0
V
-10
-9
-8
-7
-6
-5
DX1
ns

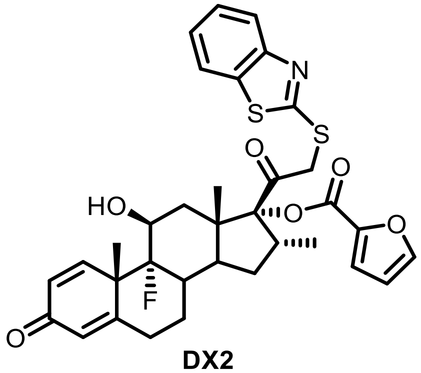

DX2

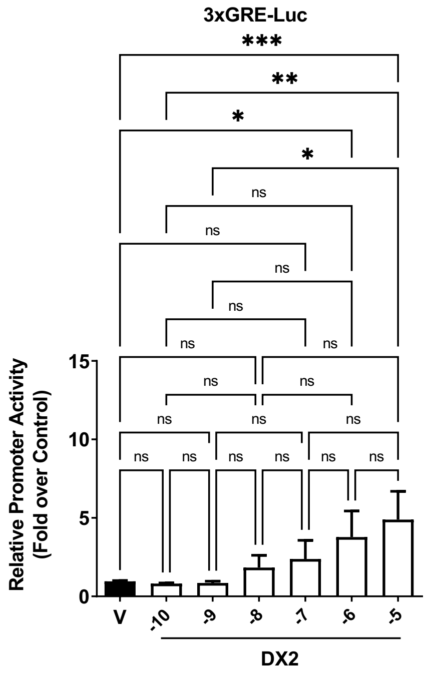
3xGRE-Luc
***
**
*
*
ns
Relative Promoter Activity (Fold over Control)
15
10
5
0
V
-10
-9
-8
-7
-6
-5
DX2

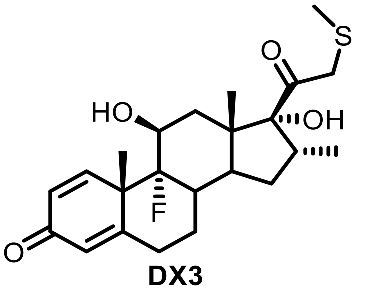
O
S
HO
OH
F
O

DX3

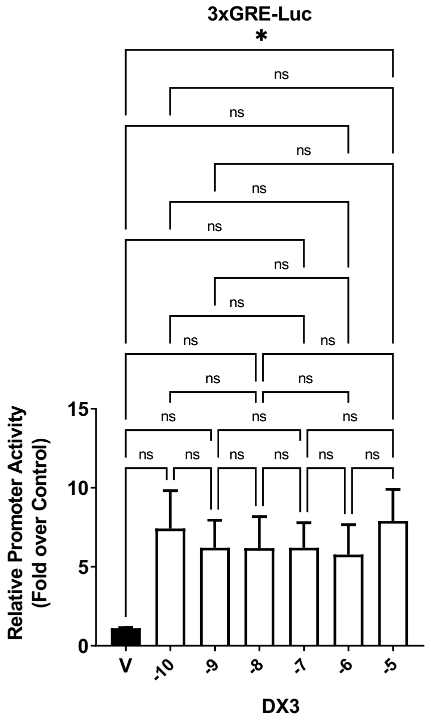
3xGRE-Luc
*
ns
Relative Promoter Activity
(Fold over Control)
0
5
10
15
V
-10
-9
-8
-7
-6
-5
DX3

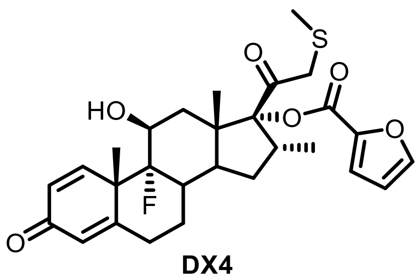
S
O
O
HO
O
O
F
O

DX4

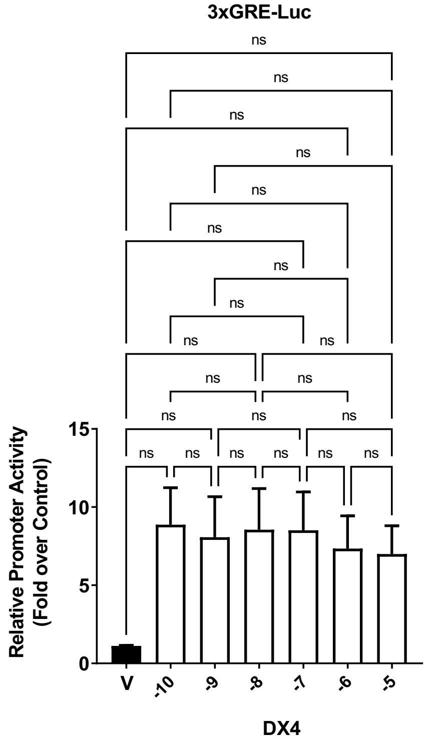
3xGRE-Luc
ns
Relative Promoter Activity
(Fold over Control)
0
5
10
15
V
-10
-9
-8
-7
-6
-5
DX4

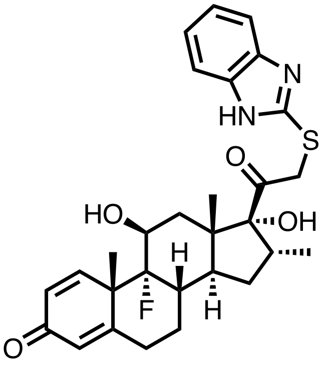

DX5

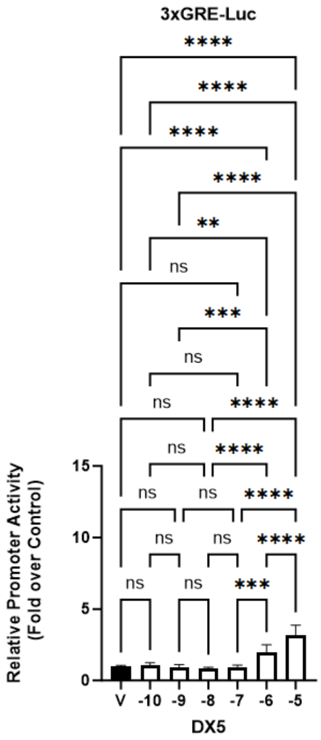
3xGRE-Luc
Relative Promoter Activity (Fold over Control)
V -10 -9 -8 -7 -6 -5
DX5

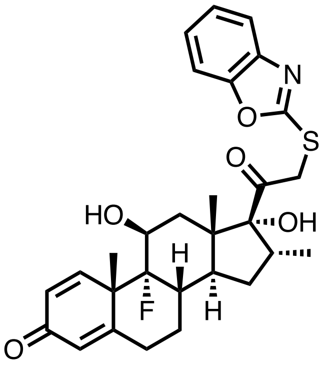

DX6

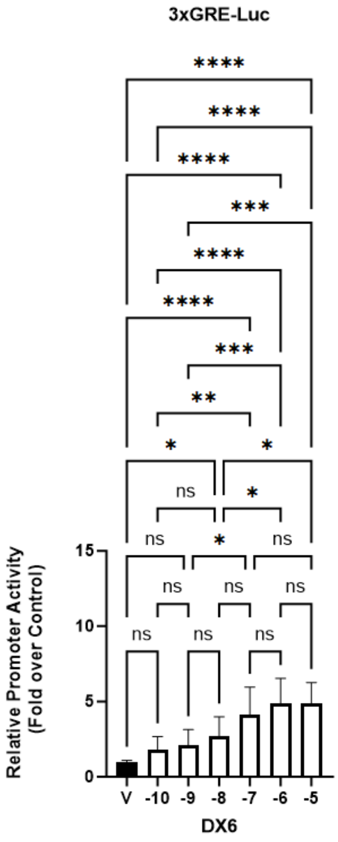
3xGRE-Luc
Relative Promoter Activity (Fold over Control)
V -10 -9 -8 -7 -6 -5
DX6

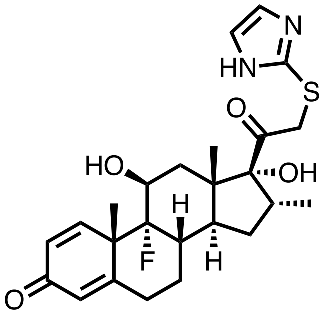
HN
N
S
O
HO
OH
H
F
H
O

DX7

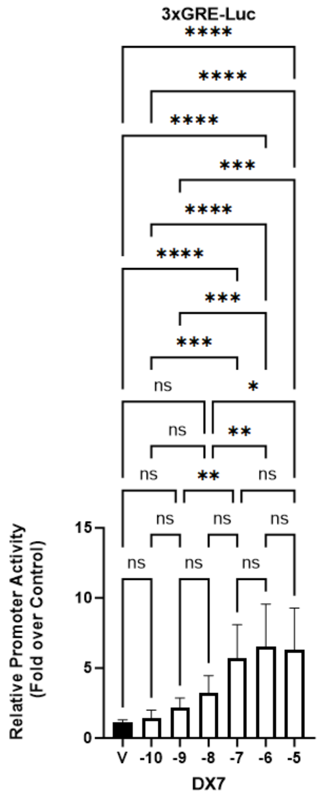
3xGRE-Luc
Relative Promoter Activity
(Fold over Control)
15
10
5
0
V
-10
-9
-8
-7
-6
-5
DX7

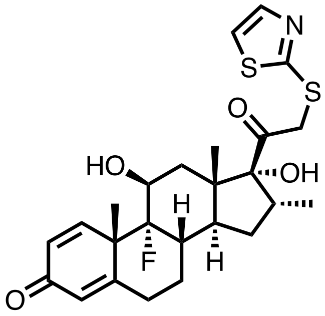
N
S
S
O
HO
OH
H
F
H
O

DX8

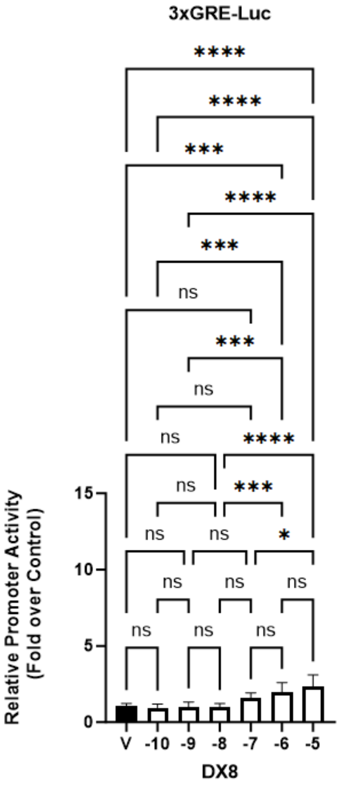
3xGRE-Luc
Relative Promoter Activity
(Fold over Control)
15
10
5
0
V
-10
-9
-8
-7
-6
-5
DX8

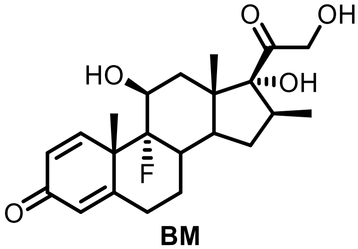
O
OH
HO
OH
F
O

BM

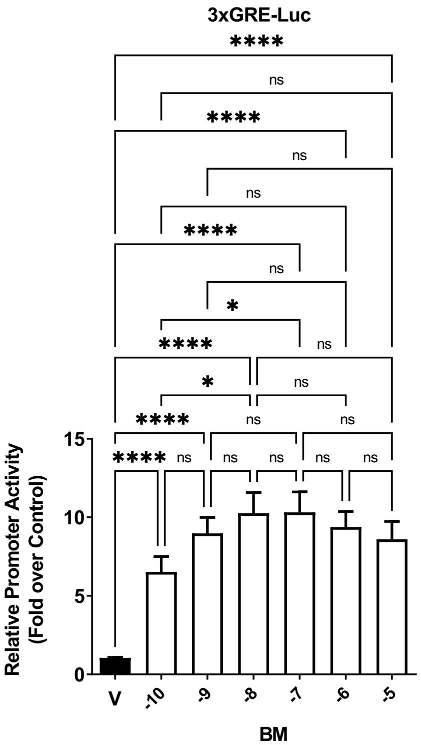
3xGRE-Luc
Relative Promoter Activity
(Fold over Control)
0
5
10
15
V
-10
-9
-8
-7
-6
-5
BM

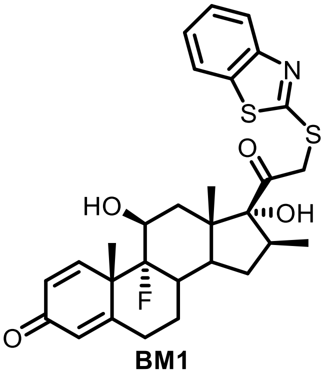
N
S
S
O
HO
OH
F
O

BM1

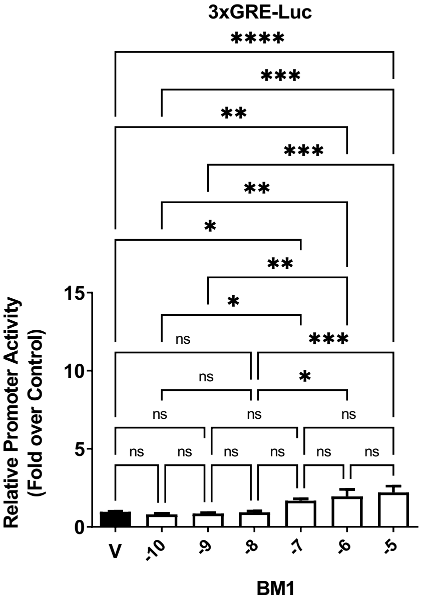
3xGRE-Luc
Relative Promoter Activity
(Fold over Control)
0
5
10
15
V
-10
-9
-8
-7
-6
-5
BM1

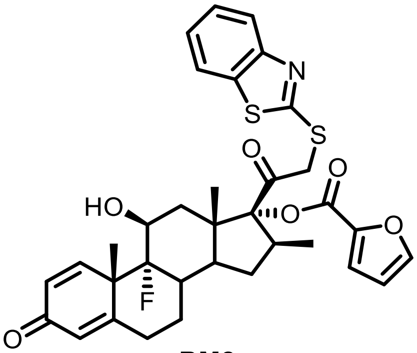

BM2

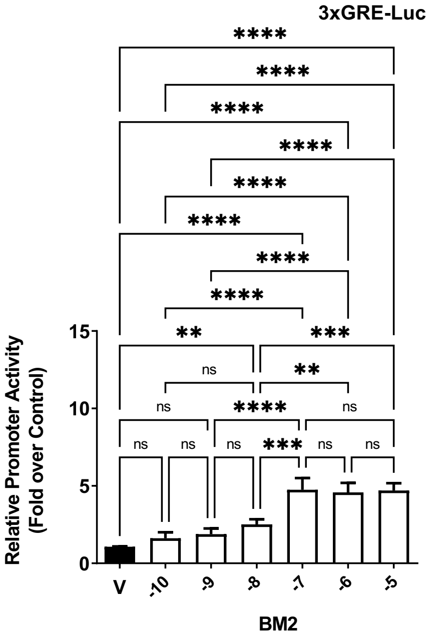
3xGRE-Luc
****
***
**
ns
Relative Promoter Activity (Fold over Control)
0
5
10
15
V
-10
-9
-8
-7
-6
-5
BM2

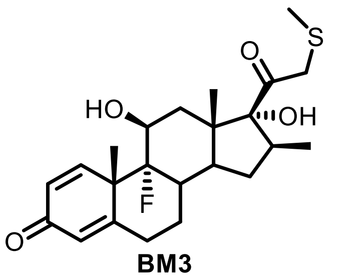

BM3

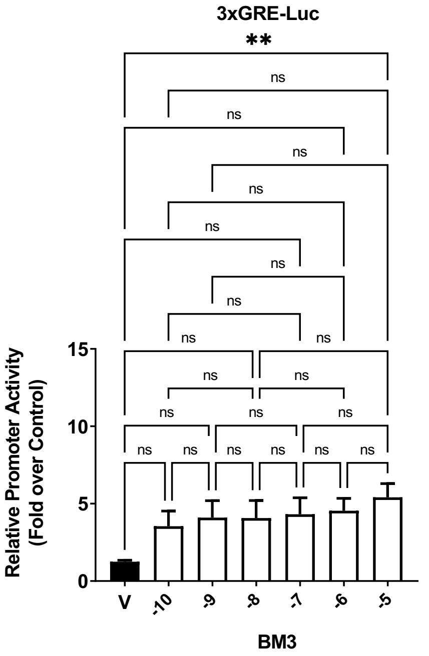
3xGRE-Luc
**
ns
Relative Promoter Activity (Fold over Control)
0
5
10
15
V
-10
-9
-8
-7
-6
-5
BM3

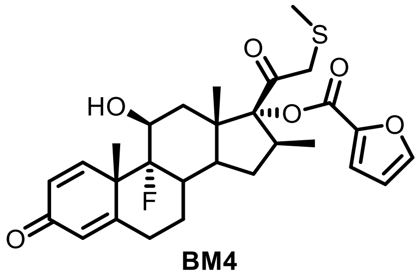

BM4

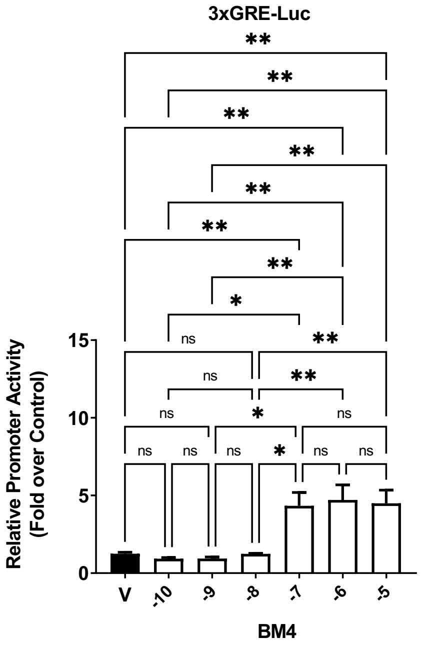
3xGRE-Luc
Relative Promoter Activity
(Fold over Control)
15
10
5
0
V
-10
-9
-8
-7
-6
-5
BM4

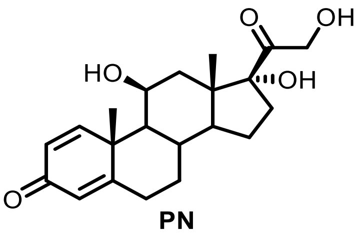

PN

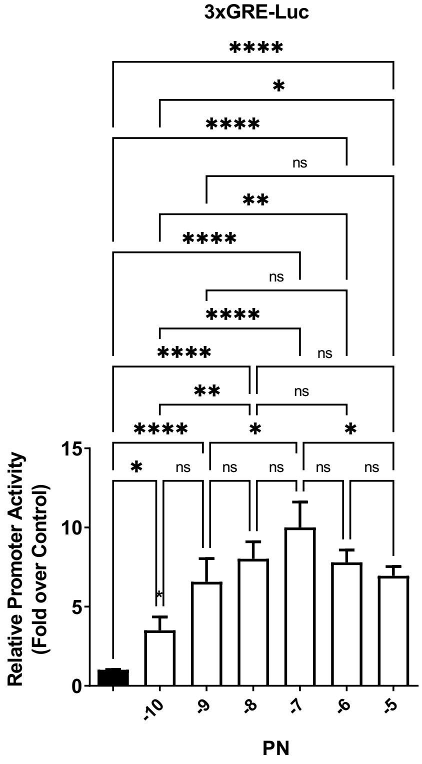
3xGRE-Luc
Relative Promoter Activity
(Fold over Control)
15
10
5
0
-10
-9
-8
-7
-6
-5
PN

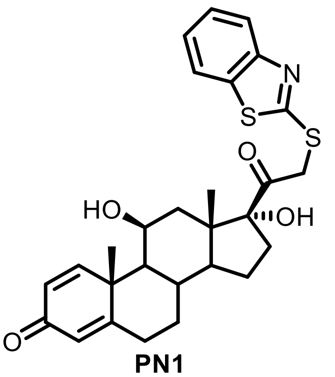
N
S
S
O
HO
OH
O

PN1

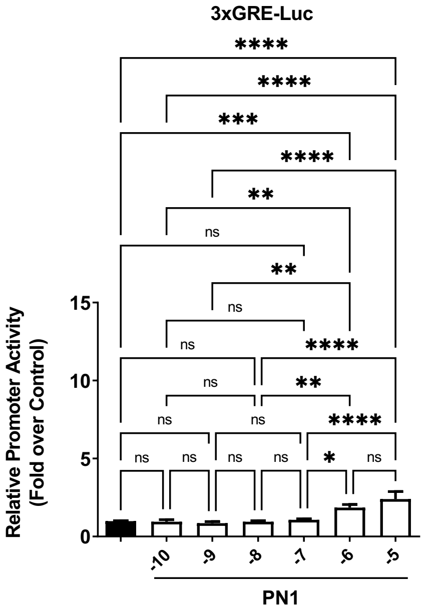
3xGRE-Luc
Relative Promoter Activity (Fold over Control)
0
5
10
15
-10
-9
-8
-7
-6
-5
PN1

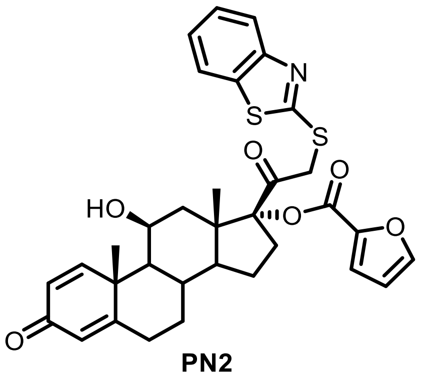
N
S
S
O
O
HO
O
O
O

PN2

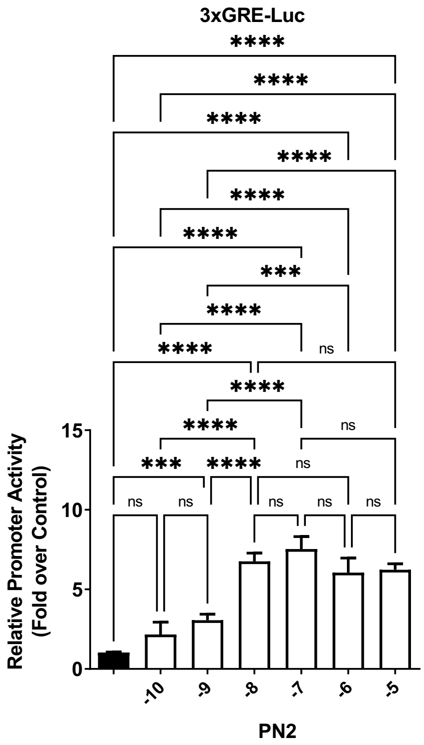
3xGRE-Luc
Relative Promoter Activity (Fold over Control)
0
5
10
15
-10
-9
-8
-7
-6
-5
PN2

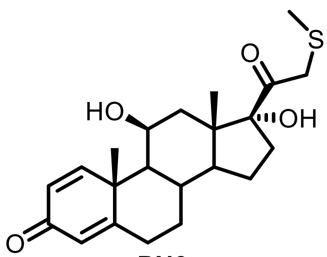

PN3

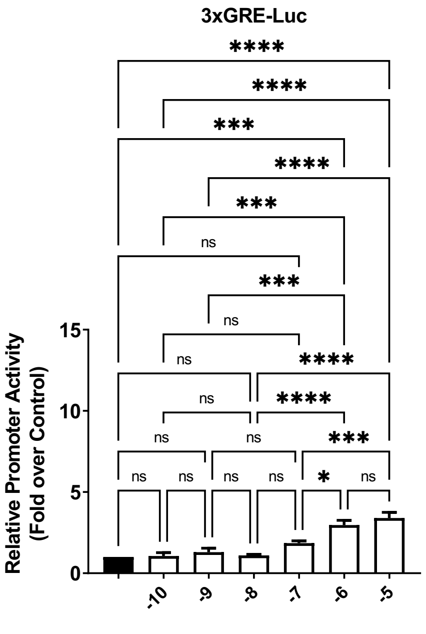

3xGRE-Luc
Relative Promoter Activity (Fold over Control)
PN3

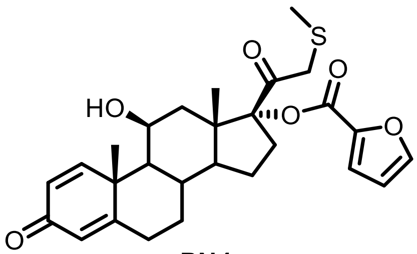

PN4

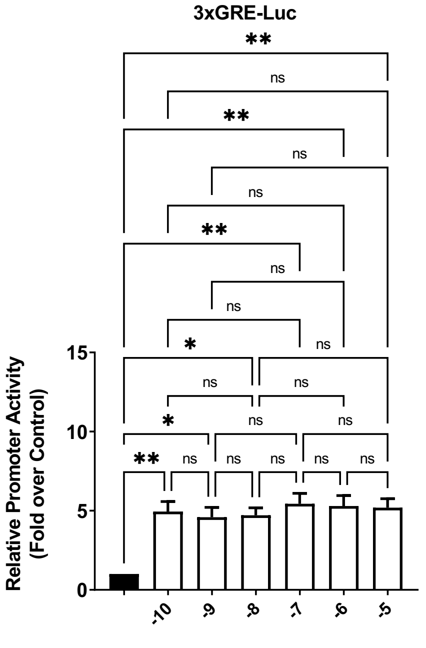

3xGRE-Luc
Relative Promoter Activity (Fold over Control)
PN4

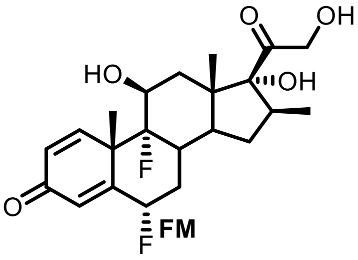
HO
OH
O
F

FM

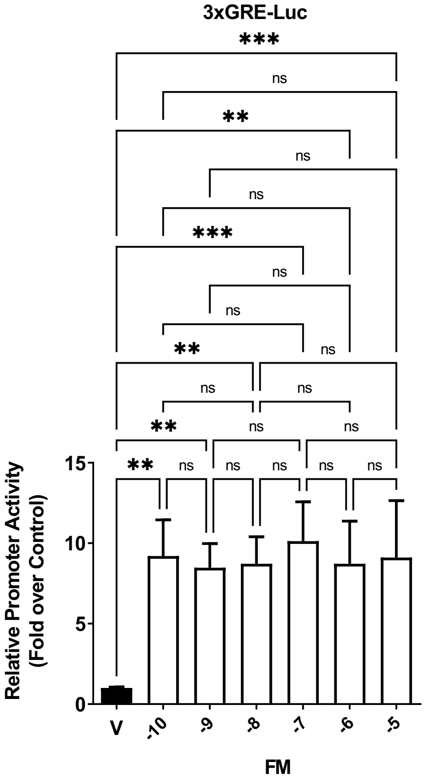
3xGRE-Luc
Relative Promoter Activity (Fold over Control)
0
5
10
15
V
-10
-9
-8
-7
-6
-5
FM

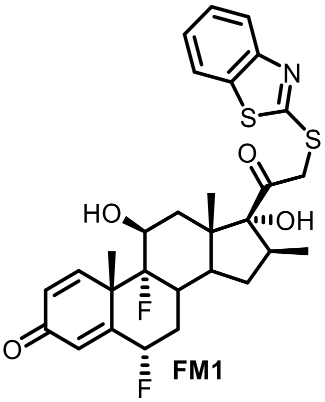
N
S
HO
OH
O
F

FM1

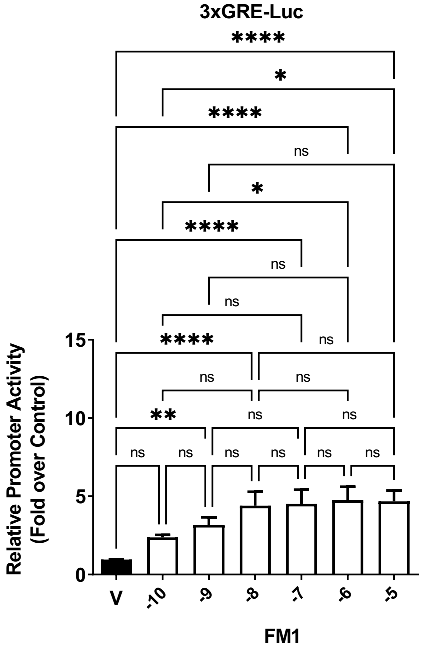
3xGRE-Luc
Relative Promoter Activity (Fold over Control)
0
5
10
15
V
-10
-9
-8
-7
-6
-5
FM1

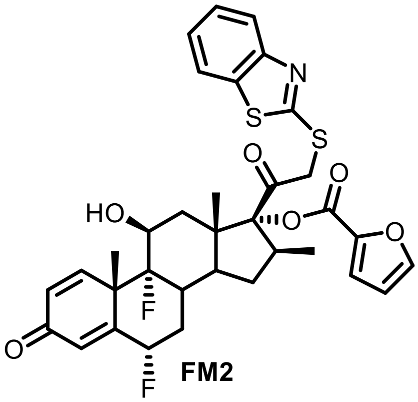

FM2

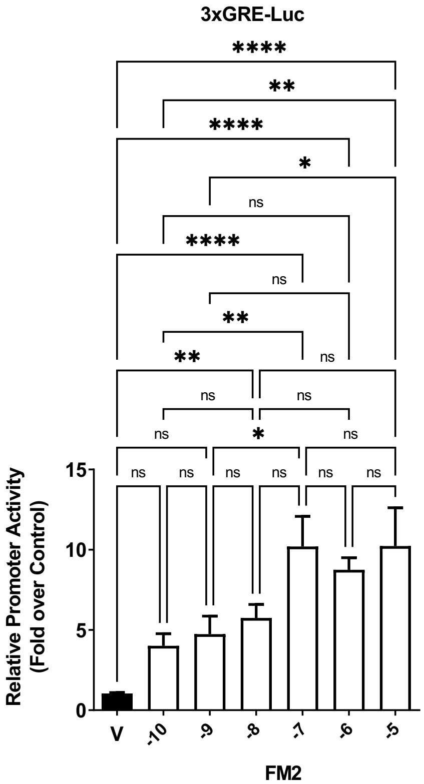
3xGRE-Luc
Relative Promoter Activity
(Fold over Control)
15
10
5
0
V
-10
-9
-8
-7
-6
-5
FM2
****
**
*
ns

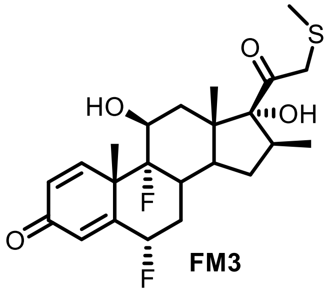

FM3

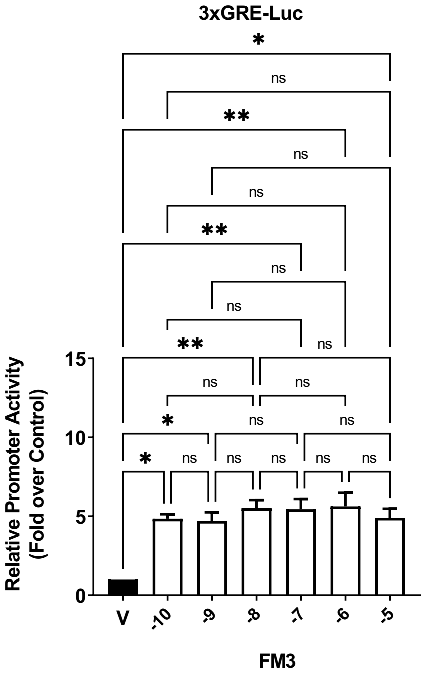
3xGRE-Luc
Relative Promoter Activity
(Fold over Control)
15
10
5
0
V
-10
-9
-8
-7
-6
-5
FM3
**
*
ns

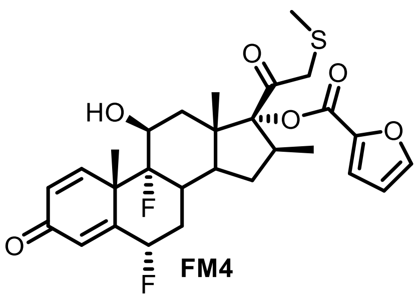

FM4

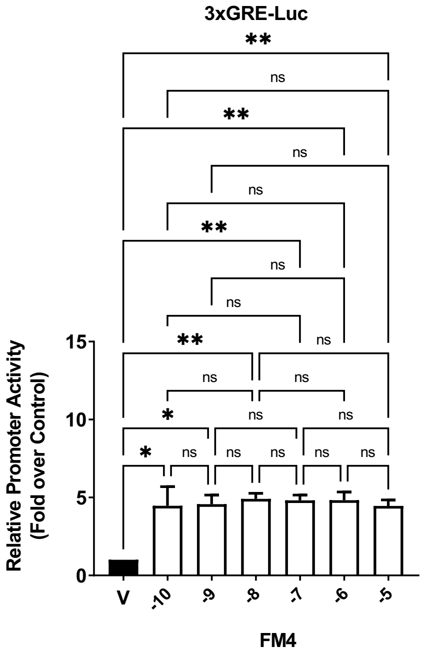

3xGRE-Luc
Relative Promoter Activity (Fold over Control)
FM4

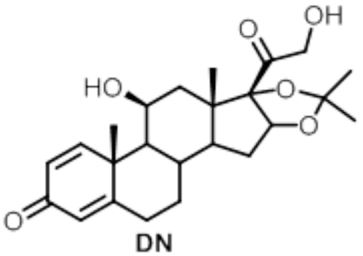

DN

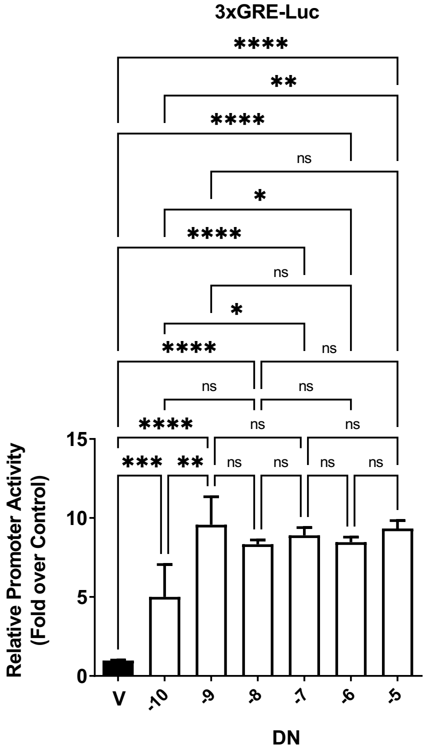

3xGRE-Luc
Relative Promoter Activity (Fold over Control)
DN

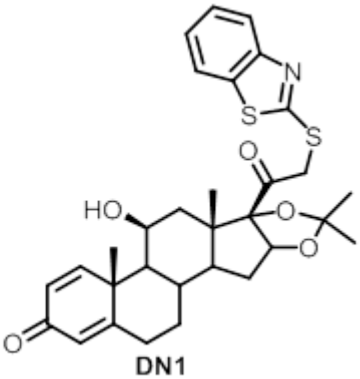
HO
O
S
N
S
O
O
O

DN1

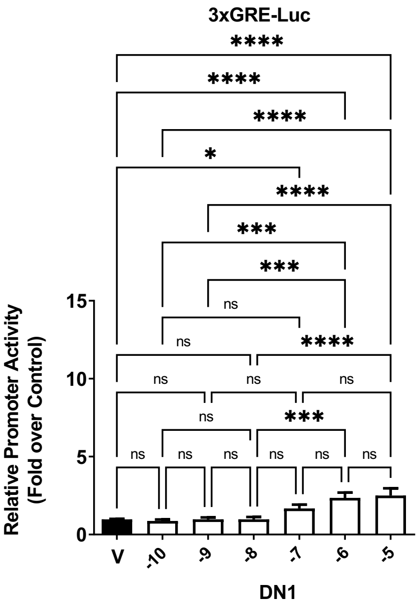
3xGRE-Luc
Relative Promoter Activity
(Fold over Control)
15
10
5
0
V
-10
-9
-8
-7
-6
-5
DN1

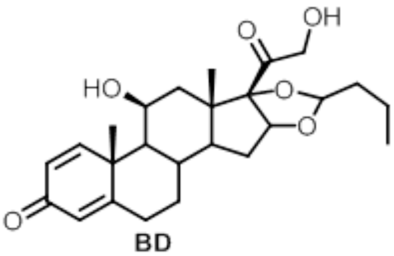
HO
O
OH
O
O
O

BD

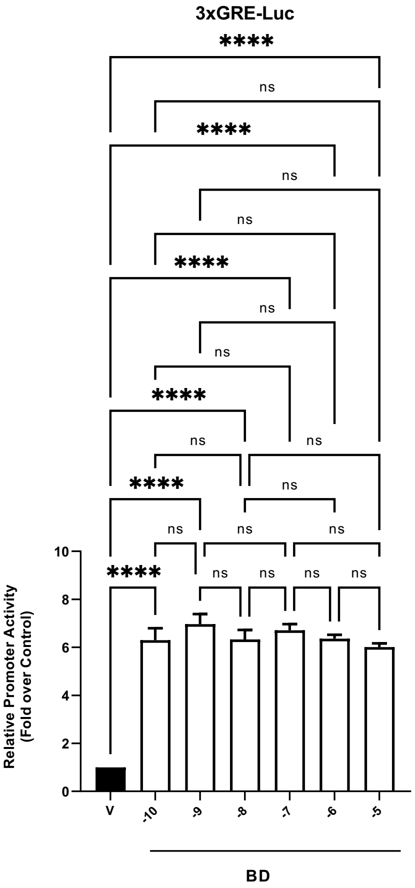
3xGRE-Luc
Relative Promoter Activity
(Fold over Control)
10
8
6
4
2
0
V
-10
-9
-8
-7
-6
-5
BD

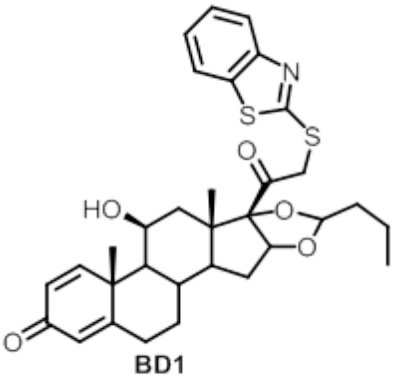
HO
O
S
N
O
O

BD1

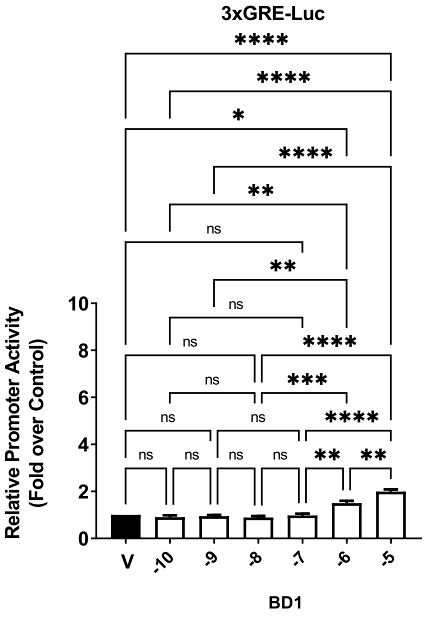
3xGRE-Luc
Relative Promoter Activity (Fold over Control)
V
-10
-9
-8
-7
-6
-5
BD1

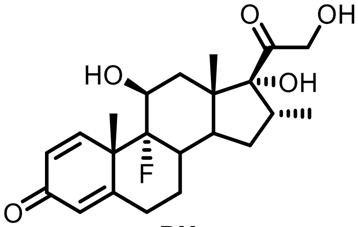
O
OH
HO
OH
F
O

DX

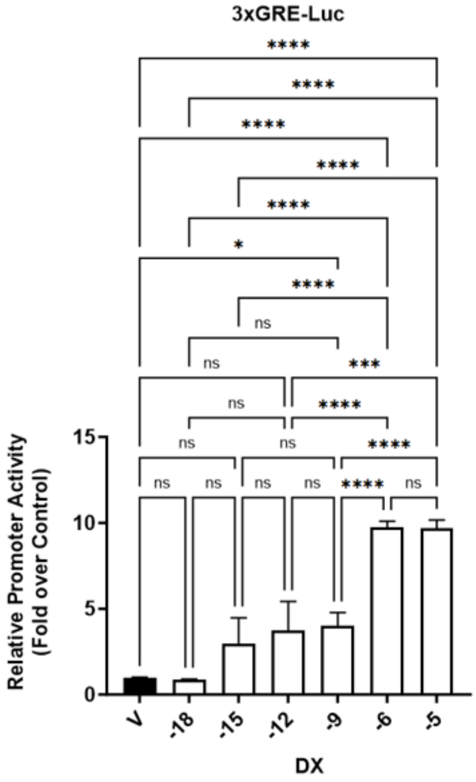
3xGRE-Luc
Relative Promoter Activity (Fold over Control)
V
-18
-15
-12
-9
-6
-5
DX

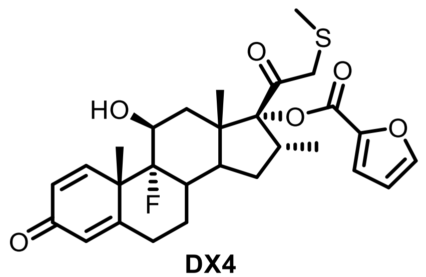
HO
O
S
O
O
O
F
O

DX4

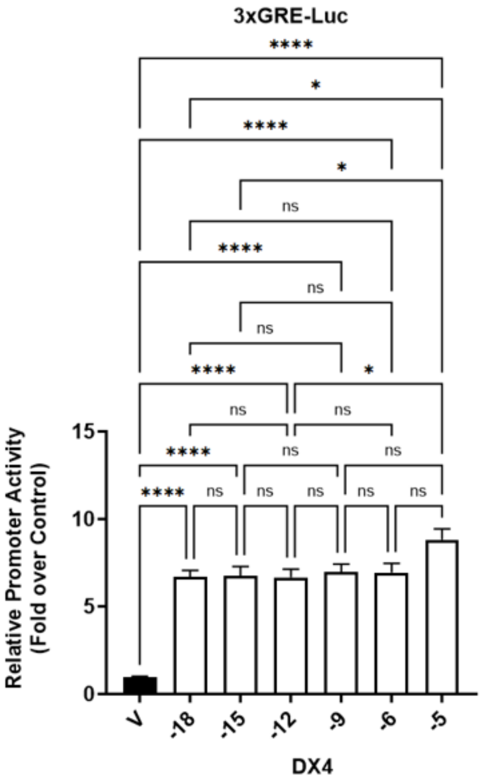
3xGRE-Luc
Relative Promoter Activity (Fold over Control)
V
-18
-15
-12
-9
-6
-5
DX4
****
*
ns

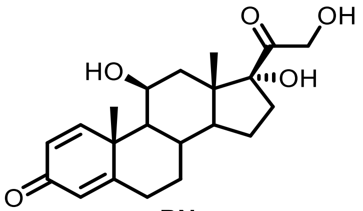

PN

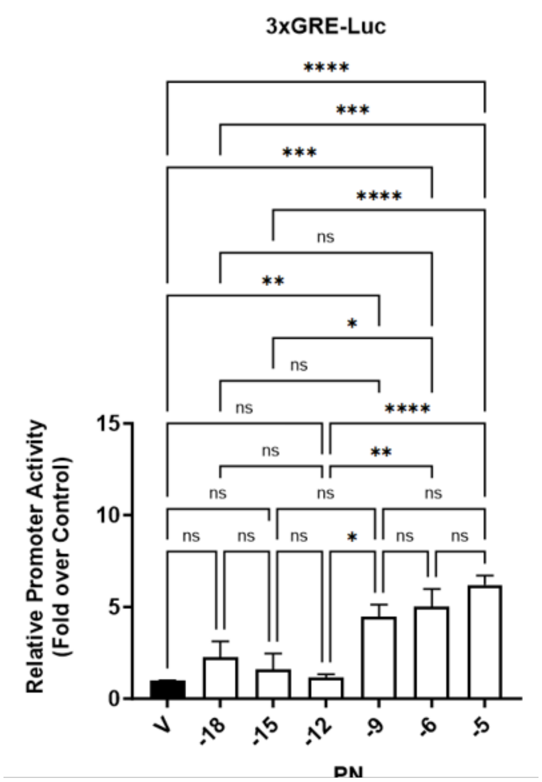
3xGRE-Luc
Relative Promoter Activity (Fold over Control)
0
5
10
15
V
-18
-15
-12
-9
-6
-5
PN

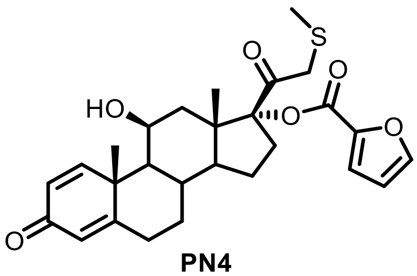

PN4

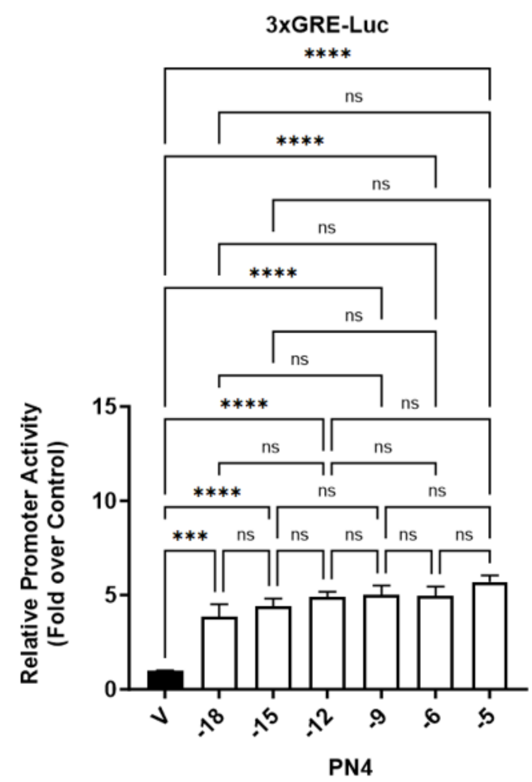
3xGRE-Luc
Relative Promoter Activity (Fold over Control)
0
5
10
15
V
-18
-15
-12
-9
-6
-5
PN4

6. Adenylate Kinase Release

Table S3: ADK Assay Raw Data

	ADK (Fold Over Control) – 100 nM
DX	1.60±0.16
HC	1.47±0.10
HC1	1.10±0.22
HC2	1.24±0.18
HC3	1.19±0.07
HC4	1.31±0.13
DX1	1.26±0.18
DX2	1.40±0.23
DX3	1.38±0.11
DX4	1.38±0.11
BM	1.63±0.19
BM1	1.23±0.07
BM2	1.18±0.20
BM3	1.05±0.16
BM4	0.99±0.02
PN	1.72±0.53
PN1	1.35±0.24
PN2	1.69±0.29
PN3	1.44±0.41
PN4	1.52±0.42
FM	1.53±0.26
FM1	1.33±0.16
FM2	1.24±0.17
FM3	1.42±0.08
FM4	1.44±0.07
DN	1.73±0.19
DN1	1.34±0.15
BD	1.52±0.16
BD1	1.28±0.09

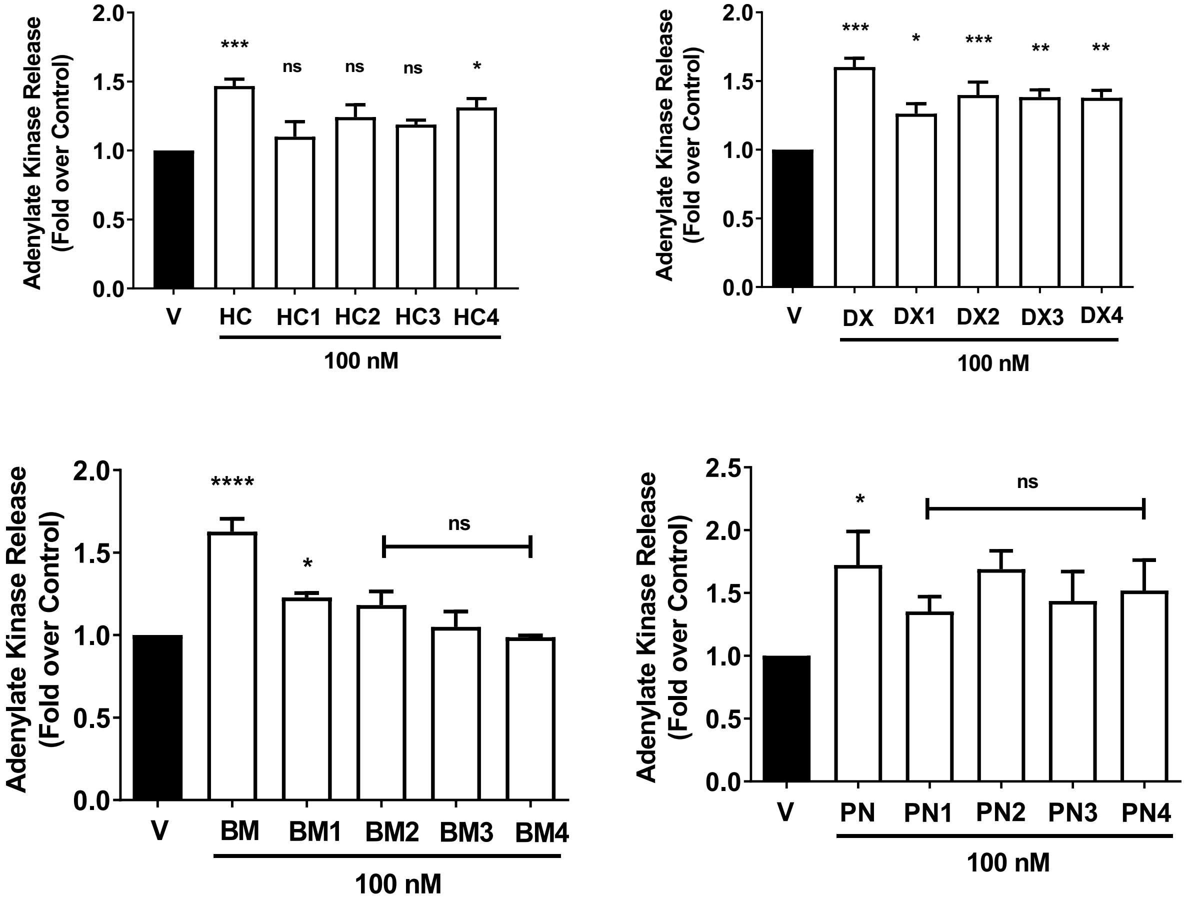
Adenylate Kinase Release (Fold over Control)
2.0
1.5
1.0
0.5
0.0
***
ns
ns
ns
*
V
HC
HC1
HC2
HC3
HC4
100 nM
Adenylate Kinase Release (Fold over Control)
2.0
1.5
1.0
0.5
0.0
***
*
***
**
**
V
DX
DX1
DX2
DX3
DX4
100 nM
Adenylate Kinase Release (Fold over Control)
2.0
1.5
1.0
0.5
0.0
****
*
ns
V
BM
BM1
BM2
BM3
BM4
100 nM
Adenylate Kinase Release (Fold over Control)
2.5
2.0
1.5
1.0
0.5
0.0
*
ns
V
PN
PN1
PN2
PN3
PN4
100 nM

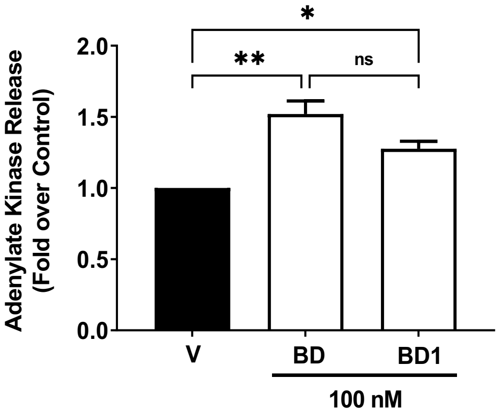
Adenylate Kinase Release
(Fold over Control)
2.0
1.5
1.0
0.5
0.0
*
**
ns
V
BD
BD1
100 nM

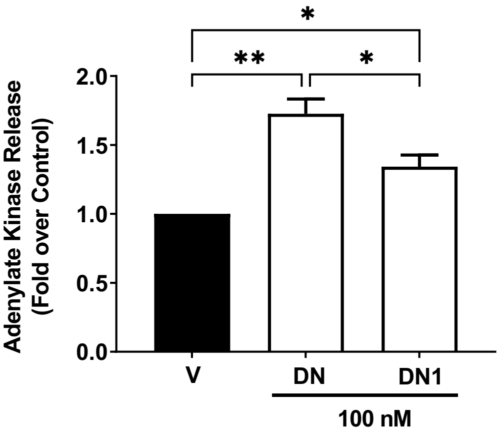
Adenylate Kinase Release
(Fold over Control)
2.0
1.5
1.0
0.5
0.0
*
**
*
V
DN
DN1
100 nM

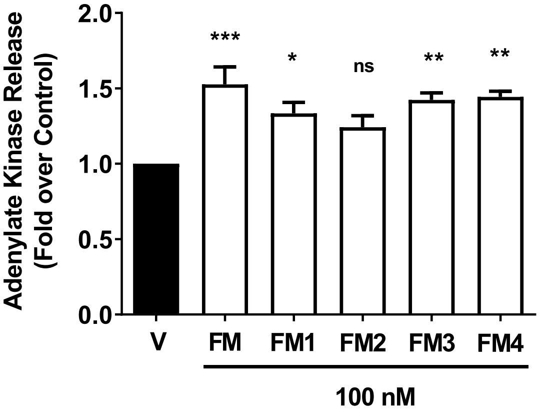
Adenylate Kinase Release
(Fold over Control)
2.0
1.5
1.0
0.5
0.0
***
*
ns
**
**
V
FM
FM1
FM2
FM3
FM4
100 nM

7. MTS Reduction

Table S4: MTS Assay Raw Data

	MTS Reduction (% of Control) – 100 nM
DX	63.4±12.2
HC	77.1±7.04
HC1	83.9±3.52
HC2	85.6±5.87
HC3	83.9±2.42
HC4	74.2±4.70
DX1	93.4±6.27
DX2	80.7±12.5
DX3	80.6±5.65
DX4	80.7±10.2
BM	66.5±6.04
BM1	79.6±11.0
BM2	79.7±10.8
BM3	69.1±8.39
BM4	72.4±13.0
PN	74.6±15.4
PN1	91.7±15.9
PN2	77.1±13.5
PN3	80.5±10.1
PN4	76.6±7.28
FM	84.9±22.2
FM1	88.8±13.7
FM2	89.1±12.3
FM3	76.1±8.62
FM4	82.4±8.60
DN	70.8±3.34
DN1	79.4±8.45
BD	81.5±13.7
BD1	89.6±10.8

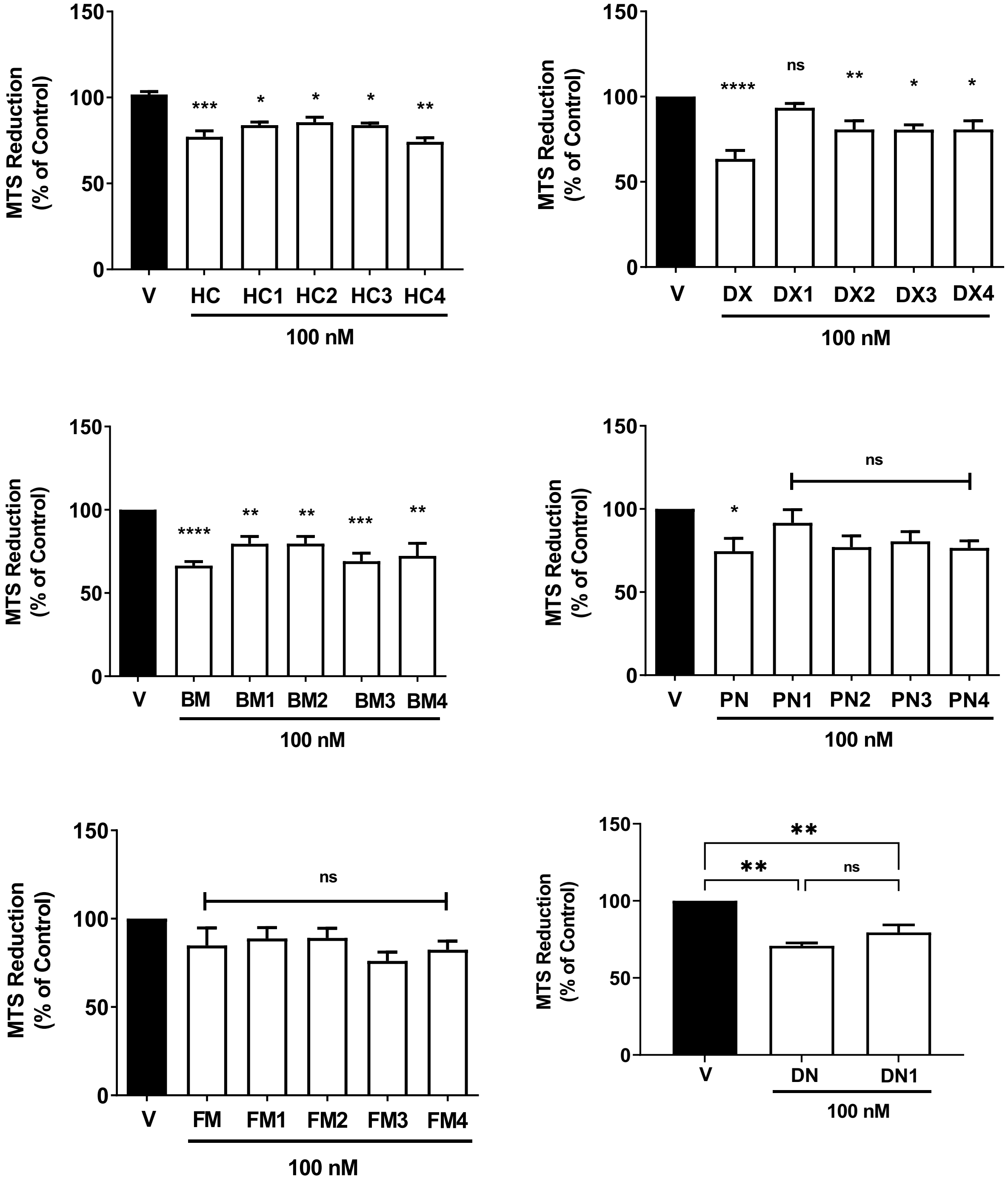
MTS Reduction (% of Control)
150
100
50
0
***
*
*
*
**
V
HC
HC1
HC2
HC3
HC4
100 nM
****
ns
**
*
*
DX
DX1
DX2
DX3
DX4
****
**
**
***
**
BM
BM1
BM2
BM3
BM4
ns
*
PN
PN1
PN2
PN3
PN4
FM
FM1
FM2
FM3
FM4
**
**
DN
DN1

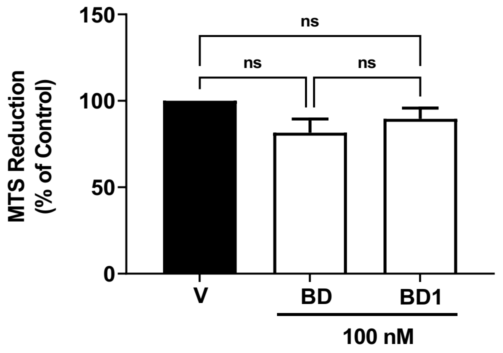
150
100
50
0
MTS Reduction
(% of Control)
ns
ns
ns
V
BD
BD1
100 nM

8. *Ccl2* and *Ccl20* Gene Expression

Table S5: *Ccl2* Assay Raw Data

	Ccl2 Relative mRNA Abundance (% IL-1β Response)
DX	18.9±8.09
HC	69.4±11.9
HC1	52.2±18.1
HC2	39.2±16.0
HC3	86.0±20.6
HC4	26.8±3.62
DX1	53.9±13.8
DX2	29.0±14.4
DX3	29.9±14.8
DX4	21.0±11.6
BM	25.0±9.23
BM1	53.3±24.8
BM2	32.7±7.77
BM3	37.1±11.3
BM4	40.4±15.6
PN	24.6±7.86
PN1	54.7±11.9
PN2	13.3±7.18
PN3	54.4±24.8
PN4	21.4±2.41
FM	19.8±7.12
FM1	33.4±11.4
FM2	34.6±8.02
FM3	38.1±25.4
FM4	21.9±12.4
DN	16.1±5.75
DN1	40.6±12.1
BD	12.1±4.07
BD1	73.8±12.7

Table S6: *Ccl20* Assay Raw Data

	Ccl20 Relative mRNA Abundance (%IL-1 β Response)
DX	23.8±12.1
HC	63.2±7.69
HC1	59.5±27.1
HC2	39.7±11.9
HC3	90.9±18.0
HC4	34.3±4.50
DX1	70.7±28.3
DX2	36.2±17.0
DX3	48.7±14.3
DX4	35.7±9.35
BM	26.4±9.85
BM1	57.4±34.8
BM2	37.3±28.2
BM3	47.6±22.7
BM4	52.6±5.59
PN	32.0±11.0
PN1	45.9±20.5
PN2	19.5±13.0
PN3	64.6±37.0
PN4	43.4±31.9
FM	22.9±9.79
FM1	32.9±24.7
FM2	35.7±5.79
FM3	38.2±19.0
FM4	28.5±16.0
DN	24.1±13.5
DN1	49.0±13.2
BD	20.7±5.43
BD1	79.8±33.8

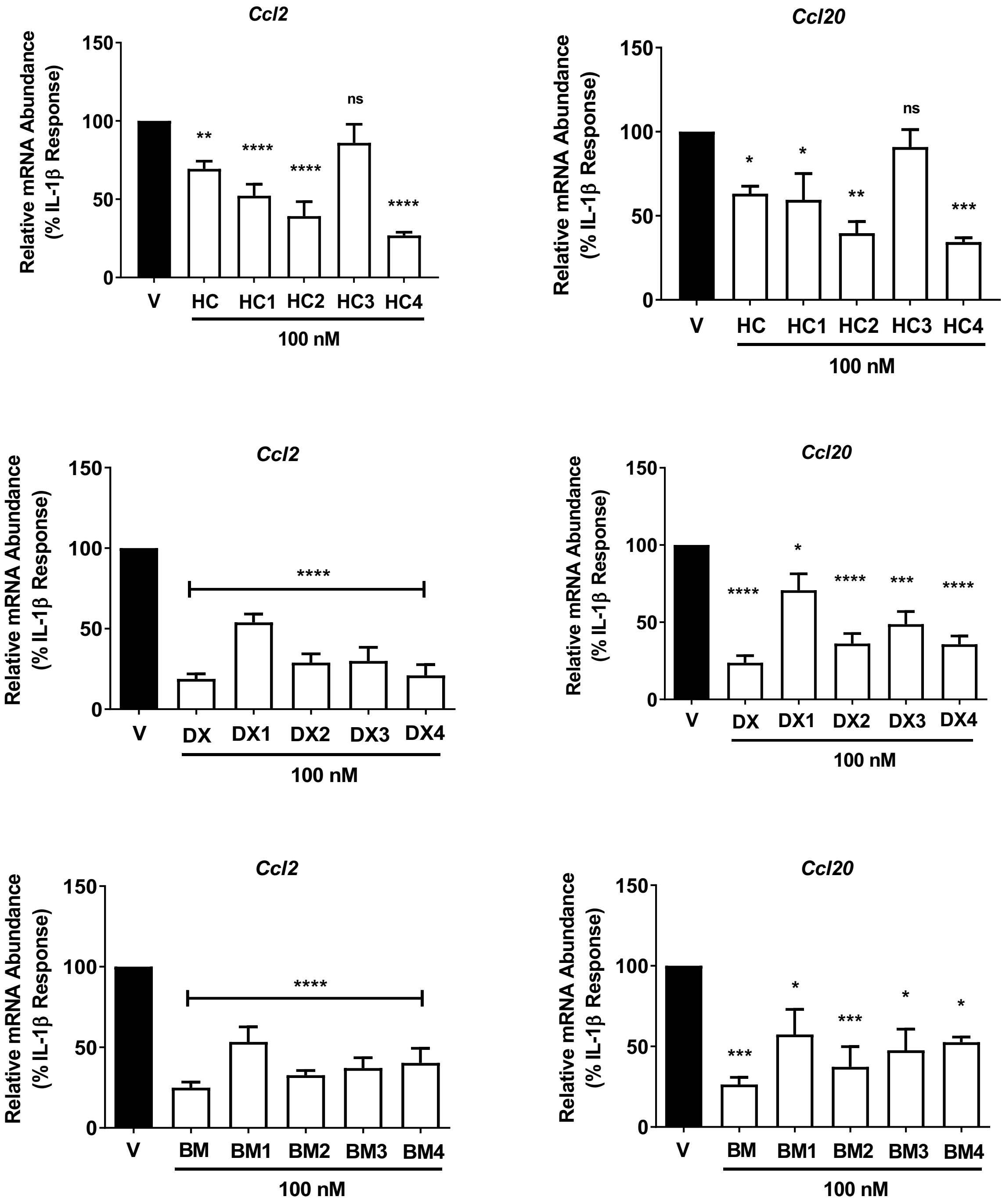
Ccl2
Relative mRNA Abundance (% IL-1β Response)
150
100
50
0
**
****
****
ns
****
V
HC
HC1
HC2
HC3
HC4
100 nM
Ccl20
Relative mRNA Abundance (% IL-1β Response)
150
100
50
0
*
*
**
ns
***
V
HC
HC1
HC2
HC3
HC4
100 nM
Ccl2
Relative mRNA Abundance (% IL-1β Response)
150
100
50
0
****
V
DX
DX1
DX2
DX3
DX4
100 nM
Ccl20
Relative mRNA Abundance (% IL-1β Response)
150
100
50
0
****
*
****
***
****
V
DX
DX1
DX2
DX3
DX4
100 nM
Ccl2
Relative mRNA Abundance (% IL-1β Response)
150
100
50
0
****
V
BM
BM1
BM2
BM3
BM4
100 nM
Ccl20
Relative mRNA Abundance (% IL-1β Response)
150
100
50
0
***
*
***
*
*
V
BM
BM1
BM2
BM3
BM4
100 nM

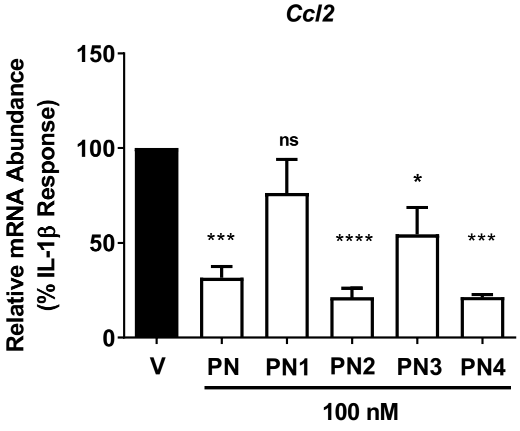
Ccl2
Relative mRNA Abundance
(% IL-1β Response)
150
100
50
0
ns
***
****
*
***
V
PN
PN1
PN2
PN3
PN4
100 nM

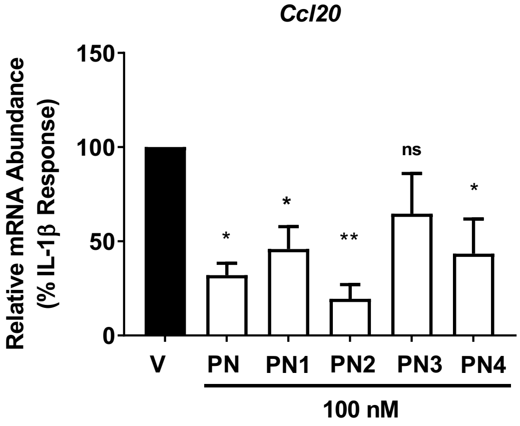
Ccl20
Relative mRNA Abundance
(% IL-1β Response)
150
100
50
0
*
*
**
ns
*
V
PN
PN1
PN2
PN3
PN4
100 nM

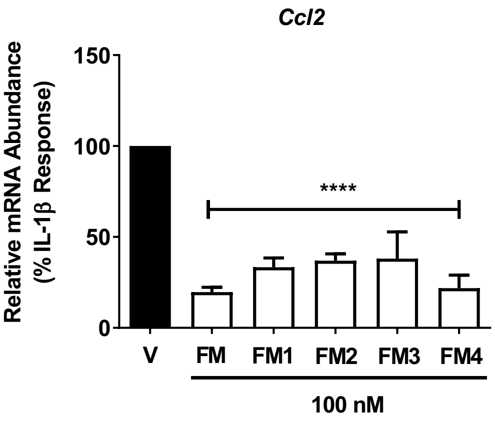
Ccl2
Relative mRNA Abundance
(% IL-1β Response)
150
100
50
0
****
V
FM
FM1
FM2
FM3
FM4
100 nM

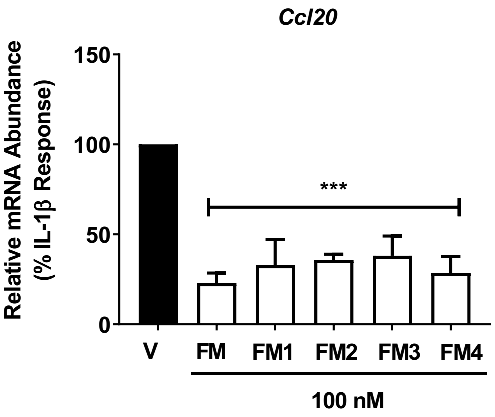
Ccl20
Relative mRNA Abundance
(% IL-1β Response)
150
100
50
0
***
V
FM
FM1
FM2
FM3
FM4
100 nM

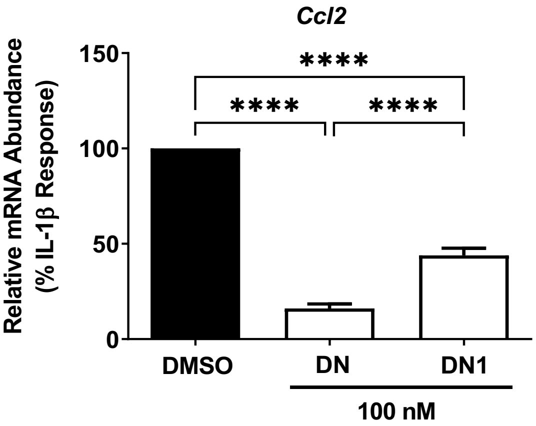
Ccl2
Relative mRNA Abundance
(% IL-1β Response)
150
100
50
0
****
****
****
DMSO
DN
DN1
100 nM

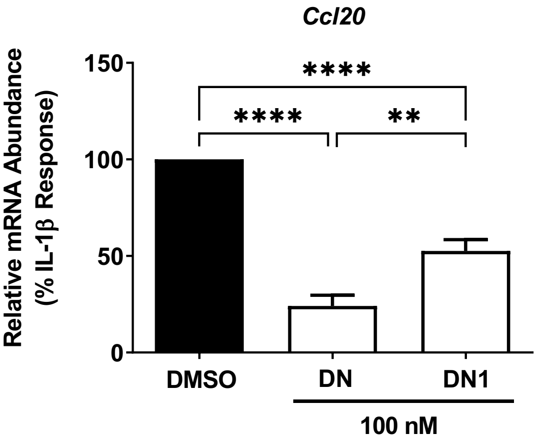
Ccl20
Relative mRNA Abundance
(% IL-1β Response)
150
100
50
0
****
****
**
DMSO
DN
DN1
100 nM

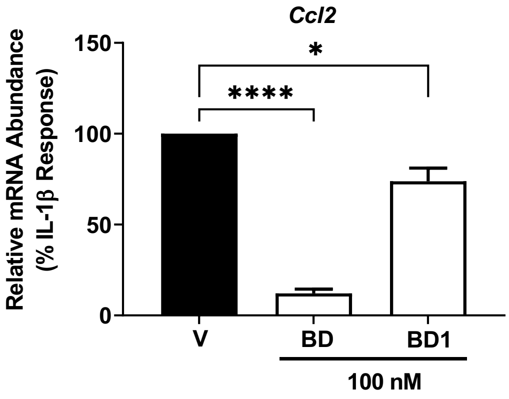
Ccl2
Relative mRNA Abundance
(% IL-1β Response)
150
100
50
0
****
*
V
BD
BD1
100 nM

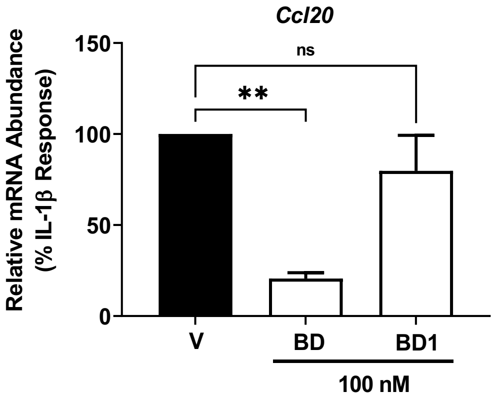
Ccl20
Relative mRNA Abundance
(% IL-1β Response)
150
100
50
0
**
ns
V
BD
BD1
100 nM

9. *Sgk1 and Rgs2 Gene Expression*

Table S7A: *Sgk1* Assay Raw Data

Sgk1 Relative mRNA Abundance (Fold over Control) – 100 nM	
DX	9.28±4.90
HC	2.69±0.22
HC1	0.97±0.19
HC2	2.18±0.56
HC3	1.17±0.39
HC4	3.05±1.65
DX1	1.35±0.52
DX2	2.86±2.62
DX3	1.54±0.34
DX4	4.77±1.13
BM	6.39±2.87
BM1	0.76±0.36
BM2	0.81±0.49
BM3	1.10±0.49
BM4	1.11±0.36
PN	4.36±3.19
PN1	1.09±0.74
PN2	1.86±1.04
PN3	1.01±0.46
PN4	4.34±1.67
FM	7.78±3.44
FM1	1.31±0.43
FM2	1.38±0.49
FM3	5.76±2.98
FM4	4.62±1.29
DN	6.65±1.48
DN1	0.57±0.29
BD	6.51±1.89
BD1	0.86±0.23

Table S8A: *Rgs2* Assay Raw Data

Rgs2 Relative mRNA Abundance (Fold over Control) – 100 nM	
DX	104±56.4
HC	4.90±0.97
HC1	1.33±1.04
HC2	4.61±3.45
HC3	2.68±1.59
HC4	31.3±8.68
DX1	9.82±9.06
DX2	37.3±37.2
DX3	33.6±12.1
DX4	71.4±14.0
BM	64.8±15.3
BM1	4.92±2.79
BM2	8.57±2.34
BM3	26.0±11.4
BM4	7.80±1.38
PN	102±43.3
PN1	2.28±0.99
PN2	50.9±16.4
PN3	4.68±2.36
PN4	52.2±17.1
FM	127±58.2
FM1	54.8±22.1
FM2	39.6±15.9
FM3	58.5±4.45
FM4	83.5±35.6
DN	82.2±18.3
DN1	3.58±2.13
BD	84.9±18.7
BD1	2.19±0.97

Table S7B: Low-Dose *Sgk1* Assay Raw Data

	Sgk1 Assay Raw Data					
	Concentration of Compound (M)					
Compd	1X10^-18	1X10^-15	1X10^-12	1X10^-9	1X10^-6	1X10^-5
DX	1.20±0.68	0.71±0.21	1.72±1.02	4.15±1.06	6.32±0.77	1.20±0.68
DX4	3.67±0.60	4.96±0.76	4.36±1.79	4.99±1.77	4.54±2.06	3.67±0.60
PN	1.17±0.36	0.77±0.06	1.75±1.23	3.81±1.60	7.51±1.87	1.17±0.36
PN4	3.43±0.29	4.87±0.82	4.30±1.44	4.75±1.26	4.37±0.49	3.43±0.29

Table S8B: Low-Dose *Rgs2* Assay Raw Data

	Rgs2 Assay Raw Data				
	Concentration of Compound (M)				
Compd	1X10^-18	1X10^-15	1X10^-12	1X10^-9	1X10^-6
DX	16.7±7.92	33.1±15.4	56.2±22.6	62.5±39.2	95.8±23.5
DX4	110±44.8	105±18.0	88.9±8.09	94.4±26.2	88.3±16.2
PN	1.02±0.45	4.70±3.70	60.7±64.7	144±53.1	153±62.8
PN4	76.2±28.6	114±37.0	106±23.6	115±59.9	170±64.6

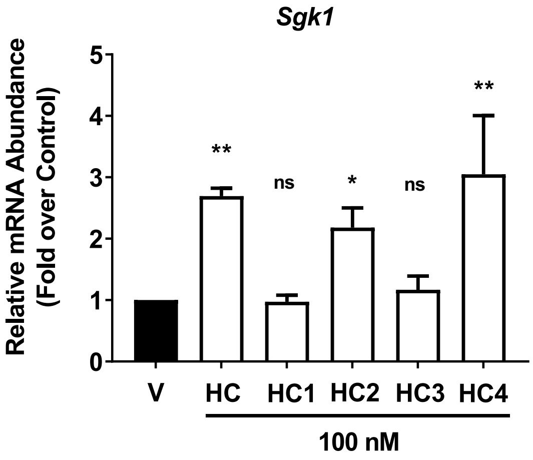

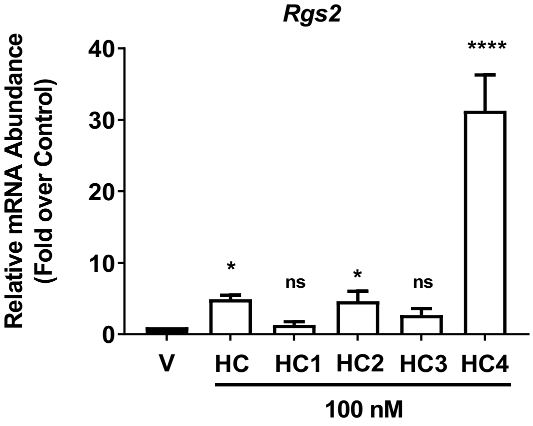

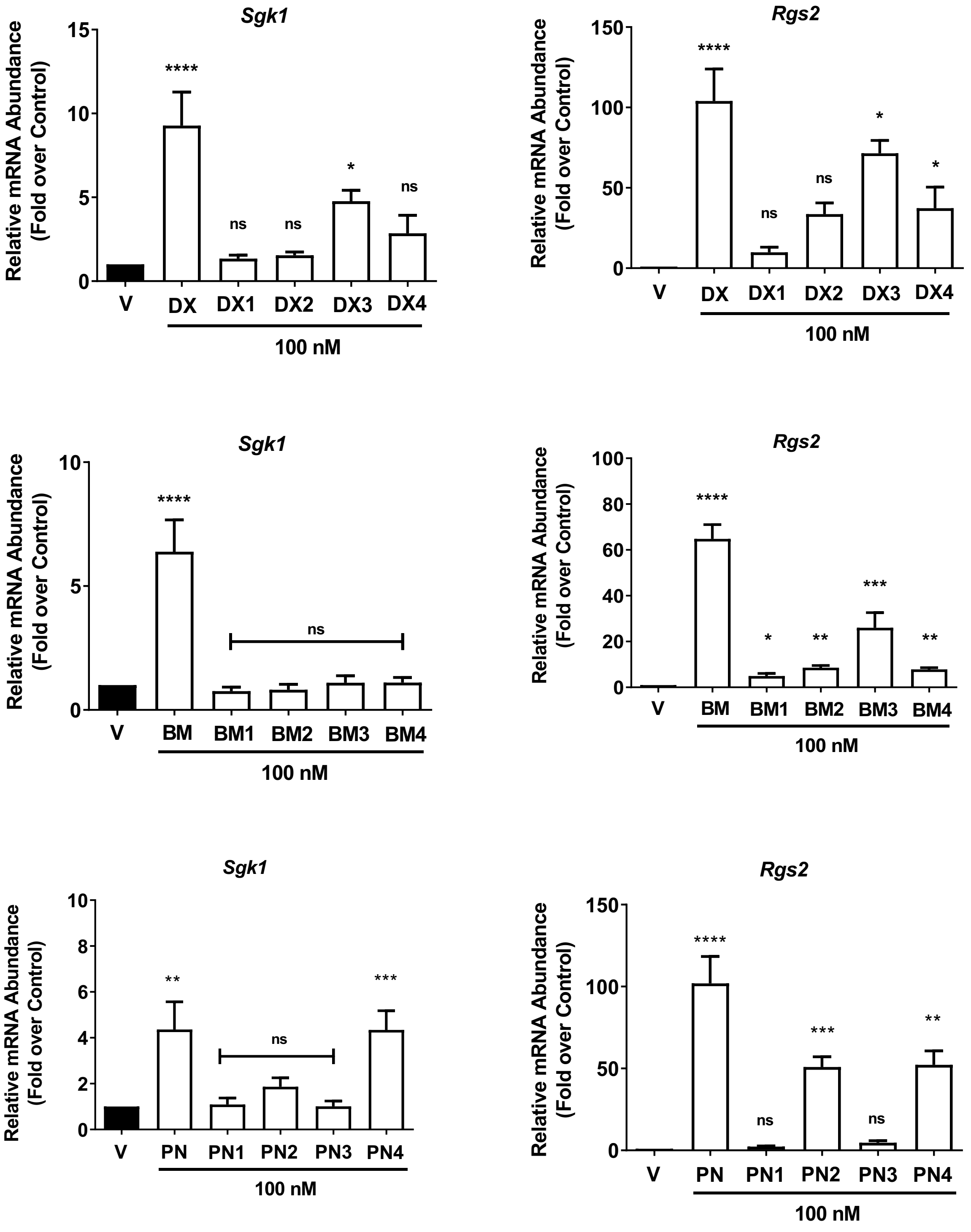
Sgk1
Relative mRNA Abundance (Fold over Control)
0
5
10
15
****
ns
ns
*
ns
V
DX
DX1
DX2
DX3
DX4
100 nM
Rgs2
Relative mRNA Abundance (Fold over Control)
0
50
100
150
****
ns
ns
*
*
V
DX
DX1
DX2
DX3
DX4
100 nM
Sgk1
Relative mRNA Abundance (Fold over Control)
0
5
10
****
ns
V
BM
BM1
BM2
BM3
BM4
100 nM
Rgs2
Relative mRNA Abundance (Fold over Control)
0
20
40
60
80
100
****
*
**
***
**
V
BM
BM1
BM2
BM3
BM4
100 nM
Sgk1
Relative mRNA Abundance (Fold over Control)
0
2
4
6
8
10
**
ns
***
V
PN
PN1
PN2
PN3
PN4
100 nM
Rgs2
Relative mRNA Abundance (Fold over Control)
0
50
100
150
****
ns
***
ns
**
V
PN
PN1
PN2
PN3
PN4
100 nM

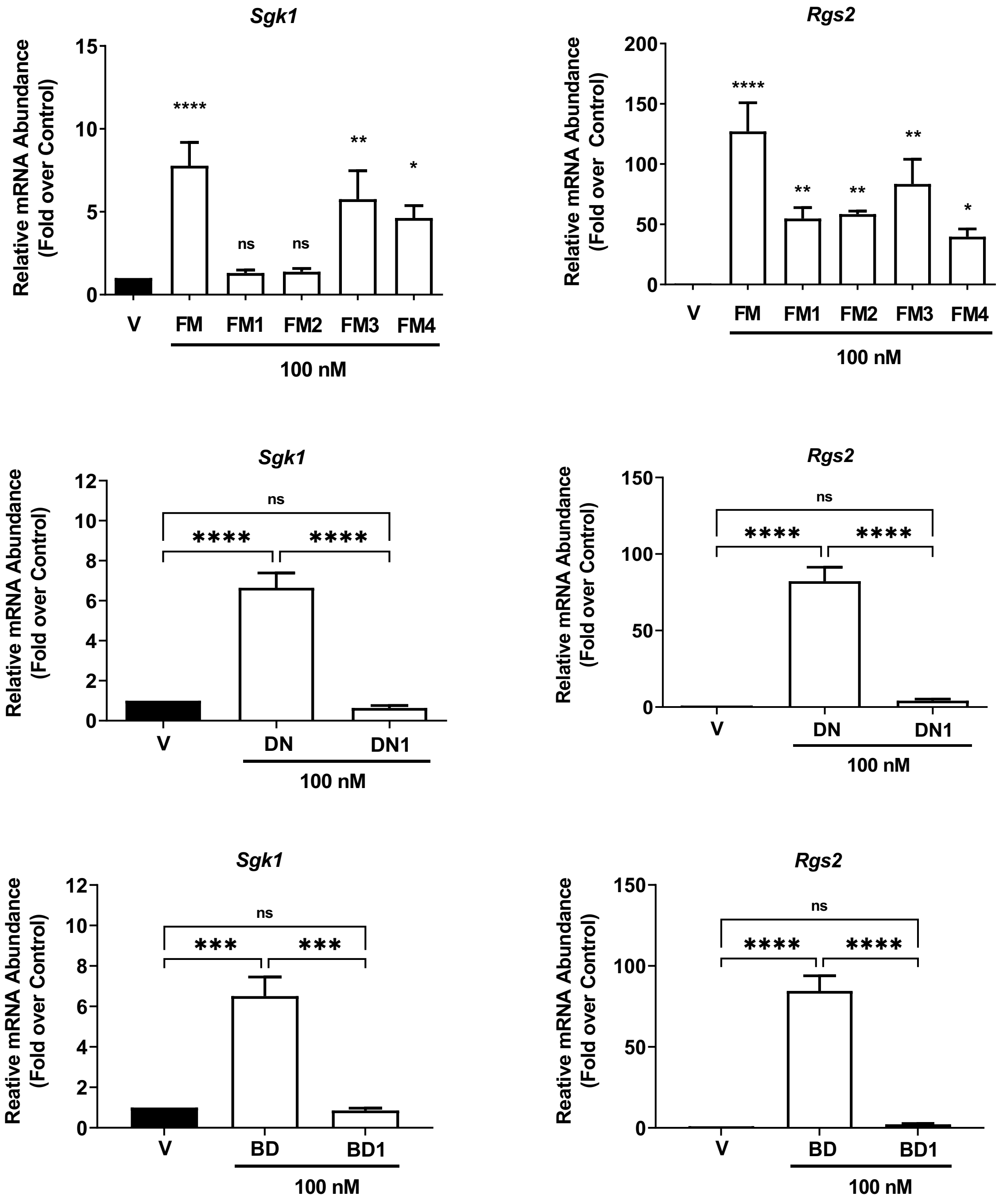
Sgk1
Relative mRNA Abundance (Fold over Control)
0
5
10
15
****
ns
ns
**
*
V
FM
FM1
FM2
FM3
FM4
100 nM
Rgs2
Relative mRNA Abundance (Fold over Control)
0
50
100
150
200
****
**
**
**
*
V
FM
FM1
FM2
FM3
FM4
100 nM
Sgk1
Relative mRNA Abundance (Fold over Control)
0
2
4
6
8
10
12
ns
****
****
V
DN
DN1
100 nM
Rgs2
Relative mRNA Abundance (Fold over Control)
0
50
100
150
ns
****
****
V
DN
DN1
100 nM
Sgk1
Reative mRNA Abundance (Fold over Control)
0
2
4
6
8
10
12
ns
***
***
V
BD
BD1
100 nM
Rgs2
Reative mRNA Abundance (Fold over Control)
0
50
100
150
ns
****
****
V
BD
BD1
100 nM

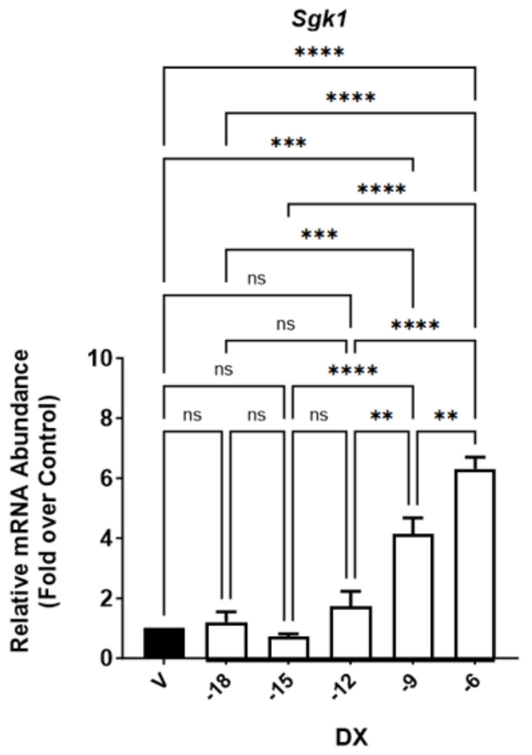
Sgk1
Relative mRNA Abundance (Fold over Control)
0 2 4 6 8 10
V -18 -15 -12 -9 -6
DX

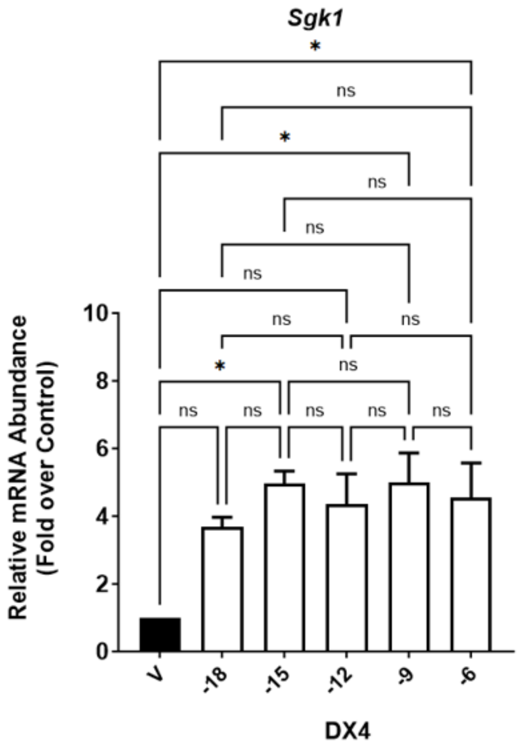
Sgk1
Relative mRNA Abundance (Fold over Control)
0 2 4 6 8 10
V -18 -15 -12 -9 -6
DX4

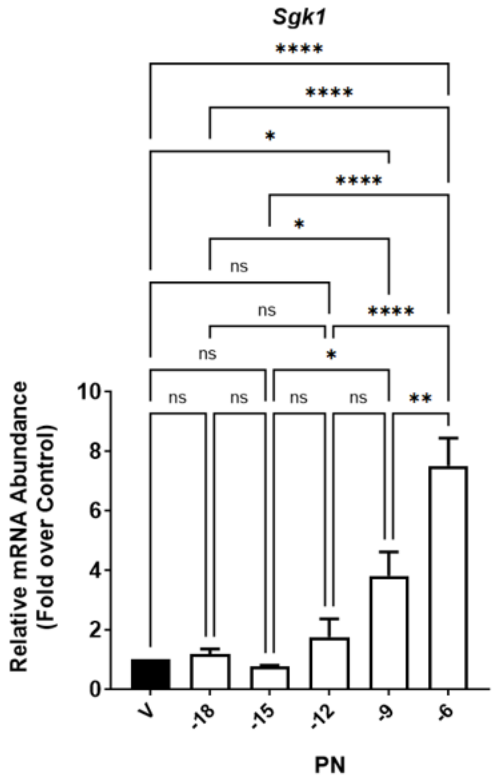
Sgk1
Relative mRNA Abundance (Fold over Control)
0 2 4 6 8 10
V -18 -15 -12 -9 -6
PN

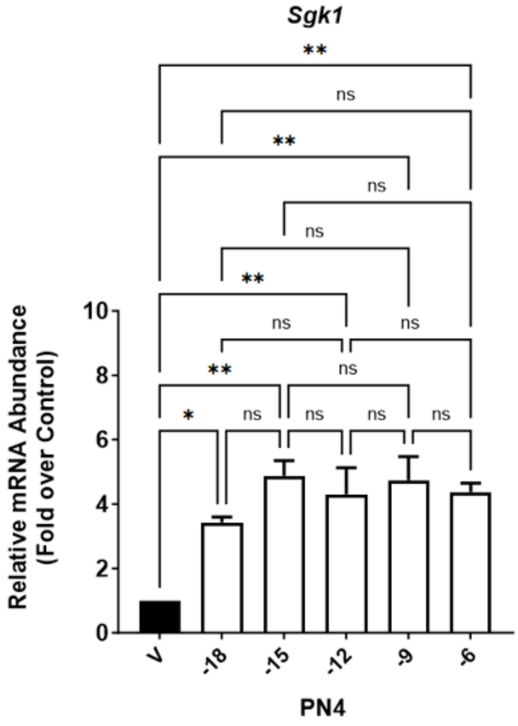
Sgk1
Relative mRNA Abundance (Fold over Control)
0 2 4 6 8 10
V -18 -15 -12 -9 -6
PN4

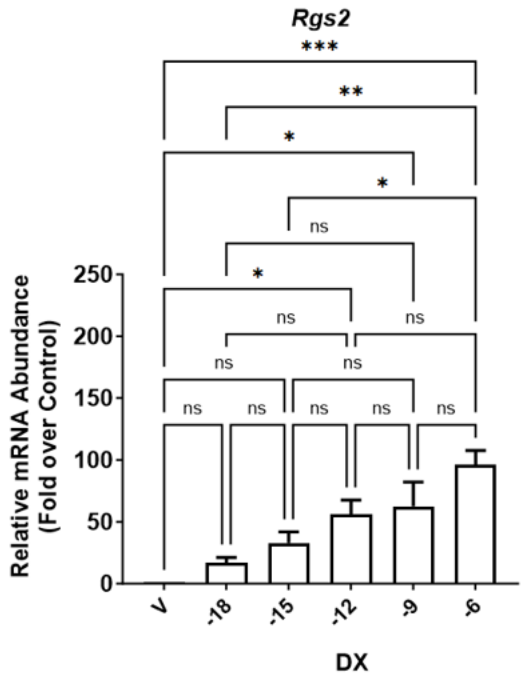
Rgs2
Relative mRNA Abundance (Fold over Control)
250
200
150
100
50
0
V
-18
-15
-12
-9
-6
DX

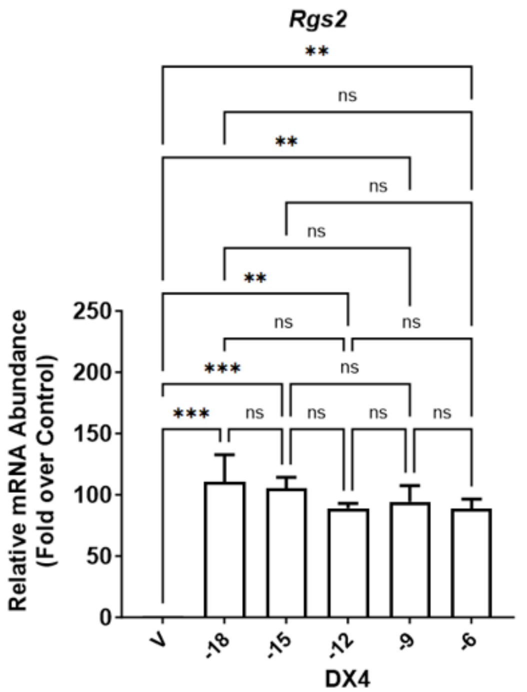
Rgs2
Relative mRNA Abundance (Fold over Control)
250
200
150
100
50
0
V
-18
-15
-12
-9
-6
DX4

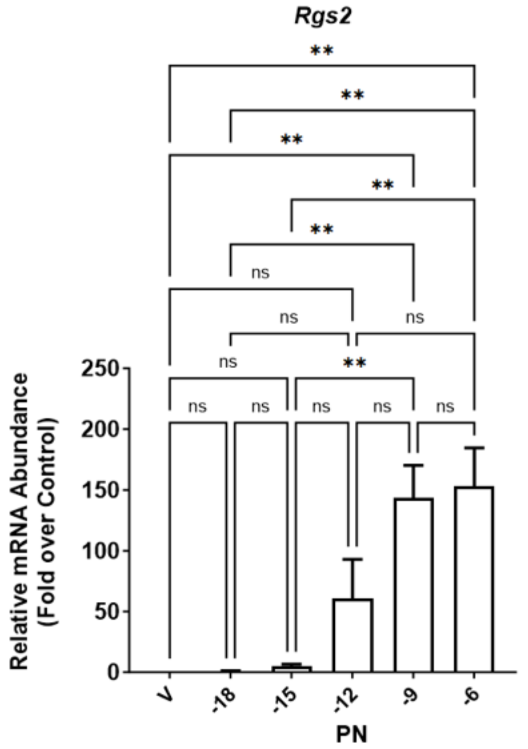
Rgs2
Relative mRNA Abundance (Fold over Control)
250
200
150
100
50
0
V
-18
-15
-12
-9
-6
PN

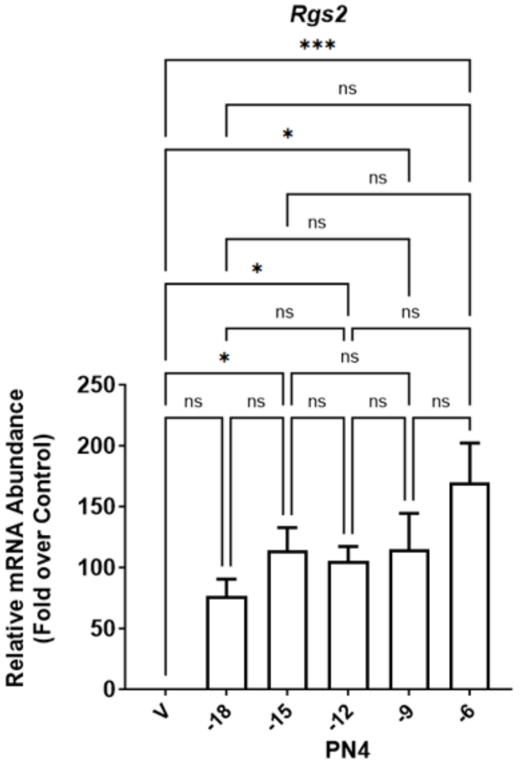
Rgs2
Relative mRNA Abundance (Fold over Control)
250
200
150
100
50
0
V
-18
-15
-12
-9
-6
PN4

10. Protein backbone RMSD plots of 7prv LBD1 with steroids and DAC in 3bqd throughout the MD trajectory of 100 ns

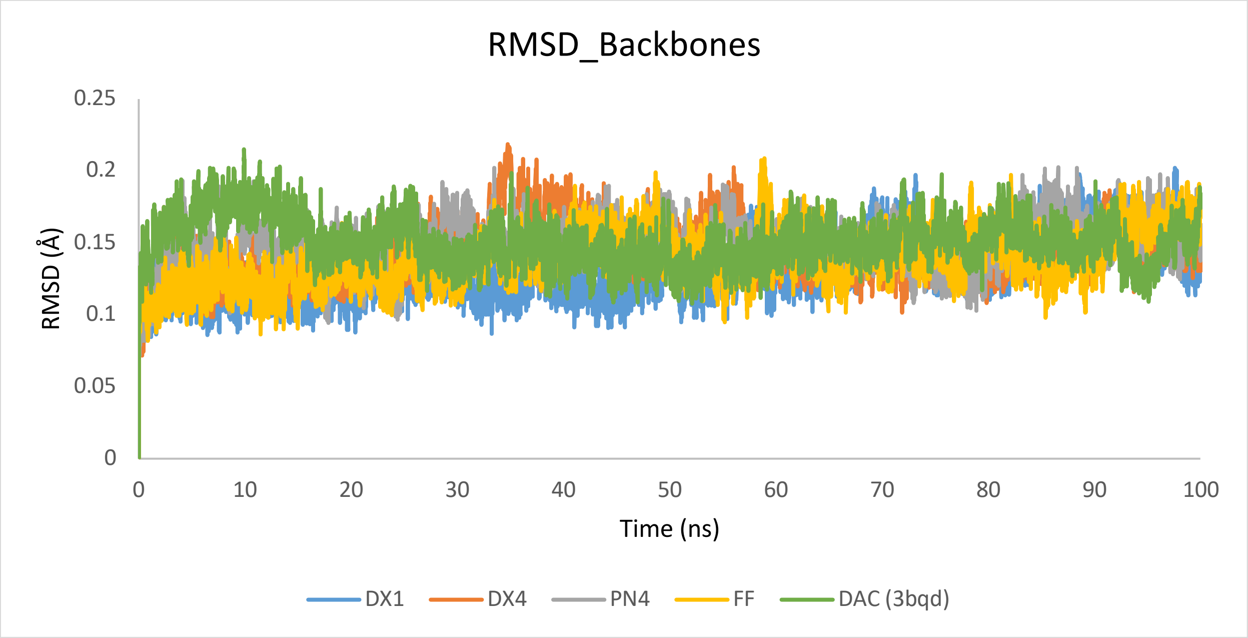

11. The ligand heavy atom RMSD plots throughout the MD trajectory of 100 ns

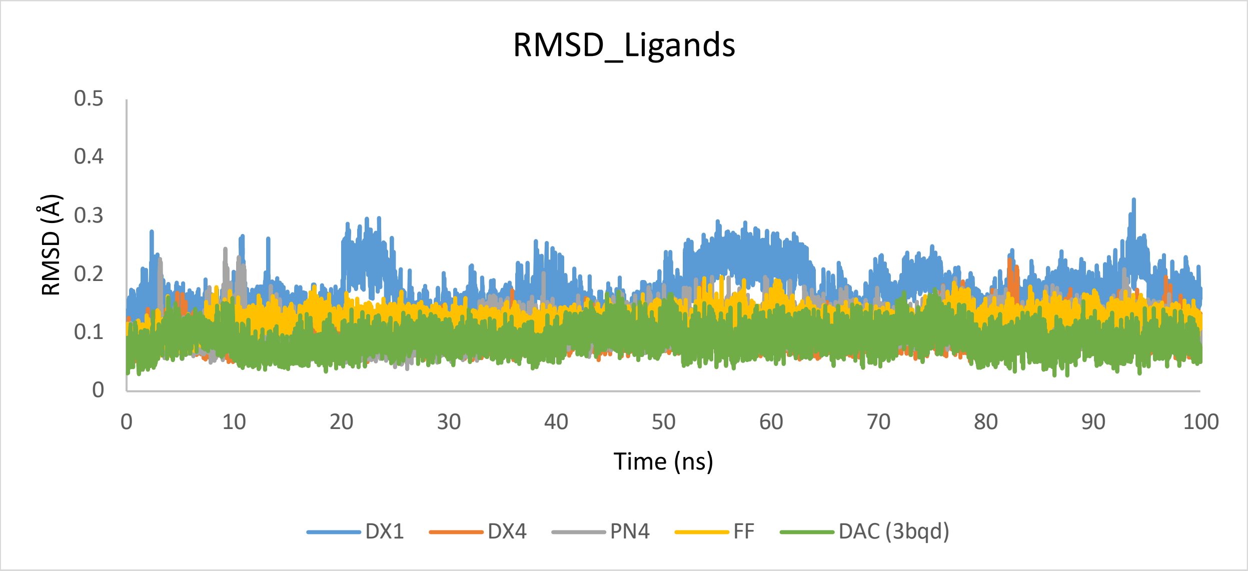

12. RMSF plot of ligand bound to 7prv LBD1 and DAC in 3bqd

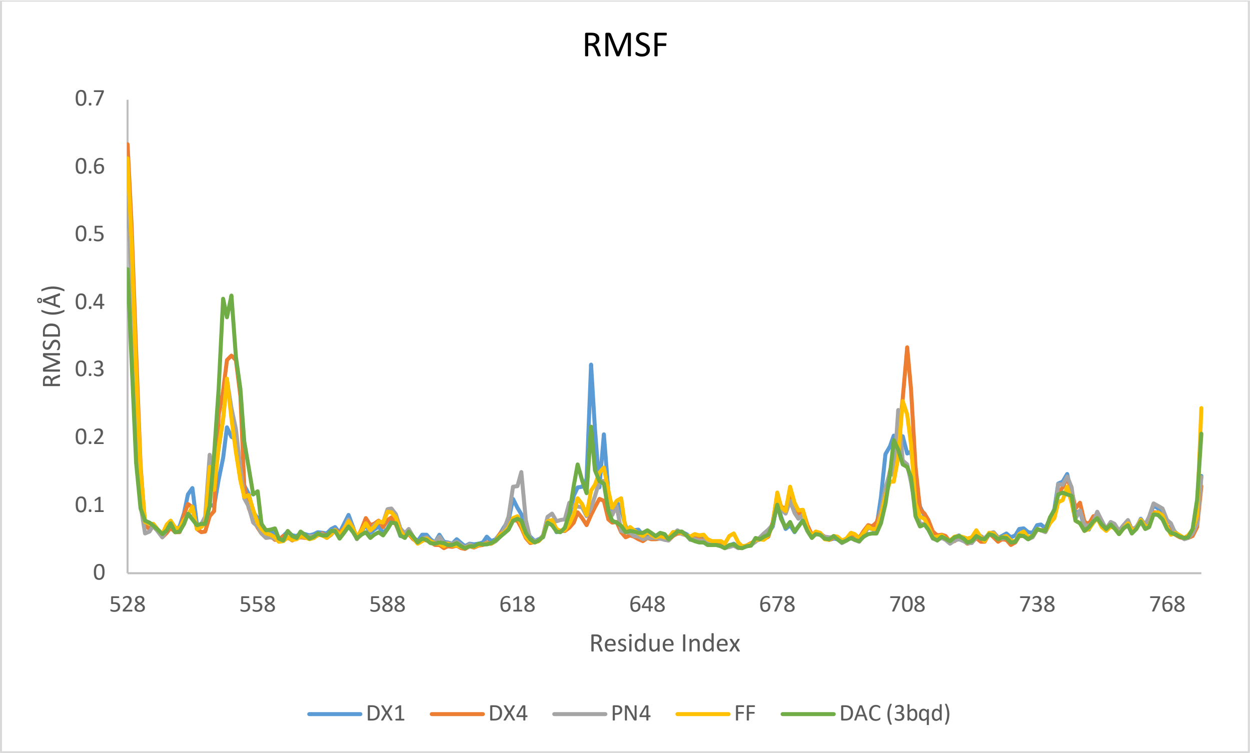

13. Relative Binding Affinities of Compounds Before and After 100 ns Simulations

Table S9: Relative Binding Affinities of Docked Compounds

	Relative Affinity (kcal/mol)	
Compd	Initial	Final
FF	-13.0	-12.0
DX1	-9.4	-12.1
DX4	-12.2	-10.2
PN4	-11.4	-10.5
DAC	-14.7	-12.9

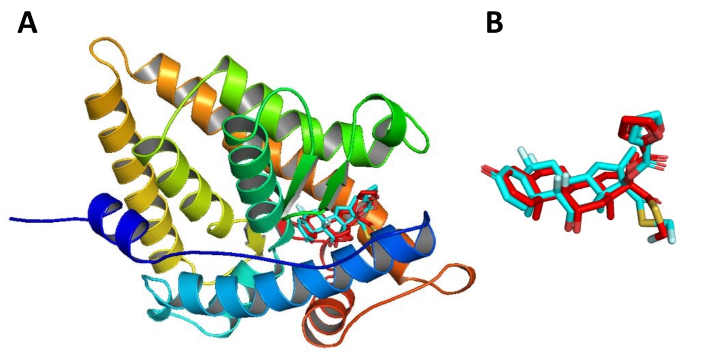

Figure S1. Confirming the reliability of docking process. (A) Docking of the native ligand fluticasone furoate (FF) in LBD1 of 7prv. (B) Zoomed in image of the native ligand crystal (red) and docked FF (blue).

Table S10: Pocket Volume Calculations

Compd	Molecular Surface Pocket Volume (Å^3)	Solvent Accessible Surface Pocket Volume (Å^3)
**FF**	1478.512	333.640
**DX4**	1876.419	484.204
**PN4**	2227.703	546.057
**DAC**	1945.266	463.038
